# Chiral Phosphoric Acid-Catalyzed Enantioselective Pictet–Spengler Reaction for Concise Synthesis of CF_3_‑Substituted Tetrahydro-β-Carbolines

**DOI:** 10.1021/acs.orglett.5c01864

**Published:** 2025-06-03

**Authors:** Shigenobu Umemiya, Shinnosuke Nara, Masahiro Terada

**Affiliations:** † Research and Analytical Center for Giant Molecules, Graduate School of Science, 208508 Tohoku University, 6-3 Aramaki Aza Aoba, Aoba-ku, Sendai 980-8578, Japan; ‡ Department of Chemistry, Graduate School of Science, Tohoku University, Sendai 980-8578, Japan

## Abstract

Fluorine-containing compounds are widely found in pharmaceuticals, agrochemicals, and functional materials. In particular, compounds containing enantioenriched trifluoromethyl groups are essential because they show metabolic stability, lipophilicity, and membrane permeability in vivo. In this study, an enantioselective Pictet–Spengler reaction of tryptamine derivatives with 1,1,1-trifluoro-4-[tris­(1-methylethyl)­silyl]-3-butyn-2-one catalyzed by a chiral phosphoric acid is demonstrated, which provides CF_3_-containing chiral tetrahydro-β-carboline derivatives in good yields with high enantioselectivities.

## Graphical abstract

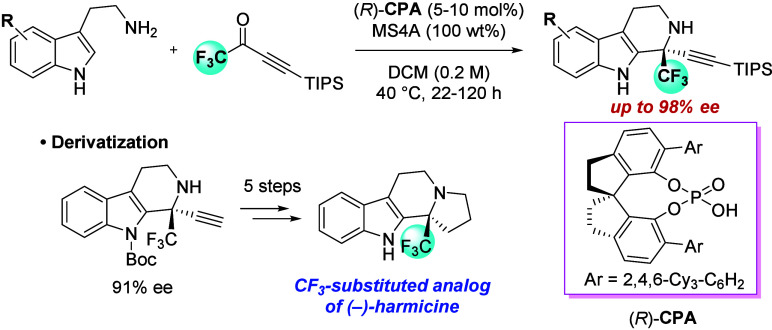

Fluorine-containing compounds are vital in modern society because of their widespread applications in pharmaceuticals, agrochemicals, polymers, and other chemicals.^1^ In particular, compounds containing trifluoromethyl groups exhibit excellent properties, including metabolic stability, lipophilicity, and membrane permeability in vivo.^2^ Because of their significance in medicinal chemistry, much effort has been channeled into the development of methodologies for the efficient synthesis of chiral building blocks containing trifluoromethyl groups.^3^ For instance, *N*-heterocyclic molecules containing trifluoromethyl groups are an important class of compounds that have attracted immense interest in drug discovery studies.^4^

Many natural products and pharmaceuticals have a tetrahydro-β-carboline skeleton.^5^ These molecules are indispensable in medicinal chemistry because of their potent biological activities.^6^ Recently, tetrahydro-β-carboline compounds with trifluoromethyl groups have captured the attention of medicinal chemists owing to their unique properties and biological activities (Figure 1).^7^ Tremendous effort has been devoted to developing methods for synthesizing tetrahydro-β-carboline derivatives containing trifluoromethyl groups in modern organic chemistry, and several racemic and chiral synthetic approaches have been reported. On the other hand, advances in catalytic and enantioselective methods for constructing these frameworks remain limited.^8^ Lin and co-workers demonstrated a chiral phosphoric acid (CPA)-catalyzed enantioselective aza-Friedel–Crafts reaction of 3,4-dihydro-β-carboline bearing a trifluoromethyl group for the synthesis of a CF_3_-containing chiral tetrahydro-β-carboline derivative (Scheme 1a).^9^ The Nakamura group reported a CPA II-promoted reaction of an α-ketoester having a CF_3_ group, which afforded the desired product in high yield but with low enantioselectivity (Scheme 1b).^10^ Despite the apparent importance of enantioenriched tetrahydro-β-carbolines bearing CF_3_ groups in drug discovery, there are few efficient catalytic and enantioselective methods for their synthesis.

**Figure 1:**
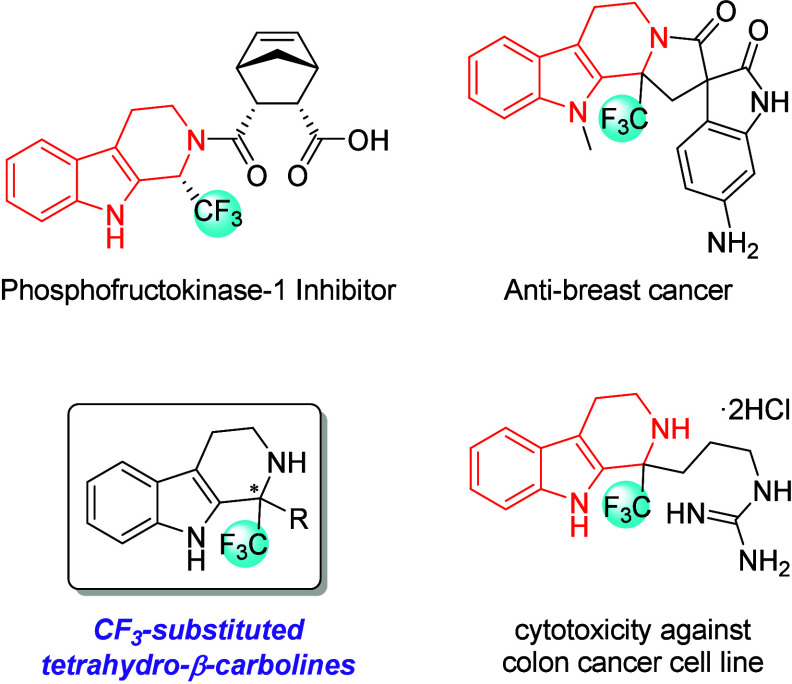
Structures of biologically active tetrahydro-β-carbolines having a CF_3_ group.

**Scheme 1:**
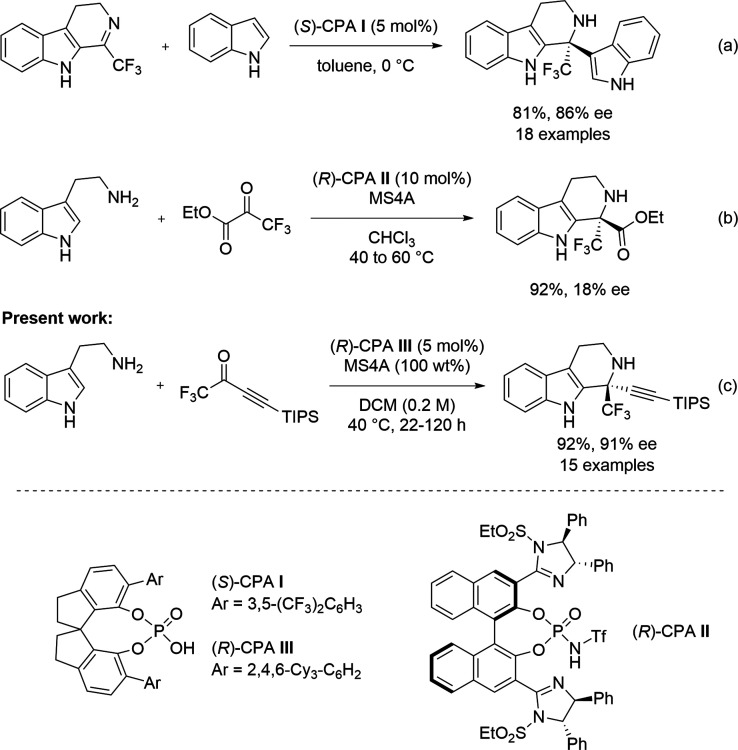
Enantioselective Synthesis of CF_3_-Substituted Tetrahydro-β-Carboline Skeleton

Aside from their versatility, CPAs are moisture- and air-stable; hence, they are easy to handle. We have developed enantioselective transformations that utilize the excellent reactivity and selectivity of CPAs.^11^ Several CPA-catalyzed Pictet–Spengler reactions of ketones have been reported in the past two decades, primarily because CPAs are one of the most attractive catalysts for the synthesis of enantioenriched tetrahydro-β-carbolines bearing a tetrasubstituted chiral center.^10,12^ However, highly enantioselective Pictet–Spengler reactions of trifluoromethyl ketones are limited to the iso-Pictet–Spengler reaction developed by Lin and co-workers.^13^ To the best of our knowledge, a highly enantioselective Pictet–Spengler reaction employing trifluoromethyl ketone derivatives to construct tetrahydro-β-carbolines bearing a CF_3_-substituted quaternary stereocenter under catalytic conditions has not yet been established. This is primarily due to the inherently low reactivity of trifluoromethyl ketones and their corresponding imines compared to aldehydes, which arises from steric hindrance at the reaction site. Herein, we report an enantioselective CPA-catalyzed Pictet–Spengler reaction of trifluoromethyl ketone containing an electron-withdrawing triple bond with tryptamine (Scheme 1c). We also demonstrated various derivatizations of the obtained products and the construction of not only CF_3_-containing tetrahydro-β-carboline derivatives but also a tetracyclic natural product analog.

First, we investigated the enantioselective Pictet–Spengler reaction of tryptamine **1a** with trifluoromethyl ketone **2a** having a Ph group at the terminus of the triple bond using SPINOL-derived CPA^14^ (*R*)-**3a** (TRIP = 2,4,6-^*i*^Pr_3_–C_6_H_2_) (Table 1). Only the 1,4-addition reaction of **1a** with **2a** proceeded without the formation of desired product **4aa** (entry 1). We performed the reaction using ketone **2b** with a bulky TBS group at the alkyne terminus instead of a Ph group to suppress the undesired 1,4-addition reaction. As expected, the steric hindrance of the TBS group effectively suppressed the side reaction, resulting in a dramatic improvement of the yield to 52%. The desired product was obtained with good enantioselectivity (entry 2). When ketone **2c** bearing a bulkier TIPS group was used as the substrate, the unwanted 1,4-addition reaction was completely suppressed, and the desired product was obtained in excellent yield with high enantioselectivity (entry 3). We also investigated the substituents of the catalyst, as shown in entries 4 to 6. When catalyst (*R*)-**3b** (Ar = 2,4,6-Cy_3_-C_6_H_2_) was utilized, **4ac** was obtained in 88% yield with 90% ee (entry 4).^15^ Other substituents, such as 2-naphthyl and 9-phenanthryl, failed to improve the enantioselectivity (entries 5 and 6). When the backbone of the catalysts was changed from SPINOL to BINOL having the same TRIP substituent, the enantioselectivity was significantly decreased from 81% ee to 42% ee (entry 3 vs entry 7). Drastic decreases in the reaction speed and enantioselectivity were observed when toluene, CHCl_3_, MeCN, or THF was used (entries 8–11). When the reaction was carried out under the conditions in entry 4 with the addition of MS4A, both chemical yield and enantioselectivity were slightly enhanced. Therefore, these were determined to be the optimum conditions (entry 12). Finally, the reaction was performed on a 1 mmol scale to afford the product in 95% yield without loss of enantioselectivity (entry 13).

**Table 1:**
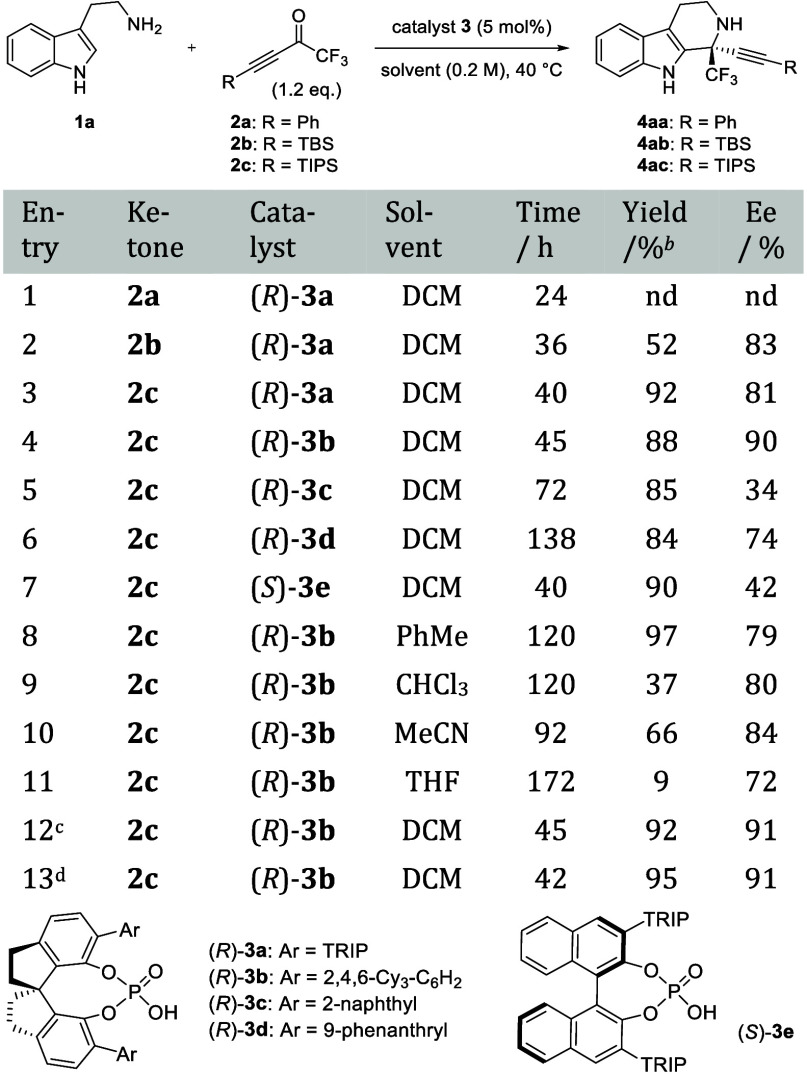
Optimization of Reaction Conditions^a^

a Unless otherwise specified, all reactions were carried out using 0.20 mmol of **1a**, 0.24 mmol (1.2 equiv) of **2**, and 0.010 mmol (5 mol %) of catalyst. TRIP = 2,4,6-^*i*^Pr_3_C_6_H_2_, nd = not determined, PhMe = toluene.

b Isolated yield.

c The reaction was performed with MS4A (100 wt % to **1a**: 16 mg).

d The reaction was performed on a 1 mmol scale.

With the optimum conditions in hand, we proceeded to examine the substrate scope of the present reaction (Figure 2). When substrate **1b** having an electron-donating MeO group at the 5-position of the aromatic ring was used, the desired product was obtained in high yield with high enantioselectivity. The corresponding enantioenriched products were also generated with good enantioselectivity for substrates with Me, fluorine, chlorine, and bromine groups substituted at the 5-position. In contrast, the substrate with an electron-withdrawing CF_3_ group afforded **4g** with a drastically reduced yield but with good enantioselectivity, and the corresponding imine was obtained in moderate yield. We presumed that the low yield was due to the reduced nucleophilicity of the indole moiety caused by the CF_3_ group. When the reaction was carried out using substrate **1h** with a MeO group substituted at the 4-position, product **4h** was obtained in good yield and with excellent enantioselectivity. The reaction of **1i** with a Br group at the 4-position proceeded very slowly, giving the desired product in only 18% yield but with excellent enantioselectivity. Examining tryptamine derivatives with substituents at the 6-position revealed that **1j** and **1k** having an electron-donating group afforded products **4j** and **4k** in good yields with high enantioselectivities. Unfortunately, substrate **1l** having a CF_3_ group at the 6-position was not suitable for the present reaction, affording a product in low yield with moderate enantioselectivity. On the other hand, substrates with halogen substituents, such as F, Cl, and Br, were tolerant, furnishing desired products with high enantioselectivities even though 10 mol % of catalyst was needed to complete the reactions using **1n** and **1o**. Although the Pictet–Spengler reaction tended to be sluggish and to give low yields when electron-deficient tryptamine derivatives (**1g**, **1l**) were utilized as substrates, it was widely applicable to electron-rich and electron-neutral substrates.

**Figure 2:**
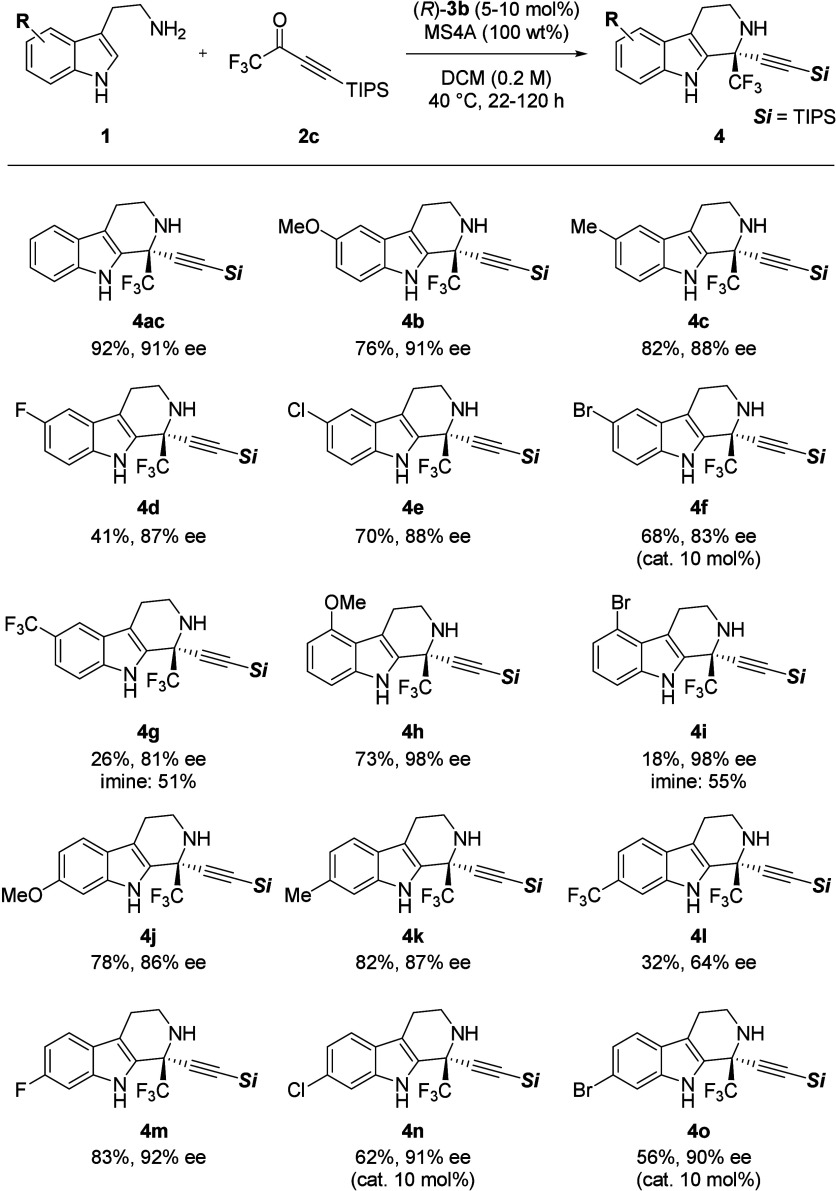
Substrate scope.

Because the reaction of **1i** having a Br group at the 4-position almost stopped at the intermediate imine step under the optimum conditions, we examined the conversion of isolated imine **5i** into the desired product (Scheme 2). To our delight, the reaction of imine **5i** proceeded smoothly in the presence of catalyst (*R*)-**3b** to afford a cyclized product in high yield with excellent enantioselectivity.^16^ On the other hand, when the reaction was performed with tryptamine **1i** and ketone **2c**, the desired product was obtained in a very low yield of 18%. This difference can be attributed to catalyst deactivation. Specifically, it has been hypothesized that the reaction between metal ions in the molecular sieves and the phosphoric acid catalyst would form metal salts of the corresponding phosphoric acids, which are inactive for the intramolecular cyclization.^17^ In the case of substrate **1i**, the reaction almost stopped at the intermediate imine step because the catalyst was deactivated by the long reaction time.

**Scheme 2:**
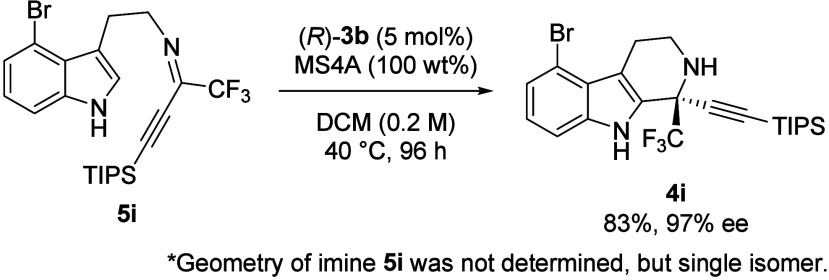
Cyclization Reaction of Isolated Imine **5i** Promoted by (*R*)-**3b**

We proceeded to derivatize the obtained products into useful chiral building blocks (Scheme 3). Selective Boc-protection of **4ac** under standard conditions, followed by desilylation using TBAF, afforded *N*-Boc indole **6**. Treatment of **4ac** with TBAF directly resulted in the deprotection of the TIPS group, generating terminal alkyne **7** in 98% yield without any loss of enantiopurity. **7** was subjected to hydrogenation with a Lindlar catalyst to afford terminal alkene **8** in good yield. The Sonogashira–Hagihara cross-coupling reaction of terminal alkyne **7** with iodobenzene provided product **9** in good yield under standard conditions. Saturation of the terminal alkyne moiety was accomplished by using a catalytic amount of Pd/C under H_2_ to furnish desired product **10** in excellent yield. Fortunately, these reactions were conducted without the erosion of enantiomeric purity.

**Scheme 3:**
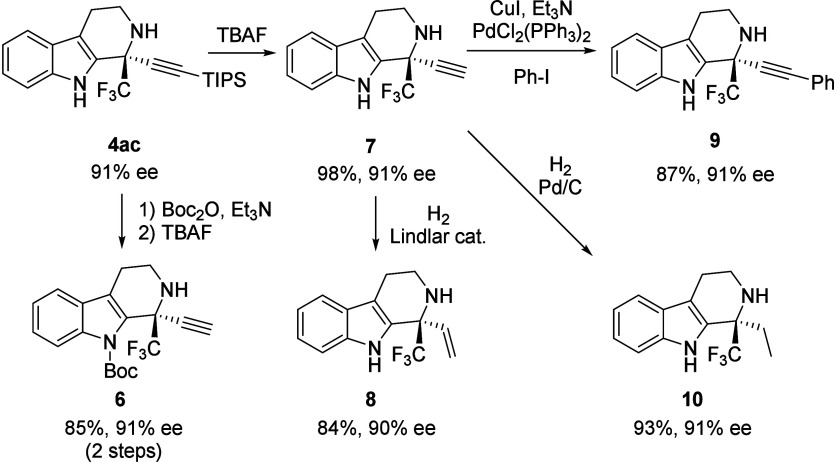
Derivatization of Product **4ac**

Next, terminal alkyne **6** was derivatized further, as shown in Scheme 4. Alkylation of the amine moiety of **6** with allyl bromide was performed under mild basic conditions to obtain *N*-allyl-protected product **11** in moderate yield. A two-step reaction involving the treatment of **6** with H_2_ and Lindlar catalyst, followed by hydroboration, gave primary alcohol **13** in 65% yield (2 steps). After terminal alkene **12** was converted into *N*-allyl product **14**, a three-step transformation that included an intramolecular olefin metathesis,^18^ deprotection of the Boc group, and reduction of the double bond gave tetracyclic compound **15**, a CF_3_-substituted analog of (−)-harmicine.^19^ These transformations were possible without the loss of enantiomeric purity. The derivatization reactions concisely provided trifluoromethyl-substituted enantioenriched tetrahydro-β-carboline derivatives, demonstrating the synthetic utility of the present Pictet–Spengler reaction for constructing functional chiral building blocks.

**Scheme 4:**
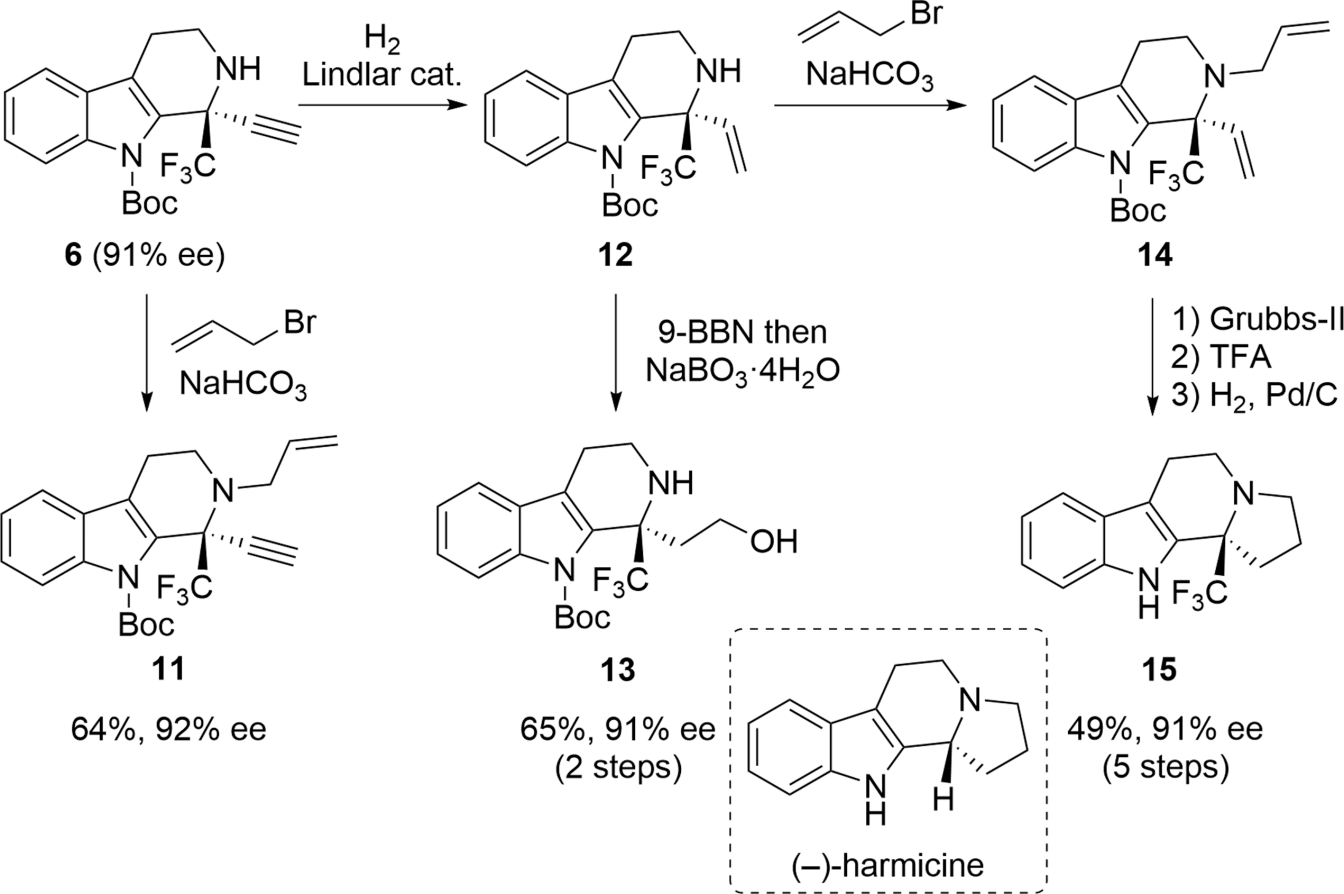
Derivatization of **6** and Synthesis of (−)-Harmicine Analog **15**

In conclusion, we have developed an enantioselective Pictet–Spengler reaction of tryptamines with trifluoromethyl ketones bearing triple bonds promoted by CPA (*R*)-**3b** to give synthetically useful tetrahydro-β-carboline derivatives substituted with trifluoromethyl groups in a highly enantioselective manner. In this reaction, tryptamine derivatives with electron-rich and electron-neutral aromatic rings can be converted into the desired products in good yields with high enantioselectivities. Although the reaction tended to stall at the intermediate imine step when a tryptamine unit bearing an electron-withdrawing group, such as a CF_3_ group, was employed, we found that the cyclization of isolated imine **5i** in the presence of CPA (*R*)-**3b** proceeded smoothly to afford tetrahydro-β-carboline **4i** in high yield with excellent enantioselectivity. The present CPA-catalyzed Pictet–Spengler reaction can efficiently synthesize tetrasubstituted carbon centers having a CF_3_ group. The synthetic utility of the obtained products was demonstrated through chemoselective transformations, including the synthesis of (−)-harmicine analog **15**, without any loss of enantiopurity. Further studies on the mechanistic insights and other enantioselective Pictet–Spengler reactions using ketones are in progress in our laboratory.

## Supplementary Material



## Data Availability

The data underlying this study are available in the published article and its Supporting Information.

## Notes

Experimental procedure and characterization data (PDF)

## References

[1] Müller K., Faeh C., Diederich F. (2007). Fluorine in
Pharmaceuticals: Looking Beyond Intuition. Science.

[2] Gillis E. P., Eastman K. J., Hill M. D., Donnelly D. J., Meanwell N. A. (2015). Applications
of Fluorine in Medicinal Chemistry. J. Med.
Chem..

[3] Prakash G. K. S., Yudin A. (1997). Perfluoroalkylation
with Organosilicon Reagents. Chem. Rev..

[4] Corbett J. W., Ko S. S., Rodgers J. D., Gearhart L. A., Magnus N. A., Bacheler L. T., Diamond S., Jeffrey S., Klabe R. M., Cordova B. C., Garber S., Logue K., Trainor G. L., Anderson P. S., Erickson-Viitanen S. (2000). Inhibition
of Clinically Relevant Mutant Variants of HIV-1 by Quinazolinone Non-Nucleoside
Reverse Transcriptase Inhibitors. J. Med. Chem..

[5] Somei M., Yamada F. (2004). Simple indole alkaloids and those with a nonrearranged
monoterpenoid unit. Nat. Prod. Rep..

[6] Laine A. E., Lood C., Koskinen A. M. P. (2014). Pharmacological
Importance of Optically
Active Tetrahydro-β-carbolines and Synthetic Approaches to Create
the C1 Stereocenter. Molecules.

[7] Kakehi R., Kobayashi H., Mashiyama H., Yajima H., Koyama H., Ito T. K., Yoshida M., Nagaoka Y., Sumiyoshi T. (2025). Asymmetric
Synthesis, Structure Determination,
and Biologic Evaluation of Isomers of TLAM as PFK1 Inhibitors. ACS Med. Chem. Lett..

[8] Wang L.-N., Shen S.-L., Qu J. (2014). Simple and efficient
synthesis of tetrahydro-β-carbolines via the Pictet–Spengler
reaction in 1,1,1,3,3,3-hexafluoro-2-propanol (HFIP). RSC Adv..

[9] Xie E., Rahman A., Lin X. (2017). Asymmetric synthesis of CF_3_- and indole-containing tetrahydro-β-carbolines via chiral
spirocyclic phosphoric acid-catalyzed aza-Friedel–Crafts reaction. Org. Chem. Front..

[10] Nakamura S., Matsuda Y., Takehara T., Suzuki T. (2022). Enantioselective Pictet–Spengler
Reaction of Acyclic α-Ketoesters Using Chiral Imidazoline-Phosphoric
Acid Catalysts. Org. Lett..

[11] Akiyama T., Itoh J., Yokota K., Fuchibe K. (2004). Enantioselective Mannich-Type Reaction Catalyzed by
a Chiral Brønsted Acid. Angew. Chem., Int.
Ed..

[12] Muratore M. E., Holloway C. A., Pilling A. W., Storer R. I., Trevitt G., Dixon D. J. (2009). Enantioselective
Brønsted Acid-Catalyzed *N*-Acyliminium Cyclization
Cascades. J. Am. Chem. Soc..

[13] Li X., Chen D., Gu H., Lin X. (2014). Enantioselective synthesis of benzazepinoindoles bearing trifluoromethylated
quaternary stereocenters catalyzed by chiral spirocyclic phosphoric
acids. Chem. Commun..

[14] Xu F., Huang D., Han C., Shen W., Lin X., Wang Y. (2010). SPINOL-Derived Phosphoric
Acids: Synthesis and Application in Enantioselective Friedel–Crafts
Reaction of Indoles with Imines. J. Org. Chem..

[15] The absolute configuration of **4ac** was determined to be (*R*) after deprotection of the TIPS group to afford alkyne **7** (Scheme 3) by single-crystal X-ray diffraction analysis.

[16] The geometry of **5i** was not determined, but the DTF calculation suggested that *trans* imine **5i** was more stable than the *cis* form by 4.4 kcal/mol at the B3LYP/6-31G(d) level of theory.

[17] Hatano M., Nishikawa K., Ishihara K. (2017). Enantioselective Cycloaddition of Styrenes with Aldimines
Catalyzed by a Chiral Magnesium Potassium Binaphthyl disulfonate Cluster
as a Chiral Brønsted Acid Catalyst. J.
Am. Chem. Soc..

[18] Grubbs R. H., Chang S. (1998). Recent advances in olefin metathesis and its application in organic
synthesis. Tetrahedron.

[19] Kam T.-S., Sim K.-M. (1998). Alkaloids from Kopsia griffithii. Phytochemistry.

