# Supplementary material for: Chiral Phosphoric Acid-Catalyzed Enantioselective Pictet–Spengler Reaction for Concise Synthesis of CF3‑Substituted Tetrahydro-β-Carbolines
Source: Org Lett. 2025 Jun 3;27(23):6204–10. doi: 10.1021/acs.orglett.5c01864 (PMC12172053; doi:10.1021/acs.orglett.5c01864)
Supplement: Supplementary file 1 [file ol5c01864_si_001.pdf]

Supporting Information

Chiral Phosphoric Acid-Catalyzed Enantioselective Pictet–Spengler Reaction for Concise Synthesis of CF_3-substituted Tetrahydro-β-Carbolines

Shigenobu Umemiya,[1] Shinnosuke Nara,[2] Masahiro Terada[2]*

[1]Research and Analytical Center for Giant Molecules, Graduate School of Science, Tohoku University, 6-3 Aramaki Aza Aoba, Aoba-ku, Sendai 980-8578, Japan
[2]Department of Chemistry, Graduate School of Science, Tohoku University, Aoba-ku, Sendai 980-8578, Japan.

Contents

1. General Information S2
2. Preparation of substrates S3
3. Enantioselective Pictet-Spengler reaction S11
4. Reaction from intermediate imine S21
5. Derivatization S22
6. Reference S30
7. X-ray Structure Reports S30
8. Optimization of Pictet Spengler reaction S35
9. NMR spectra S37
10. HPLC charts S98

1. General Information

Unless otherwise noted, all reactions were carried out under an atmosphere of standard grade nitrogen or argon gas in flame-dried glassware equipped with a magnetic stir bar. Dichloromethane (DCM), toluene, 1,2-dichloroethane (DCE), acetonitrile (MeCN), chloroform (CH_3Cl) and tetrahydrofuran (THF) were supplied from KANTO Chemical Co., Inc. as "Dehydrated solvent". Other solvents and reagents were purchased from commercial suppliers and used without further purification. Flash column chromatography was performed on silica gel 60N (spherical, neutral, 40-50 μm; Kanto Chemical Co., Inc.). Analytical thin layer chromatography (TLC) was performed on Merck precoated TLC plates (silica gel 60 GF254, 0.25 mm). ^{1}H NMR spectra were recorded on a JEOL JNM-ECA600 (600 MHz) spectrometer. Chemical shifts are reported in ppm from the solvent resonance or tetramethylsilane (TMS) as the internal standard ($CDCl_3$: 7.26 ppm, TMS: 0.00 ppm, CD_3OD: 3.31 ppm). Data are reported as follows: chemical shift, multiplicity (s = singlet, d = doublet, t = triplet, br = broad, m = multiplet), coupling constants (Hz) and integration. ^{13}C NMR spectra were recorded on a JEOL JNM-ECA600 (151 MHz) spectrometer with complete proton decoupling. Chemical shifts are reported in ppm from the solvent resonance as the internal standard ($CDCl_3$ 77.0 ppm). ^{19}F NMR spectra were recorded on JEOL JNM-ECA-600 (565 MHz) spectrometer. Optical rotations were measured on a Jasco P-1020 digital polarimeter with a sodium lamp and reported as follows; $[\alpha]^{T\,^{\circ}C}$D (c = g/100 mL, solvent, % ee). HPLC analysis was performed on a Jasco LC-2000 Plus system with UV and CD detectors. Chiral stationary phase HPLC analysis was performed on a Jasco LC-2000 Plus Series system with DAICEL chiral analytical column (4.6 mmΦ* 250 mm length) (AD-3, OD-3, ID-3). High resolution mass spectra analysis was performed on a Bruker Daltonics solariX 9.4T FT-ICR-MS spectrometer at the Research and Analytical Center for Giant Molecules, Graduate School of Science, Tohoku University. X-ray crystallographic analysis was conducted on a Rigaku XtaLAB Synergy at the Research and Analytical Center for Giant Molecules, Graduate School of Science, Tohoku University.

2. Preparation of substrates

2-1: Preparation of tryptamine (**1f–1j**, **1l**, **1n** and **1o**)

The tryptamines (**1f-1h**, **1l**, **1n**, and **1o)** were synthesized from the corresponding indoles (**S1f-S1h**, **S1l, S1n**, **S1o**). While the tryptamines (**1i** and **1j**) were synthesized from the corresponding indole-3-carboxaldehyde (**S2i** and **S2j**). Others (**1a-1e**, **1k** and **1m**) were commercially available in the form of tryptamine or its hydrochloride salt.

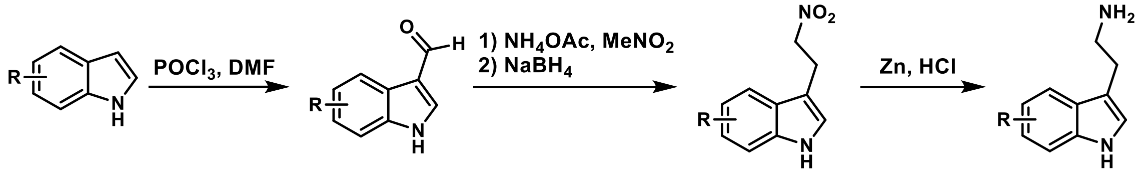

S1 **S2** **S3** **1**

General procedure A

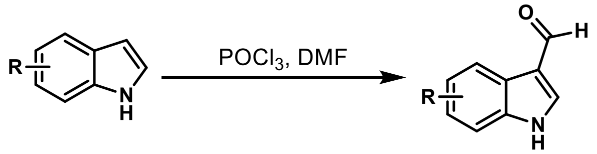

S1
commercially available

S2

Vilsmeier–Haack reaction: To a dried flask with DMF (4.0 mL, 3.0 M for $POCl_3$) was added $POCl_3$ (12 mmol, 1.12 mL, 2.0 eq.) dropwise at 0 °C and stirred for 1.5 h. The resulting mixture was added to a solution of corresponding indole **S1** (6.0 mmol, 1.0 eq.) in DMF (3.3 mL, 1.8 M for **S1**) dropwise at 0 °C and allowed to stir at room temperature for another 40 min. Then 3.8 M KOH (15.8 mL, 0.38 M) was added to the reaction mixture and the reaction mixture was refluxed using an oil bath for 1 h. The resulting mixture was cooled to 0 °C and quenched with sat. NH_4Cl aq. The aqueous layer was extracted with EtOAc (3 x 30 mL). The combined organic layers were dried over Na_2SO_4, filtered and concentrated under reduced pressure to give crude product of **S2**. The crude product was used with the next reaction without further purification.

General procedure B

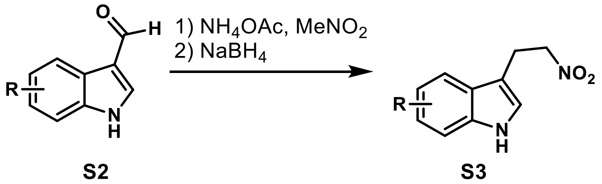

S2
commercially available
or crude compound

S3

Henry reaction: To a flask with NH_4OAc (3 mmol, 305 mg, 0.5 eq.) and corresponding aldehyde **S2** (1.0 eq.), $MeNO_2$ (13.6 mL, 0.44 M) was added, and the reaction mixture was stirred at 75 °C in an

oil bath. After confirming consumption of **S2** by TLC, the resulting mixture was cooled to 0 °C and quenched with sat. NH_4Cl aq. The aqueous layer was extracted with EtOAc (3 x 30 mL). The combined organic layers were dried over Na_2SO_4, filtered and concentrated under reduced pressure to give crude product of **S2**. The crude product was used with the next reaction without further purification.

Reduction of olefin: To a flask with corresponding nitroolefin (1.0 eq.) and dried silica gel (6 g, 1 g/mmol) in $CHCl_3$ (46 mL, 0.13 M) and isopropanol (14 mL, 0.43 M), $NaBH_4$ (15 mmol, 567 mg, 2.5 eq.) was added in four portions over 15 mins at 0 °C. The reaction mixture was turned to room temperature and stirred for a further 5 h. The excess $NaBH_4$ was then destroyed with 2 M HCl aq. Then the reaction mixture was filtered through a plug of Celite and washed with DCM. The aqueous phase was extracted with DCM (3 x 20 mL) and the combined organic layers were washed with brine (20 mL), dried over Na_2SO_4, filtered, and concentrated under reduced pressure. The crude material was purified by flash column chromatography on silica gel to give **S3**.

5-bromo-3-(2-nitroethyl)-1H-indole (**S3f**)

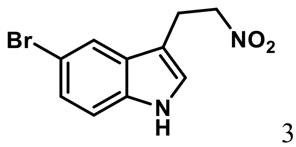

3 steps 54% yield (872 mg).

Prepared according to the **general procedure B** and purified by column chromatography (hexane/EtOAc, 9:2). Yellow solid: All spectroscopic data for **S3f** was known.[1]

3-(2-nitroethyl)-5-(trifluoromethyl)-1H-indole (**S3g**)

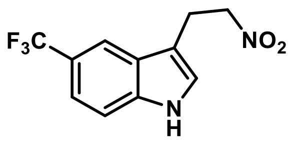

3 steps 56% yield (868 mg).

Prepared according to the **general procedure B** and purified by column chromatography (hexane/EtOAc, 9:2). R_f = 0.42 (Hexane/EtOAc= 2/1); White solid (m.p. 123.7-124.4 °C); ^{1}H NMR (600 MHz, $CDCl_3$) δ 8.29 (brs, 1H), 7.86 (s, 1H), 7.47-7.44 (m, 2H), 7.17 (d, J = 2.4 Hz, 1H), 4.69 (t, J = 7.2 Hz, 2H), 3.51 (t, J = 7.2 Hz, 2H); ^{13}C NMR (151 MHz, $CDCl_3$) δ 137.5, 126.1, 125.2 (q, J = 271.8 Hz), 124.3, 122.4 (q, J = 31.9 Hz), 119.4 (q, J = 3.0 Hz), 115.9 (q, J = 4.4 Hz), 111.8, 111.1, 75.6, 23.2; ^{19}F NMR (565 MHz, $CDCl_3$) δ -60.2; HRMS (ESI) m/z: $[M+Na]^+$ Calcd for $C_{11}H_9F_3N_2NaO2^+$, 281.0508, found 281.0508.

4-methoxy-3-(2-nitroethyl)-1H-indole (**S3h**)

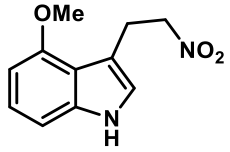
3 steps 43% yield (568 mg).

Prepared according to the **general procedure B** and purified by column chromatography (hexane/EtOAc, 9:2). Yellow solid: All spectroscopic data for **S3h** was known.[2]

4-bromo-3-(2-nitroethyl)-1H-indole (**S3i**)

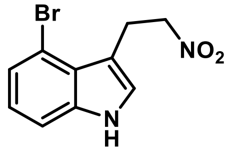
2 steps 27% yield (436 mg).

Prepared according to the **general procedure B** and purified by column chromatography (hexane/EtOAc, 9:2). Yellow solid: All spectroscopic data for **S3i** was known.[1]

6-methoxy-3-(2-nitroethyl)-1H-indole (**S3j**)

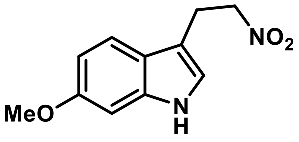
2 steps, 40% yield (529 mg).

Prepared according to the **general procedure B** and purified by column chromatography (hexane/EtOAc, 9:2). R_f = 0.42 (Hexane/EtOAc= 2/1); pale Yellow solid (m.p. 105.3-106.0 °C); ^{1}H NMR (600 MHz, $CDCl_3$) δ 7.94 (brs, 1H), 7.43 (d, *J* = 8.4 Hz, 1H), 6.95 (d, *J* = 1.2 Hz, 1H), 6.87 (d, *J* = 1.8 Hz, 1H), 6.83 (dd, *J* = 8.4 Hz, 1.8 Hz, 1H), 4.66 (t, *J* = 7.2 Hz, 2H), 3.85 (s, 3H), 3.46 (t, *J* = 7.2 Hz, 2H); ^{13}C NMR (151 MHz, $CDCl_3$) δ 156.7, 137.0, 121.2, 121.0, 118.8, 110.1, 110.0, 94.8, 75.8, 55.7, 23.7; HRMS (ESI) m/z: $[M+Na]^+$ Calcd for $C_{11}H_{12}N_2NaO_3^+$, 243.0740, found 243.0741.

3-(2-nitroethyl)-6-(trifluoromethyl)-1H-indole (**S3l**)

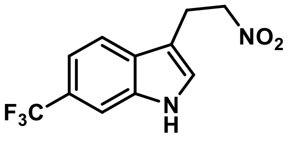
3 steps 54% yield (837 mg).

Prepared according to the **general procedure B** and purified by column chromatography (hexane/EtOAc, 9:2). R_f = 0.42 (Hexane/EtOAc= 2/1); Yellow solid (m.p. 70.3-71.0 °C); ^{1}H NMR (600 MHz, $CDCl_3$) δ 8.32 (brs, 1H), 7.66 (s, 1H), 7.65 (d, *J* = 8.4 Hz, 1H), 7.13 (dd, *J* = 8.4 Hz, 1.8

Hz, 1H), 7.07 (d, J = 1.8 Hz, 1H), 4.67 (t, J = 7.2 Hz, 2H), 3.47 (td, J = 7.2 Hz, 0.6 Hz, 2H); ^{13}C NMR (151 MHz, $CDCl_3$) δ 135.1, 128.9, 125.3, 125.0 (q, J = 271.8 Hz), 124.7 (q, J = 31.9 Hz), 118.6, 116.6 (q, J = 2.9 Hz), 110.5, 119.0 (q, J = 4.4 Hz), 75.6, 23.3; ^{19}F NMR (565 MHz, $CDCl_3$) δ -60.6; HRMS (ESI) m/z: $[M+Na]^+$ Calcd for $C_{11}H_9F_3N_2NaO2^+$, 281.0508, found 281.0508.

6-chloro-3-(2-nitroethyl)-1H-indole (**S3n**)

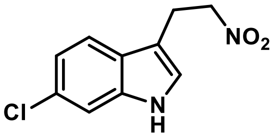

3 steps 57% yield (768 mg).

Prepared according to the **general procedure B** and purified by column chromatography (hexane/EtOAc, 9:2). R_f = 0.42 (Hexane/EtOAc= 2/1); Yellow solid (m.p. 85.3-86.1 °C); ^{1}H NMR (600 MHz, $CDCl_3$) δ 8.07 (brs, 1H), 7.47 (d, J = 8.4 Hz, 1H), 7.38 (s, 1H), 7.13 (dd, J = 8.4 Hz, 1.8 Hz, 1H), 7.07 (d, J = 1.8 Hz, 1H), 4.66 (t, J = 7.2 Hz, 2H), 3.47 (t, J = 7.2 Hz, 2H); ^{13}C NMR (151 MHz, $CDCl_3$) δ 136.5, 128.6, 125.3, 123.2, 120.7, 119.0, 111.4, 110.3, 76.6, 23.4; HRMS (ESI) m/z: $[M+Na]^+$ Calcd for $C_{10}H_9ClN_2NaO_2^+$, 247.0245, 249.0216, found 247.0245, 249.0216.

6-bromo-3-(2-nitroethyl)-1H-indole (**S3o**)

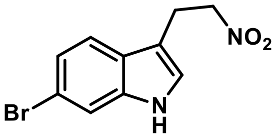

3 steps 61% yield (978 mg).

Prepared according to the **general procedure B** and purified by column chromatography (hexane/EtOAc, 9:2). Yellow solid: All spectroscopic data for **S3o** was known.[1]

General procedure C

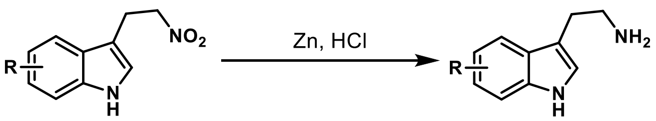

S3 **1**

<u>**Clemmensen Reduction**</u>: To a flask with corresponding nitroalkane **S3** (1.0 mmol, 1.0 eq.) in methanol (20 mL, 0.05 M) and 2.0 M hydrochloric acid (20 mL, 0.05 M), zinc (981 mg, 15 mmol, 15 eq.) was added in four portions over 15 mins and the reaction mixture was heated to reflux using an oil bath over 5 h. The reaction mixture was turned to room temperature and filtered to remove excess zinc. Then, the reaction mixture was quenched with aq. 2.0 M NaOH and filtered through a Buchner

funnel. After evaporating the methanol under reduced pressure, the aqueous phase was extracted with EtOAc (3 x 20 mL) and the combined organic layers were washed with brine (20 mL), dried over Na_2SO_4, filtered, and concentrated under reduced pressure. The crude material was purified by flash column chromatography on silica gel (DCM/MeOH = 20/3 to 20/5). The compound was washed with aq. 2.0 M NaOH (2 x 15 mL) and the organic layers were dried over Na_2SO_4, filtered, and concentrated to give **1.**

2-(5-bromo-1H-indol-3-yl) ethan-1-amine (**1f**)

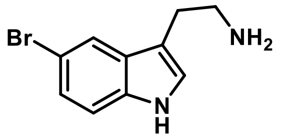

160.2 mg, 0.67 mmol, 67% yield: white solid.

R_f = 0.12 (DCM/MeOH= 20/3); ^{1}H NMR (600 MHz, CD_3OD) δ 7.57 (s, 1H), 7.14 (d, *J* = 8.4 Hz, 1H), 7.05 (dd, *J* = 8.4 Hz, 1.8 Hz, 1H), 6.96 (s, 1H), 2.75 (t, *J* = 6.0 Hz, 2H), 2.69 (t, *J* = 6.0 Hz, 2H); ^{13}C NMR (151 MHz, CD_3OD) δ 136.8, 130.5, 125.2, 125.0, 121.8, 113.9, 113.2, 112.8, 43.0, 29.2; HRMS (ESI)m/z: $[M+H]^+$ Calcd for $C_{10}H_{12}BrN_2^+$, 239.0178, 241.0158, found 239.0178, 241.0158; All spectroscopic data for **1f** was known.[1]

2-(5-(trifluoromethyl)-1H-indol-3-yl) ethan-1-amine (**1g**)

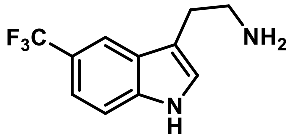

223.5 mg, 0.98 mmol, 98% yield: light yellow oil.

R_f = 0.11 (DCM/MeOH= 20/3); ^{1}H NMR (600 MHz, CD_3OD) δ 7.86 (d, *J* = 1.2 Hz, 1H), 7.45 (d, *J* = 8.4 Hz, 1H), 7.32 (dd, *J* = 8.4 Hz, 1.2 Hz, 1H), 7.20 (s, 1H), 2.91 (s, 4H); ^{13}C NMR (151 MHz, CD_3OD) δ 139.6, 128.1, 127.2 (q, *J* = 270.4 Hz), 125.8 (q, *J* = 15.1 Hz), 121.6 (q, *J* = 31.7 Hz), 118.9, 117.0 (d, *J* = 4.4 Hz), 114.6, 112.8, 43.1, 29.0; ^{19}F NMR (565 MHz, CD_3OD) δ -61.3; HRMS (ESI)m/z: $[M+H]^+$ Calcd for $C_{11}H_{11}F_3N_2^+$, 229.0947, found 229.0947; All spectroscopic data for **1g** was known.[3]

2-(4-methoxy-1H-indol-3-yl) ethan-1-amine (**1h**)

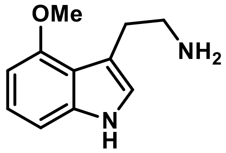

19.2 mg, 0.1 mmol, 10% yield: light yellow oil.

R_f = 0.11 (DCM/MeOH= 20/3); ^{1}H NMR (600 MHz, CD_3OD) δ 6.97 (t, *J* = 7.8 Hz, 1H), 6.91 (d, *J* = 7.8 Hz, 1H), 6.88 (s, 1H), 6.42 (d, *J* = 7.8 Hz, 1H), 3.87 (t, *J* = 1.2 Hz, 3H), 2.97 (t, *J* = 6.6 Hz, 2H), 2.89 (t, *J* = 6.6 Hz, 2H); ^{13}C NMR (151 MHz, CD_3OD) δ 155.9, 140.1, 123.2, 122.5 (d, *J* = 4.4 Hz), 118.4, 113.7, 105.8, 99.7, 55.4 (t, *J* = 4.4 Hz), 44.0, 31.3; HRMS (ESI)m/z: $[M+H]^+$ Calcd for $C_{11}H_{14}N_2O^+$, 191.1179, found 191.1179; All spectroscopic data for **1h** was known.[3]

2-(4-bromo-1H-indol-3-yl) ethan-1-amine (**1i**)

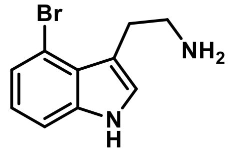

203.3 mg, 0.85 mmol, 85% yield: yellow powder.

R_f = 0.11 (DCM/MeOH= 20/3); ^{1}H NMR (600 MHz, CD_3OD) δ 7.22 (d, *J* = 8.4 Hz, 1H), 7.05 (d, *J* = 8.4 Hz, 1H), 7.04 (s, 1H), 6.83 (t, *J* = 8.4 Hz, 1H), 3.02 (t, *J* = 6.6 Hz, 2H), 2.87 (t, *J* = 6.6 Hz, 2H); ^{13}C NMR (151 MHz, CD_3OD) δ 139.7, 126.4, 126.0, 124.1, 123.2, 114.6, 114.0, 111.9, 44.4, 30.2; HRMS (ESI)m/z: $[M+H]^+$ Calcd for $C_{10}H_{12}BrN_2^+$, 239.0178, 241.0158, found 239.0178, 241.0158; All spectroscopic data for **1i** was known.[1]

2-(6-methoxy-1H-indol-3-yl) ethan-1-amine (**1j**)

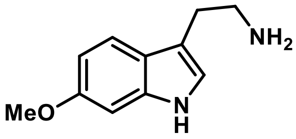

114.1 mg, 0.60 mmol, 60% yield: light brown solid.

R_f = 0.11 (DCM/MeOH= 20/3); ^{1}H NMR (600 MHz, CD_3OD) δ 7.29 (d, *J* = 8.4 Hz, 1H), 6.84 (s, 1H), 6.77 (d, *J* = 1.8 Hz, 1H), 6.57 (dd, *J* = 8.4 Hz, 1.8 Hz, 1H), 3.69 (s, 3H), 2.82 (t, *J* = 6.6 Hz, 2H), 2.76 (t, *J* = 6.6 Hz, 2H); ^{13}C NMR (151 MHz, CD_3OD) δ 157.6, 139.0, 123.2, 122.3, 119.8, 113.1, 109.9, 95.5, 55.9, 42.9, 29.2; HRMS (ESI)m/z: $[M+H]^+$ Calcd for $C_{11}H_{15}N_2O^+$, 191.1179, found 191.1179; All spectroscopic data for **1j** was known.[4]

2-(6-(trifluoromethyl)-1H-indol-3-yl) ethan-1-amine (**1l**)

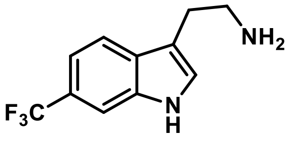

123.2 mg, 0.54 mmol, 54% yield: light yellow oil.

R_f = 0.11 (DCM/MeOH= 20/3); ^{1}H NMR (600 MHz, CD_3OD) δ 7.66 (d, *J* = 8.4 Hz, 1H), 7.62 (s,

1H), 7.25 (s, 1H), 7.21 (d, J = 8.4 Hz, 1H), 2.91 (s, 4H); ^{13}C NMR (151 MHz, CD_3OD) δ 137.0, 131.1, 127.0 (q, J = 270.4 Hz), 126.7 (q, J = 15.0 Hz), 124.3 (q, J = 31.7 Hz), 117.0 (d, J = 5.7 Hz), 116.0 (d, J = 13.0 Hz), 113.9, 119.8, 43.0, 29.0; ^{19}F NMR (565 MHz, CD_3OD) δ -61.7; HRMS (ESI)m/z: $[M+H]^+$ Calcd for $C_{11}H_{12}F_3N_2^+$, 229.0947, found 229.0947; All spectroscopic data for **1l** was known.[3]

2-(6-chloro-1H-indol-3-yl) ethan-1-amine (**1n**)

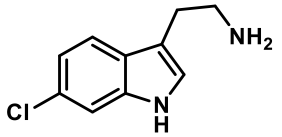

145.6 mg, 0.75 mmol, 75% yield: light brown solid.

R_f = 0.11 (DCM/MeOH= 20/3); ^{1}H NMR (600 MHz, CD_3OD) δ 7.35 (d, J = 8.4 Hz, 1H), 7.23 (d, J = 1.8 Hz, 1H), 6.95 (s, 1H), 6.86 (dd, J = 8.4 Hz, 1.8 Hz, 1H), 2.77 (t, J = 6.0 Hz, 2H), 2.73 (t, J = 6.0 Hz, 2H); ^{13}C NMR (151 MHz, CD_3OD) δ 137.2, 126.9, 126.1, 123.2, 119.0, 118.8, 112.5, 110.7, 41.7, 28.0; HRMS (ESI)m/z: $[M+H]^+$ Calcd for $C_{10}H_{12}ClN_2^+$, 195.0684, 197.0654, found 195.0684, 197.0654; All spectroscopic data for **1n** was known.[5]

2-(6-bromo-1H-indol-3-yl) ethan-1-amine (**1o**)

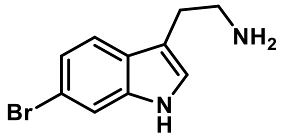

117.2 mg, 0.48 mmol, 48% yield: light brown solid.

R_f = 0.11 (DCM/MeOH= 20/3); ^{1}H NMR (600 MHz, CD_3OD) δ 7.39 (d, J = 1.8 Hz, 1H), 7.32 (d, J = 8.4 Hz, 1H), 6.99 (dd, J = 8.4 Hz, 1.8 Hz, 1H), 6.95 (s, 1H), 2.78 (t, J = 6.0 Hz, 2H), 2.74 (t, J = 6.0 Hz, 2H); ^{13}C NMR (151 MHz, CD_3OD) δ 139.0, 127.7, 124.5, 122.7, 120,8, 115.8, 115.1, 113.8, 43.0, 29.3; HRMS (ESI)m/z: $[M+H]^+$ Calcd for $C_{10}H_{12}BrN_2^+$, 239.0178, 241.0158, found 239.0178, 241.0158; All spectroscopic data for **1o** was known.[1]

2-2: Preparation of ketone (**2a**, **2b** and **2c**)

The ketones (**2a**, **2b** and **2c)** were synthesized from the corresponding acetylenes (**S4a**, **S4b** and **S4c**) and ethyl 2,2,2-trifluoroacetate.

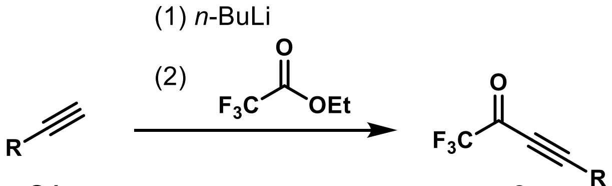

S4 → 2

General procedure D

To a solution of alkyne **S4** (5 mmol, 1.0 eq.) in THF (1.67 mL, 0.3 M), *n*-BuLi (1.6 M in Hexene, 3.13 mL, 0.2 mmol, 1.0 eq.) was added dropwise at -78 °C. After the reaction mixture was stirred at -78 °C for 30 min, 2,2,2-trifluoroacetate (592 mL, 5.0 mmol, 1.0 eq.) was added to the reaction vessel and the resulting mixture was stirred at 0 °C to room temperature for 12 h. The reaction mixture was quenched with sat. NH_4Cl aq. at 0 °C. The resulting mixture was extracted with EtOAc (3 x 10 mL). Combined organic layers were dried over Na_2SO_4, filtrated, and concentrated under reduced pressure. The residue was purified by flash column chromatography on silica gel (Hexane/EtOAc = 30/1) to give **2**.

1,1,1-trifluoro-4-phenylbut-3-yn-2-one (**2a**)

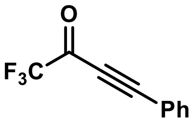

355 mg, 1.79 mmol, 78% yield. colorless oil.

All spectroscopic data for **2a** was known.[6]

4-(*tert*-butyldimethylsilyl)-1,1,1-trifluorobut-3-yn-2-one (**2b**)

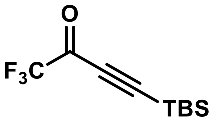

791 mg, 3.35 mmol, 67% yield. colorless oil.

R_f = 0.48 (Hexane/EtOAc= 10/1); ^{1}H NMR (600 MHz, $CDCl_3$) δ 0.99 (s, 9H), 0.24 (s, 6H); ^{13}C NMR (151 MHz, $CDCl_3$) δ 166.7 (q, *J* = 41.8 Hz), 114.6 (q, *J* = 289.2Hz), 109.7, 97.0, 25.8 (6C), 16.7, -5.6 (3C); ^{19}F NMR (565 MHz, $CDCl_3$) δ -78.0; HRMS (ESI)m/z: $[M + Na + MeOH]^+$ calculated for $C_{11}H_{19}F_3NaO_2Si^+$, 291.0999, found 291.0999.

1,1,1-trifluoro-4-(triisopropylsilyl) but-3-yn-2-one (**2c**)

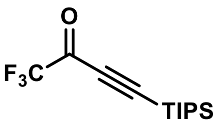

1.32 g, 4.60 mmol, 92% yield. colorless oil

R_f = 0.36 (Hexane/EtOAc= 20/1); ^{1}H NMR (600 MHz, $CDCl_3$) δ 1.22-1.09 (m, 21H); ^{13}C NMR (151

MHz, $CDCl_3$) δ 166.7 (q, J = 42.0 Hz), 114.8 (q, J = 289.2Hz), 109.0, 98.5, 18.4 (6C), 10.9 (3C); ^{19}F NMR (565 MHz, $CDCl_3$) δ -78.2; HRMS (ESI)m/z: $[M+H]^+$ Calcd for $C_{13}H_{22}F_3OSi^+$, 279.1387, found 279.1387; All spectroscopic data for **2c** was known.[6]

3. Enantioselective Pictet-Spengler reaction (Figure 2)

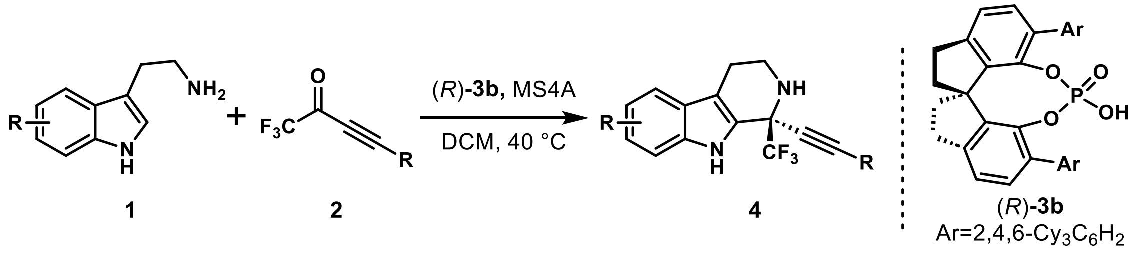

General procedure E

To a solution of tryptamine **1** (0.1 mmol, 1.0 eq.), catalyst (*R*)-**3b** and MS4A (100 wt% for **1**) in DCM (500 μL, 0.2 M), ketone **2** (0.12 mmol, 1.2 eq.) was added at room temperature and the reaction mixture was heated to 40 °C using a heating block. After confirming completion of the reaction by monitoring the TLC, the reaction mixture was quenched with sat. $NaHCO_3$ aq. at room temperature. The resulting mixture was extracted with EtOAc (3 x 5 mL). Combined organic layers were dried over Na_2SO_4, filtrated, and concentrated under reduced pressure. The residue was purified by flash column chromatography on silica gel (Hexane/DCM = 8/1 to 9/2) to give **4**.

Note: In the general procedure E, the heating block was exclusively used as the heat source.

(*R*)-1-(trifluoromethyl)-1-((triisopropylsilyl)ethynyl)-2,3,4,9-tetrahydro-1H-pyrido[3,4-b] indole (**4ac**)

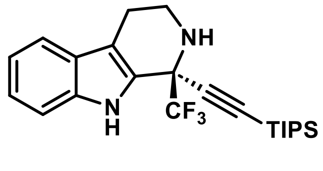

4ac was prepared according to **General procedure E**, starting from **1a** (0.1 mmol, 16.0 mg, 1.0 eq.) and ketone **2c** (0.12 mmol, 33.4 mg, 1.2 eq.) with catalyst (*R*)-**3b** (0.005 mmol, 4.4 mg, 0.05 eq.) and MS4A (16.0 mg, 100 wt% for **1a**). The reaction mixture was stirred at 40 °C for 45 h. **4ac** was afforded 38.7 mg, 0.092 mmol, 92% yield.

$[\alpha]_D^{23}$ =+48.8 (c = 0.35, $CHCl_3$, 91% ee); R_f = 0.62 (Hexane/EtOAc= 5/1); White solid (m.p. 45.3-46.0 °C); ^{1}H NMR (600 MHz, $CDCl_3$) δ 8.04 (brs, 1H), 7.55 (d, J = 7.8 Hz, 1H), 7.39 (d, J = 7.8 Hz, 1H), 7.24 (td, J = 7.8 Hz, 0.6Hz, 1H), 7.14 (td, J = 7.8 Hz, 0.6Hz, 1H), 3.37-3.29 (m, 2H), 2.90-2.77 (m, 2H), 2.28 (brs, 1H)1.07-1.02 (m, 21H); ^{13}C NMR (151 MHz, $CDCl_3$) δ 136.2, 126.4, 125.8, 124.4 (q, J = 284.8 Hz), 123.1, 119.8, 118.9, 112.5, 111.3, 102.1, 88.1, 57.1 (q, J = 30.4Hz), 40.4, 21.5, 18.5

(6C), 11.0 (3C); ^{19}F NMR (565 MHz, $CDCl_3$) δ -77.5; HRMS (ESI) m/z: $[M+H]^+$ Calcd for $C_{23}H_{32}F_3N_2Si^+$ 421.2281, found 421.2281; HPLC analysis CHIRALCEL AD-3 (Hexane:*i*PrOH = 90/10, 1.0 mL/min, 30 °C, 254 nm) 12.6 min (major), 17.4 min (minor). (Ee% was measured after deprotection of silyl groups with TBAF.); **Configuration Assignment**: The absolute configuration of this compound was determined by X-ray crystallographic analysis after its derivatization to compound **7** by deprotection of TIPS group.

(*R*)-6-methoxy-1-(trifluoromethyl)-1-((triisopropylsilyl)ethynyl)-2,3,4,9-tetrahydro-1H-pyrido[3,4-b] indole (**4b**)

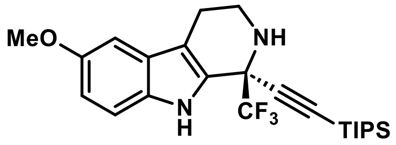

4b was prepared according to **General procedure E**, starting from **1b** (0.1 mmol, 19.0 mg, 1.0 eq.) and ketone **2c** (0.12 mmol, 33.4 mg, 1.2 eq.) with catalyst (*R*)-**3b** (0.005 mmol, 4.4 mg, 0.05 eq.) and MS4A (19.0 mg, 100 wt% for **1b**). The reaction mixture was stirred at 40 °C for 45 h. **4b** was afforded 34.3 mg, 0.076 mmol, 76% yield.

$[\alpha]_D^{23}$ =+11.2 (*c* = 0.10, $CHCl_3$, 91% ee); R_f = 0.43 (Hexane/EtOAc= 5/1); pale yellow foam; ^{1}H NMR (600 MHz, $CDCl_3$) δ 7.95 (brs, 1H), 7.28 (d, *J* = 9.0 Hz, 1H), 6.98 (d, *J* = 2.4 Hz, 1H), 6.90 (dd, *J* = 9.0 Hz, 2.4 Hz, 1H), 3.86 (s, 3H), 3.37-3.28 (m, 2H), 2.88-2.83 (m, 1H), 2.77-2.73 (m, 1H), 2.28 (brs, 1H), 1.08-1.01 (m, 21H); ^{13}C NMR (151 MHz, $CDCl_3$) δ 154.2, 131.3, 126.7, 126.5, 124.4 (q, *J* = 284.9 Hz), 113.3, 112.1, 112.1, 102.2, 100.6, 88.1, 57.1 (q, *J* = 30.4Hz), 56.9, 40.4, 21.6 , 18.5 (d, *J* = 4.4 Hz, 6C), 11,0 (3C); ^{19}F NMR (565 MHz, $CDCl_3$) δ -77.5; HRMS (ESI) m/z: $[M+H]^+$ Calcd for $C_{24}H_{34}F_3N_2OSi^+$, 451.2387, found 451.2387; HPLC analysis CHIRALCEL ID-3 (Hexane:*i*PrOH = 90/10, 1.0mL/min, 30 °C, 254 nm) 7.7 min (major), 8.8 min (minor). (Ee% was measured after deprotection of silyl groups with TBAF.); **Configuration Assignment**: The absolute configuration was assigned as (*R*) by an analogy of absolute configuration of **7**.

(*R*)-6-methyl-1-(trifluoromethyl)-1-((triisopropylsilyl)ethynyl)-2,3,4,9-tetrahydro-1H-pyrido[3,4-b] indole (**4c**)

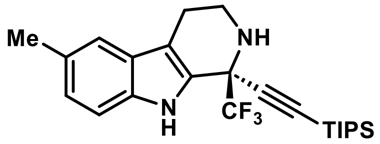

4c was prepared according to **General procedure E**, starting from **1c** (0.1 mmol, 17.4 mg, 1.0 eq.)

and ketone **2c** (0.12 mmol, 33.4 mg, 1.2 eq.) with catalyst (*R*)-**3b** (0.005 mmol, 4.4 mg, 0.05 eq.) and MS4A (17.4 mg, 100 wt% for **1c**). The reaction mixture was stirred at 40 °C for 24 h. **4c** was afforded 35.6 mg, 0.082 mmol, 82% yield.

$[\alpha]_D^{23}$ =+27.6 (*c* = 0.12, $CHCl_3$, 88% ee); R_f = 0.67 (Hexane/EtOAc= 5/1); yellow oil; ^1H NMR (600 MHz, $CDCl_3$) δ 7.94 (brs, 1H), 7.33 (s, 1H), 7.28 (d, *J* = 8.4 Hz, 1H), 7.07 (dd, *J* = 8.4 Hz, 1.2 Hz, 1H), 3.35-3.27 (m, 2H), 2.88-2.83 (m, 1H), 2.77-2.73 (m, 1H), 2.45 (s, 3H), 2.27 (brs, 1H), 1.09-1.01 (m, 21H); ^{13}C NMR (151 MHz, $CDCl_3$) δ 134.5, 129.1, 126.6, 125.9, 124.6, 124.4 (q, *J* = 284.8 Hz), 118.5, 111.9, 111.0, 102.2, 88.0, 57.1 (q, *J* = 30.5Hz), 40.4, 21.5, 21.4, 18.5 (6C), 11,0 (3C); ^{19}F NMR (565 MHz, $CDCl_3$) δ -77.5; HRMS (ESI) m/z: $[M+H]^+$ Calcd for $C_{24}H_{34}F_3N_2Si^+$, 435.2438, found 435.2438; HPLC analysis CHIRALCEL OD-3 (Hexane:*i*PrOH = 95/5, 0.8mL/min, 40 °C, 254 nm) 12.2 min (major), 17.4 min (minor). (Ee% was measured after deprotection of silyl groups with TBAF.); **Configuration Assignment**: The absolute configuration was assigned as (*R*) by an analogy of absolute configuration of **7**.

(*R*)-6-fluoro-1-(trifluoromethyl)-1-((triisopropylsilyl)ethynyl)-2,3,4,9-tetrahydro-1H-pyrido[3,4-b] indole (**4d**)

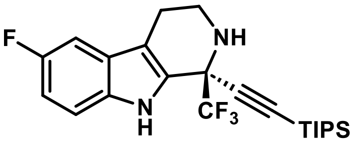

4d was prepared according to **General procedure E**, starting from **1d** (0.1 mmol, 17.8 mg, 1.0 eq.) and ketone **2c** (0.12 mmol, 33.4 mg, 1.2 eq.) with catalyst (*R*)-**3b** (0.005 mmol, 4.4 mg, 0.05 eq.) and MS4A (17.8 mg, 100 wt% for **1d**). The reaction mixture was stirred at 40 °C for 192 h. **4d** was afforded 18.0 mg, 0.041 mmol, 41% yield.

$[\alpha]_D^{23}$ =+45.9 (*c* = 0.19, $CHCl_3$, 87% ee); R_f = 0.67 (Hexane/EtOAc= 5/1); pale yellow solid (m.p. 65.3-66.0 °C); ^1H NMR (600 MHz, $CDCl_3$) δ 8.03 (brs, 1H), 7.30 (dd, *J* = 8.4 Hz, 4.2 Hz, 1H), 7.18 (dd, *J* = 8.4 Hz, 2.4 Hz, 1H), 6.99 (td, *J* = 8.4 Hz, 2.4 Hz, 1H), 3.37-3.28 (m, 2H), 2.86-2.81 (m, 1H), 2.75-2.71 (m, 1H), 2.29 (brs, 1H), 1.09-1.01 (m, 21H); ^{13}C NMR (151 MHz, $CDCl_3$) δ 157.9 (d, *J* = 235.7 Hz), 132.7, 127.6, 126.7 (d, *J* = 10.1 Hz), 124.3 (q, *J* = 284.9 Hz), 112.6 (d, *J* = 4.4 Hz), 112.0 (d, *J* = 8.6 Hz), 111.4 (d, *J* = 26.1 Hz), 104.0 (d, *J* = 23.1 Hz), 101.9, 88.4, 57.1 (q, *J* = 30.4Hz), 40.3, 21.5, 18.5 (d, *J* = 2.9 Hz, 6C), 11,0; ^{19}F NMR (565 MHz, $CDCl_3$) δ -77.5 (s, 3F), -124.0 (m, 1F); HRMS (ESI) m/z: $[M+H]^+$ Calcd for $C_{23}H_{31}F_4N_2Si^+$, 439.2187, found 439.2187; HPLC analysis CHIRALCEL AD-3 (Hexane:*i*PrOH = 90/10, 1.0mL/min, 40 °C, 254 nm) 9.7 min (major), 13.2 min (minor). (Ee% was measured after deprotection of silyl groups with TBAF.); **Configuration Assignment**: The absolute configuration was assigned as (*R*) by an analogy of absolute configuration

of **7**.

(*R*)-6-chloro-1-(trifluoromethyl)-1-((triisopropylsilyl)ethynyl)-2,3,4,9-tetrahydro-1H-pyrido[3,4-b] indole (**4e**)

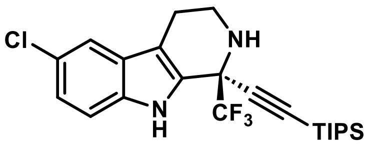

4e was prepared according to **General procedure E**, starting from **1e** (0.1 mmol, 22.8 mg, 1.0 eq.) and ketone **2c** (0.12 mmol, 33.4 mg, 1.2 eq.) with catalyst (*R*)-**3b** (0.005 mmol, 4.4 mg, 0.05 eq.) and MS4A (22.8 mg, 100 wt% for **1e**). The reaction mixture was stirred at 40 °C for 120 h. **4e** was afforded 31.9 mg, 0.070 mmol, 70% yield.

$[\alpha]_D^{23}$ =+1.4 (*c* = 0.53, $CHCl_3$, 88% ee); R_f = 0.67 (Hexane/EtOAc= 5/1); pale yellow solid (m.p. 65.3-66.0 °C); ^{1}H NMR (600 MHz, $CDCl_3$) δ 8.07 (brs, 1H), 7.51 (d, *J* = 2.4 Hz, 1H), 7.30 (d, *J* = 9.0 Hz, 1H), 7.19 (dd, *J* = 9.0 Hz, 2.4 Hz, 1H), 3.37-3.27 (m, 2H), 2.86-2.72 (m, 2H), 2.15 (brs, 1H), 1.07-1.02 (m, 21H); ^{13}C NMR (151 MHz, $CDCl_3$) δ 134.5, 127.5, 127.3, 125.6, 124.3 (q, *J* = 284.8 Hz), 123.4, 118.5, 112.3, 112.2, 101.8, 88.6, 57.0 (q, *J* = 30.4Hz), 40.2, 21.4, 18.5 (6C), 10.9 (3C); ^{19}F NMR (565 MHz, $CDCl_3$) δ -77.4; HRMS (ESI) m/z: $[M+H]^+$ Calcd for $C_{23}H_{31}ClF_3N_2Si^+$ 455.1892, 457.1863, found 455.1892, 457.1862; HPLC analysis CHIRALCEL ID-3 (Hexane:*i*PrOH = 98/2, 0.7mL/min, 40 °C, 254 nm) 11.6 min (major), 12.5 min (minor). (Ee% was measured after deprotection of silyl groups with TBAF.); **Configuration Assignment**: The absolute configuration was assigned as (*R*) by an analogy of absolute configuration of **7**.

(*R*)-6-bromo-1-(trifluoromethyl)-1-((triisopropylsilyl)ethynyl)-2,3,4,9-tetrahydro-1H-pyrido[3,4-b] indole (**4f**)

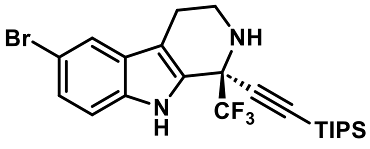

4f was prepared according to **General procedure E**, starting from **1f** (0.1 mmol, 23.9 mg, 1.0 eq.) and ketone **2c** (0.12 mmol, 33.4 mg, 1.2 eq.) with catalyst (*R*)-**3b** (0.010 mmol, 8.8 mg, 0.10 eq.) and MS4A (23.9 mg, 100 wt% for **1f**). The reaction mixture was stirred at 40 °C for 144 h. **4f** was afforded 33.9 mg, 0.068 mmol, 68% yield.

$[\alpha]_D^{23}$ =-11.4 (*c* = 0.71, $CHCl_3$, 83% ee); R_f = 0.67 (Hexane/EtOAc= 5/1); yellow solid (m.p. 57.0-57.9 °C); ^{1}H NMR (600 MHz, $CDCl_3$) δ 8.06 (brs, 1H), 7.67 (d, *J* = 2.4 Hz, 1H), 7.32 (dd, *J* = 9.0 Hz,

2.4 Hz, 1H), 7.26 (d, J = 9.0 Hz, 1H), 3.37-3.27 (m, 2H), 2.86-2.81 (m, 1H), 2.76-2.72 (m, 1H), 2.29 (brs, 1H), 1.08-1.02 (m, 21H); ^{13}C NMR (151 MHz, $CDCl_3$) δ 134.8, 128.1, 127.2, 125.9, 124.3 (q, J = 284.8 Hz), 121.6, 113.0, 112.7, 112.1, 101.7, 88.6, 57.0 (q, J = 30.4Hz), 40.2, 21.4, 18.5(6C), 10.9(3C); ^{19}F NMR (565 MHz, $CDCl_3$) δ -77.5; HRMS (ESI) m/z: $[M+H]^+$ Calcd for $C_{23}H_{31}BrF_3N_2Si^+$, 499.1387, 501.1366, found 499.1387, 501.1366; HPLC analysis CHIRALCEL OD-3 (Hexane:*i*PrOH = 90/10, 1.0mL/min, 40 °C, 254 nm) 7.0 min (major), 10.6 min (minor). (Ee% was measured after deprotection of silyl groups with TBAF.); **Configuration Assignment**: The absolute configuration was assigned as (*R*) by an analogy of absolute configuration of **7**.

(*R*)-1,6-bis(trifluoromethyl)-1-((triisopropylsilyl)ethynyl)-2,3,4,9-tetrahydro-1H-pyrido[3,4-b] indole (**4g**)

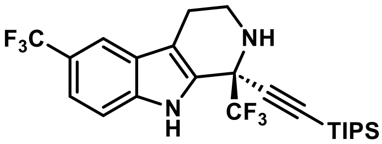

4g was prepared according to **General procedure E**, starting from **1g** (0.1 mmol, 22.8 mg, 1.0 eq.) and ketone **2c** (0.12 mmol, 33.4 mg, 1.2 eq.) with catalyst (*R*)-**3b** (0.005 mmol, 4.4 mg, 0.05 eq.) and MS4A (22.8 mg, 100 wt% for **1g**). The reaction mixture was stirred at 40 °C for 240 h. **4g** was afforded 12.9 mg, 0.026 mmol, 26% yield.

$[\alpha]_D^{23}$ =+16.7 (c = 0.24, $CHCl_3$, 81% ee); R_f = 0.67 (Hexane/EtOAc= 5/1); white solid (m.p. 42.0-42.7 °C); ^{1}H NMR (600 MHz, $CDCl_3$) δ 8.24 (brs, 1H), 7.85 (s, 1H), 7.49-7.46 (m, 2H), 3.39-3.30 (m, 2H), 2.93-2.79 (m, 2H), 2.32 (brs, 1H), 1.08-1.02 (m, 21H); ^{13}C NMR (151 MHz, $CDCl_3$) δ 137.5, 127.8, 125.8, 125.2 (q, J = 271.8 Hz), 124.3 (q, J = 284.8 Hz), 122.4 (q, J = 31.9 Hz), 119.9(d, J = 4.4 Hz), 116.8 (d, J = 4.4 Hz), 113.4, 111.5, 101.6, 88.8, 57.0 (q, J = 30.4Hz), 40.2, 21.4, 18.5 (6C), 10.9 (3C); ^{19}F NMR (565 MHz, $CDCl_3$) δ -60.3, -77.4; HRMS-ESI (m/z): $[M + H]^+$ Calcd for $C_{24}H_{30}F_6N_2Si^+$, 489.2155, found 489.2155; HPLC analysis CHIRALCEL AD-3 (Hexane:*i*PrOH = 90/10, 1.0mL/min, 30 °C, 254 nm) 8.2 min (major), 11.6 min (minor). (Ee% was measured after deprotection of silyl groups with TBAF.); **Configuration Assignment**: The absolute configuration was assigned as (*R*) by an analogy of absolute configuration of **7**.

(*R*)-5-methoxy-1-(trifluoromethyl)-1-((triisopropylsilyl)ethynyl)-2,3,4,9-tetrahydro-1H-pyrido[3,4-b] indole (**4h**)

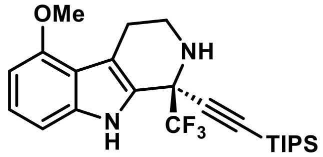

4h was prepared according to **General procedure E**, starting from **1h** (0.1 mmol, 19.0 mg, 1.0 eq.) and ketone **2c** (0.12 mmol, 33.4 mg, 1.2 eq.) with catalyst (*R*)**-3b** (0.005 mmol, 4.4 mg, 0.05 eq.) and MS4A (19.0 mg, 100 wt% for **1**). The reaction mixture was stirred at 40 °C for 43 h. **4h** was afforded 33.0 mg, 0.073 mmol, 73% yield.

$[\alpha]_D^{23}$ =-10.6 (*c* = 0.21, $CHCl_3$, 98% ee); R_f = 0.63 (Hexane/EtOAc= 5/1); yellow solid (m.p. 63.6-64.5 °C); ^{1}H NMR (600 MHz, $CDCl_3$) δ 7.99 (brs, 1H), 7.12 (t, *J* = 7.8 Hz, 1H), 6.98 (d, *J* = 7.8 Hz, 1H), 6.50 (d, *J* = 7.8 Hz, 1H), 3.90 (s, 3H), 3.32-3.25 (m, 2H), 3.09-3.01 (m, 2H), 2.24 (brs, 1H), 1.07-1.01 (m, 21H); ^{13}C NMR (151 MHz, $CDCl_3$) δ 154.9, 137.6, 124.4 (q, *J* = 284.8 Hz), 123.9, 123.8, 116.6, 112.5, 104.5, 102.4, 99.8, 87.9, 57.0(q, *J* = 30.4Hz), 55.2, 40.6, 23.5, 18.5 (6C), 11.0 (3C); ^{19}F NMR (565 MHz, $CDCl_3$) δ -77.6; HRMS (ESI) m/z: $[M+H]^+$ Calcd for $C_{24}H_{34}F_3N_2OSi^+$, 451.2387, found 451.2387; HPLC analysis CHIRALCEL AD-3(Hexane:*i*PrOH = 90/10, 1.0mL/min, 30 °C, 254 nm) 11.9min (major), 16.7min (minor). (Ee% was measured after deprotection of silyl groups with TBAF.); **Configuration Assignment**: The absolute configuration was assigned as (*R*) by an analogy of absolute configuration of **7**.

(*R*)-5-bromo-1-(trifluoromethyl)-1-((triisopropylsilyl)ethynyl)-2,3,4,9-tetrahydro-1H-pyrido[3,4-b] indole (**4i**)

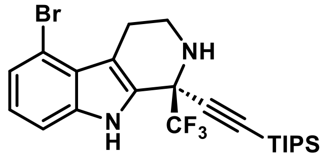

4i was prepared according to **General procedure E**, starting from **1i** (0.1 mmol, 23.9 mg, 1.0 eq.) and ketone **2c** (0.12 mmol, 33.4 mg, 1.2 eq.) with catalyst (*R*)**-3b** (0.005 mmol, 4.4 mg, 0.05 eq.) and MS4A (23.9 mg, 100 wt% for **1i**). The reaction mixture was stirred at 40 °C for 240 h. **4i** was afforded 9.0 mg, 0.018 mmol, 18% yield.

$[\alpha]_D^{23}$ =-39.3 (*c* = 0.57, $CHCl_3$, 98% ee); R_f = 0.63 (Hexane/EtOAc= 5/1); yellow oil; ^{1}H NMR (600 MHz, $CDCl_3$) δ 8.13 (brs, 1H), 7.32 (dd, *J* = 7.8 Hz, 1.2 Hz, 1H), 7.27 (dd, *J* = 7.8 Hz, 1.2 Hz, 1H), 7.05 (t, *J* = 7.8 Hz, 1H), 3.37-3.13 (m, 4H), 2.22 (brs, 1H), 1.08-1.02 (m, 21H); ^{13}C NMR (151 MHz, $CDCl_3$) δ 137.2, 126.9, 125.3, 124.3 (q, *J* = 284.8 Hz), 123.9, 123.8, 114.6, 113.3, 110.5, 101.8, 88.5, 57.1 (q, *J* = 30.4Hz), 40.3, 23.5, 18.5 (6C), 11.0(3C); ^{19}F NMR (565 MHz, $CDCl_3$) δ -77.4; HRMS

(ESI) m/z: $[M+H]^+$ Calcd for $C_{23}H_{32}BrF_3N_2Si^+$, 499.1387, 501.1366, found 499.1387, 501.1366; HPLC analysis CHIRALCEL OD-3 (Hexane:*i*PrOH = 95/5, 0.8mL/min, 40 °C, 254 nm) 11.2 min (major), 12.8 min (minor). (Ee% was measured after deprotection of silyl groups with TBAF.); **Configuration Assignment**: The absolute configuration was assigned as (*R*) by an analogy of absolute configuration of **7**.

(*R*)-7-methoxy-1-(trifluoromethyl)-1-((triisopropylsilyl)ethynyl)-2,3,4,9-tetrahydro-1H-pyrido[3,4-b] indole (**4j**)

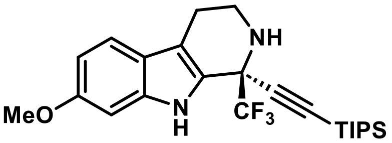

4j was prepared according to **General procedure E**, starting from **1j** (0.1 mmol, 19.0 mg, 1.0 eq.) and ketone **2c** (0.12 mmol, 33.4 mg, 1.2 eq.) with catalyst (*R*)-**3b** (0.005 mmol, 4.4 mg, 0.05 eq.) and MS4A (19.0 mg, 100 wt% for **1j**). The reaction mixture was stirred at 40 °C for 22 h. **4j** was afforded 35.2 mg, 0.078 mmol, 78% yield.

$[\alpha]_D^{23}$ = +82.2 (*c* = 0.62, $CHCl_3$, 86% ee); R_f = 0.63 (Hexane/EtOAc= 5/1); yellow foam; ^1H NMR (600 MHz, $CDCl_3$) δ 7.92 (brs, 1H), 7.41 (d, *J* = 8.4 Hz, 1H), 6.89 (d, *J* = 1.2 Hz, 1H), 6.80 (dd, *J* = 7.8 Hz, 1.2 Hz, 1H), 3.85 (s, 3H), 3.33-3.29 (m, 2H), 2.84-2.73 (m, 2H), 2.28 (brs, 1H), 1.09-1.02 (m, 21H),; ^{13}C NMR (151 MHz, $CDCl_3$) δ 157.2, 137.1, 124.5 (q, *J* = 284.8 Hz), 124.4, 120.8, 119.5, 112.4, 109.8, 102.3, 94.8, 87.9, 57.0(q, *J* = 30.4Hz), 55.7, 40.4, 21.6, 18.5 (6C), 11.0 (3C); ^{19}F NMR (565 MHz, $CDCl_3$) δ -77.6; HRMS (ESI) m/z: $[M+H]^+$ Calcd for $C_{24}H_{34}F_3N_2OSi^+$, 451.2387, found 451.2387; HPLC analysis CHIRALCEL AD-3 (Hexane:*i*PrOH = 90/10, 1.0mL/min, 30 °C, 254 nm) 13.7min (major), 22.3min (minor). (Ee% was measured after deprotection of silyl groups with TBAF.); **Configuration Assignment**: The absolute configuration was assigned as (*R*) by an analogy of absolute configuration of **7**.

(*R*)-7-methyl-1-(trifluoromethyl)-1-((triisopropylsilyl)ethynyl)-2,3,4,9-tetrahydro-1H-pyrido[3,4-b] indole (**4k**)

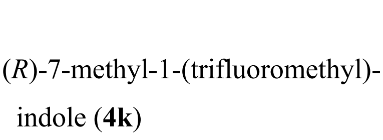

4k was prepared according to **General procedure E**, starting from **1k** (0.1 mmol, 17.4 mg, 1.0 eq.) and ketone **2c** (0.12 mmol, 33.4 mg, 1.2 eq.) with catalyst (*R*)-**3b** (0.005 mmol, 4.4 mg, 0.05 eq.) and MS4A (17.4 mg, 100 wt% for **1k**). The reaction mixture was stirred at 40 °C for 18 h. **4k** was afforded

35.7 mg, 0.082 mmol, 82% yield.

$[\alpha]_D^{23}$ =+83.6 (c = 0.08, $CHCl_3$, 87% ee); R_f = 0.67 (Hexane/EtOAc= 5/1); yellow foam; ^1H NMR (600 MHz, $CDCl_3$) δ 7.91 (brs, 1H), 7.42 (d, J = 8.4 Hz, 1H), 7.18 (d, J = 0.6 Hz, 1H), 6.97 (dd, J = 8.4 Hz, 0.6 Hz, 1H), 3.36-3.27 (m, 2H), 2.88-2.83 (m, 1H), 2.77-2.74 (m, 1H), 2.46 (s, 3H), 2.27 (brs, 1H), 1.08-1.00 (m, 21H); ^{13}C NMR (151 MHz, $CDCl_3$) δ 136.7, 133.0, 125.1, 124.4 (q, J = 284.9 Hz) 124.3, 121.5, 118.5, 112.3, 111.3, 102.2, 87.9, 57.1 (q, J = 30.4Hz), 40.4, 21.8, 21.6, 18.5 (d, J = 4.4 Hz, 6C), 11,0 (3C); ^{19}F NMR (565 MHz, $CDCl_3$) δ -77.5; HRMS (ESI) m/z: $[M+H]^+$ Calcd for $C_{24}H_{34}F_3N_2Si^+$, 435.2438, found 435.2438; HPLC analysis CHIRALCEL ID-3 (Hexane:*i*PrOH = 95/5, 1.0mL/min, 30 °C, 254 nm) 6.8 min (major), 7.8 min (minor). (Ee% was measured after deprotection of silyl groups with TBAF.); **Configuration Assignment**: The absolute configuration was assigned as (*R*) by an analogy of absolute configuration of **7**.

(*R*)-1,7-bis(trifluoromethyl)-1-((triisopropylsilyl)ethynyl)-2,3,4,9-tetrahydro-1H-pyrido[3,4-b] indole (**4l**)

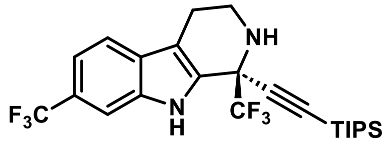

4l was prepared according to **General procedure E**, starting from **1l** (0.1 mmol, 22.8 mg, 1.0 eq.) and ketone **2c** (0.12 mmol, 33.4 mg, 1.2 eq.) with catalyst (*R*)-**3b** (0.005 mmol, 4.4 mg, 0.05 eq.) and MS4A (22.8 mg, 100 wt% for **1l**). The reaction mixture was stirred at 40 °C for 240 h. **4l** was afforded 15.5 mg, 0.032 mmol, 32% yield.

$[\alpha]_D^{23}$ =+29.4 (c = 0.54, $CHCl_3$, 64% ee); R_f = 0.67 (Hexane/EtOAc= 5/1); yellow oil; ^1H NMR (600 MHz, $CDCl_3$) δ 8.26 (brs, 1H), 7.69 (s, 1H), 7.63 (d, J = 8.4 Hz, 1H), 7.38 (d, J = 8.4 Hz, 1H), 3.40-3.30 (m, 2H), 2.92-2.79 (m, 2H), 2.32 (brs, 1H), 1.07-1.02 (m, 21H); ^{13}C NMR (151 MHz, $CDCl_3$) δ 135.1, 128.6, 125.2 (q, J = 31.9 Hz), 125.0 (q, J = 271.8 Hz), 124.3 (q, J = 284.8 Hz), 119.4, 116.6 (d, J = 4.4 Hz), 112.7, 108.9 (d, J = 4.4 Hz), 101.5, 88.8, 57.0 (q, J = 30.4Hz), 40.2, 21.4, 18.5 (6C), 10.9 (3C); ^{19}F NMR (565 MHz, $CDCl_3$) δ -60.7, -77.4; HRMS-ESI (m/z): $[M + H]^+$ Calcd for $C_{24}H_{30}F_6N_2Si^+$, 489.2155, found 489.2155; HPLC analysis CHIRALCEL AD-3 (Hexane:*i*PrOH = 90/10, 1.0mL/min, 30 °C, 254 nm) 7.9 min (major), 14.3 min (minor). (Ee% was measured after deprotection of silyl groups with TBAF.); **Configuration Assignment**: The absolute configuration was assigned as (*R*) by an analogy of absolute configuration of **7**.

(*R*)-7-fluoro-1-(trifluoromethyl)-1-((triisopropylsilyl)ethynyl)-2,3,4,9-tetrahydro-1H-pyrido[3,4-b] indole (**4m**)

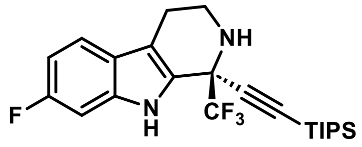

4m was prepared according to **General procedure E**, starting from **1m** (0.1 mmol, 17.8 mg, 1.0 eq.) and ketone **2c** (0.12 mmol, 33.4 mg, 1.2 eq.) with catalyst (*R*)**-3b** (0.005 mmol, 4.4 mg, 0.05 eq.) and MS4A (17.8 mg, 100 wt% for **1m**). The reaction mixture was stirred at 40 °C for 64 h. **4m** was afforded 36.6 mg, 0.083 mmol, 83% yield.

$[\alpha]_D^{23}$ =+38.7 (*c* = 0.18, $CHCl_3$, 92% ee); R_f = 0.67 (Hexane/EtOAc= 5/1); yellow oil; ^{1}H NMR (600 MHz, $CDCl_3$) δ 8.03 (brs, 1H), 7.45 (dd, *J* = 9.0 Hz, 4.8 Hz 1H), 7.18 (dd, *J* = 9.0 Hz, 2.4 Hz, 1H), 6.91 (td, *J* = 9.0 Hz, 2.4 Hz, 1H), 3.36-3.29 (m, 2H), 2.88-2.83 (m, 1H), 2.78-2.74 (m, 1H), 2.28 (brs, 1H), 1.09-1.01 (m, 21H); ^{13}C NMR (151 MHz, $CDCl_3$) δ 160.5 (d, *J* = 238.6 Hz), 136.2 (d, *J* = 13.0 Hz), 126.1 (d, *J* = 3.0 Hz), 124.4 (q, *J* = 284.9 Hz), 123.0, 119.7 (d, *J* = 10.1 Hz), 112.5, 108.6 (d, *J* = 24.6 Hz), 101.9, 97.8 (d, *J* = 26.0 Hz), 88.3, 57.0 (q, *J* = 31.7Hz), 40.3, 21.5, 18.5 (d, *J* = 2.9 Hz, 6C), 11,0 (3C); ^{19}F NMR (565 MHz, $CDCl_3$) δ -77.5 (s, 3F), -119.2--119.3 (m, 1F); HRMS (ESI) m/z: $[M+H]^+$ Calcd for $C_{23}H_{31}F_4N_2Si^+$, 439.2187, found 439.2187; HPLC analysis CHIRALCEL OD-3 (Hexane:*i*PrOH = 98/2, 0.6 mL/min, 40 °C, 254 nm) 15.3 min (minor), 16.9 min (major). (Ee% was measured after deprotection of silyl groups with TBAF.); **Configuration Assignment**: The absolute configuration was assigned as (*R*) by an analogy of absolute configuration of **7**.

(*R*)-7-chloro-1-(trifluoromethyl)-1-((triisopropylsilyl)ethynyl)-2,3,4,9-tetrahydro-1H-pyrido[3,4-b] indole (**4n**)

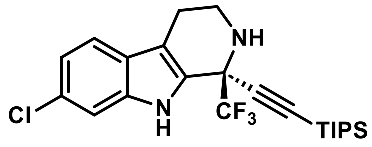

4n was prepared according to **General procedure E**, starting from **1n** (0.1 mmol, 22.8 mg, 1.0 eq.) and ketone **2c** (0.12 mmol, 33.4 mg, 1.2 eq.) with catalyst (*R*)**-3b** (0.010 mmol, 8.8 mg, 0.10 eq.) and MS4A (22.8 mg, 100 wt% for **1n**). The reaction mixture was stirred at 40 °C for 185 h. **4n** was afforded 28.2 mg, 0.062 mmol, 62% yield.

$[\alpha]_D^{23}$ =+81.6 (*c* = 0.59, $CHCl_3$, 91% ee); R_f = 0.63 (Hexane/EtOAc= 5/1); light yellow oil; ^{1}H NMR (600 MHz, $CDCl_3$) δ 8.04 (brs, 1H), 7.44 (d, *J* = 8.4 Hz, 1H), 7.38 (d, *J* = 1.2 Hz, 1H), 7.11 (dd, *J* = 8.4 Hz, 1.2 Hz, 1H), 3.37-3.27 (m, 2H), 2.87-2.73 (m, 2H), 2.31 (brs, 1H), 1.09-1.01 (m, 21H); ^{13}C NMR (151 MHz, $CDCl_3$) δ 136.5, 128.9, 126.5, 125.0, 124.3 (q, *J* = 284.8 Hz), 120.6, 119.8, 112.6, 111.3, 101.8, 88.5, 57.0 (q, *J* = 30.4Hz), 40.3, 21.4, 18.5 (6C), 11.0 (3C); ^{19}F NMR (565 MHz, $CDCl_3$)

δ -77.5; HRMS (ESI) m/z: $[M+H]^+$ Calcd for $C_{23}H_{31}ClF_3N_2Si^+$, 455.1892, 457.1862, found 455.1892, 457.1862; HPLC analysis CHIRALCEL AD-3 (Hexane:*i*PrOH = 90/10, 1.0mL/min, 40 °C, 254 nm) 12.7 min (major), 25.0 min (minor). (Ee% was measured after deprotection of silyl groups with TBAF.); **Configuration Assignment**: The absolute configuration was assigned as (*R*) by an analogy of absolute configuration of **7**.

(*R*)-7-bromo-1-(trifluoromethyl)-1-((triisopropylsilyl)ethynyl)-2,3,4,9-tetrahydro-1H-pyrido[3,4-b] indole (**4o**)

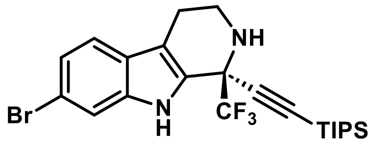

4o was prepared according to **General procedure E**, starting from **1o** (0.1 mmol, 23.9 mg, 1.0 eq.) and ketone **2c** (0.12 mmol, 33.4 mg, 1.2 eq.) with catalyst (*R*)**-3b** (0.010 mmol, 8.8 mg, 0.10 eq.) and MS4A (23.9 mg, 100 wt% for **1o**). The reaction mixture was stirred at 40 °C for 185 h. **4o** was afforded 28.0 mg, 0.056 mmol, 56% yield.

$[\alpha]_D^{23}$ =+184.3 (*c* = 0.16, $CHCl_3$, 90% ee); R_f = 0.63 (Hexane/EtOAc= 5/1); light yellow oil; ^{1}H NMR (600 MHz, $CDCl_3$) δ 8.04 (brs, 1H), 7.55 (d, *J* = 1.2 Hz, 1H), 7.40 (d, *J* = 8.4 Hz, 1H), 7.24 (dd, *J* = 8.4 Hz, 1.2 Hz, 1H), 3.37-3.27 (m, 2H), 2.87-2.74 (m, 2H), 2.31 (brs, 1H), 1.08-1.01 (m, 21H),; ^{13}C NMR (151 MHz, $CDCl_3$) δ 136.9, 126.5, 125.3, 124.2 (q, *J* = 284.8 Hz), 123.2, 120.2, 116.6, 114.3, 112.6, 101.7, 88.5, 57.0 (q, *J* = 30.4Hz), 40.2, 21.4, 18.5 (6C), 10.9 (3C); ^{19}F NMR (565 MHz, $CDCl_3$) δ -77.5; HRMS (ESI) m/z: $[M+H]^+$ Calcd for $C_{23}H_{31}BrF_3N_2Si^+$, 499.1387, 501.1367, found 499.1387, 501.1366; HPLC analysis CHIRALCEL AD-3 (Hexane:*i*PrOH = 80/20, 0.8mL/min, 40 °C, 254 nm) 10.1 min (major), 20.2 min (minor). (Ee% was measured after deprotection of silyl groups with TBAF.); **Configuration Assignment**: The absolute configuration was assigned as (*R*) by an analogy of absolute configuration of **7**.

(*R*)-1-(*tert*-butyldimethylsilyl) ethynyl)-1-(trifluoromethyl)-2,3,4,9-tetrahydro-1H-pyrido[3,4-b] indole (**4ab**)

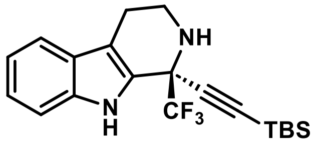

4ab was prepared according to **General procedure E**, starting from **1a** (0.1 mmol, 16.0 mg, 1.0 eq.) and ketone **2b** (0.12 mmol, 28.4 mg, 1.2 eq.) with catalyst (*R*)**-3a** (0.005 mmol, 4.4 mg, 0.05 eq.). The

reaction mixture was stirred at 40 °C for 36 h. **4ab** was afforded 19.8 mg, 0.052 mmol, 52% yield. $[\alpha]_D^{23}$ =+44.4 (*c* = 0.82, $CHCl_3$, 83% ee); R_f = 0.62 (Hexane/EtOAc= 5/1); white solid (m.p. 93.6-94.7 °C); ^1H NMR (600 MHz, $CDCl_3$) δ 8.06 (brs, 1H), 7.55 (d, *J* = 7.8 Hz, 1H), 7.39 (d, *J* = 7.8 Hz, 1H), 7.24 (td, *J* = 7.8 Hz, 1.2 Hz, 1H), 7.14 (td, *J* = 7.8 Hz, 1.2 Hz, 1H), 3.36-3.27 (m, 2H), 2.90-2.85 (m, 1H), 2.81-2.77 (m, 1H), 2.30 (brs, 1H), 0.92 (s, 9H), 0.10 (s, 6H),; ^{13}C NMR (151 MHz, $CDCl_3$) δ 136.2, 126.4, 125.6, 124.4 (q, *J* = 284.8 Hz), 123.1, 119.8, 118.9, 112.5, 111.3, 100.5, 89.9, 57.0 (q, *J* = 31.7 Hz), 40.3, 25.9 (3C), 21.5, 16.5, -4.9 (2C); ^{19}F NMR (565 MHz, $CDCl_3$) δ -77.4; HRMS (ESI) m/z: $[M+H]^+$ Calcd for $C_{20}H_{26}F_3N_2Si^+$ 379.1812, found 379.1812; HPLC analysis CHIRALCEL AD-3 (Hexane:*i*PrOH = 90/10, 1.0 mL/min, 30 °C, 254 nm) 12.6 min (major), 17.4 min (minor). (Ee% was measured after deprotection of silyl groups with TBAF.); **Configuration Assignment**: The absolute configuration was assigned as (*R*) by derivatization to **7** by deprotection of TBS.

Pictet-spengler reaction at 1 mmol scale

To a solution of tryptamine **1a** (1.0 mmol, 160.0 mg, 1.0 eq.), catalyst (*R*)**-3b** (0.05 mmol, 44.0 mg, 0.05 eq.) and MS4A (160.0 mg, 100 wt% for **1a**) in DCM (5.0 ml, 0.2 M), ketone **2** (1.2 mmol, 334.0 mg, 1.2 eq.) was added at room temperature and the reaction mixture was heated to 40 °C using a heating block for 42 h. Then, the reaction mixture was quenched with sat. $NaHCO_3$ aq. at room temperature. The resulting mixture was extracted with EtOAc (3 x 20 mL). Combined organic layers were dried over Na_2SO_4, filtrated, and concentrated under reduced pressure. The residue was purified by flash column chromatography on silica gel (Hexane/DCM = 8/1 to 9/2) to give **4ac** (400.9 mg) in 95% yield, 91% ee.

4. Reaction from intermediate imine 5 (Scheme 2)

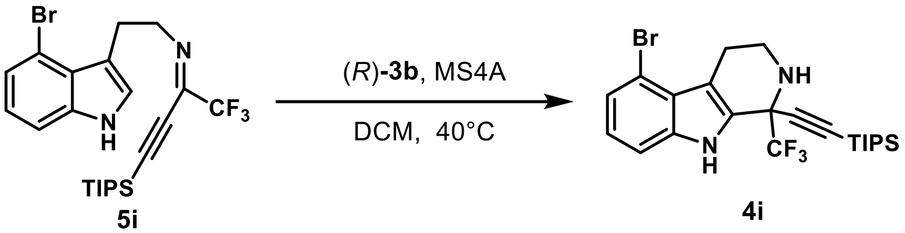

5i 4i

To a solution of tryptamine **5i** (20.4 mg, 0.041 mmol, 1.0 eq.) and MS4A (9.76 mg) in DCM (204 µL, 0.2 M), catalyst (*R*)**-3b** (1.96 mg, 0.0021 mmol, 0.05 eq.) was added at room temperature. The mixture was allowed to heat 40 °C using a heating block and stirred for 90 h. The reaction mixture was quenched with sat. $NaHCO_3$ aq. at room temperature. The resulting mixture was extracted with EtOAc (3 x 5 mL). Combined organic layers were dried over Na_2SO_4, filtrated, and concentrated under reduced pressure. The residue was purified by flash column chromatography on silica gel

(Hexane/DCM = 8/1 to 9/2) and **4i** was afforded 17.0 mg, 0.034 mmol, 83% yield.

(*E*)-N-(2-(4-bromo-1H-indol-3-yl)ethyl)-1,1,1-trifluoro-4-(triisopropylsilyl)but-3-yn-2-imine (**5i**)

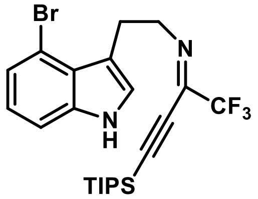

R_f = 0.41 (Hexane/EtOAc= 5/1); yellow foam; ^{1}H NMR (600 MHz, $CDCl_3$) δ 8.08 (brs, 1H), 7.28 (dd, *J* = 7.8 Hz, 0.6 Hz, 1H), 7.25 (dd, *J* = 7.8 Hz, 0.6 Hz, 1H), 7.01 (d, *J* = 1.8 Hz, 1H), 6.98 (t, *J* = 7.8 Hz, 1H), 4.16 (td, *J* = 7.2 Hz, 1.2 Hz, 2H), 3.46 (td, *J* = 7.2 Hz, 1.2 Hz, 2H), 1.07-0.99 (m, 21H); ^{13}C NMR (151 MHz, $CDCl_3$) δ 142.1 (q, *J* = 37.6 Hz), 137.6, 125.4, 124.1, 124.0, 122.8, 118.3 (q, *J* = 277.7 Hz), 114.3, 114.2, 110.4, 106.4, 93.1, 57.8, 26.4, 18.5 (6C), 10.9 (3C); ^{19}F NMR (565 MHz, $CDCl_3$) δ -71.4; HRMS (ESI) m/z: $[M+H]^+$ Calcd for $C_{23}H_{30}BrF_3N_2Si^+$, 499.1387, 501.1366, found 499.1387, 501.1366.

5. Derivatization (Scheme 3 and 4)

tert-butyl-(*R*)-1-(trifluoromethyl)-1-((triisopropylsilyl)ethynyl)-1,2,3,4-tetrahydro-9H-pyrido[3,4-b]-indole-9-carboxylate (**S5**)

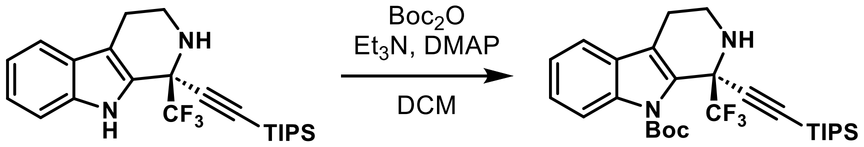

4ac **S5**

To a solution of **4ac** (551 mg, 1.31 mmol) and Boc_2O (1.43 g, 6.55 mmol, 5.0 eq.) in DCM (5.24 mL, 0.25 M), Et_3N (1.0 mL, 7.21 mmol, 5.5 eq.) and DMAP (16 mg, 0.131 mmol, 0.1 eq.) were added at room temperature. After the reaction mixture was stirred at the room temperature for 12 h, the reaction mixture was concentrated under reduced pressure. The residue was purified by flash column chromatography on silica gel (Hexane/EtOAc = 30/1 to 25/1) to give **S5** (633.9 mg) in 93% yield. $[\alpha]_D^{23}$ =+67.8 (*c* = 0.11, $CHCl_3$, 91% ee); R_f = 0.62 (Hexane/EtOAc= 5/1); White solid (m.p. 92.0-92.8 °C); ^{1}H NMR (600 MHz, $CDCl_3$) δ 7.90 (d, *J* = 7.8 Hz, 1H), 7.49 (d, *J* = 7.8 Hz, 1H), 7.33 (td, *J* = 7.8 Hz, 1.2Hz, 1H), 7.23 (td, *J* = 7.8 Hz, 1.2Hz, 1H), 3.42-3.29 (m, 2H), 2.92-2.73 (m, 2H), 2.40 (brs, 1H), 1.66 (s, 9H), 1.10 (m, 21H); ^{13}C NMR (151 MHz, $CDCl_3$) δ 149.2, 137.0, 127.8, 126.7, 125.3, 124.0 (q, *J* = 284.8 Hz), 122.3, 121.7, 118.9, 114.8, 103.4, 86.6, 83.8, 58.5 (q, *J* = 30.4Hz), 40.0, 28.1 (3C), 22.5, 18.6 (6C), 11.2 (3C); ^{19}F NMR (565 MHz, $CDCl_3$) δ -75.3; HRMS (ESI) m/z: [M +

H]$^+$ calculated for $C_{28}H_{40}F_3N_2O_2Si^+$, 521.2806, found 521.2806; HPLC analysis CHIRALCEL OD-3 (Hexane:*i*PrOH = 98/2, 1.0 mL/min, 40 °C, 254 nm) 9.6 min (minor), 10.7 min (major). (Ee% was measured after its derivatization to compound **6** by deprotection of TIPS group.)

tert-butyl(*R*)-1-ethynyl-1-(trifluoromethyl)-1,2,3,4-tetrahydro-9H-pyrido[3,4-b]indole-9 carboxylate (**6**)

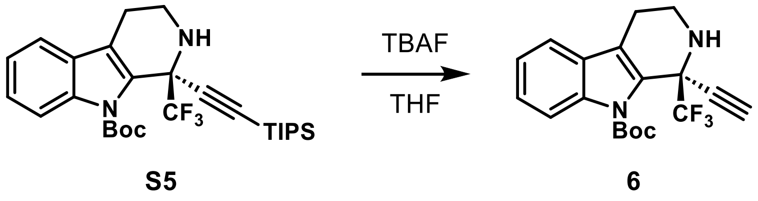

To a solution of **S5** (625 mg, 1.2 mmol, 1.0 eq.) in THF (3.0 mL, 0.40 M), TBAF (1.0 M in THF, 6 mL, 6.0 mmol, 5.0 eq.) was added at room temperature. The reaction mixture was stirred in an oil bath at 40 °C for 40 min. Then the reaction mixture was quenched with sat. NH_4Cl aq. The resulting mixture was extracted with EtOAc (3 x 20 mL) and washed with brine. Combined organic layers were dried over Na_2SO_4, filtrated, and concentrated under reduced pressure. The residue was purified by flash column chromatography on silica gel (Hexane/EtOAc = 8/1) to give **6** (415 mg) in 91% yield.
$[\alpha]_D^{23}$ =+16.9 (*c* = 0.17, $CHCl_3$, 91% ee); R_f = 0.39 (Hexane/EtOAc= 5/1); White solid (m.p. 145.3-146.0 °C); ^1H NMR (600 MHz, $CDCl_3$) δ 7.93 (d, *J* = 7.8 Hz, 1H), 7.49 (d, *J* = 7.8 Hz, 1H), 7.35 (td, *J* = 7.8 Hz, 1.2Hz, 1H), 7.24 (td, *J* = 7.8 Hz, 1.2Hz, 1H), 3.41-3.30 (m, 2H), 2.93-2.75 (m, 2H), 2.70 (s, 1H), 2.44 (brs, 1H), 1.69 (s, 9H); ^{13}C NMR (151 MHz, $CDCl_3$) δ 150.0, 137.2, 127.5, 125.7, 125.5, 123.8 (q, *J* = 284.8 Hz), 122.5, 122.0, 118.9, 114.8, 84.8, 80.7, 73.9, 83.8, 57.9 (q, *J* = 30.4Hz), 38.8, 28.2 (3C), 22.3; ^{19}F NMR (565 MHz, $CDCl_3$) δ -74.8; HRMS (ESI) m/z: [M + H]$^+$ calculated for $C_{19}H_{20}F_3N_2O_2^+$, 365.1471, found 365.1471; HPLC analysis CHIRALCEL OD-3 (Hexane:*i*PrOH = 98/2, 1.0 mL/min, 40 °C, 254 nm) 9.6 min (minor), 10.7 min (major).

(*R*)-1-ethynyl-1-(trifluoromethyl)-2,3,4,9-tetrahydro-1H-pyrido[3,4-b] indole (**7**)

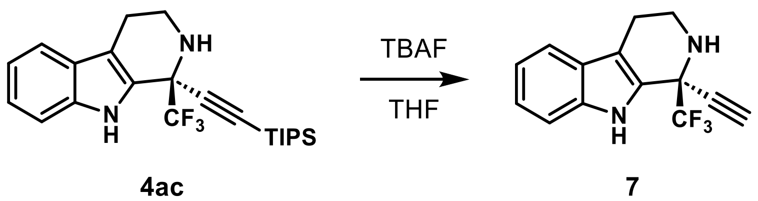

To a solution of **4ac** (252 mg, 0.60 mmol, 1.0 eq.) in THF (1.2 mL, 0.40 M), TBAF (1.0 M in THF, 3 mL, 3.0 mmol, 5.0 eq.) was added at room temperature. The reaction mixture was stirred in an oil bath at 40 °C for 40 min. Then the reaction mixture was quenched with sat. NH_4Cl aq. The resulting mixture

was extracted with EtOAc (3 x 20 mL) and washed with brine. Combined organic layers were dried over Na_2SO_4, filtrated, and concentrated under reduced pressure. The residue was purified by flash column chromatography on silica gel (Hexane/EtOAc = 8/1) to give **7** (155 mg) in 98% yield.
$[\alpha]_D^{23}$ =+82.2 (*c* = 0.01, $CHCl_3$, 91% ee); R_f = 0.39 (Hexane/EtOAc= 5/1); White solid (m.p. 165.3-166.0 °C); ^1H NMR (600 MHz, $CDCl_3$) δ 8.09 (brs, 1H), 7.54 (d, *J* = 7.8 Hz, 1H), 7.37 (d, *J* = 7.8 Hz, 1H), 7.24 (td, *J* = 7.8 Hz, 1.2Hz, 1H), 7.14 (td, *J* = 7.8 Hz, 1.2Hz, 1H), 3.37-3.27 (m, 2H), 2.90-2.77 (m, 2H), 2.59 (s, 1H), 2.27 (brs, 1H); ^{13}C NMR (151 MHz, $CDCl_3$) δ 136.2, 126.3, 125.1, 124.3 (q, *J* = 284.8 Hz), 123.3, 120.0, 119.0, 112.9, 111.3, 79.2, 74.5, 56.5 (q, *J* = 30.4Hz), 40.3, 21.4; ^{19}F NMR (565 MHz, $CDCl_3$) δ -77.4; HRMS (ESI) m/z: $[M + H]^+$ calculated for $C_{14}H_{21}F_3N_2^+$, 265.0947 found265.0947; HPLC analysis CHIRALCEL AD-3 (Hexane:*i*PrOH = 90/10, 1.0 mL/min, 30 °C, 254 nm) 12.6 min (major), 17.4 min (minor).
ORTEP drawing of (*R*)-**7** showing thermal elipsoids at the 50% probability level. CCDC No. 2402262. Recrystallization from hexane/1,2-DCE.

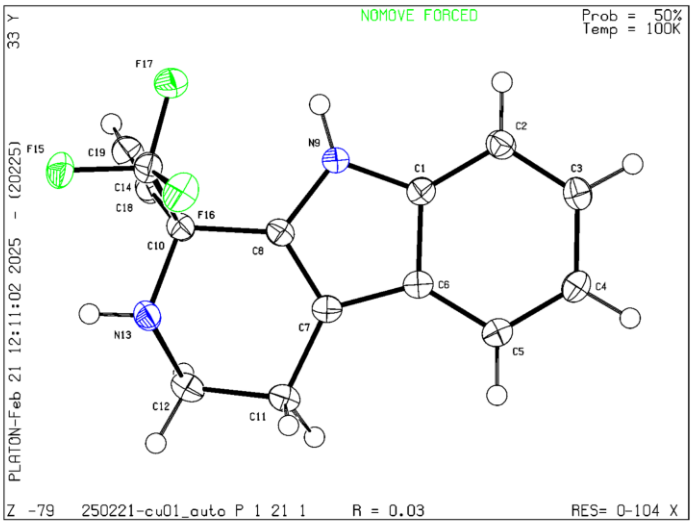

(*R*)-1-(trifluoromethyl)-1-vinyl-2,3,4,9-tetrahydro-1H-pyrido[3,4-b] indole (**8**)

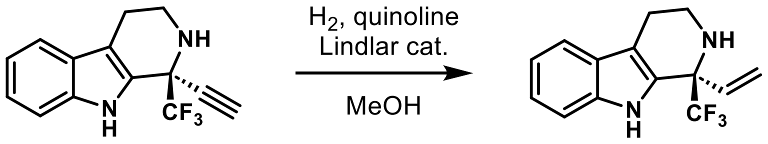

7 **8**

To a solution of **7** (13.2 mg, 0.05 mmol, 1.0 eq.) and quinoline (21.3 µL, 8µ for Lindler cat 1 mg) in MeOH (0.50 mL, 0.1 M), Lindlar cat. (2.64 mg, 20 wt%) was added at room temperature under an atmosphere of nitrogen. The reaction vessel was sealed with a septum and purged with H_2 three times. The reaction mixture was stirred at the same temperature for 1 h 10 min. The reaction mixture was filtered through a plug of celite, washed with EtOAc, and concentrated in vacuo. The residue was

purified by flash column chromatography on silica gel (Hexane/EtOAc = 5/1) to give **8** (11.2 mg) in 84% yield.

$[\alpha]_D^{23}$ =+58.9 (*c* = 0.48, $CHCl_3$, 90% ee); R_f = 0.39 (Hexane/EtOAc= 5/1); White solid (m.p. 82.0-82.7 °C); ^{1}H NMR (600 MHz, $CDCl_3$) δ 7.93 (brs, 1H), 7.56 (d, *J* = 7.8 Hz, 1H), 7.37 (d, *J* = 7.8 Hz, 1H), 7.23 (td, *J* = 7.8 Hz, 1.2Hz, 1H), 7.14 (td, *J* = 7.8 Hz, 1.2Hz, 1H), 6.10 (dd, *J* = 17.4 Hz, 10.8 Hz, 1H), 5.46 (d, *J* = 10.8 Hz, 1H), 5.10 (d, *J* = 17.4 Hz, 1H), 3.29-3.25 (m, 1H), 3.11-3.07 (m, 1H), 2.90-2.85 (m, 1H), 2.77-2.73 (m, 1H), 1.92 (brs, 1H); ^{13}C NMR (151 MHz, $CDCl_3$) δ 136.1, 133.9, 126.4, 126.3 126.0 (q, *J* = 284.9 Hz), 122.9, 120.8, 119.7, 118.7, 113.8, 111.2, 62.6 (q, *J* = 27.5Hz), 39.1, 22.0; ^{19}F NMR (565 MHz, $CDCl_3$) δ -76.3; HRMS (ESI) m/z: $[M + H]^+$ calculated for $C_{14}H_{14}F_3N_2^+$, 267.1104, found 267.1104; HPLC analysis CHIRALCEL AD-3 (Hexane:*i*PrOH = 90/10, 1.0 mL/min, 30 °C, 254 nm) 7.4min(minor), 8.4min (major).

(*R*)-1-(phenylethynyl)-1-(trifluoromethyl)-2,3,4,9-tetrahydro-1H-pyrido[3,4-b] indole (**9**)

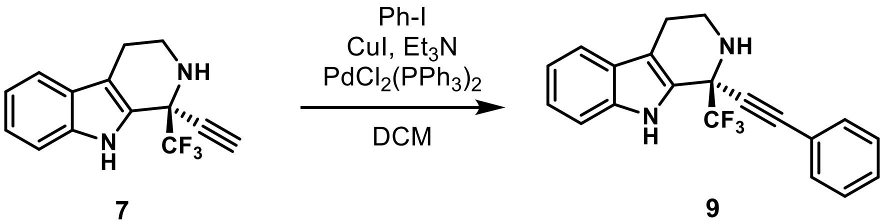

7 **9**

To a solution of **7** (26.4 mg, 0.1 mmol, 1.0 eq.), iodobenzene (13.4 μL, 0.12 mmol, 1.2 eq.), and CuI (1,0 mg, 0.05 mmol, 0.05 mmol, 0.05 eq.) in NEt_3 (625 μL, 0.16 M), $PdCl_2(PPh_3)_2$ (3.5 mg, 0.05 mmol) was added at room temperature. The reaction mixture was stirred at the same temperature for 24 h. Then the reaction mixture was filtrated, extracted with EtOAc (3 x 5 mL) and washed with brine (5 mL). Combined organic layers were dried over Na_2SO_4, filtrated, and concentrated under reduced pressure. The residue was purified by flash column chromatography on silica gel (Hexane/EtOAc = 15/1) to give **9** (29.6 mg) in 87% yield.

$[\alpha]_D^{23}$ =+51.6 (*c* = 0.20, $CHCl_3$, 91% ee); R_f = 0.36 (Hexane/EtOAc= 5/1); White solid (m.p. 173.6-174.3 °C); ^{1}H NMR (600 MHz, $CDCl_3$) δ 8.16 (brs, 1H), 7.57 (d, *J* = 7.8 Hz, 1H), 7.43 (td, *J* = 7.8 Hz, 1.2 Hz, 2H) 7.39 (d, *J* = 7.8 Hz, 1H), 7.34 (tt, *J* = 7.8 Hz, 1.2Hz, 1H), 7.30 (tt, *J* = 7.8 Hz, 1.2Hz, 2H), 7.25 (td, *J* = 7.8 Hz, 1.2 Hz, 1H), 7.15 (td, *J* = 7.8 Hz, 1.2 Hz, 1H), 3.42-3.35 (m, 2H), 2.94-2.81 (m, 2H), 2.39 (brs, 1H); ^{13}C NMR (151 MHz, $CDCl_3$) δ 136.2, 132.0 (2C), 129.2, 128.4 (2C), 126.4, 125.8, 124.5 (q, *J* = 284.8 Hz), 123.2, 121.3, 119.9, 119.0, 112.6, 111.3, 86.0, 84.2, 57.1 (q, *J* = 30.4Hz), 40.5, 21.6; ^{19}F NMR (565 MHz, $CDCl_3$) δ -77.2; HRMS (ESI) m/z: $[M + H]^+$ calcd for $C_{20}H_{15}F_3N_2$+, 341.1260 found 341.1260; HPLC analysis CHIRALCEL OD-3 (Hexane:*i*PrOH = 90/10, 1.0 mL/min, 30 °C, 254 nm) 6.2min (major), 6.9min (minor).

(*R*)-1-ethyl-1-(trifluoromethyl)-2,3,4,9-tetrahydro-1H-pyrido[3,4-b] indole (**10**)

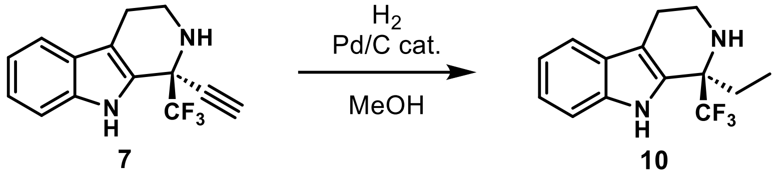

To a solution of **7** (13.2 mg, 0.05 mmol) in MeOH (625 μL, 0.08 M), Pd/C cat. was added (1.32 mg, 10 wt%) at room temperature under an atmosphere of nitrogen. The reaction vessel was sealed with a septum and purged with H_2 three times. The reaction mixture was stirred at the same temperature for 12 h. The reaction mixture was filtered through a plug of celite, washed with MeOH, and concentrated in vacuo. The residue was purified by flash column chromatography on silica gel (Hexane/EtOAc = 10/1) to give **10** (12.5 mg) in 93% yield.

$[\alpha]_D^{23}$ =+106.0 (*c* = 0.56, $CHCl_3$, 91% ee); R_f = 0.40 (Hexane/EtOAc= 5/1); White solid (m.p. 55.3-56.0 °C); ^{1}H NMR (600 MHz, $CDCl_3$) δ 7.88 (brs, 1H), 7.55 (d, *J* = 8.4 Hz, 1H), 7.37 (d, *J* = 8.4 Hz, 1H), 7.22 (td, *J* = 8.4 Hz, 1.2Hz, 1H), 7.13 (td, *J* = 8.4 Hz, 1.2Hz, 1H), 3.29-3.27 (m, 2H), 2.81-2.69 (m, 2H), 2.08-2.01 (m, 1H), 1.90-1.86 (m, 1H), 1.70 (brs, 1H), 0.72 (t, *J* = 7.8 Hz, 3H); ^{13}C NMR (151 MHz, $CDCl_3$) δ 136.1, 127.8, 127.4 (q, *J* = 287.7 Hz), 126.6 122.7, 119.6, 118.6, 114.3, 111.1, 59.8 (q, *J* = 26.1Hz), 40.5, 27.5, 22.2, 7.1; ^{19}F NMR (565 MHz, $CDCl_3$) δ -76.0; HRMS (ESI) m/z: $[M + H]^+$ calcd for $C_{14}H_{16}F_3N_2^+$, 269.1260, found 269.1260; HPLC analysis CHIRALCEL OD-3 (Hexane:*i*PrOH = 90/10, 1.0 mL/min, 30 °C, 254 nm) 5.1min (major), 9.1min (minor).

tert-butyl-(*R*)-2-allyl-1-ethynyl-1-(trifluoromethyl)-1,2,3,4-tetrahydro-9H-pyrido[3,4-b]indole-9-carboxylate (**11**)

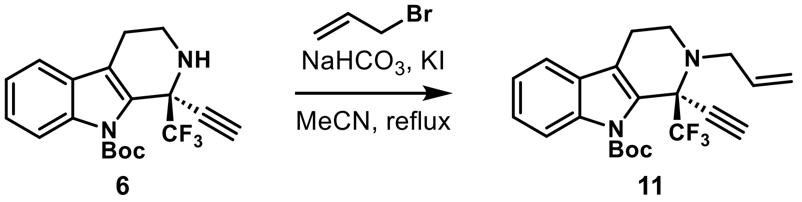

To a solution of **6** (72.8 mg, 0.20 mmol, 1.0 eq.), $NaHCO_3$ (83 mg, 1.0 mmol, 5.0 eq.) and KI (3.4 mg, 0.02 mmol, 0.1 eq.) in MeCN (600 μL, 0.33 M), allyl bromide (86 μL, 1.0 mmol, 5.0 eq.) was added at room temperature. The reaction mixture was stirred in an oil bath at 80 °C for 40 h. Then the reaction mixture was cooled to room temperature and quenched with brain. The resulting mixture was extracted with EtOAc (3 x 10 mL) and washed with brine. Combined organic layers were dried over Na_2SO_4, filtrated, and concentrated under reduced pressure. The residue was purified by flash column chromatography on silica gel (Hexane/EtOAc = 50/1) to give **11** (51.4 mg) in 64% yield.

$[\alpha]_D^{23}$ =+42.5 (*c* = 0.09, $CHCl_3$, 92% ee); R_f = 0.54 (Hexane/EtOAc= 5/1); colorless oil ; ^{1}H NMR

(600 MHz, $CDCl_3$) δ 7.96 (d, *J* = 8.4 Hz, 1H), 7.48 (d, *J* = 8.4 Hz, 1H), 7.34 (td, *J* = 8.4 Hz, 1.2Hz, 1H), 7.23 (td, *J* = 8.4 Hz, 1.2Hz, 1H), 5.93-5.87 (m, 1H), 5.30 (dq, *J* = 17.4 Hz, 1.8 Hz, 1H), 5.17 (dq, *J* = 10.2 Hz, 1.8 Hz, 1H), 3.60-3.57 (m, 1H), 3.44-3.40 (m, 1H), 3.18-3.14 (m, 1H), 2.94-2.90 (m, 1H), 2.79-2.74 (m, 2H), 2.67 (s, 1H), 1.70 (s, 9H); ^{13}C NMR (151 MHz, $CDCl_3$) δ 149.7, 136.9, 136.1, 127.4, 127.3, 125.4, 123.8 (q, *J* = 292.0 Hz), 122.4, 120.6, 118.8, 116.7, 115.3, 84.5, 75.3, 64.4 (q, *J* = 30.4Hz), 55.7, 43.5, 28.2 (3C), 21.5; ^{19}F NMR (565 MHz, $CDCl_3$) δ -64.5; HRMS (ESI) m/z: $[M + H]^+$ calculated for $C_{22}H_{24}F_3N_2O_2^+$, 405.1784, found 405.1784; HPLC analysis CHIRALCEL AD-3 (Hexane:*i*PrOH = 90/10, 1.0 mL/min, 30 °C, 254 nm) 4.0 min (major), 5.5 min (minor)

tert-butyl-(*R*)-1-(trifluoromethyl)-1-vinyl-1,2,3,4-tetrahydro-9H-pyrido[3,4-b]indole-9-carboxylate (**12**)

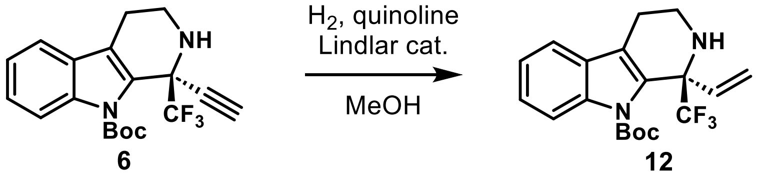

To a solution of **6** (324 mg, 0.89 mmol, 1.0 eq.) and quinoline (519 μL, 8μ for Lindler cat 1 mg) in MeOH (8.9 mL, 0.1 M), Lindlar cat. (64.9 mg, 20 wt%) was added at room temperature under an atmosphere of nitrogen. The reaction vessel was sealed with a septum and purged with H_2 three times. The reaction mixture was stirred at the same temperature for 1 h min. The reaction mixture was filtered through a plug of celite, washed with EtOAc, and concentrated in vacuo. In the main text, the residue was used with the next reaction without further purification.

Purification method: The residue was purified by flash column chromatography on silica gel (Hexane/EtOAc = 5/1) to give **12** (291 mg) in 89% yield.

$[\alpha]_D^{23}$ =+34.5 (*c* = 0.28); R_f = 0.38 (Hexane/EtOAc= 5/1); White solid (m.p. 58.6-59.3 °C); ^1H NMR (600 MHz, $CDCl_3$) δ 7.91 (d, *J* = 7.8 Hz, 1H), 7.50 (d, *J* = 7.8 Hz, 1H), 7.34 (td, *J* = 7.8 Hz, 1.2Hz, 1H), 7.25 (td, *J* = 7.8 Hz, 1.2Hz, 1H), 6.65 (dd, *J* = 17.4 Hz, 10.8 Hz, 1H), 5.40 (d, *J* = 10.8 Hz, 1H), 5.39 (d, *J* = 17.4 Hz, 1H), 3.25-3.16 (m, 2H), 2.89-2.76 (m, 2H), 1.98 (brs, 1H), 1.65 (s, 9H); ^{13}C NMR (151 MHz, $CDCl_3$) δ 150.2 137.0, 135.9, 128.4, 127.7 125.8 (q, *J* = 287.7 Hz), 125.1, 122.2, 121.2, 118.6, 117.1, 114.8, 84.4, 63.6 (q, *J* = 27.5Hz), 38.0, 28.1 (3C), 22.6; ^{19}F NMR (565 MHz, $CDCl_3$) δ -73.1,; HRMS (ESI) m/z: $[M + H]^+$ calculated for $C_{19}H_{22}F_3N_2O_2^+$, 367.1628, found 367.1628.

tert-butyl-(*R*)-1-(2-hydroxyethyl)-1-(trifluoromethyl)-1,2,3,4-tetrahydro-9H-pyrido[3,4-b]indole-9-carboxylate (**13**)

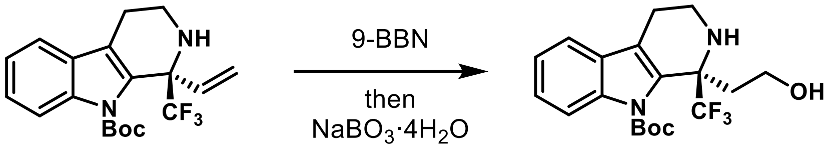

12 **13**

To a solution of crude **12** (36.6 mg, 0.1 mmol) in THF (100 μL, 1.0 M) 9-BBN (0.5 M in THF, 400 μL, 0.2 mmol) was added at 0 °C. After the reaction mixture was stirred in a heating block at 40 °C for 1 h, cooled to 0 °C. Then $NaBO_3 \cdot 4H_2O$ (77 mg, 0.5 mmol, 5 eq.) was added to the reaction vessel and the resulting mixture was stirred at room temperature for 5 h. The reaction mixture was quenched with sat. $Na_2S_2O_3$ aq. at 0 °C. The resulting mixture was extracted with EtOAc (3 x 5 mL). Combined organic layers were dried over Na_2SO_4, filtrated, and concentrated under reduced pressure. The residue was purified by flash column chromatography on silica gel (Hexane/EtOAc = 3/1) to give **13** (28 mg) in 65% yield (2 steps from compound **6**).

$[\alpha]_D^{23}$ =+40.5 (*c* = 0.05, $CHCl_3$, 91% ee); R_f = 0.19 (Hexane/EtOAc= 2/1); Yellow solid (m.p. 73.6-74.3 °C) ; ^1H NMR (600 MHz, $CDCl_3$) δ 7.80 (d, *J* = 7.8 Hz, 1H), 7.52 (d, *J* = 7.8 Hz, 1H), 7.34 (td, *J* = 7.8 Hz, 1.2Hz, 1H), 7.25 (td, *J* = 7.8 Hz, 1.2Hz, 1H), 3.82-3.71 (m, 2H), 3.50-3.00 (brs, 1H, OH), 3.23-3.14 (m, 4H), 2.90-2.81 (m, 2H), 1.96 (ddd, *J* = 14.4 Hz, 10.8 Hz, 3.6 Hz 1H), 1.67 (s, 9H); ^{13}C NMR (151 MHz, $CDCl_3$) δ 150.6, 136.7, 127.7, 126.7, 126.4 (q, *J* = 290.7 Hz), 125.1, 122.2, 121.9, 118.7, 114.4, 84.5, 63.8 (q, *J* = 26.0Hz), 60.6, 38.9, 36.7, 28.0 (3C); ^{19}F NMR (565 MHz, $CDCl_3$) δ -73.1; HRMS (ESI) m/z: $[M + H]^+$ calculated for $C_{19}H_{24}F_3N_2O_3^+$, 385.1734, found 385.1734; HPLC analysis CHIRALCEL OD-3 (Hexane:*i*PrOH = 98/2, 0.6 mL/min, 40 °C, 254 nm) 22.7 min (major), 25.5 min (minor).

Note: In our ^1H NMR conditions, the integral value for the hydroxyl group is observed to be low.

(*R*)-11b-(trifluoromethyl)-5,6,11,11b-tetrahydro-3H-indolizino[8,7-b] indole (**S6**)

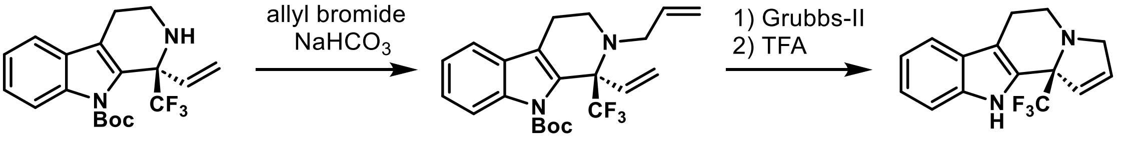

12 **14** **S6**

To a solution of **12** (72.8 mg, 0.20 mmol, 1.0 eq.), $NaHCO_3$ (83 mg, 1.0 mmol, 5.0 eq.) and KI (3.4 mg, 0.02 mmol, 0.1 eq.) in MeCN (600 μL, 0.33 M), allyl bromide (86 μL, 1.0 mmol, 5.0 eq.) was added at room temperature. The reaction mixture was stirred in an oil bath at 80 °C for 40 h. Then the reaction mixture was cooled to room temperature and quenched with brain. The resulting mixture was extracted with EtOAc (3 x 10 mL) and washed with brine. Combined organic layers were dried over Na_2SO_4, filtrated, and concentrated under reduced pressure. The residue was filtered through a pad of

silica gel (Hexane/EtOAc = 50/1) to give crude **14.** The crude compound was used with the next reaction without further purification.

A solution of **14** (1.0 eq.) in DCM (6.4 mL) was added Grubbs-II catalyst (6.79 mg, 0.008 mmol.) at room temperature. The reaction mixture was stirred in an oil bath at 40 °C for 2 h. Then the reaction mixture was concentrated under reduced pressure. The residue was roughly purified by flash column chromatography on silica gel (Hexane/EtOAc = 50/1) to remove Grubbs-II catalyst**.** The result product was solved in DCM (0.64 mL) and the mixture was added Trifluoroacetic acid (0.64mL) at room temperature. After stirring it for 14 h at room temperature, the reaction mixture was quenched with sat. $NaHCO_3$ aq.. The resulting mixture was extracted with EtOAc (3 x 5 mL) and washed with brine (5 mL). Combined organic layers were dried over Na_2SO_4, filtrated, and concentrated under reduced pressure. The residue was purified by flash column chromatography on silica gel (Hexane/EtOAc = 20/1) to give **S6** (27.3 mg) in 49% yield with 4 steps from **6**.
$[\alpha]_D^{23}$ =+289.8 (*c* = 0.11, $CHCl_3$, 91% ee); R_f = 0.32 (Hexane/EtOAc= 5/1); Yellow oil; ^{1}H NMR (600 MHz, $CDCl_3$) δ 7.98 (brs, 1H), 7.55 (d, *J* = 7.8 Hz, 1H), 7.37 (d, *J* = 7.8 Hz, 1H) 7.22 (td, *J* = 7.8 Hz, 1.2 Hz, 1H), 7.13 (td, *J* = 7.8 Hz, 1.2Hz, 1H), 6.07-6.06 (m, 1H), 5.92-5.90 (m, 1H), 4.01-3.98 (m, 1H), 3.82-3.80 (m, 1H), 3.44-3.33 (m, 2H), 3.04-2.99 (m, 1H), 2.62-2.58 (m, 1H); ^{13}C NMR (151 MHz, $CDCl_3$) δ 136.3, 132.9, 129.0, 126.7, 125.9 (q, *J* = 284.8 Hz), 125.8, 122.8, 119.8, 118.8, 111.6, 111.2, 71.2 (q, *J* = 30.4Hz), 57.4, 43.9, 16.2; ^{19}F NMR (565 MHz, $CDCl_3$) δ -75.6; HRMS (ESI) m/z: $[M + H]^+$ calcd for $C_{15}H_{14}F_3N_2^+$, 279.1104, found 279.1104; HPLC analysis CHIRALCEL OD-3 (Hexane:*i*PrOH = 90/10, 1.0 mL/min, 30 °C, 254 nm) 5.3min (major), 8.4min (minor).

(*R*)-11b-(trifluoromethyl)-2,3,5,6,11,11b-hexahydro-1H-indolizino[8,7-b] indole (**15**)

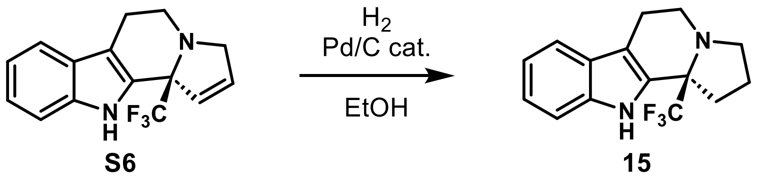

To a solution of **S6** (23.4 mg, 0.084 mmol) in MeOH (1.05 mL, 0.08 M) Pd/C cat. (2.34 mg, 10 wt%) was added at room temperature under an atmosphere of nitrogen. The reaction vessel was sealed with a septum and purged with H_2 three times. The reaction mixture was stirred at the same temperature for 24 h. The reaction mixture was filtered through a plug of celite, washed with MeOH, and concentrated in vacuo. The residue was purified by flash column chromatography on silica gel (Hexane/EtOAc = 20/1) to give **15** (11.1 mg) in quant.
$[\alpha]_D^{23}$ =+120.2 (*c* = 0.04, $CHCl_3$, 91% ee); R_f = 0.32 (Hexane/EtOAc= 5/1); White solid (m.p. 83.6-84.3 °C); ^{1}H NMR (600 MHz, $CDCl_3$) δ 7.91 (brs, 1H), 7.55 (d, *J* = 7.8 Hz, 1H), 7.35 (d, *J* = 7.8 Hz,

1H) 7.22 (td, J = 7.8 Hz, 1.2 Hz, 1H), 7.13 (td, J = 7.8 Hz, 1.2Hz, 1H), 63.38-3.30 (m, 2H), 3.12-3.10 (m, 1H), 2.98-2.91 (m, 2H), 2.62-2.54 (m, 2H), 2.03-1.92 (m, 2H), 1.79-1.74 (m, 1H);^{13}C NMR (151 MHz, $CDCl_3$) δ 136.3, 130.3, 127.1 (q, J = 284.8 Hz), 126.4, 122.7, 119.7, 118.7, 111.4, 111.0, 63.5 (q, J = 27.5 Hz), 51.1, 44.1, 35.2, 23.7, 15.9; ^{19}F NMR (565 MHz, $CDCl_3$) δ -76.2,; HRMS (ESI) m/z: $[M + H]^+$ calcd for $C_{15}H_{16}F_3N_2^+$, 281.1260, found 281.1260; HPLC analysis CHIRALCEL OD-3 (Hexane:*i*PrOH = 90/10, 1.0 mL/min, 30 °C, 254 nm) 4.5 min (major), 10.3 min (minor).

6. Reference

[1] E. K. Olesen, E. Hansen, L. K. Moodie, J. Isaksson, K. Sepčić, M. Cergolj, J. Svenson, J. H.Andersen, *Org. Biomol. Chem.* **2016**, *14*, 1629–1640.

[2] Q. V. Vo, C. Trenerry, S. Rochfort, J. Wadeson, C. Leyton, A. B. Hughes, *Bioorg. Med. Chem.* **2014**, *22*, 856–864.

[3] K. C. Nicolaou, A. Krasovskiy, U, Majumder, V. É. Trépanier. D. Y.-K. Chen, *J. Am. Chem. Soc.* **2009**, *131*, 3690–3699.

[4] G. Hi, B. List, M. Christmann, *Angew. Chem. Int. Ed.* **2021**, 60, 13591–13596.

[5] T. Hara, S. R. Durell, M. C. Myers, D. H. Appella, *J. Am. Chem. Soc*. **2006**, *128,* 1995–2004.

[6] L. Feng, T. Hu, S. Zhang, H-Y. Xiong, G. Zhang, *Org. Lett.* **2019**, *21, 23*, 9487–9492.

7. X-ray Structure Reports for 7

Table S1 Crystal data and structure refinement for 7

Identification code	**7**
Empirical formula	C14H11F3N2
Formula weight	264.25
Temperature/K	99.95(10)
Crystal system	monoclinic
Space group	P21
a/Å	7.63934(8)
b/Å	6.98708(9)
c/Å	11.29142(14)
α/°	90
β/°	91.5708(11)
γ/°	90
Volume/Å3	602.472(13)
Z	2
ρcalcg/cm3	1.457

μ/mm-1 1.031

F(000) 272.0

Crystal size/mm3 0.2 × 0.1 × 0.1

RadiationCu Kα (λ = 1.54184)

2Θ range for data collection/° 7.832 to 152.96

Index ranges -9 ≤ h ≤ 9, -8 ≤ k ≤ 8, -14 ≤ l ≤ 13

Reflections collected 12513

Independent reflections 2398 [Rint = 0.0338, Rsigma = 0.0172]

Data/restraints/parameters 2398/1/177

Goodness-of-fit on F2 1.063

Final R indexes [I>=2σ (I)] R1 = 0.0283, wR2 = 0.0741

Final R indexes [all data] R1 = 0.0285, wR2 = 0.0743

Largest diff. peak/hole / e Å-3 0.24/-0.18

Flack parameter 0.00(4)

Table 2 Fractional Atomic Coordinates (×104) and Equivalent Isotropic Displacement Parameters (Å2×103) for 7. Ueq is defined as 1/3 of the trace of the orthogonalised UIJ tensor.

Atom	x	y	z	U(eq)
F15	8530.7(15)	6133(2)	-180.9(10)	27.0(3)
F17	10184.7(14)	5064.1(19)	1252.2(10)	26.4(3)
F16	9389.1(16)	8016.6(18)	1230.2(11)	28.5(3)
N9	8826(2)	5270(3)	3666.1(14)	19.5(3)
N13	5845(2)	7081(3)	1249.2(15)	20.9(3)
C18	6816(2)	3665(3)	1447.4(18)	20.8(4)
C1	8594(2)	5811(3)	4825.3(16)	18.4(4)
C6	7065(2)	6949(3)	4863.7(17)	18.1(4)
C2	9619(2)	5411(3)	5842.1(17)	21.1(4)
C7	6366(2)	7062(3)	3675.0(17)	18.7(4)
C5	6553(2)	719(3)	5950.2(17)	20.2(4)
C14	8845(3)	6248(3)	987.9(16)21.3(4)	
C8	7467(2)	6044(3)	2986.6(16)	18.2(4)
C10	7196(2)	5723(3)	1672.4(16)	19.0(4)
C3	9075(2)	6168(3)	6900.5(16)	21.3(4)
C4	7561(3)	7308(3)	6956.4(17)	20.9(4)
C19	6616(3)	1981(3)	1325.5(18)	24.2(4)

C12	4274(2)	7142(3)	1985.5(18)	23.6(4)
C11	4737(2)	8009(3)	3189.8(17)	23.6(4)

Table 3 Anisotropic Displacement Parameters (Å2×103) for 7. The Anisotropic displacement factor exponent takes the form: -2π2[h2a*2U11+2hka*b*U12+…].

Atom	U11	U22	U33	U23	U13	U12
F15	32.5(6)	32.0(7)	16.6(5)	0.0(5)	2.4(4)	0.7(5)
F17	21.0(5)	32.1(7)	26.2(6)	0.4(5)	2.7(4)	3.3(5)
F16	31.6(6)	24.1(7)	30.0(6)	-2.4(5)	5.6(5)	-8.9(5)
N9	18.4(7)	22.7(8)	17.4(7)	-1.0(6)	0.2(6)	4.8(6)
N13	23.0(8)	21.3(8)	18.3(8)	0.9(7)	-2.9(6)	2.1(6)
C18	19.7(8)	24.9(11)	17.6(8)	1.0(7)	-2.1(6)	-0.2(7)
C1	19.5(8)	16.3(9)	19.3(8)	0.3(7)	1.5(7)	-0.4(7)
C6	17.3(8)	15.9(9)	21.2(9)	1.0(7)	1.8(7)	-1.2(7)
C2	21.5(8)	20.1(10)	21.6(9)	0.7(7)	-1.5(7)	1.4(7)
C7	17.9(8)	16.7(8)	21.5(9)	0.8(7)	1.2(7)	-0.2(7)
C5	19.7(8)	18.8(9)	22.1(9)	-0.9(7)	2.5(7)	-0.1(7)
C14	23.8(9)	20.8(10)	19.2(8)	-0.2(8)	1.2(6)	0.0(8)
C8	17.8(8)	17.3(9)	19.5(8)	1.2(7)	-1.0(6)	0.3(7)
C10	18.6(8)	19.5(10)	18.8(9)	1.3(7)	-0.5(6)	0.5(7)
C3	24.0(8)	19.8(10)	19.9(8)	1.8(7)	-1.8(7)	-2.9(8)
C4	23.1(9)	20.4(10)	19.5(9)	-1.7(7)	4.2(7)	-2.5(7)
C19	26.1(10)	20.2(11)	26.0(10)	0.1(8)	-3.2(8)	-0.5(8)
C12	18.5(9)	24.8(10)	27.3(10)	2.8(8)	-2.6(7)	1.3(8)
C11	20.8(8)	26.0(11)	24.1(9)	1.4(8)	-1.0(7)	5.5(8)

Table 4 Bond Lengths for 7.

Atom	Atom	Length/Å	Atom	Atom	Length/Å
F15	C14	1.337(2)	C6	C7	1.433(3)
F17	C14	1.343(2)	C6	C5	1.405(3)
F16	C14	1.329(2)	C2	C3	1.381(3)
N9	C1	1.378(2)	C7	C8	1.361(3)
N9	C8	1.384(2)	C7	C11	1.500(3)
N13	C10	1.472(2)	C5	C4	1.385(3)
N13	C12	1.479(2)	C14	C10	1.541(2)

C18	C10	1.487(3)	C8	C10	1.509(2)
C18	C19	1.194(3)	C3	C4	1.407(3)
C1	C6	1.415(2)	C12	C11	1.521(3)
C1	C2	1.400(3)			

Table 5 Bond Angles for 7.

Atom	Atom	Atom	Angle/°	Atom	Atom	Atom	Angle/°
C1	N9	C8	107.77(15)	F16	C14	F15	107.82(16)
C10	N13	C12	114.12(15)	F16	C14	F17	107.16(16)
C19	C18	C10	175.0(2)	F16	C14	C10	111.93(16)
N9	C1	C6	108.12(16)	N9	C8	C10	124.57(16)
N9	C1	C2	129.82(17)	C7	C8	N9	110.70(16)
C2	C1	C6	122.05(17)	C7	C8	C10	124.66(16)
C1	C6	C7	106.80(16)	N13	C10	C18	115.85(15)
C5	C6	C1	119.45(17)	N13	C10	C14	105.02(15)
C5	C6	C7	133.75(18)	N13	C10	C8	107.42(15)
C3	C2	C1	117.28(18)	C18	C10	C14	107.65(16)
C6	C7	C11	130.58(17)	C18	C10	C8	109.43(16)
C8	C7	C6	106.61(16)	C8	C10	C14	111.46(14)
C8	C7	C11	122.79(17)	C2	C3	C4	121.52(18)
C4	C5	C6	118.40(18)	C5	C4	C3	121.29(18)
F15	C14	F17	107.29(15)	N13	C12	C11	109.99(15)
F15	C14	C10	110.69(15)	C7	C11	C12	108.70(17)
F17	C14	C10	111.73(15)				

Table 6 Torsion Angles for 7.

A	B	C	D	Angle/°
F15	C14	C10	N13	-58.0(2)
C6	C7	C8	N9	0.5(2)
F15	C14	C10	C18	66.0(2)
C6	C7	C8	C10	177.73(17)
F15	C14	C10	C8	-174.04(17)
C6	C7	C11	C12	-160.5(2)
F17	C14	C10	N13	-177.53(15)
C6	C5	C4	C3	0.8(3)

F17	C14	C10	C18	-53.5(2)
C2	C1	C6	C7	179.73(17)
F17	C14	C10	C8	66.5(2)
C2	C1	C6	C5	0.5(3)
F16	C14	C10	N13	62.28(18)
C2	C3	C4	C5	-0.1(3)
F16	C14	C10	C18	-173.73(16)
C7	C6	C5	C4	-180.0(2)
F16	C14	C10	C8	-53.7(2)
C7	C8	C10	N13	14.4(2)
N9	C1	C6	C7	0.5(2)
C7	C8	C10	C18	-112.2(2)
N9	C1	C6	C5	-178.74(17)
C7	C8	C10	C14	128.9(2)
N9	C1	C2	C3	179.26(19)
C5	C6	C7	C8	178.5(2)
N9	C8	C10	N13	-168.77(17)
C5	C6	C7	C11	-3.4(4)
N9	C8	C10	C18	64.7(2)
C8	N9	C1	C6	-0.2(2)
N9	C8	C10	C14	-54.2(2)
C8	N9	C1	C2	-179.35(19)
N13	C12	C11	C7	-48.1(2)
C8	C7	C11	C12	17.4(3)
C1	N9	C8	C7	-0.2(2)
C10	N13	C12	C11	68.3(2)
C1	N9	C8	C10	-177.41(17)
C12	N13	C10	C18	75.1(2)
C1	C6	C7	C8	-0.6(2)
C12	N13	C10	C14	-166.32(16)
C1	C6	C7	C11	177.5(2)
C12	N13	C10	C8	-47.6(2)
C1	C6	C5	C4	-1.0(3)
C11	C7	C8	N9	-177.82(18)
C1	C2	C3	C4	-0.5(3)

C11	C7	C8	C10	-0.6(3)
C6	C1	C2	C3	0.2(3)

Table 7 Hydrogen Atom Coordinates (Å×104) and Isotropic Displacement Parameters (Å2×103) for 7.

Atom	x	y	z	U(eq)
H9	9685.24	4559.85	3404.83	23
H2	10646.03	4649.14	5805.79	25
H5	5540.53	8502.13	5993.56	24
H3	9738.38	5913.88	7606.87	26
H4	7222.86	7806.12	7699.06	25
H19	6456.09	641.33	1228.5	29
H12A	3814.27	5830.2	2090.26	28
H12B	3351.83	7919.02	1583.37	28
H11A	4934.13	9402.97	3108.58	28
H11B	3761.85	7810.76	3736.36	28
H13	5570(30)	6750(30)	510(20)	15(5)

8. Optimization of Pictet-Spengler reaction of ketone 2c[a]

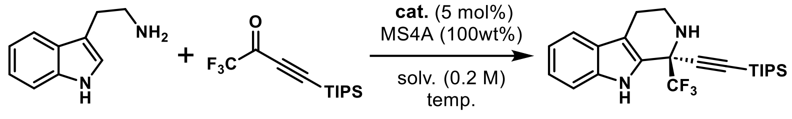

1 2c 4

Table S1.

Entry	Catalyst	Temp. / °C	Solvent	Time / h	Yield / %[b]	Ee / %
1	(*R*)-**3a**	40	DCM	40	92	81
2	(*R*)-**3b**	40	DCM	45	92	91
3	(*R*)-**3c**	40	DCM	72	85	34
4	(*R*)-**3d**	40	DCM	138	84	74
5	(*S*)-**3e**	40	DCM	40	90	42
6	(*S*)-**3f**	40	DCM	96	8	Rac
7	(*S*)-**3g**	40	DCM	40	92	41
8	(*R*)-**3h**	40	DCM	45	90	-58
9	(*S*)-**3i**	40	DCM	35	90	43
10	(*S*)-**3j**	40	DCM	120	81	31

11	(*S*)-**3k**	40	DCM	96	84	-77
12	(*S*)-**3l**	40	DCM	166	75	-19
13[c]	(*R*)-**3b**	40	DCM	45	91	90
14	(*R*)-**3b**	60	DCM	24	90	85
15	(*R*)-**3b**	25	DCM	65	89	91
16	(*R*)-**3b**	40	$CHCl_3$	120	37	80
17	(*R*)-**3b**	40	1,2-DCE	98	76	85
18	(*R*)-**3b**	40	Toluene	120	97	79
19	(*R*)-**3b**	40	MeCN	92	66	84
20	(*R*)-**3b**	40	THF	172	9	72

[a] Unless otherwise specified, all reactions were carried out using 0.1 mmol of **1,** 0.12 mmol (1.2 eq.) of **2** and 0.0005 mmol (5 mol%) of catalyst. [b] Isolated yield. [c] Reaction without MS4A. TRIP = 2,4,6-^{i}Pr$_3$-C$_6$H$_2$.

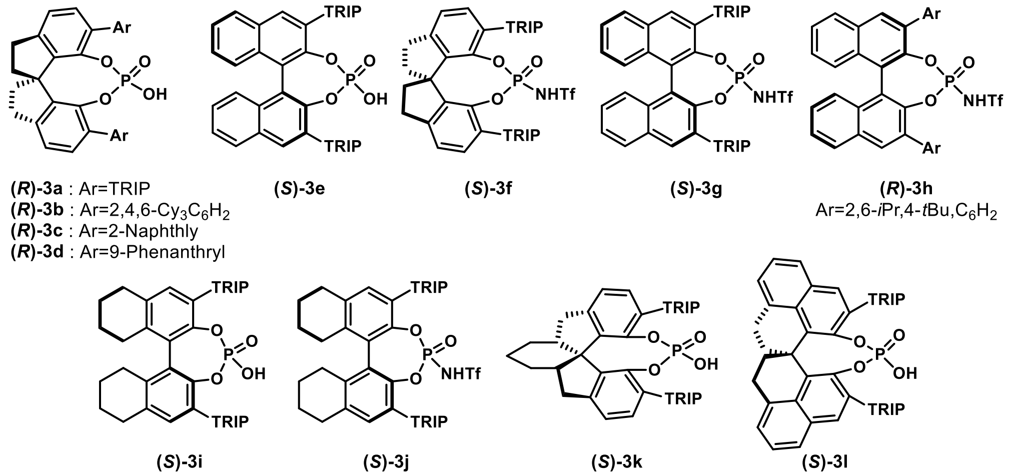

Substrate scope of ketones (CF$_2$Br group)

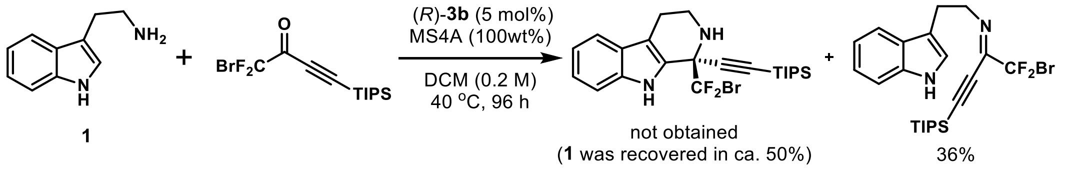

^{1}H NMR (600 MHz, $CDCl_3$), ^{13}C NMR (151 MHz $CDCl_3$) and ^{19}F NMR (565 MHz $CDCl_3$) spectra of **S3g**

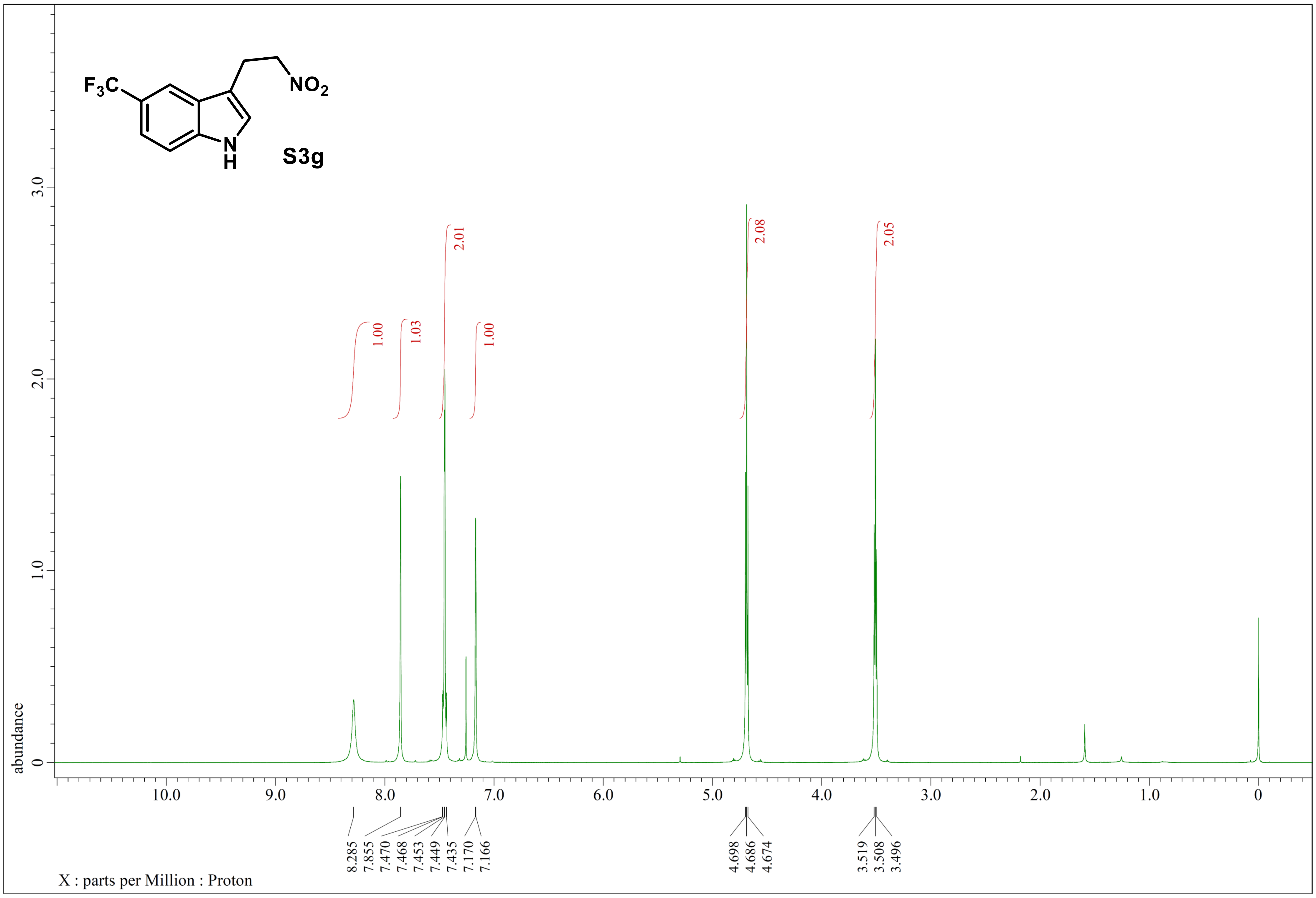

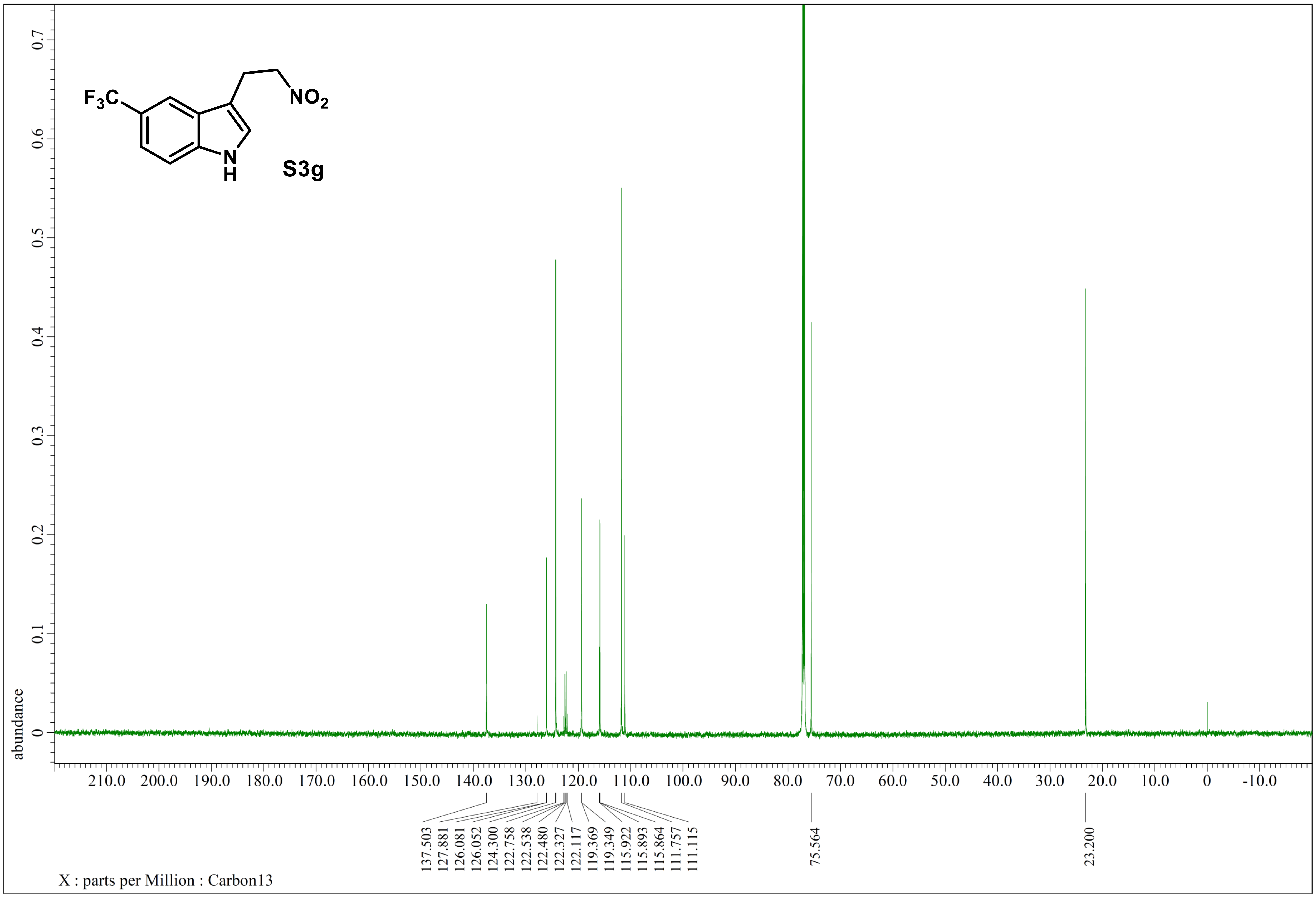

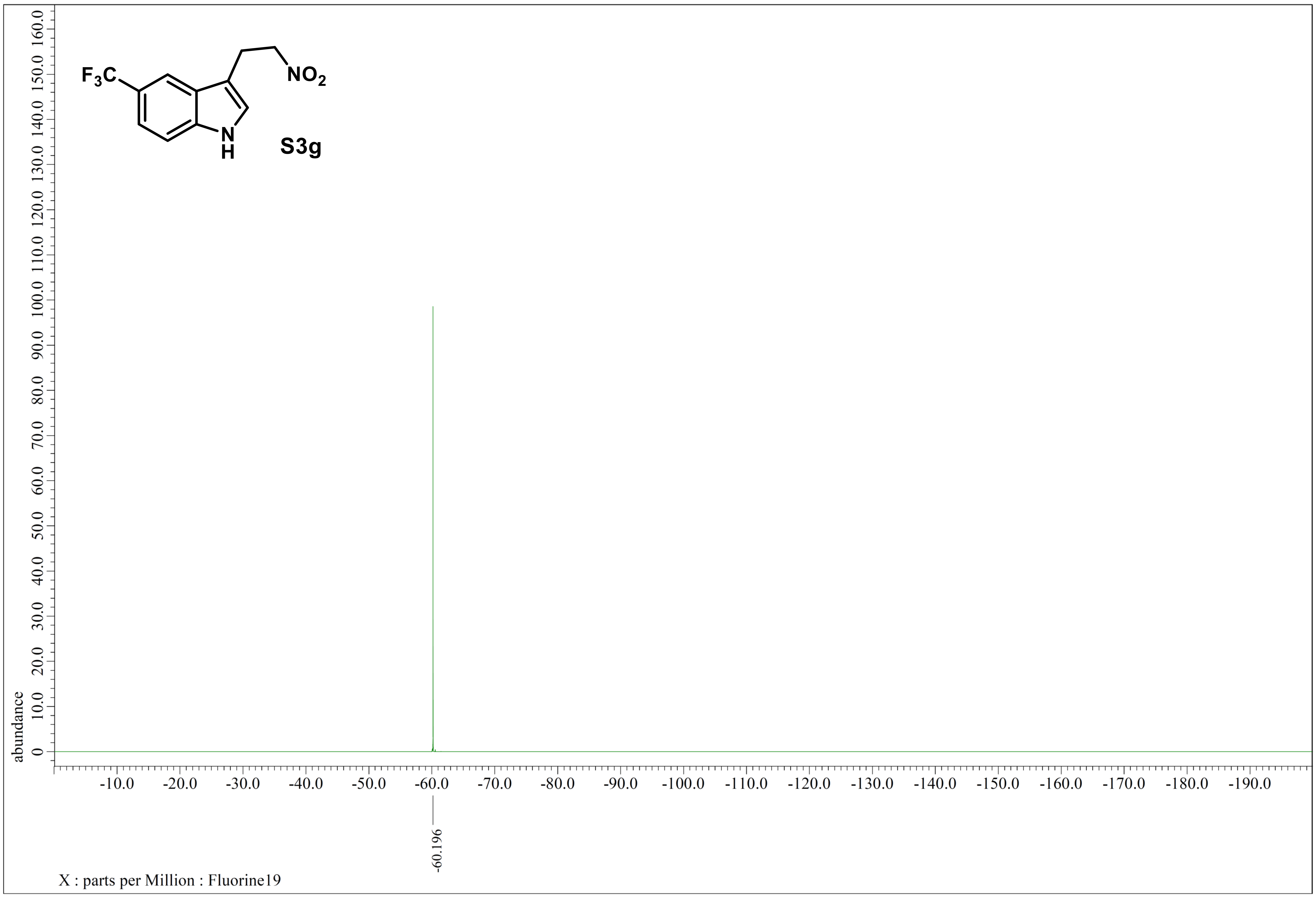

^{1}H NMR (600 MHz, $CDCl_3$) and ^{13}C NMR (151 MHz $CDCl_3$) spectra of **S3j**

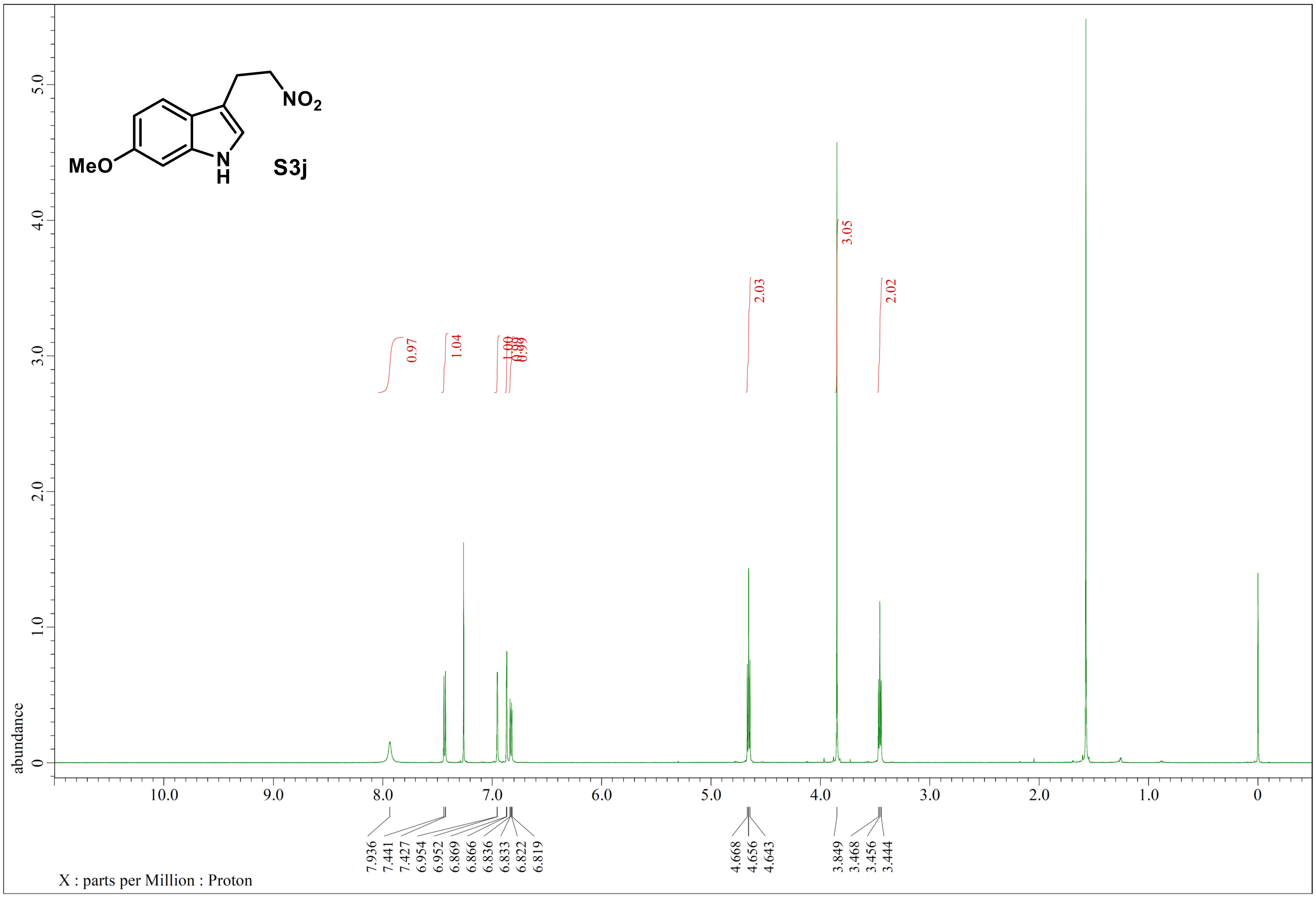

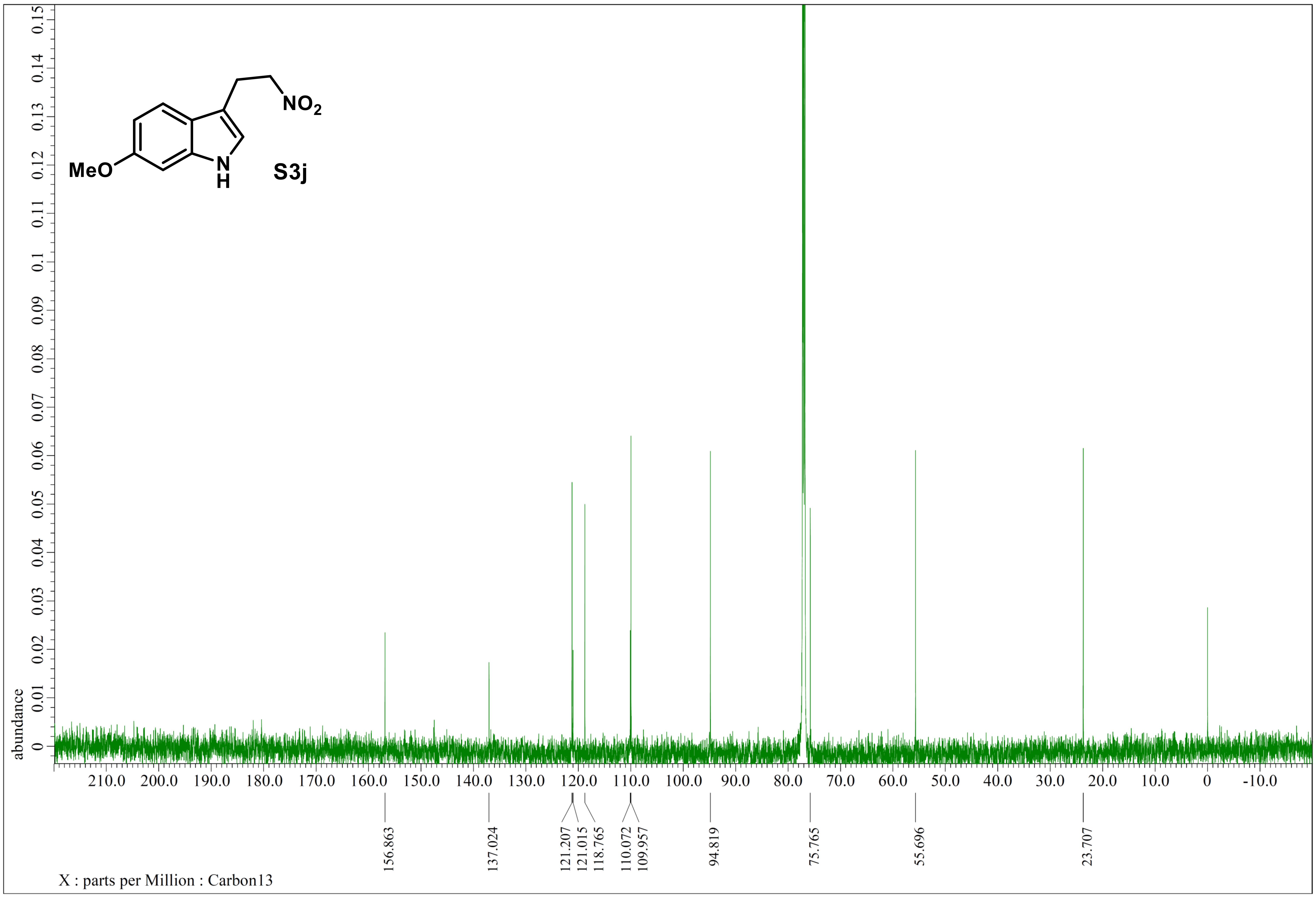

^{1}H NMR (600 MHz, $CDCl_3$), ^{13}C NMR (151 MHz $CDCl_3$) and ^{19}F NMR (565 MHz $CDCl_3$) spectra of **S3l**

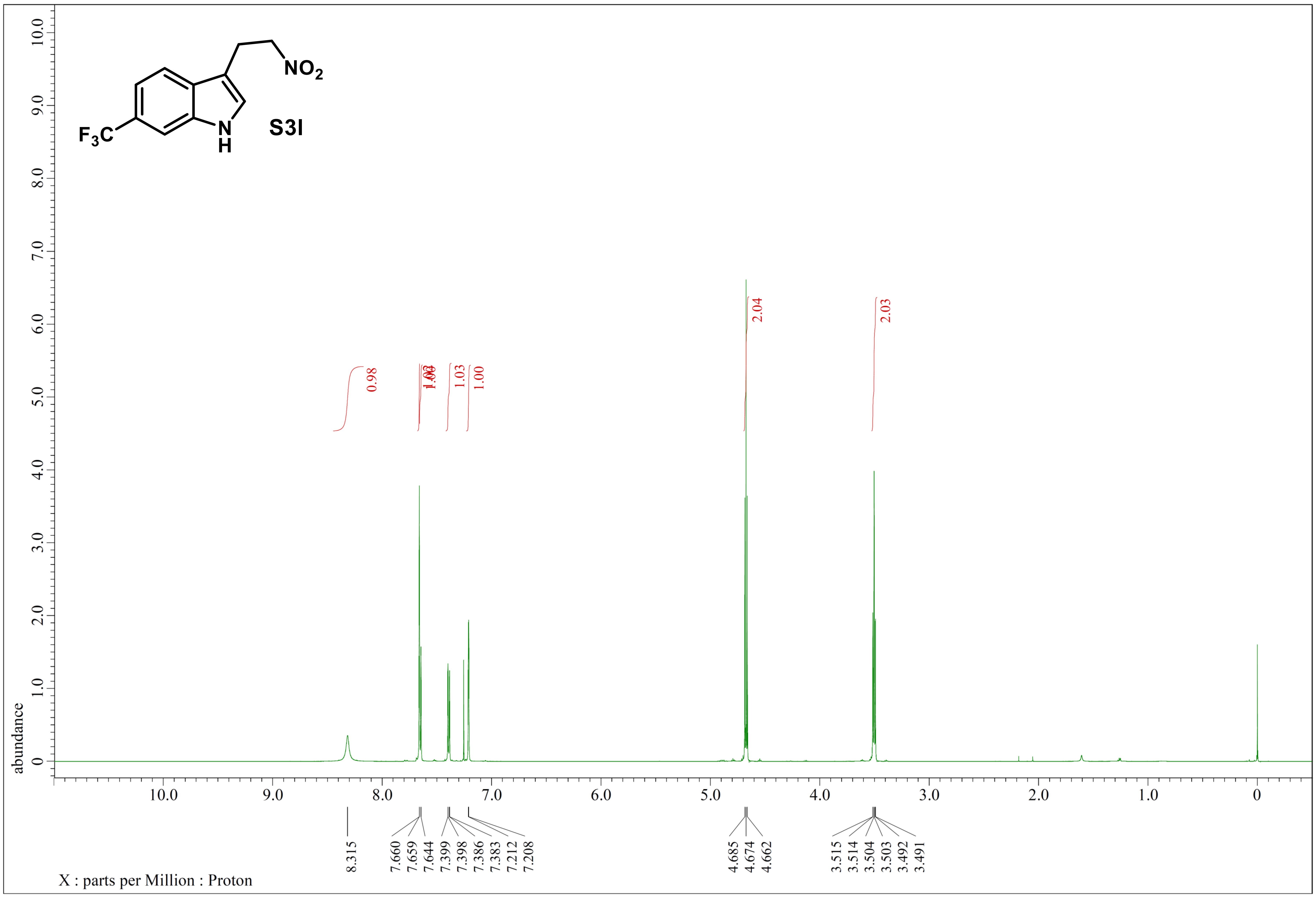

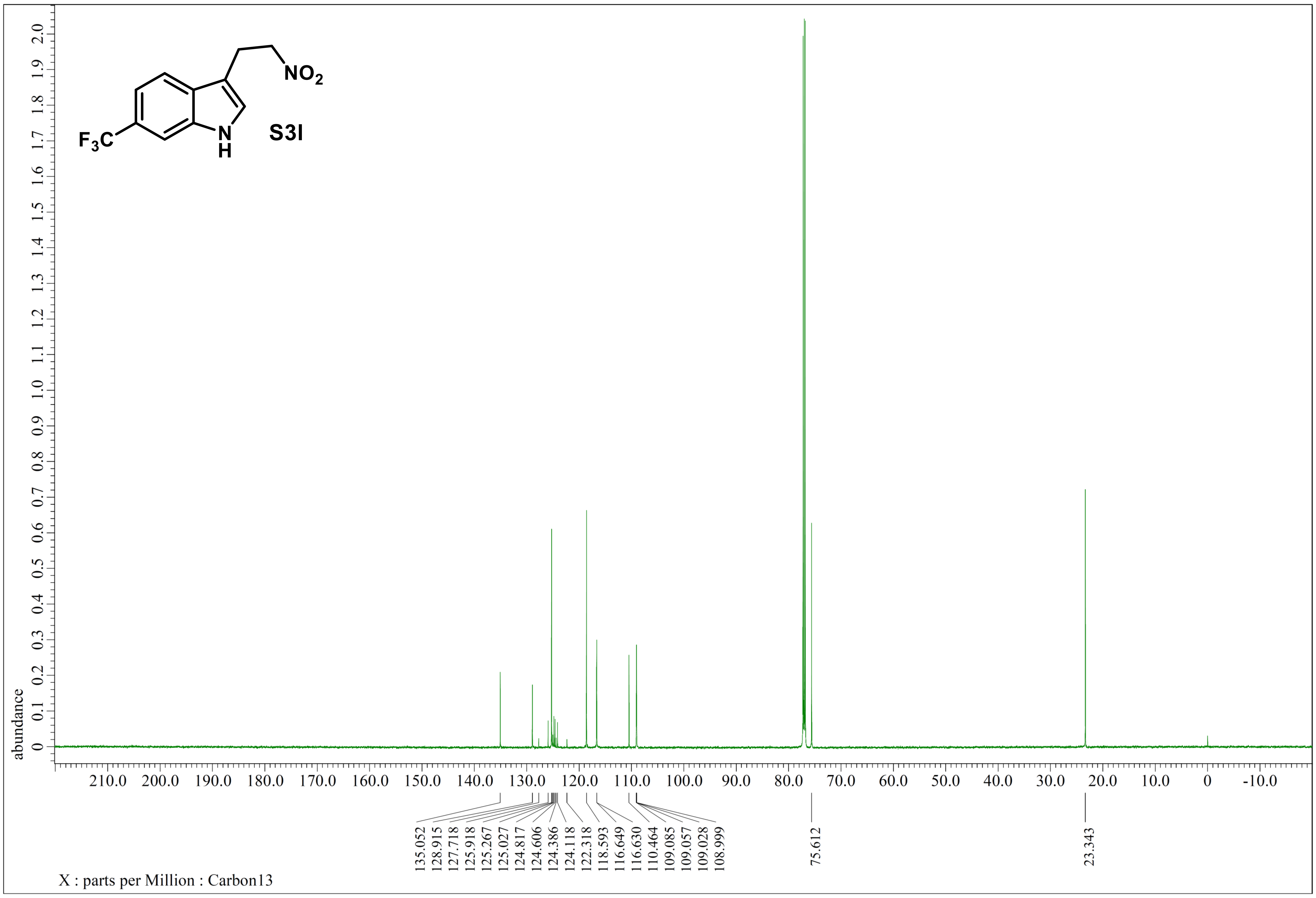
NO2
F3C
N
H
S3l
abundance
210.0 200.0 190.0 180.0 170.0 160.0 150.0 140.0 130.0 120.0 110.0 100.0 90.0 80.0 70.0 60.0 50.0 40.0 30.0 20.0 10.0 0 -10.0
135.052
128.915
127.718
125.918
125.267
125.027
124.817
124.606
124.386
124.118
122.318
118.593
116.649
116.630
110.464
109.085
109.057
109.028
108.999
75.612
23.343
X : parts per Million : Carbon13

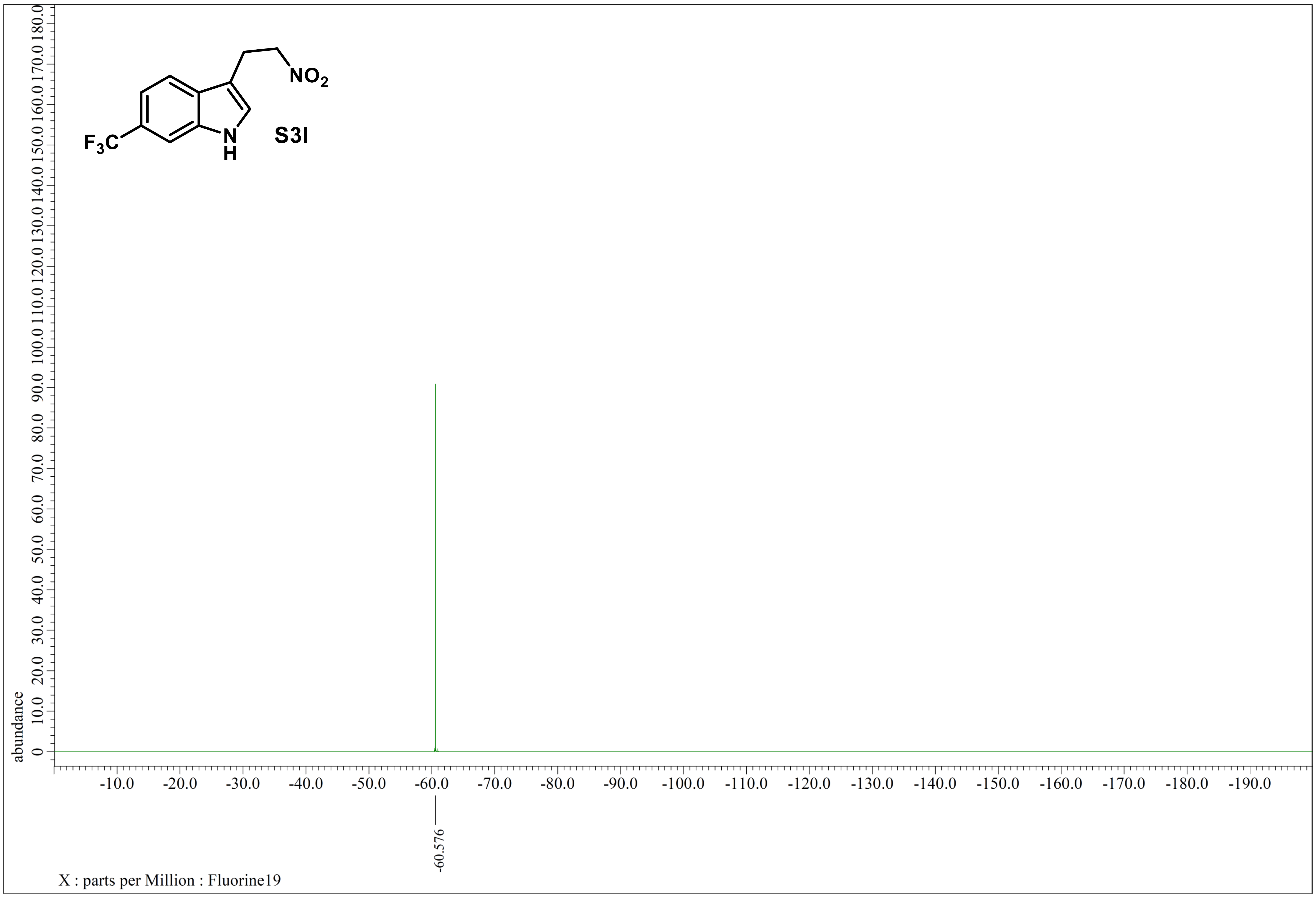
NO2
F3C
N
H
S3l
abundance
-10.0 -20.0 -30.0 -40.0 -50.0 -60.0 -70.0 -80.0 -90.0 -100.0 -110.0 -120.0 -130.0 -140.0 -150.0 -160.0 -170.0 -180.0 -190.0
-60.576
X : parts per Million : Fluorine19

^{1}H NMR (600 MHz, $CDCl_3$) and ^{13}C NMR (151 MHz $CDCl_3$) spectra of **S3n**

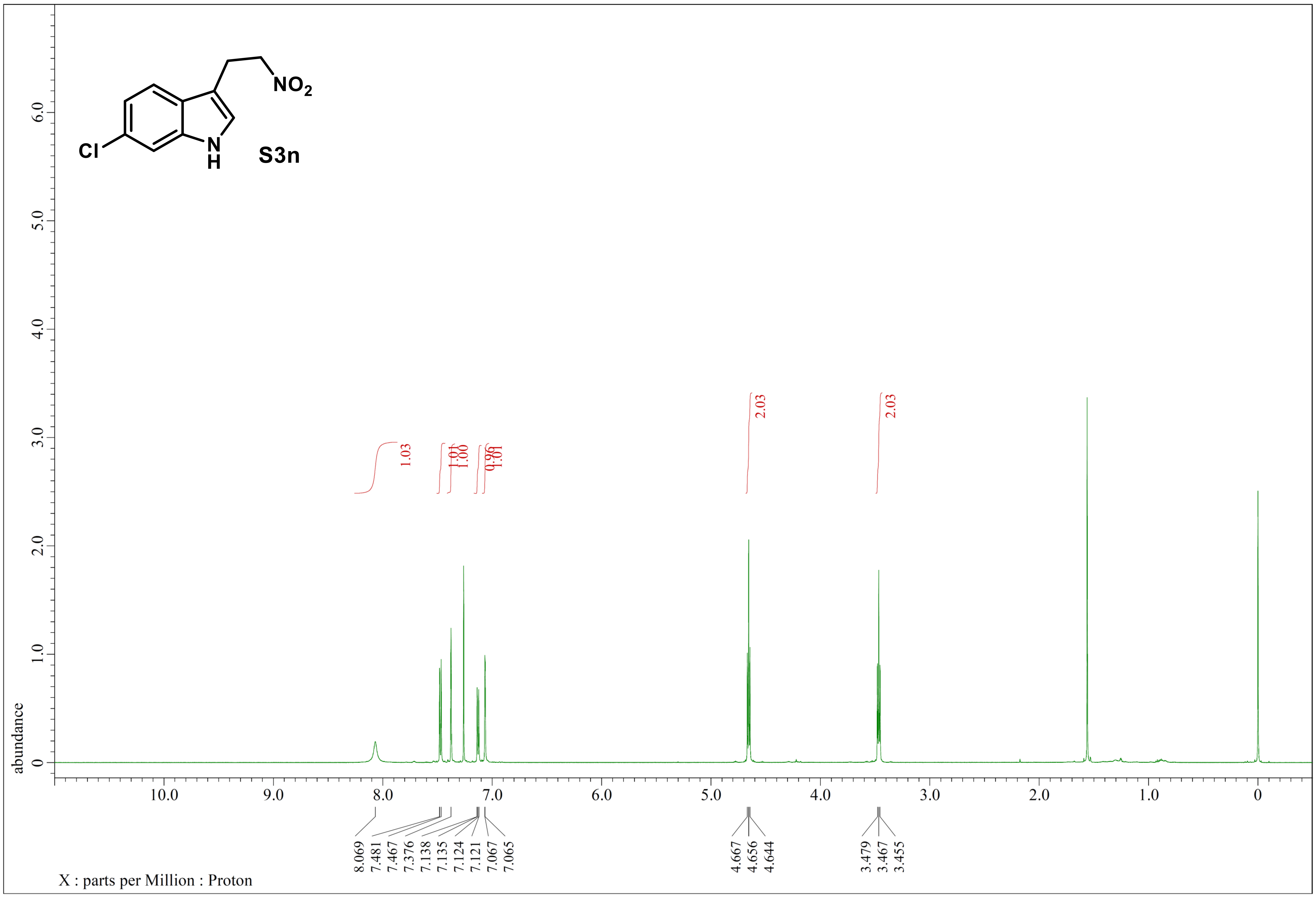

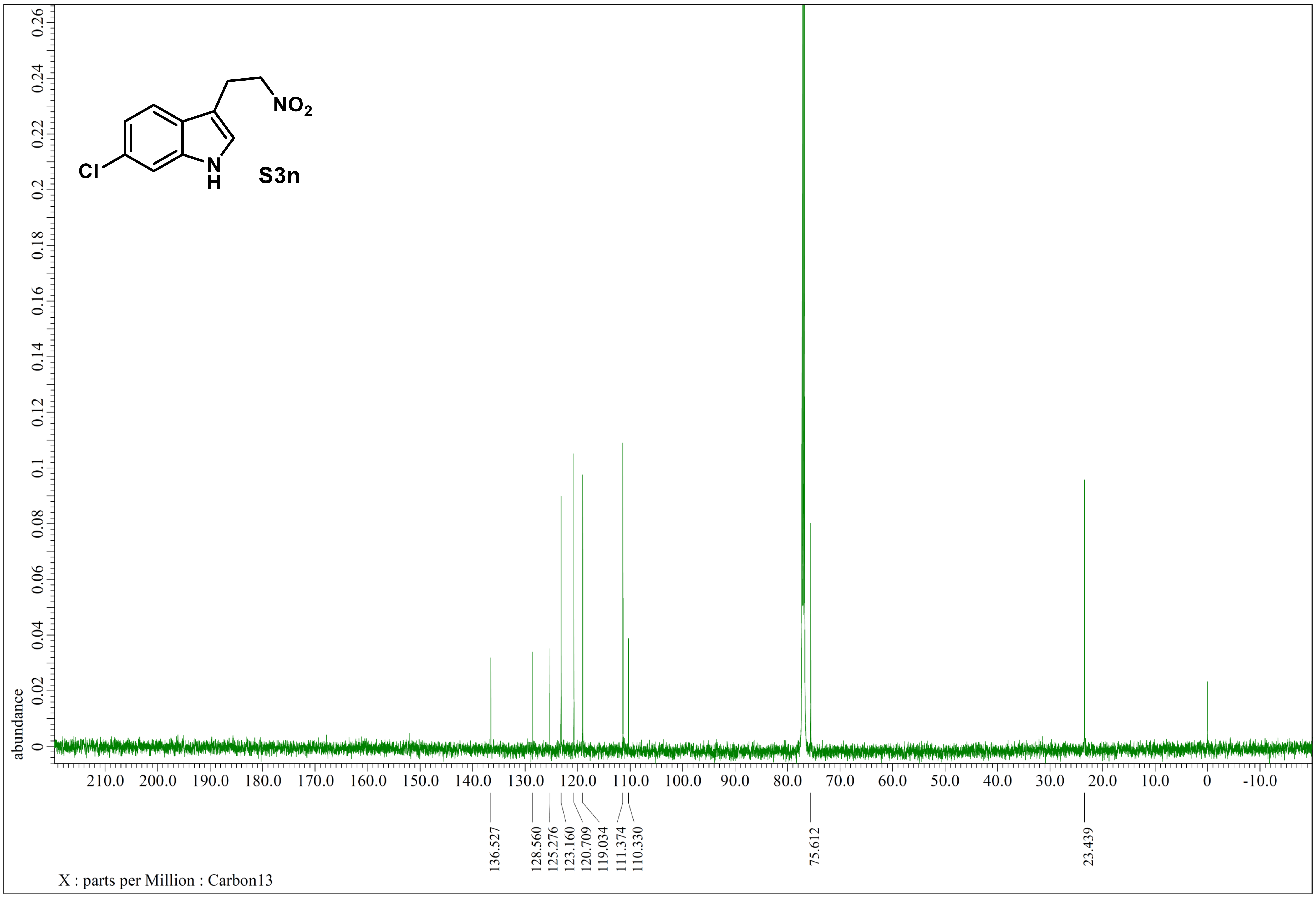

^{1}H NMR (600 MHz, CD_3OD) and ^{13}C NMR (151 MHz CD_3OD) spectra of **1f**

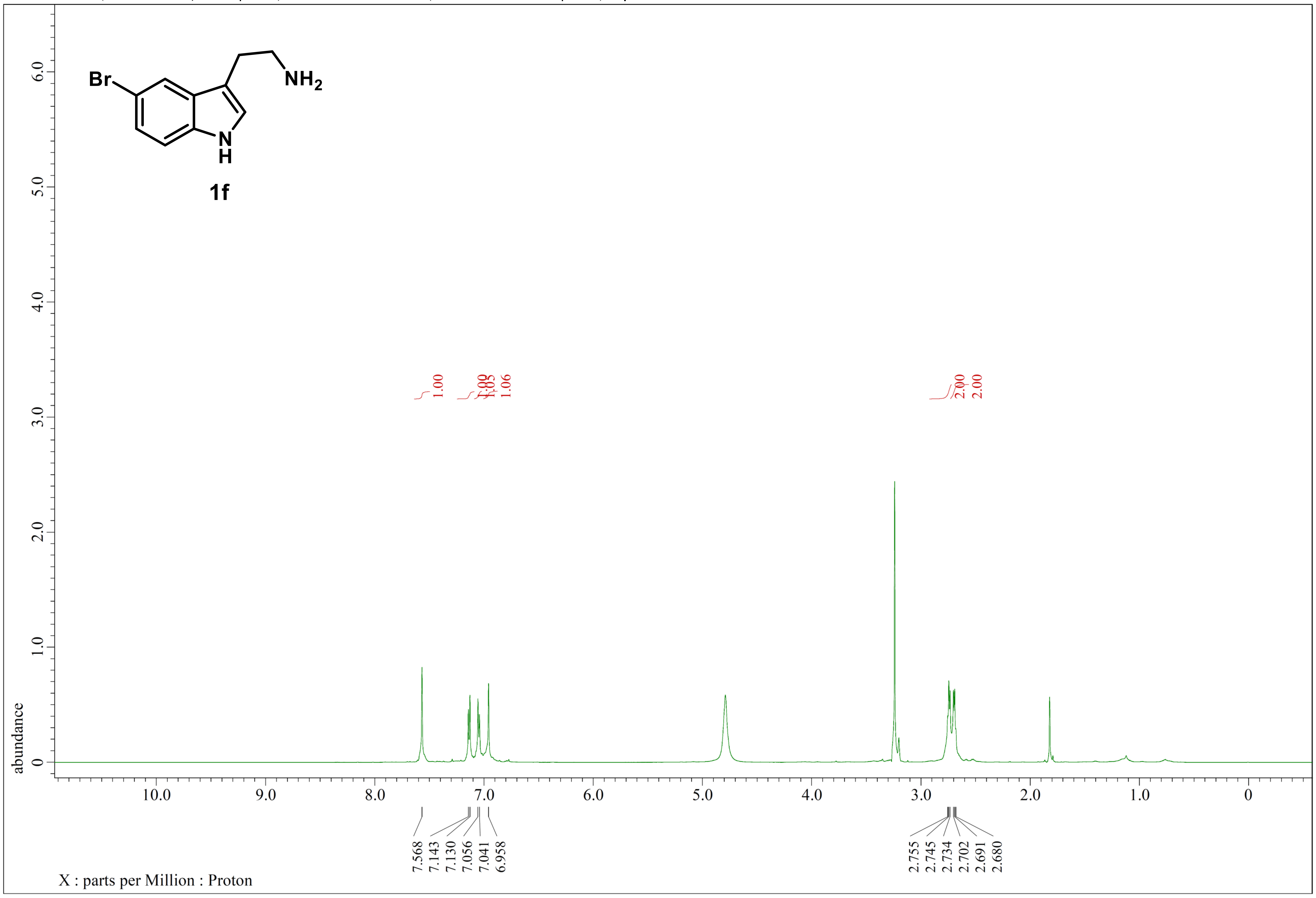

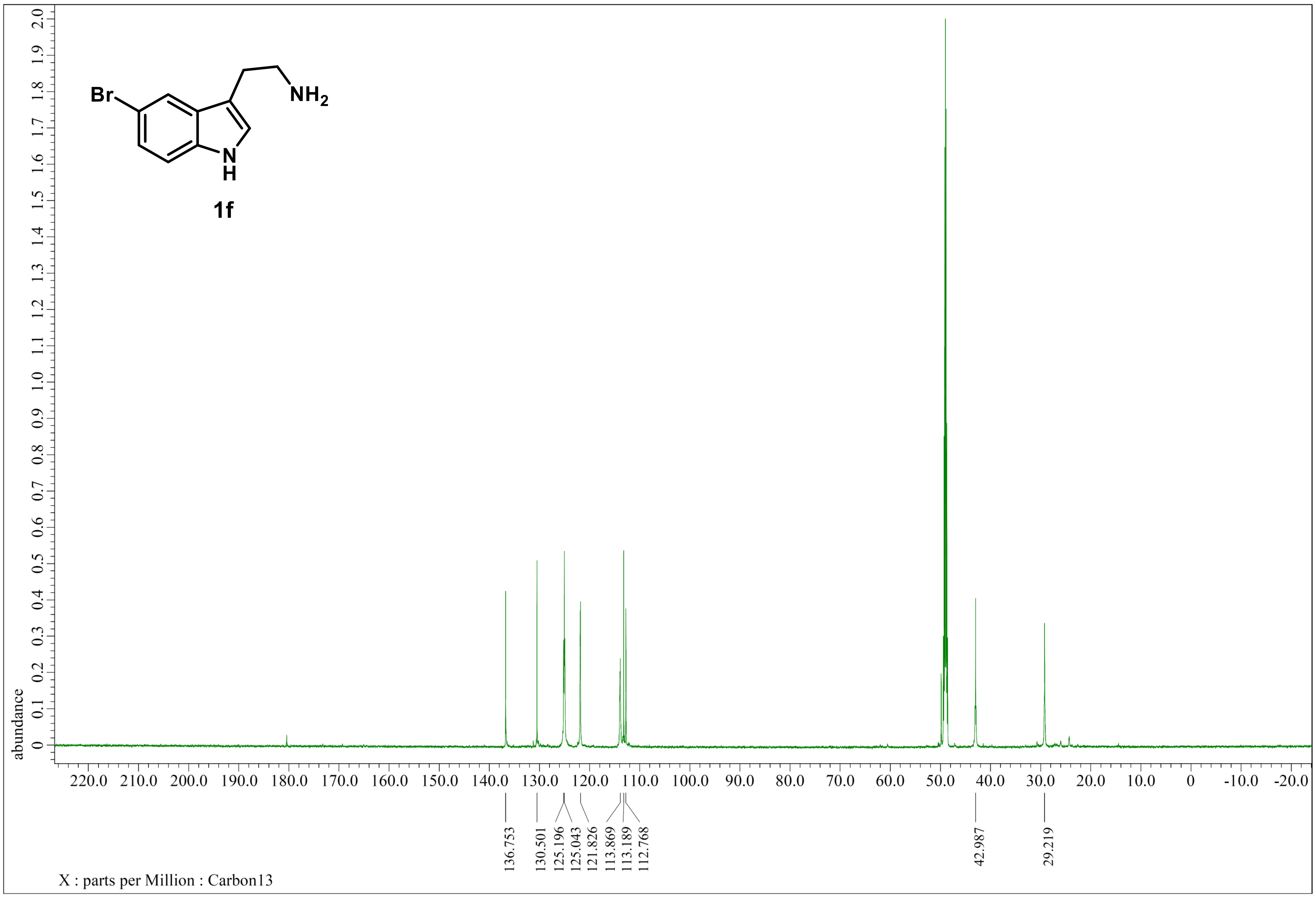

^{1}H NMR (600 MHz, CD_3OD), ^{13}C NMR (151 MHz CD_3OD) and ^{19}F NMR (565 MHz CD_3OD) spectra of **1g**

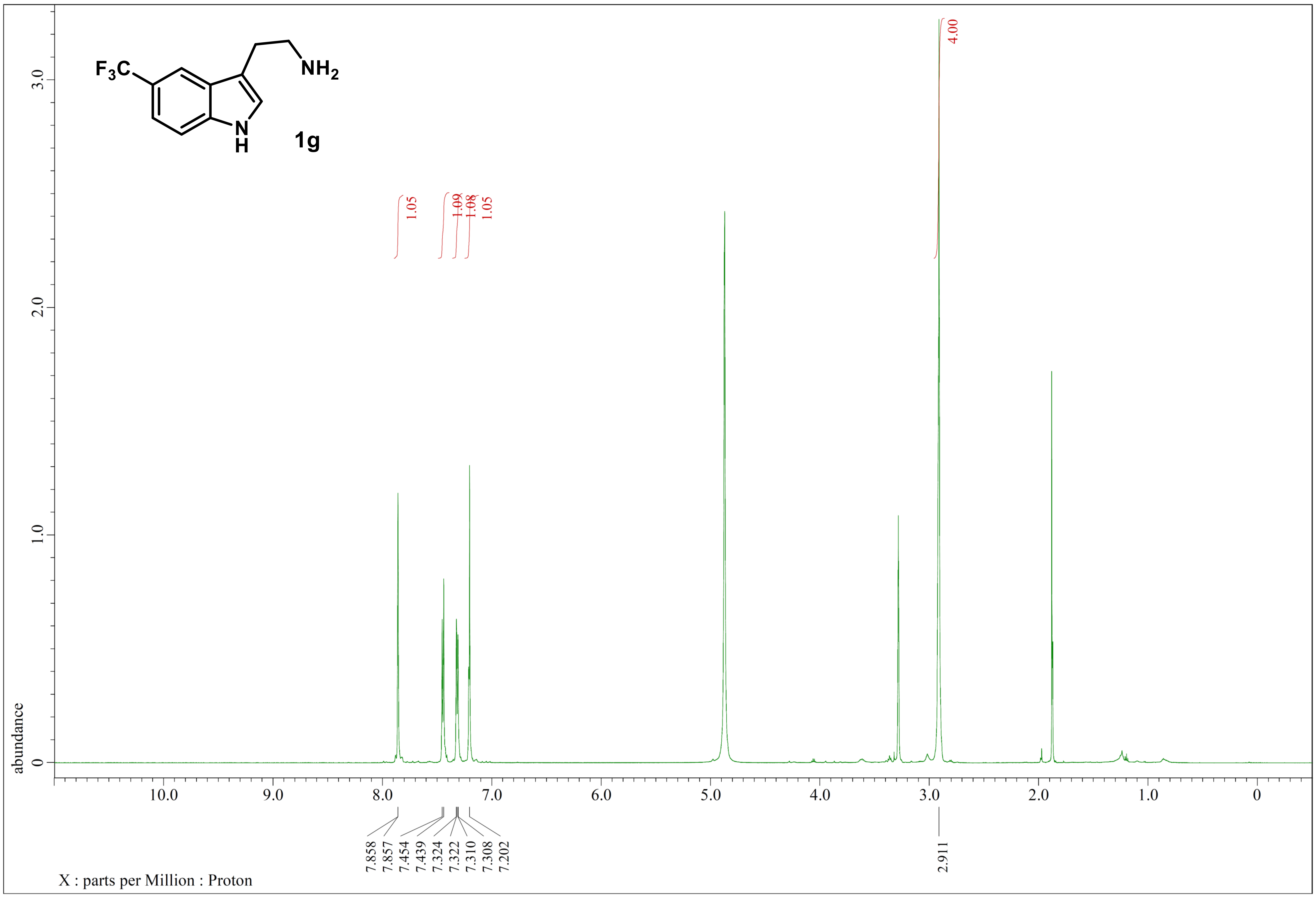

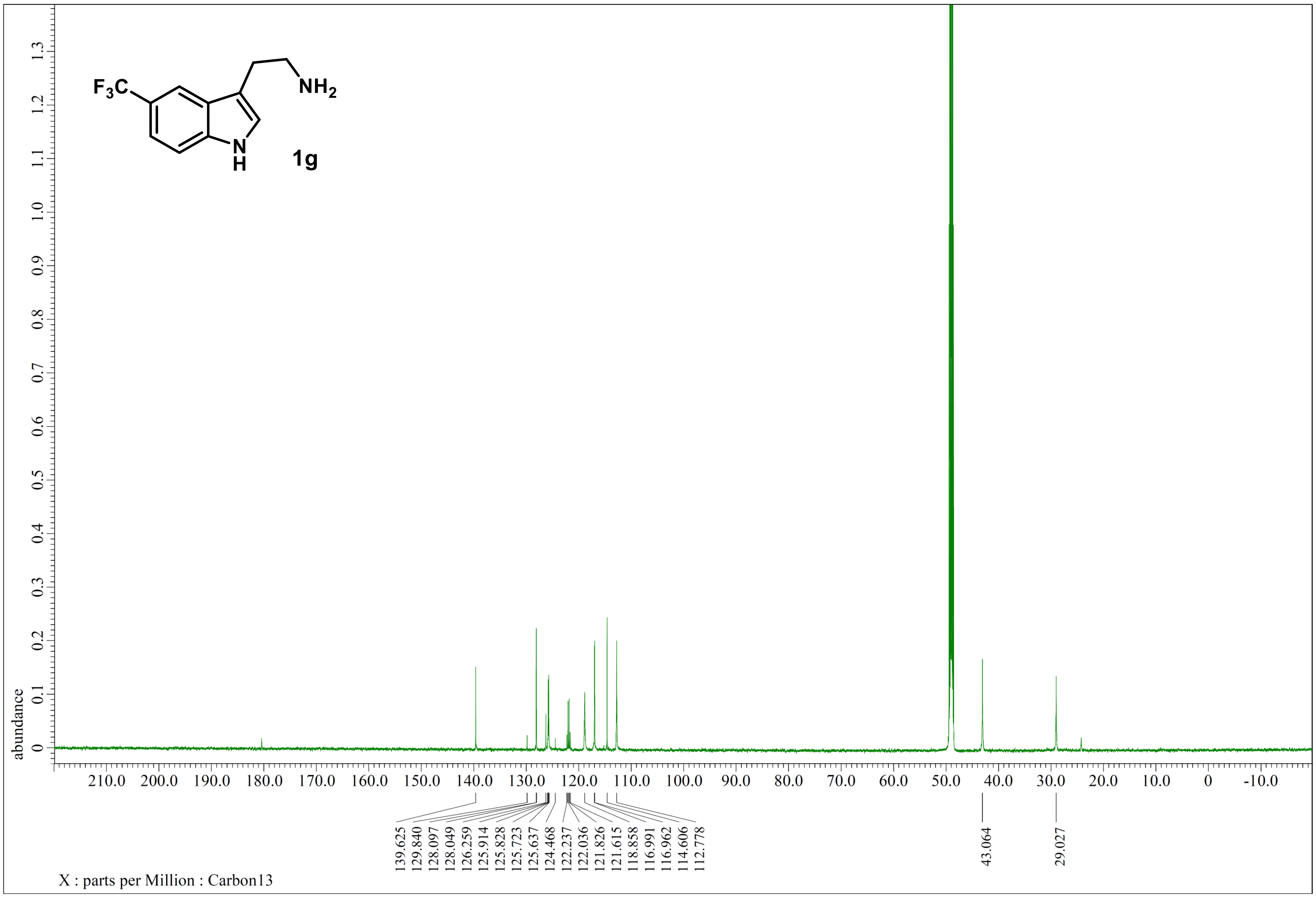

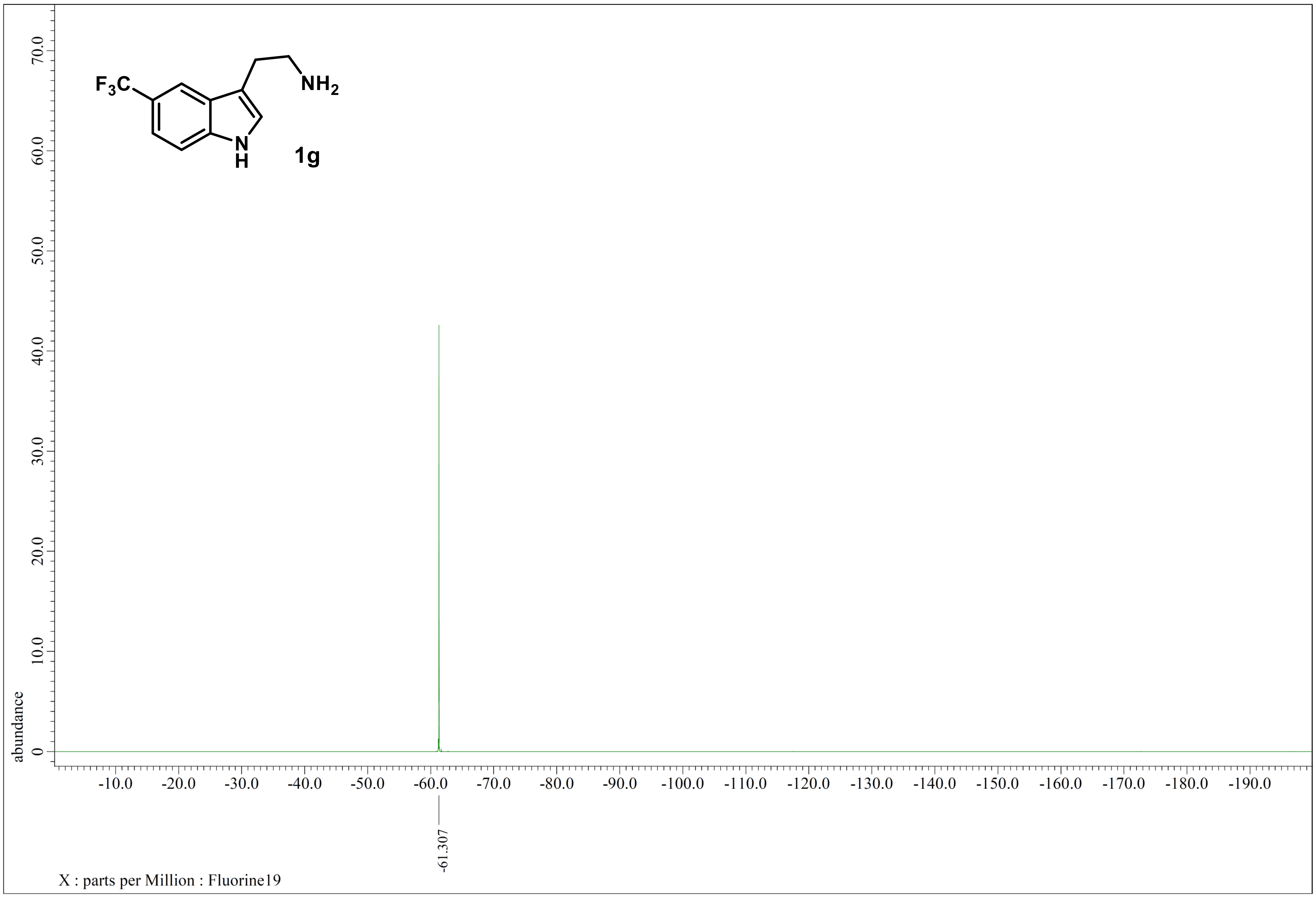

^{1}H NMR (600 MHz, CD_3OD) and ^{13}C NMR (151 MHz CD_3OD) spectra of **1h**

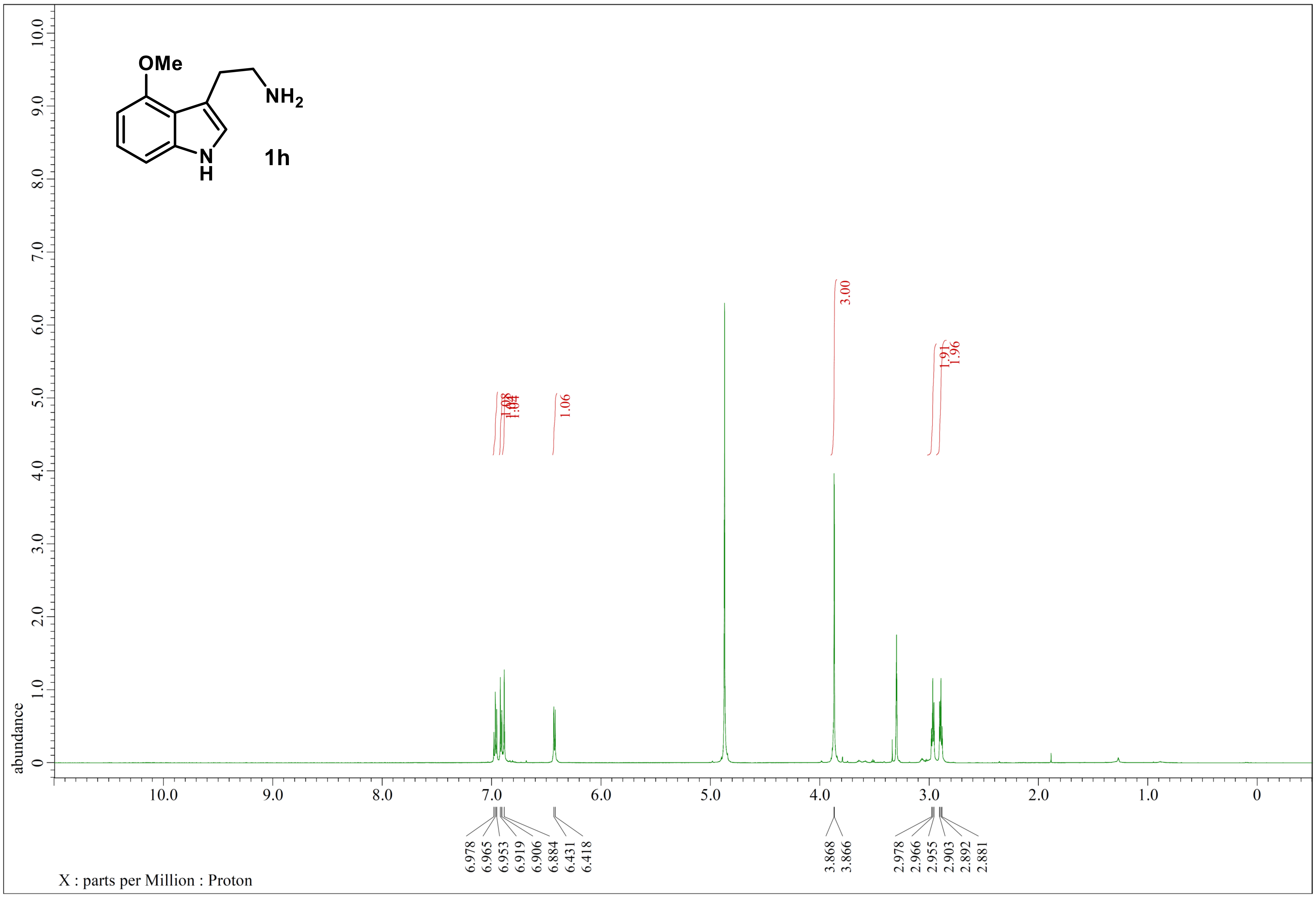

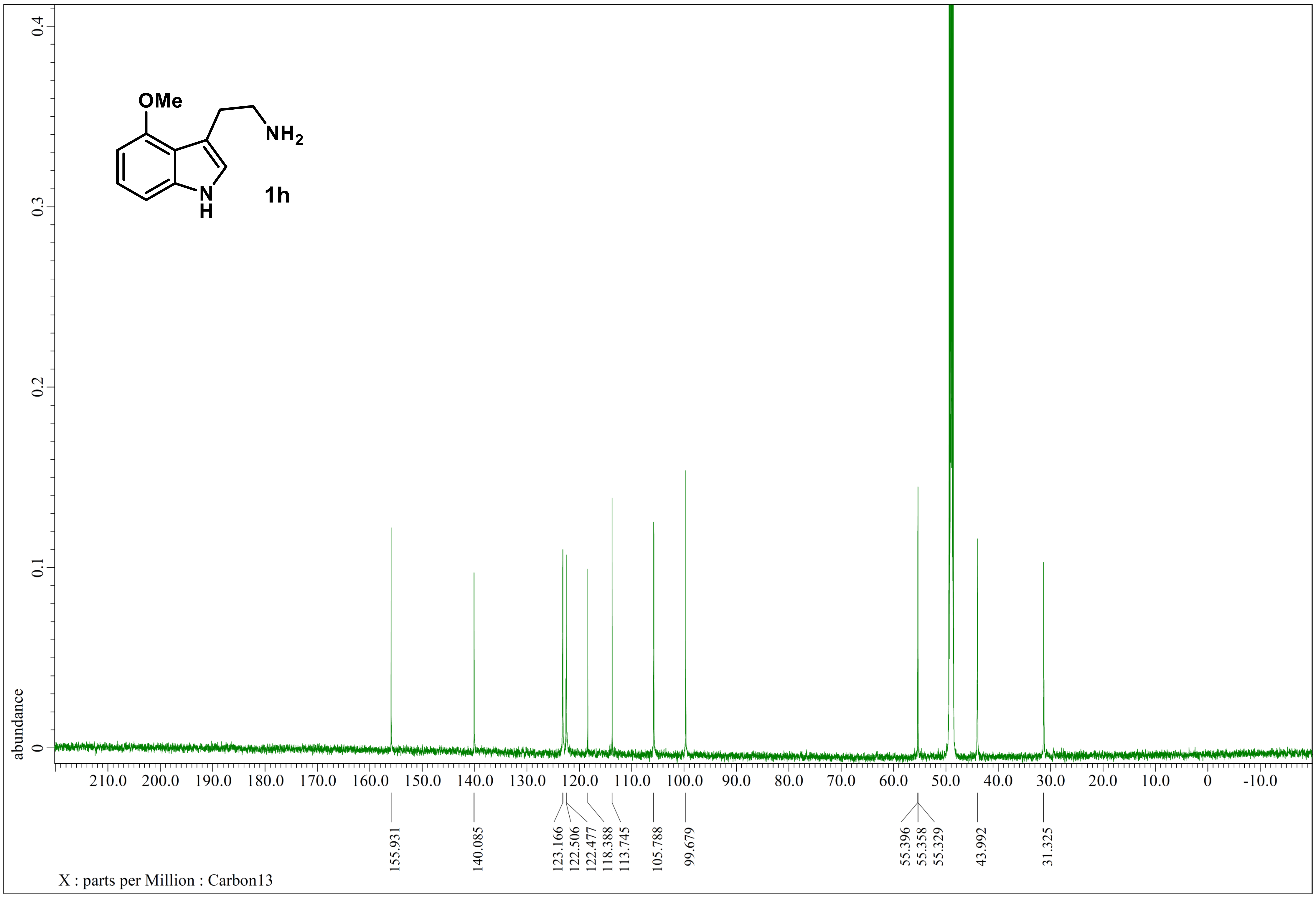

^{1}H NMR (600 MHz, CD_3OD) and ^{13}C NMR (151 MHz CD_3OD) spectra of **1i**

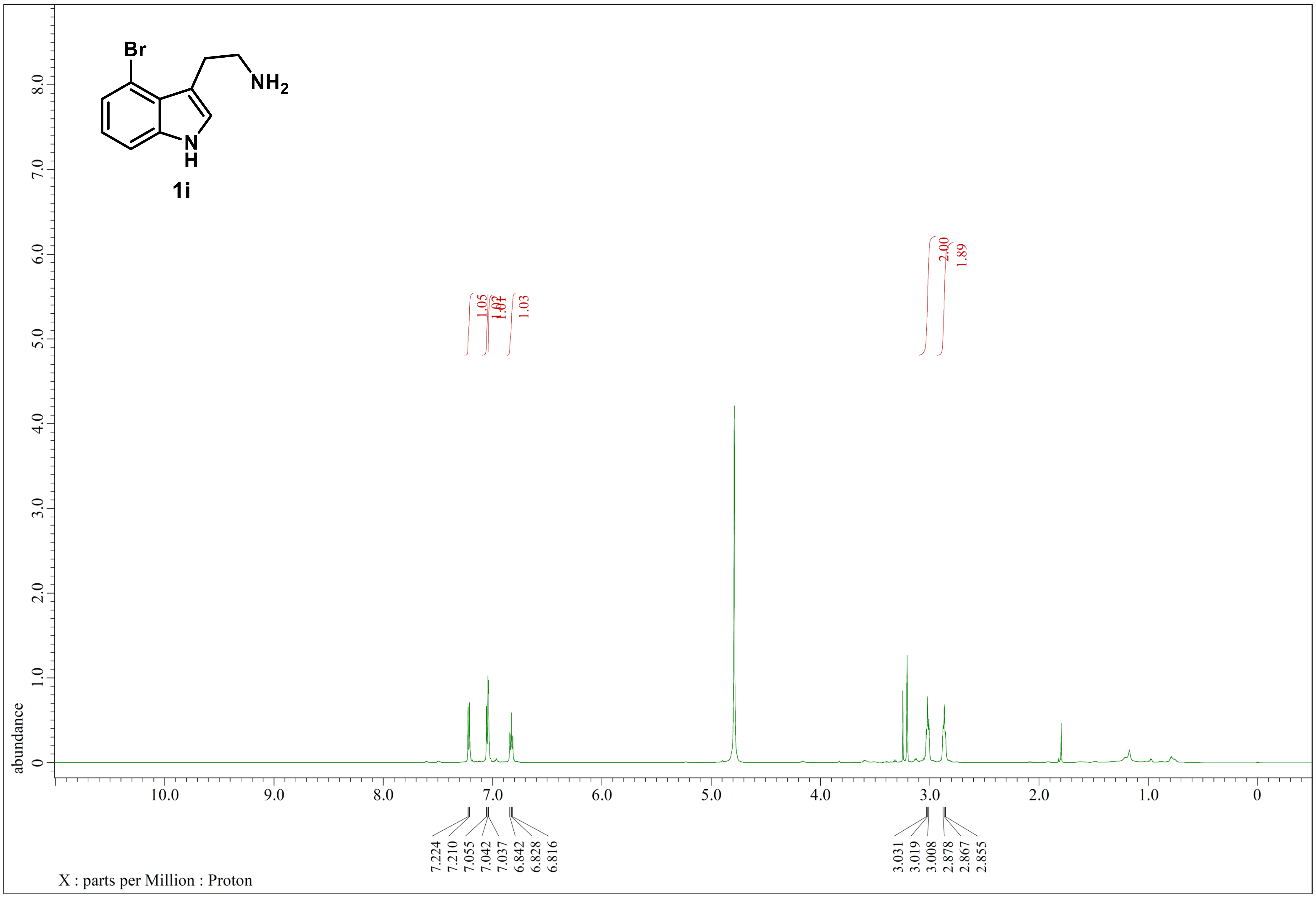

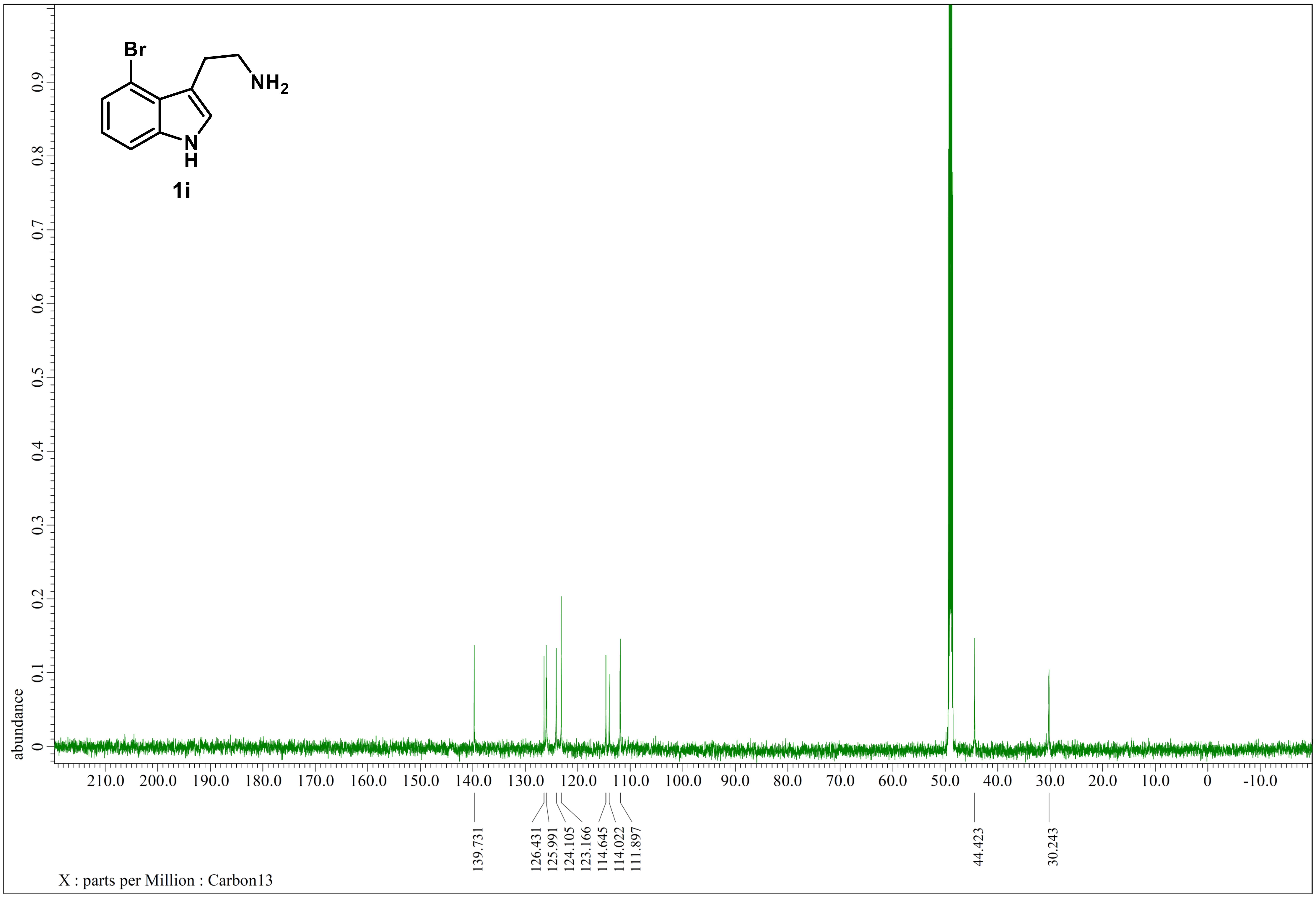

^{1}H NMR (600 MHz, CD_3OD) and ^{13}C NMR (151 MHz CD_3OD) spectra of **1j**

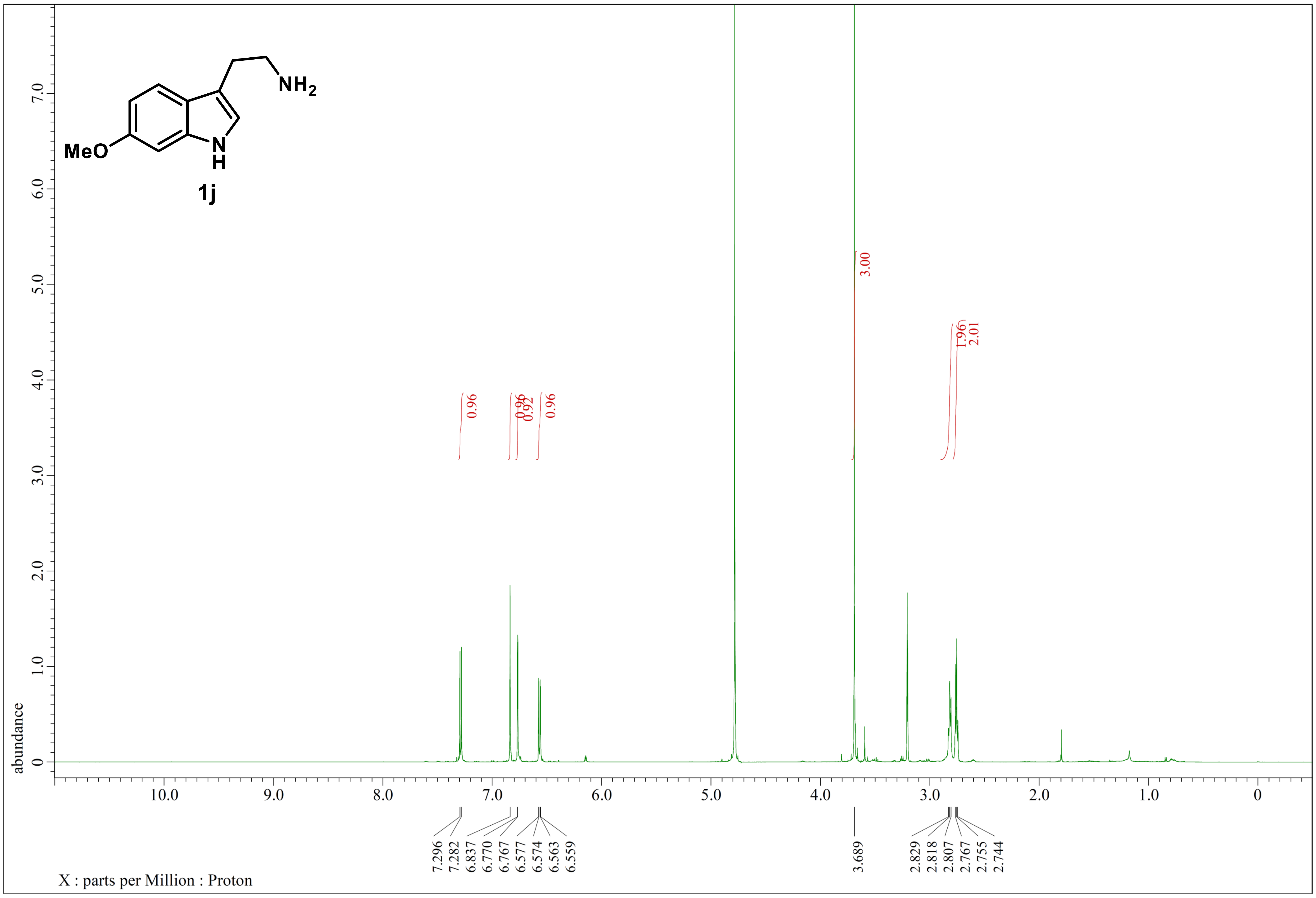

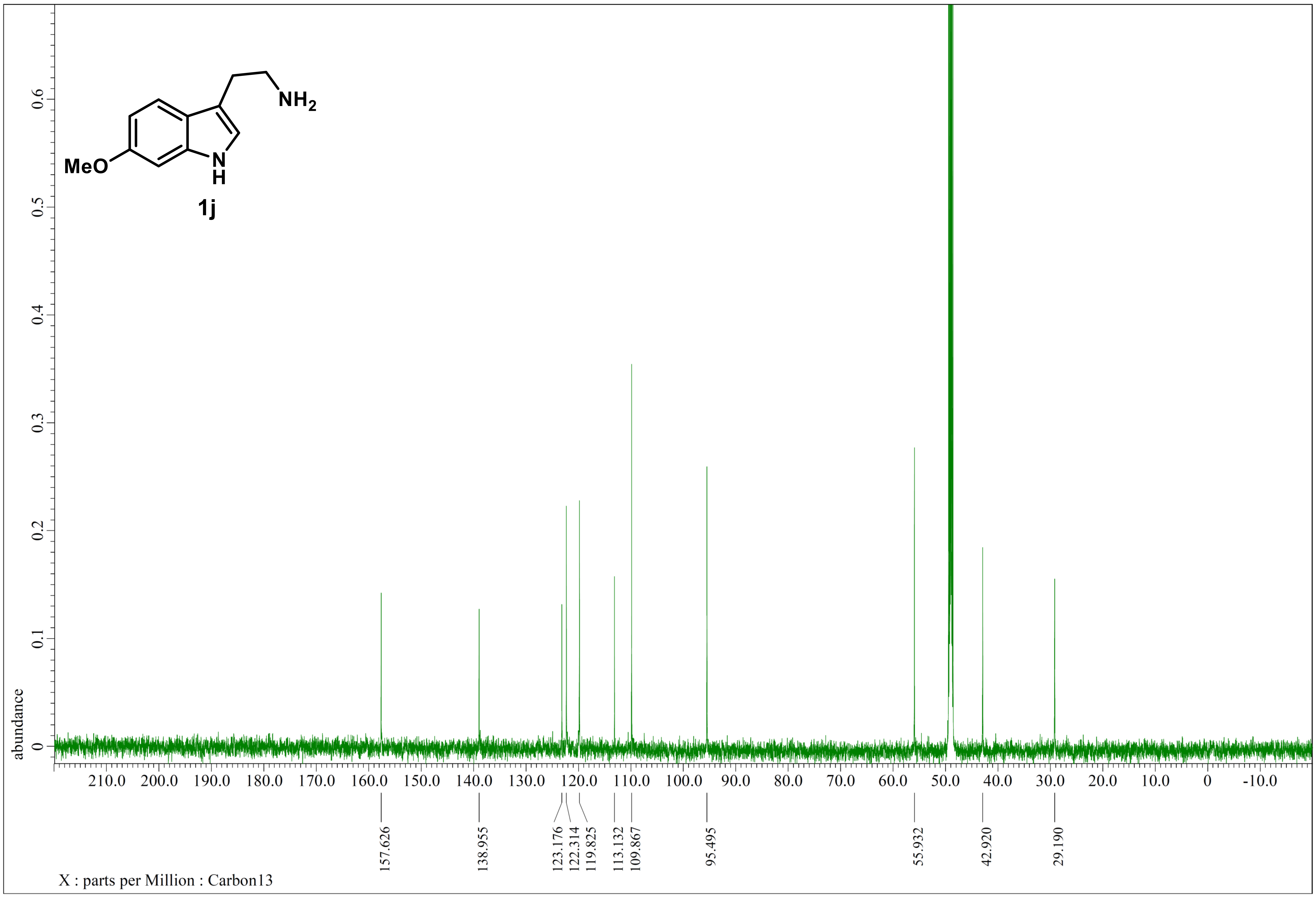

^{1}H NMR (600 MHz, CD_3OD), ^{13}C NMR (151 MHz CD_3OD) and ^{19}F NMR (565 MHz CD_3OD) spectra of **1l**

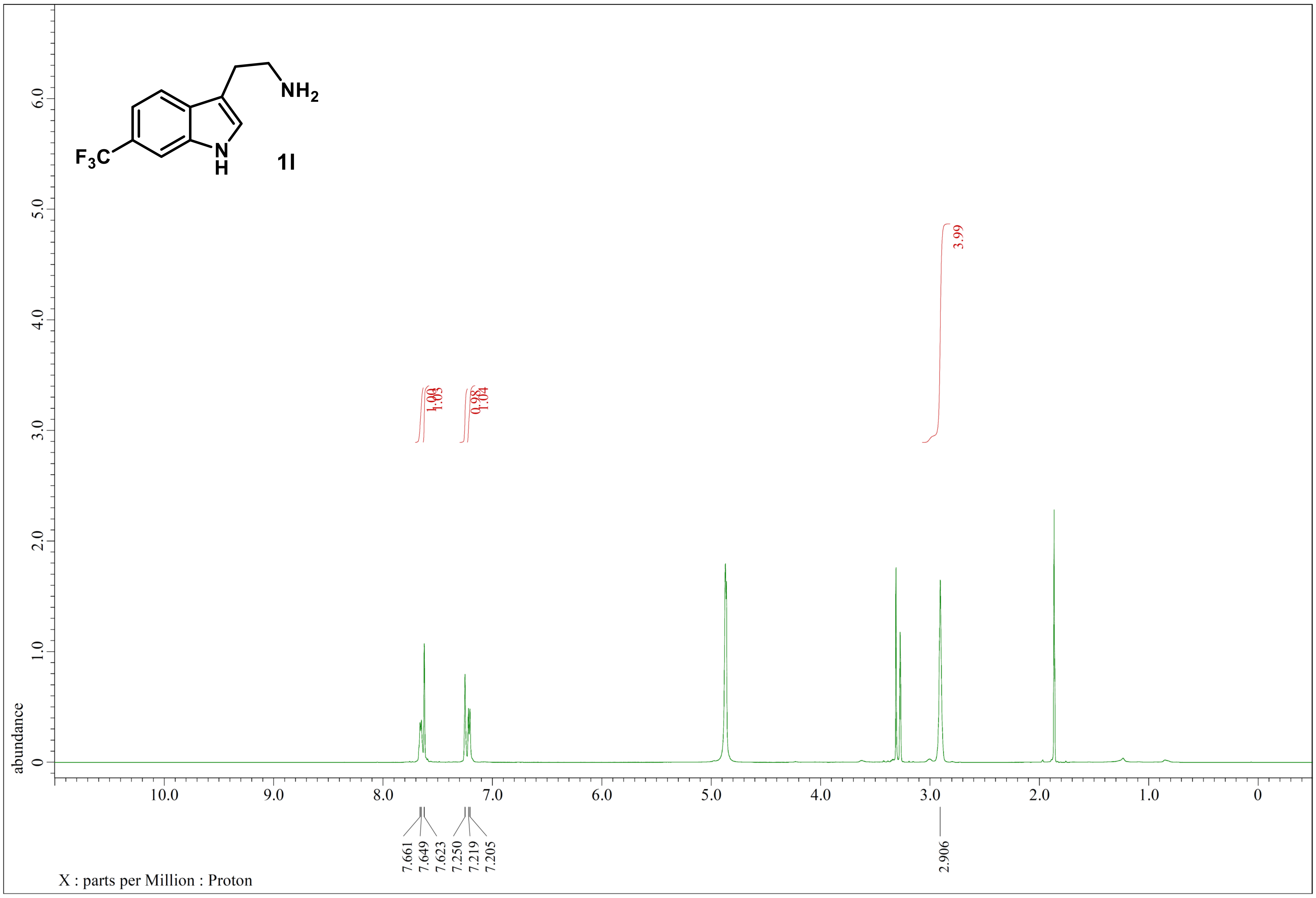

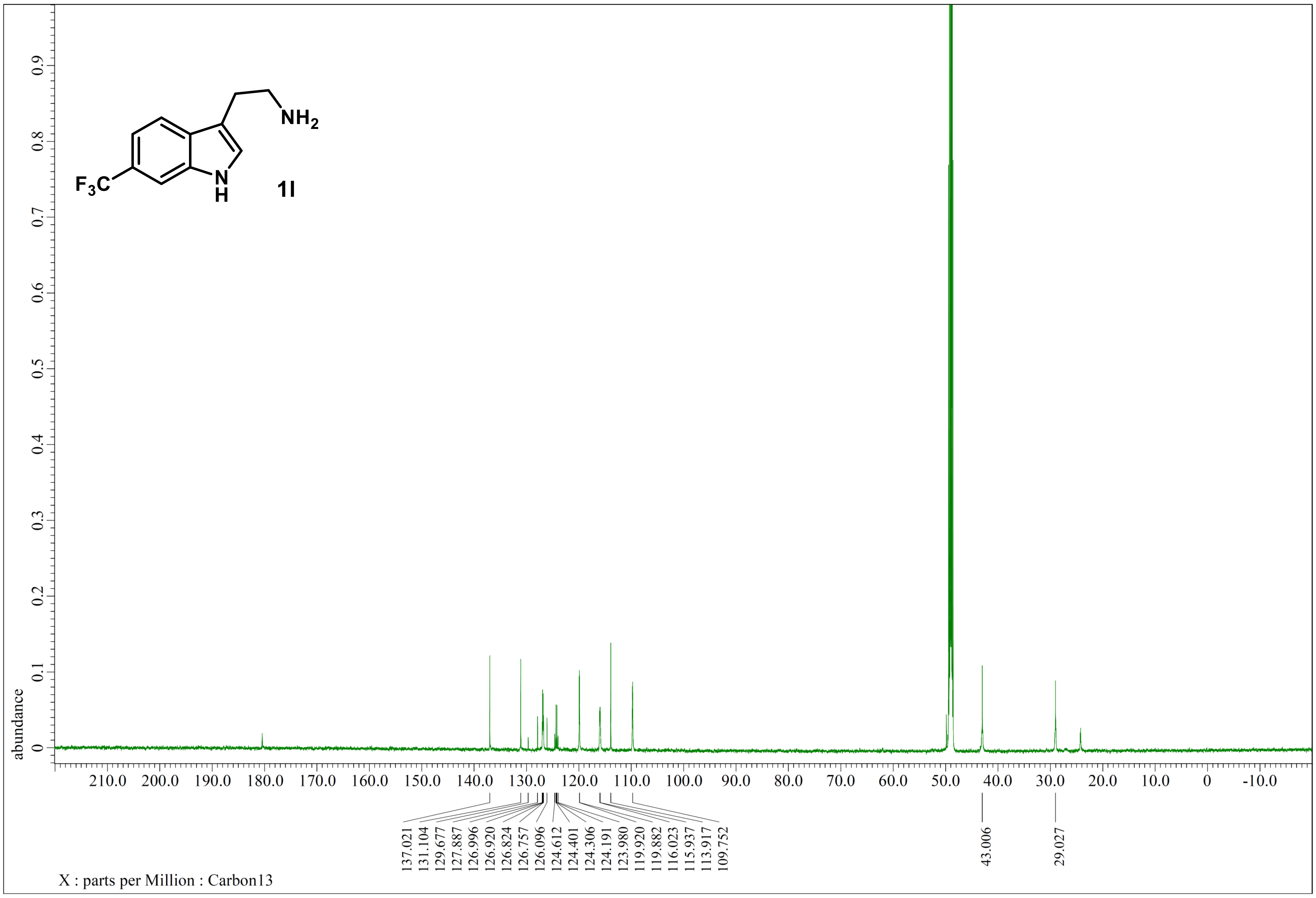
NH2
F3C
N
H
1l
abundance
X : parts per Million : Carbon13
137.021
131.104
129.677
127.887
126.996
126.920
126.824
126.757
126.096
124.612
124.401
124.306
124.191
123.980
119.920
119.882
116.023
115.937
113.917
109.752
43.006
29.027

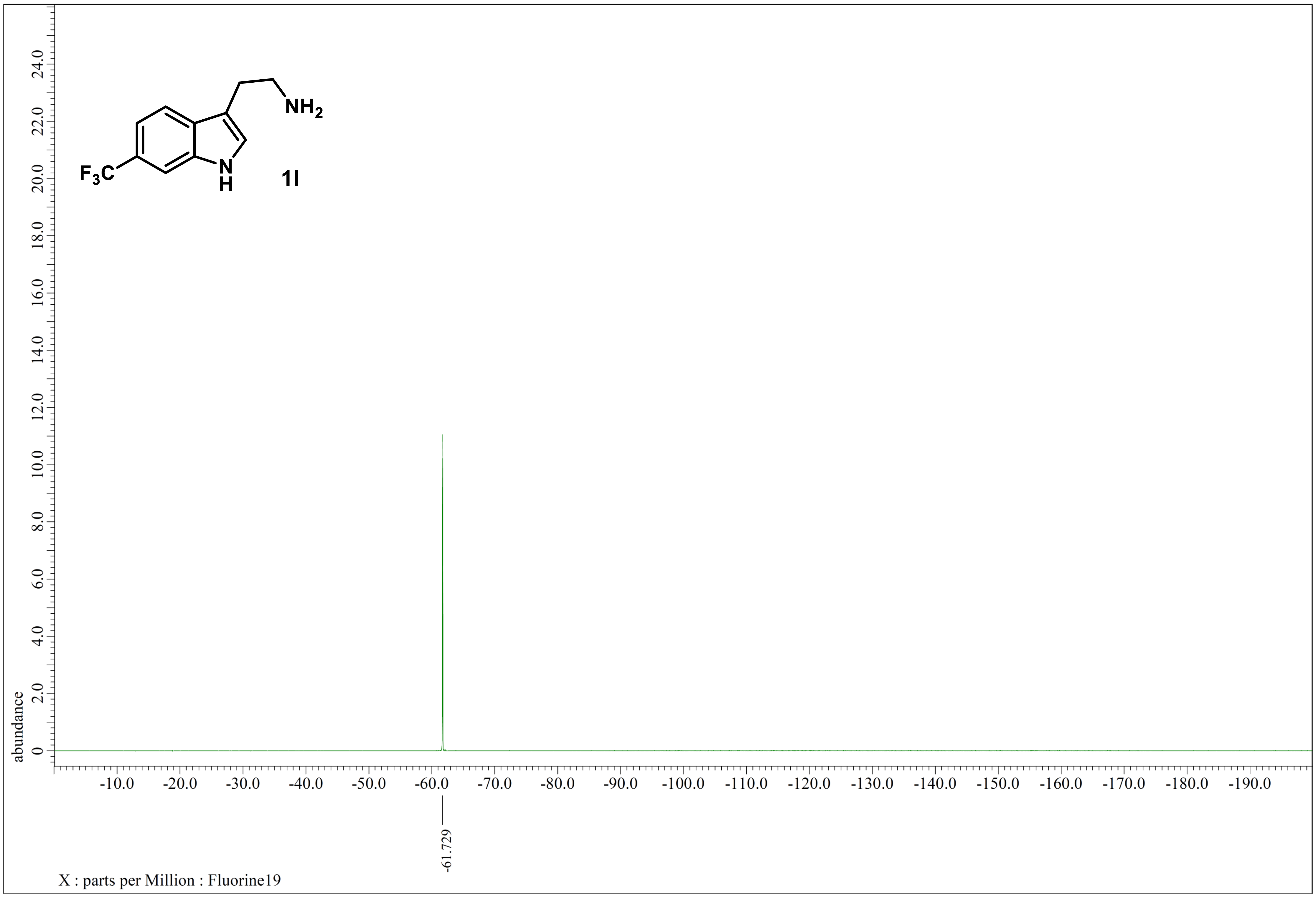
NH2
F3C
N
H
1l
abundance
X : parts per Million : Fluorine19
-61.729

^{1}H NMR (600 MHz, CD_3OD) and ^{13}C NMR (151 MHz CD_3OD) spectra of **1n**

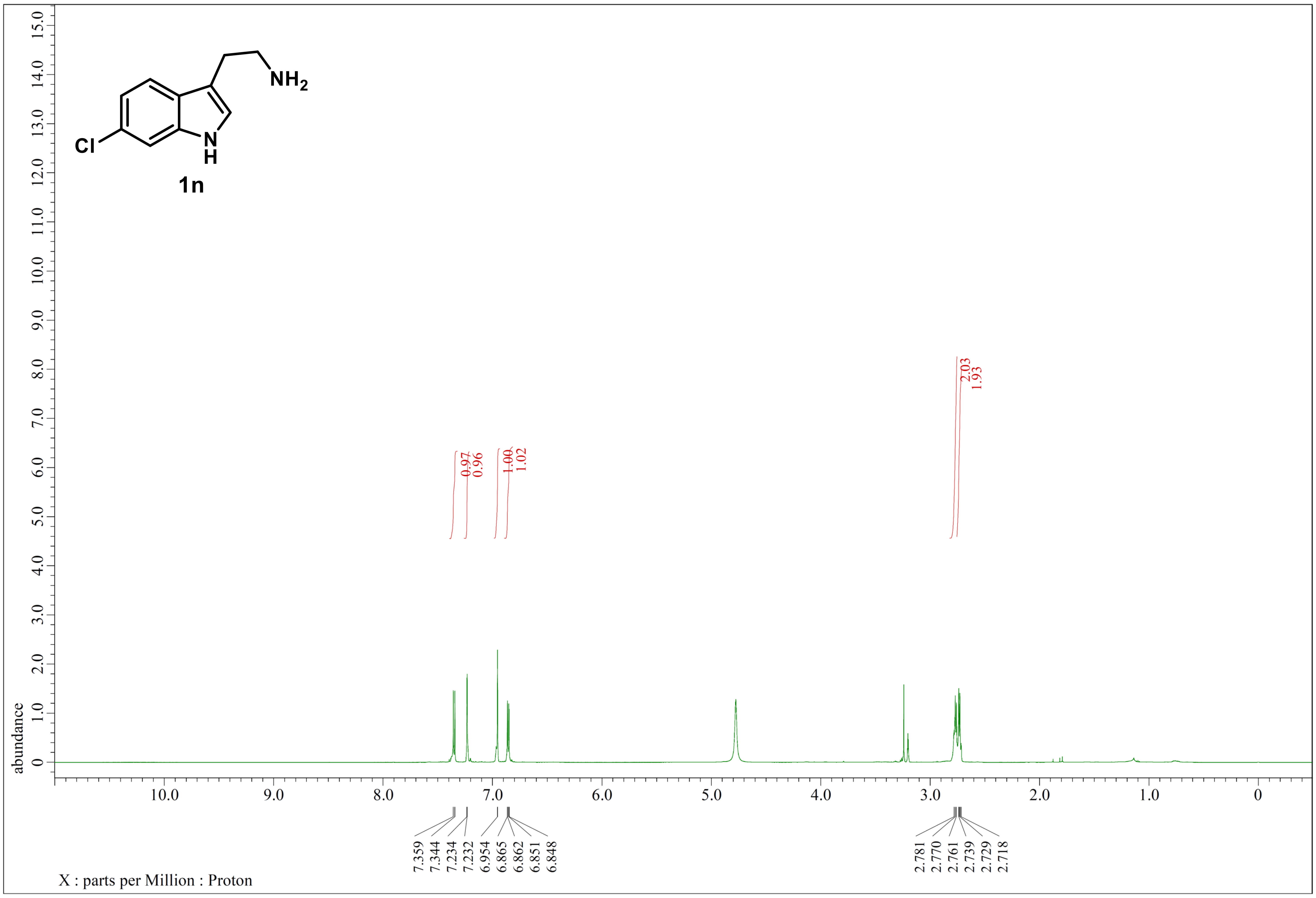

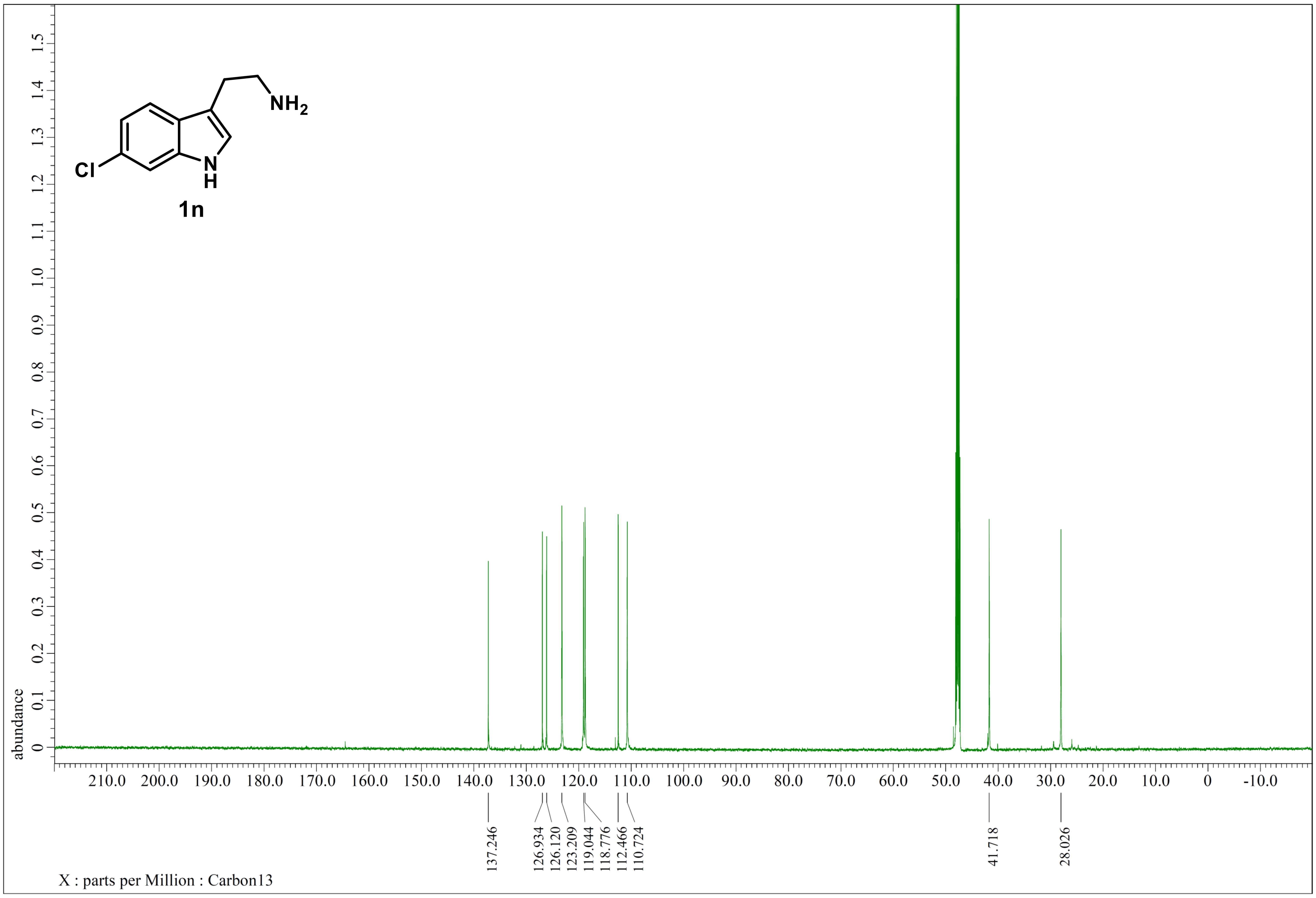

^{1}H NMR (600 MHz, CD_3OD) and ^{13}C NMR (151 MHz CD_3OD) spectra of **1o**

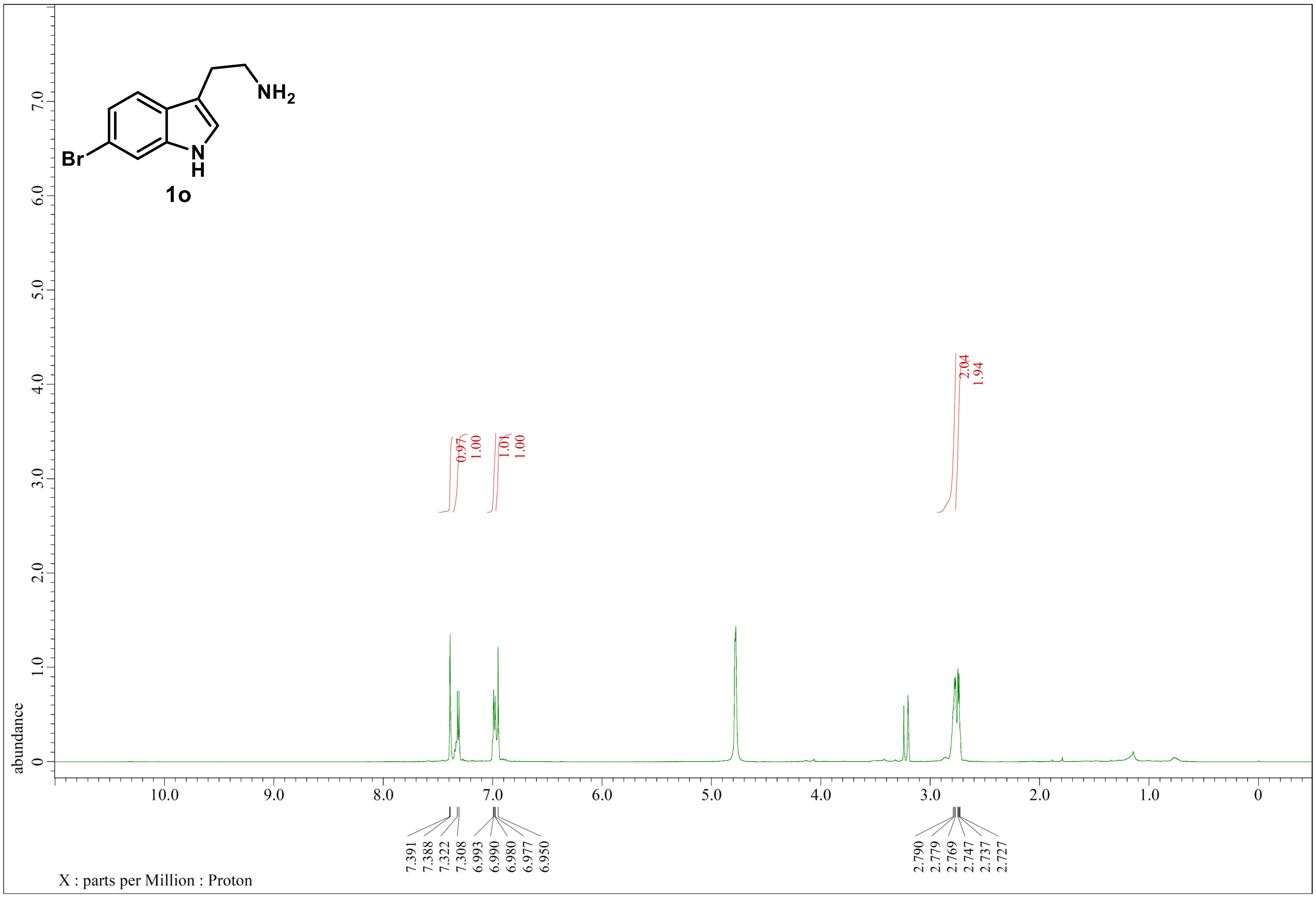

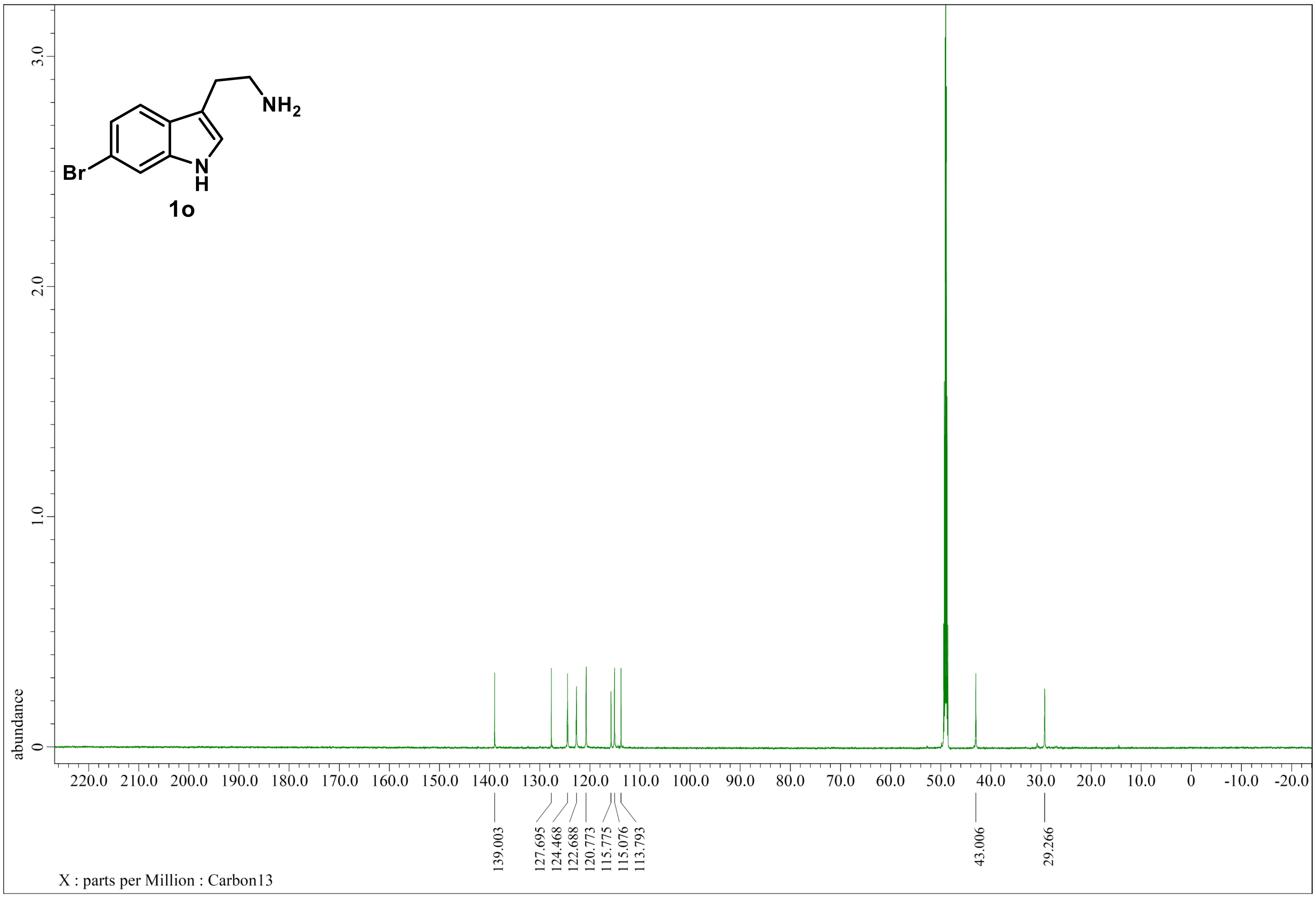

^{1}H NMR (600 MHz, $CDCl_3$), ^{13}C NMR (151 MHz $CDCl_3$) and ^{19}F NMR (565 MHz $CDCl_3$) spectra of **2b**

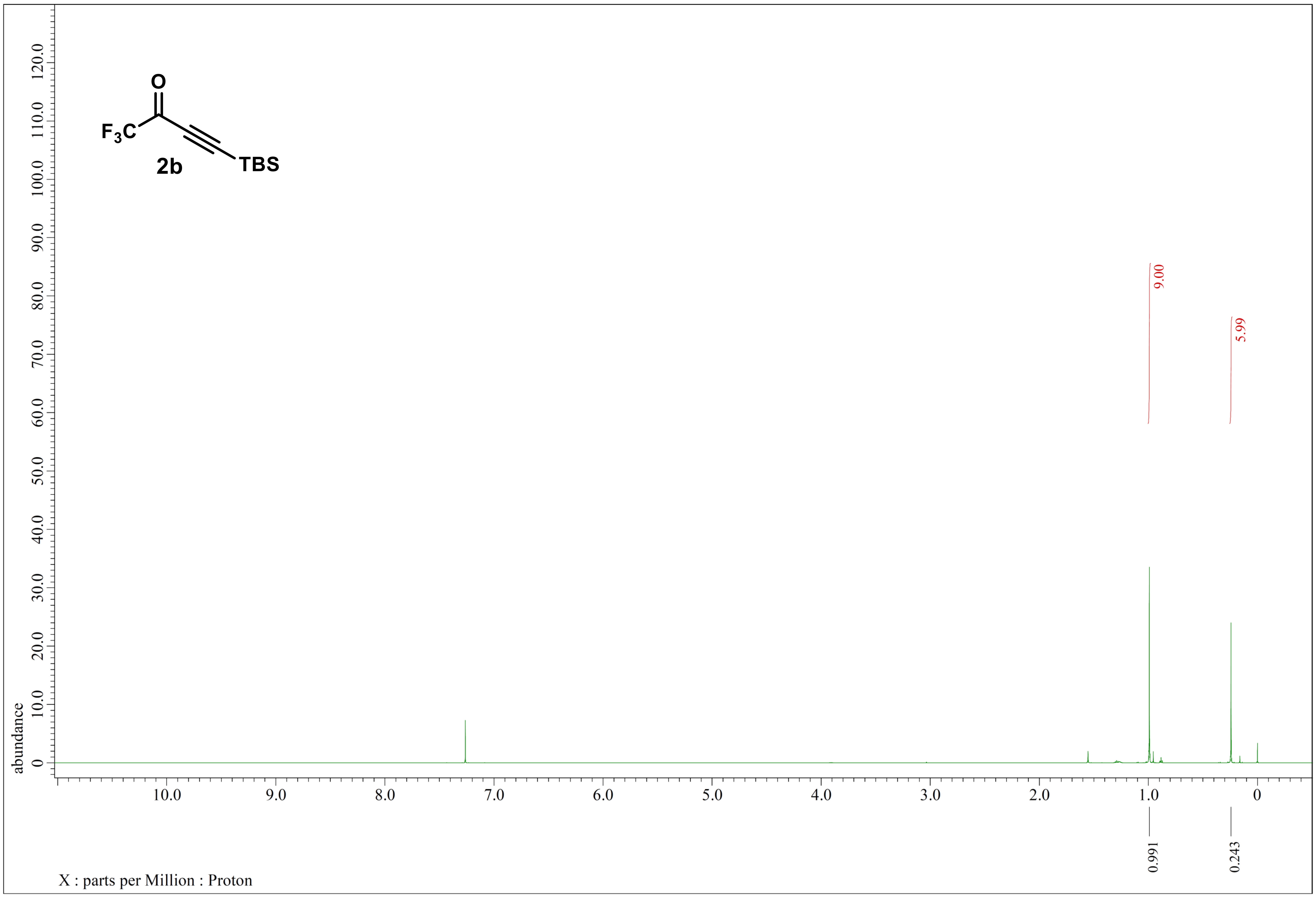

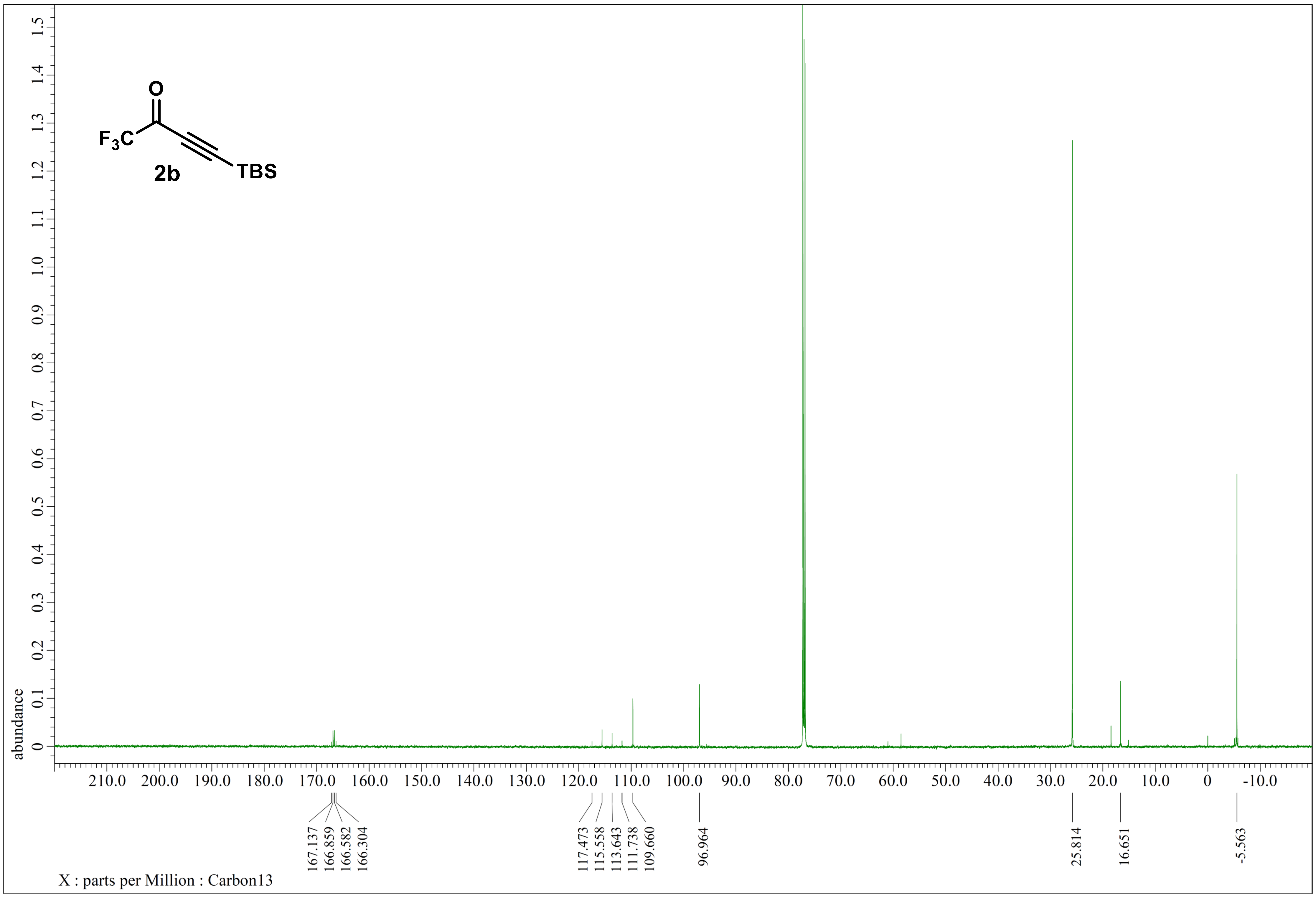

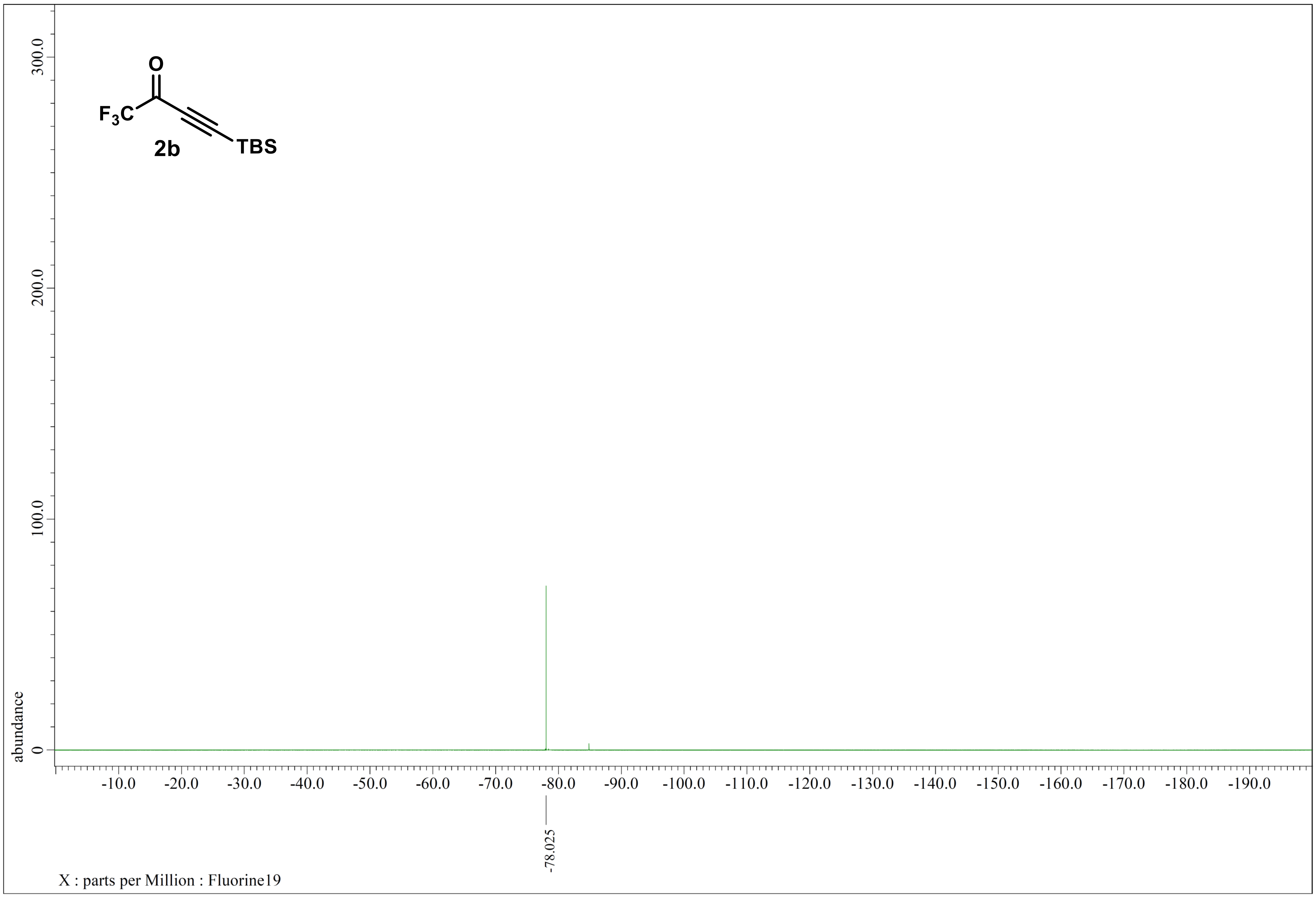

^{1}H NMR (600 MHz, $CDCl_3$), ^{13}C NMR (151 MHz $CDCl_3$) and ^{19}F NMR (565 MHz $CDCl_3$) spectra of **2b**

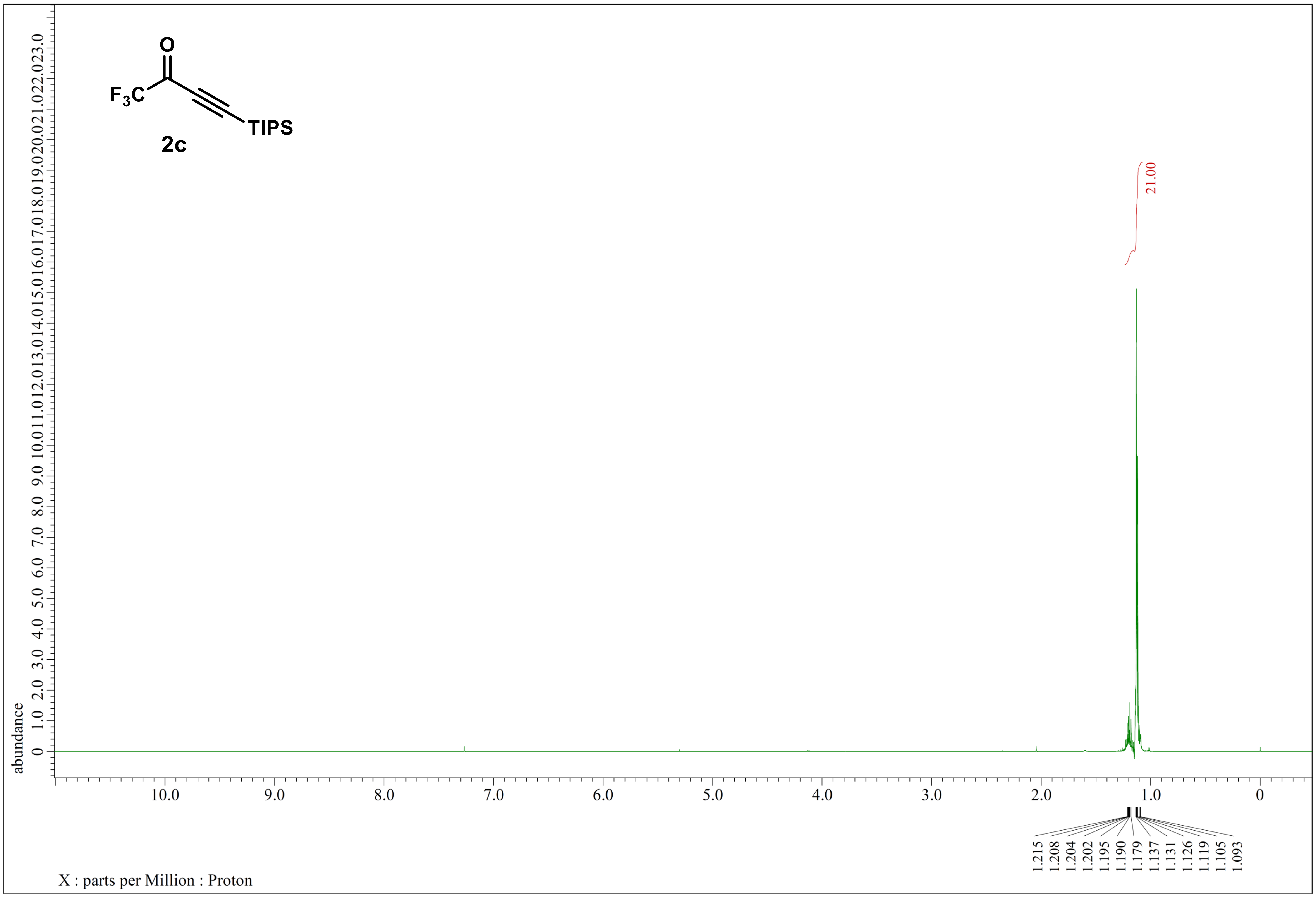

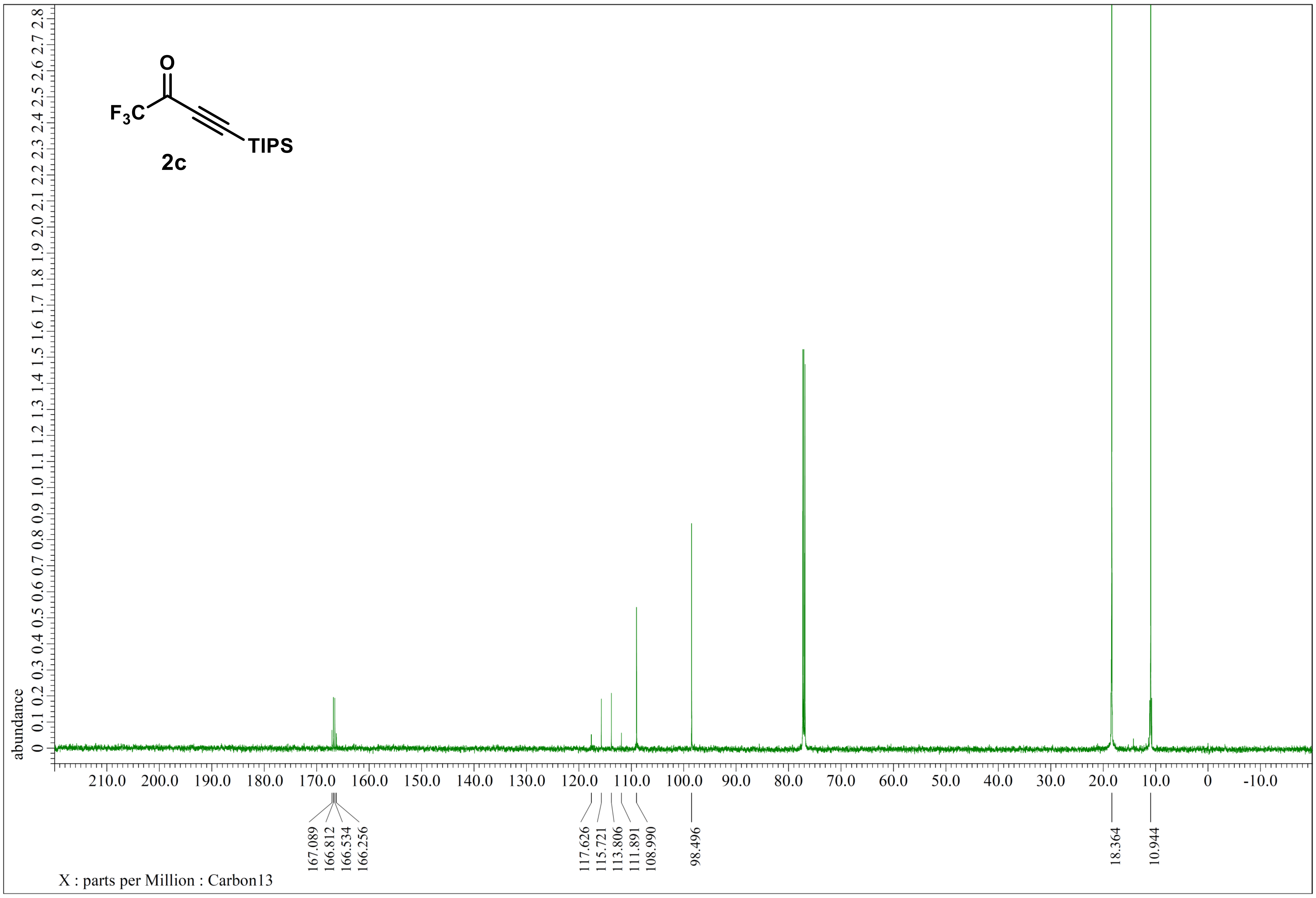
F3C
O
TIPS
2c
abundance
X : parts per Million : Carbon13
167.089
166.812
166.534
166.256
117.626
115.721
113.806
111.891
108.990
98.496
18.364
10.944

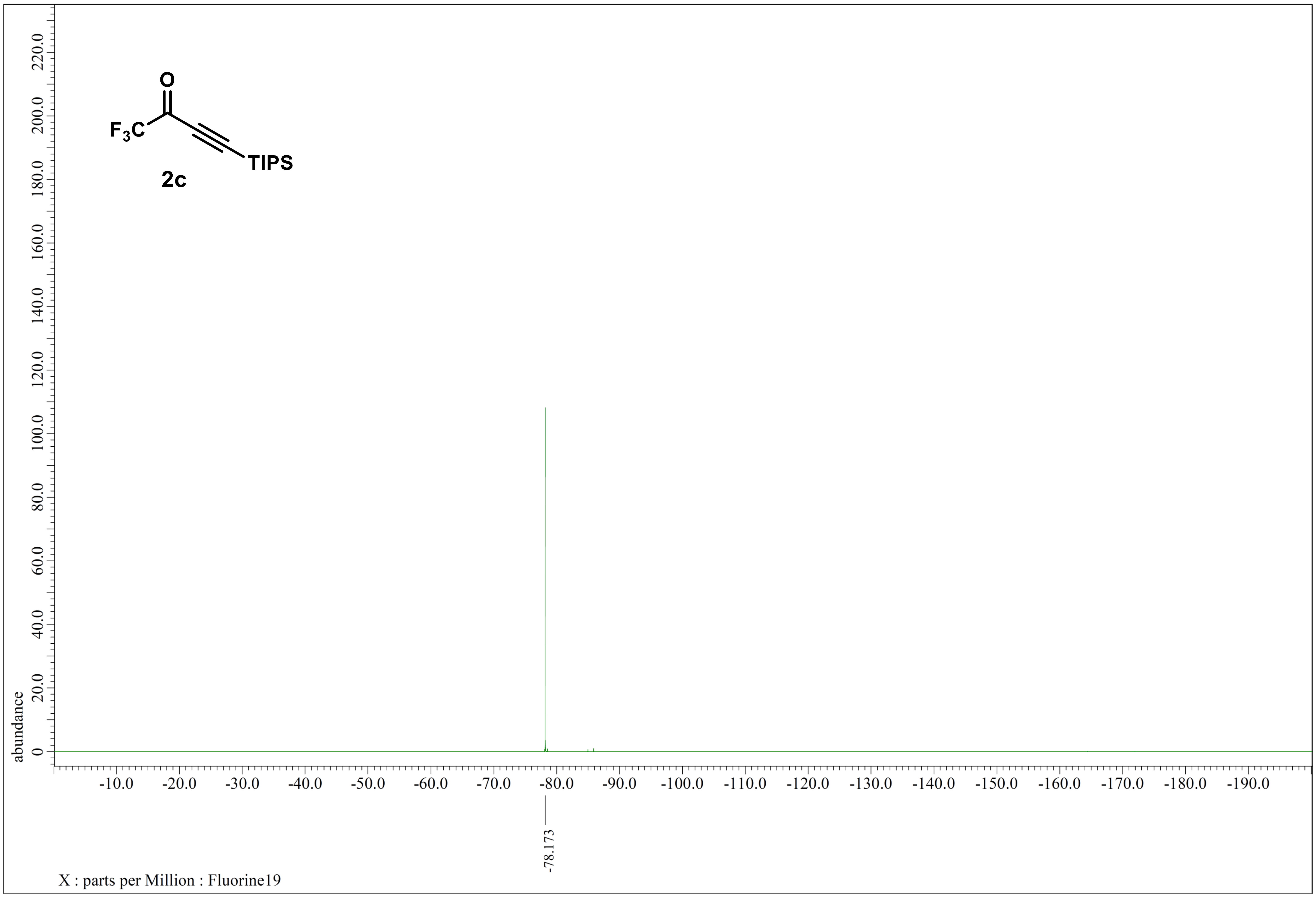
F3C
O
TIPS
2c
abundance
X : parts per Million : Fluorine19
-78.173

^{1}H NMR (600 MHz, $CDCl_3$), ^{13}C NMR (151 MHz $CDCl_3$) and ^{19}F NMR (565 MHz $CDCl_3$) spectra of **4ac**

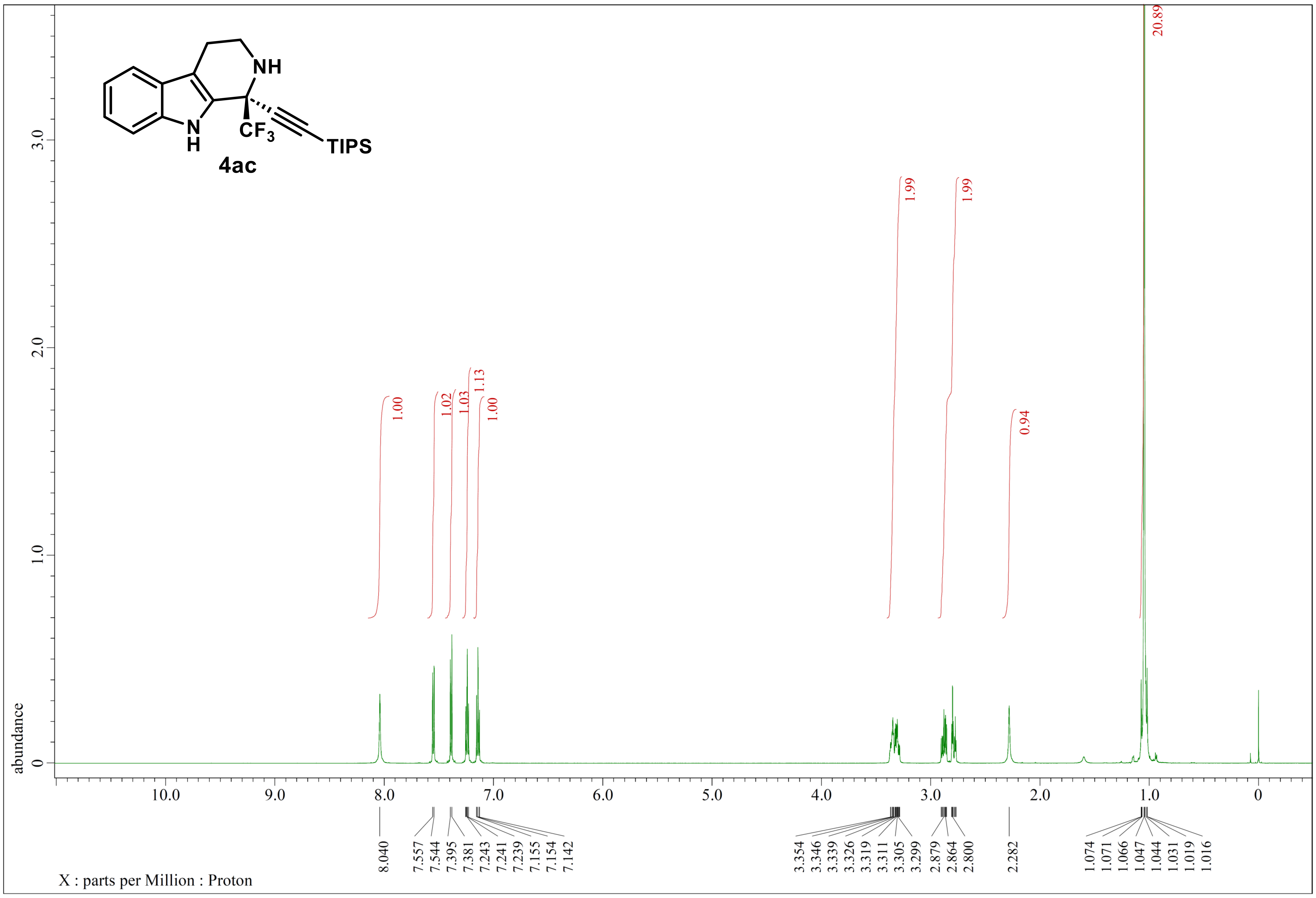

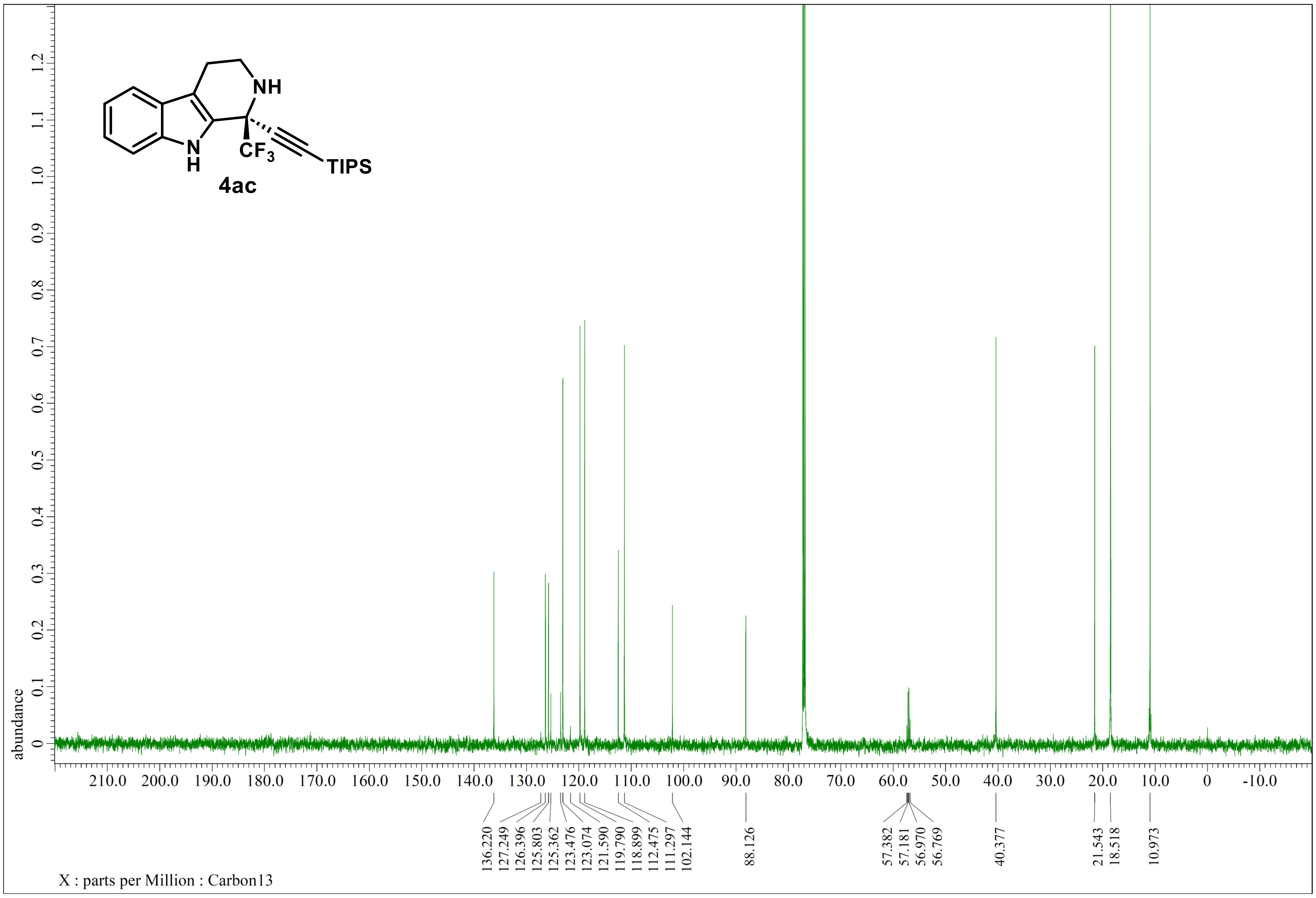

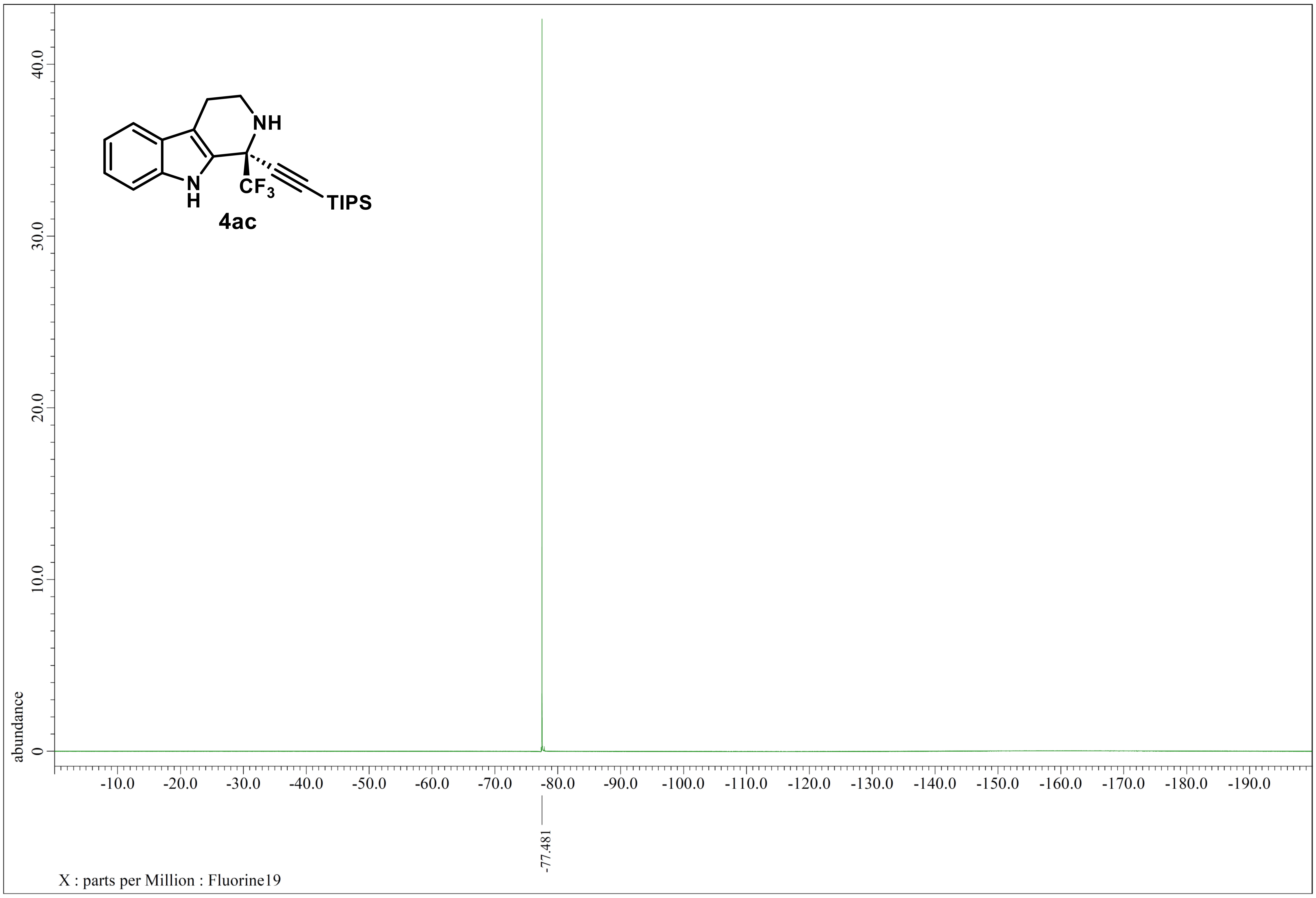

^{1}H NMR (600 MHz, $CDCl_3$), ^{13}C NMR (151 MHz $CDCl_3$) and ^{19}F NMR (565 MHz $CDCl_3$) spectra of **4b**

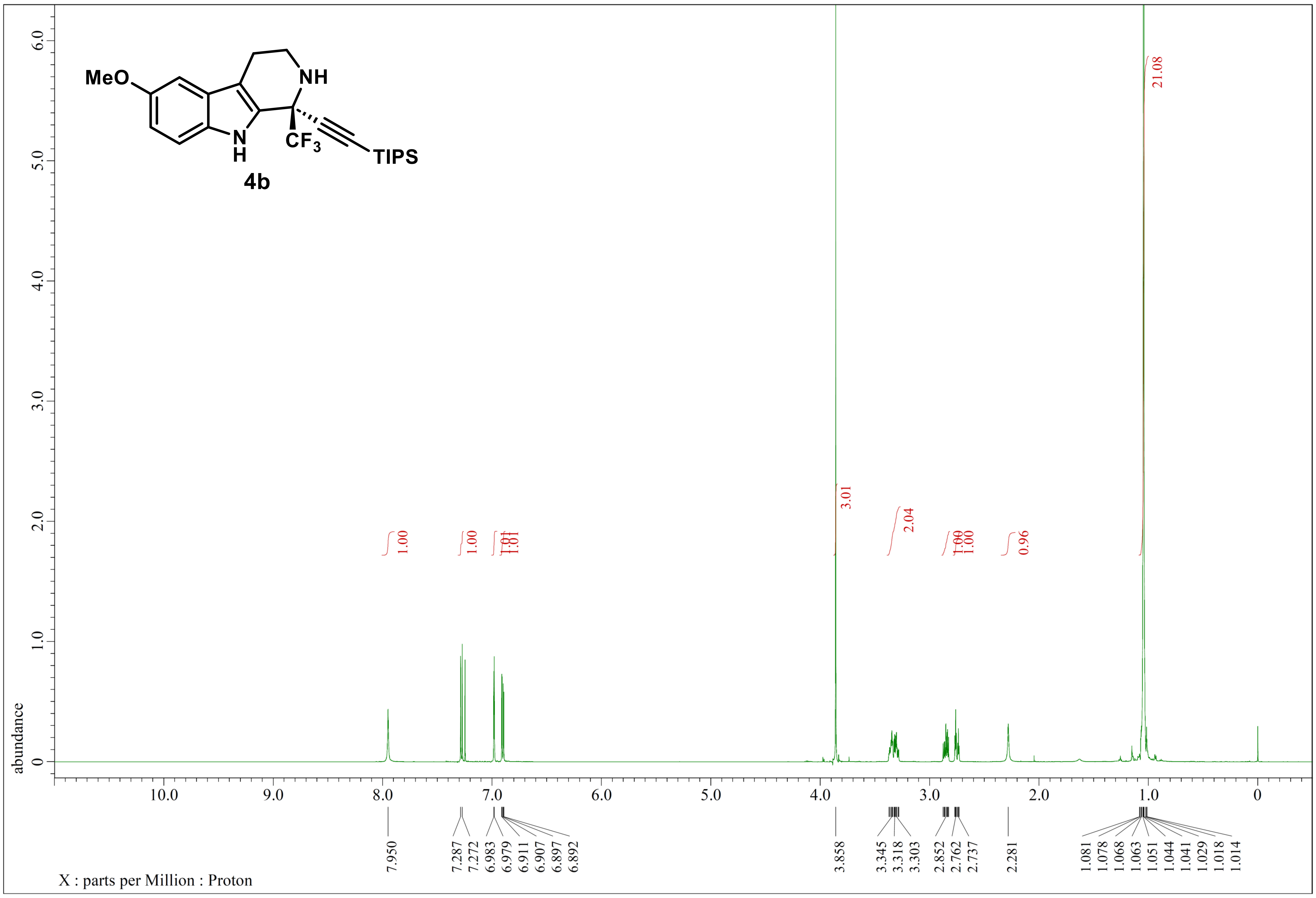

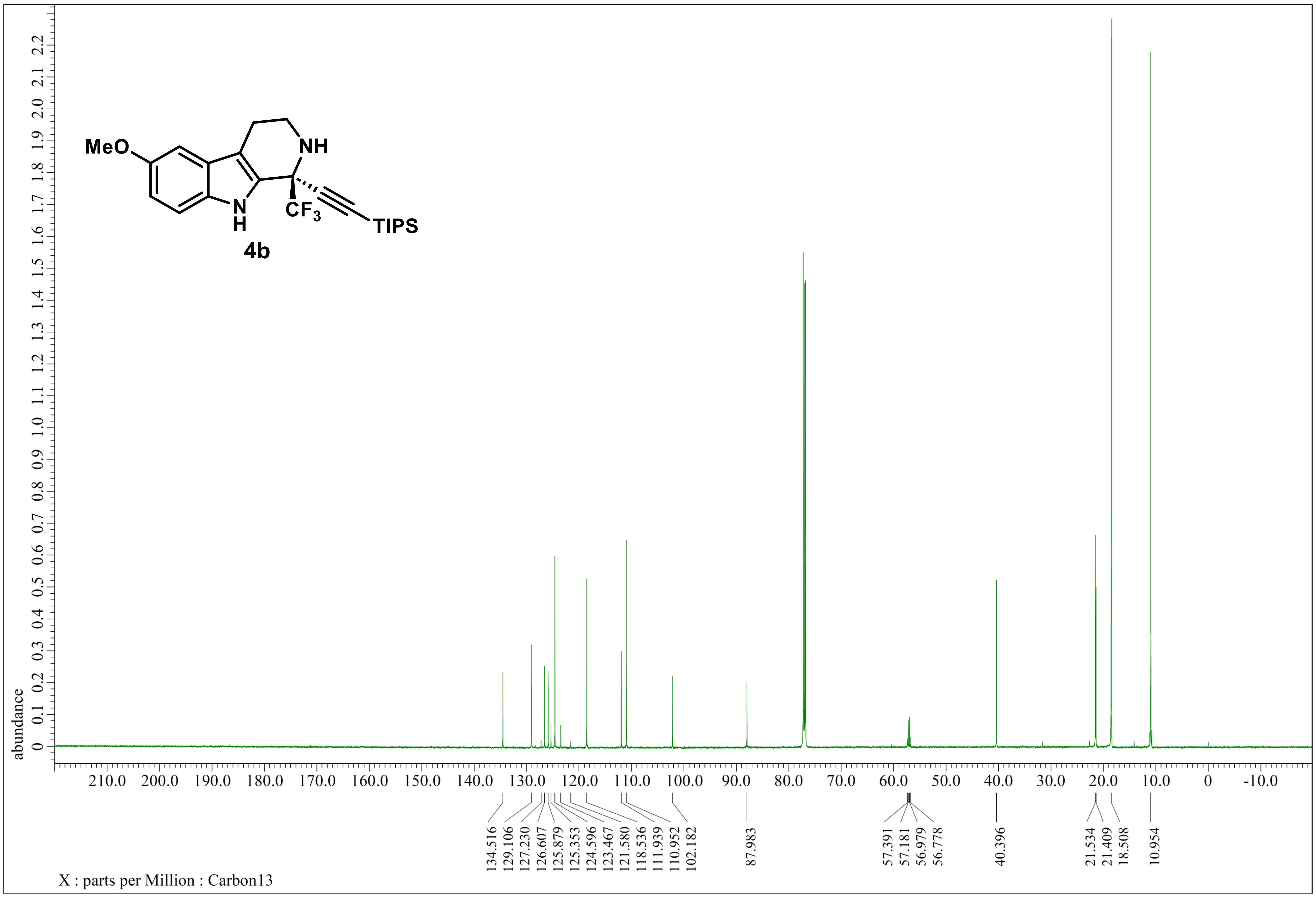
MeO
NH
N
H
CF3
TIPS
4b
abundance
134.516
129.106
127.230
126.607
125.879
125.353
124.596
123.467
121.580
118.536
111.939
110.952
102.182
87.983
57.391
57.181
56.979
56.778
40.396
21.534
21.409
18.508
10.954
X : parts per Million : Carbon13

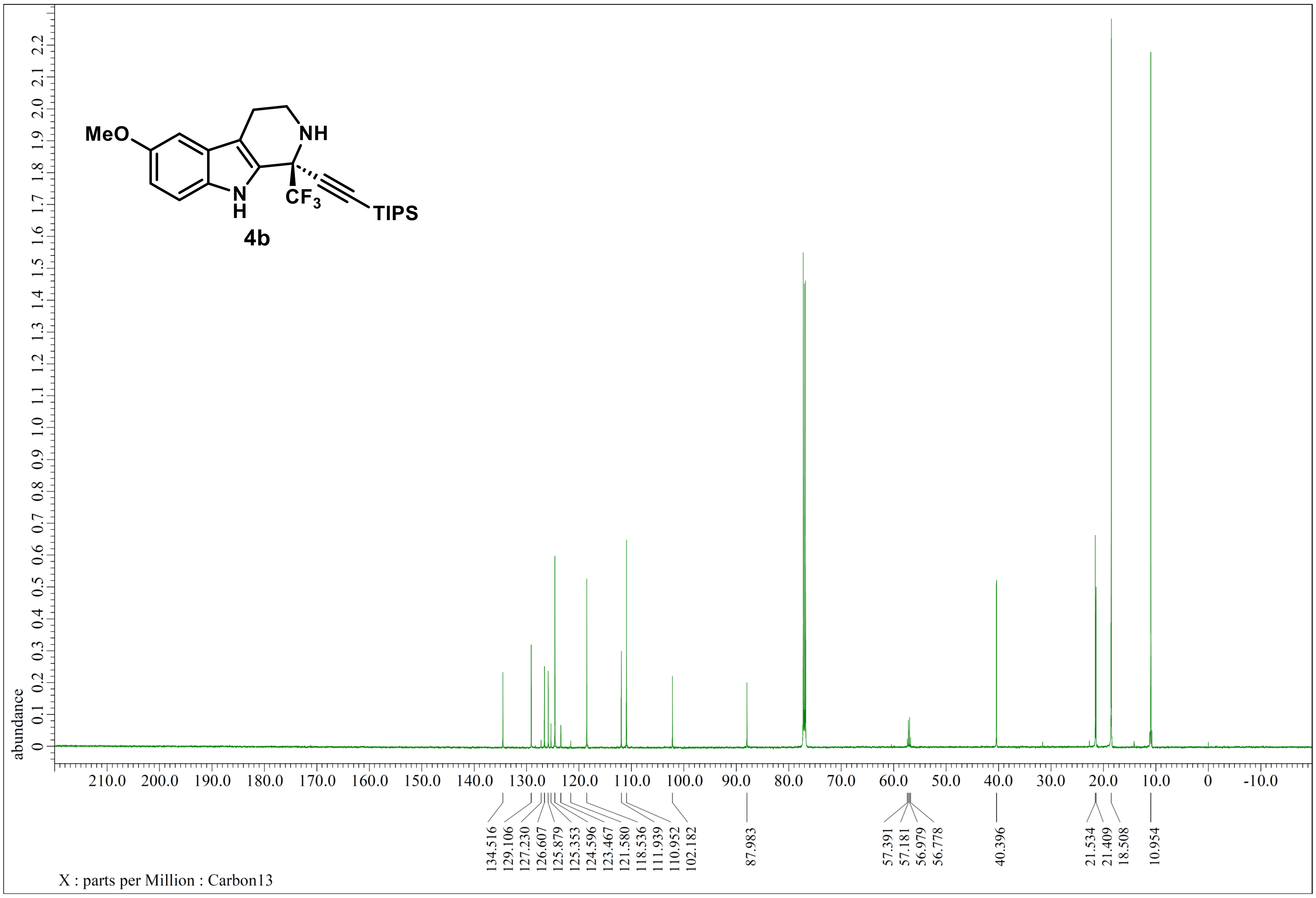
MeO
NH
N
H
CF3
TIPS
4b
abundance
134.516
129.106
127.230
126.607
125.879
125.353
124.596
123.467
121.580
118.536
111.939
110.952
102.182
87.983
57.391
57.181
56.979
56.778
40.396
21.534
21.409
18.508
10.954
X : parts per Million : Carbon13

^{1}H NMR (600 MHz, $CDCl_3$), ^{13}C NMR (151 MHz $CDCl_3$) and ^{19}F NMR (565 MHz $CDCl_3$) spectra of **4c**

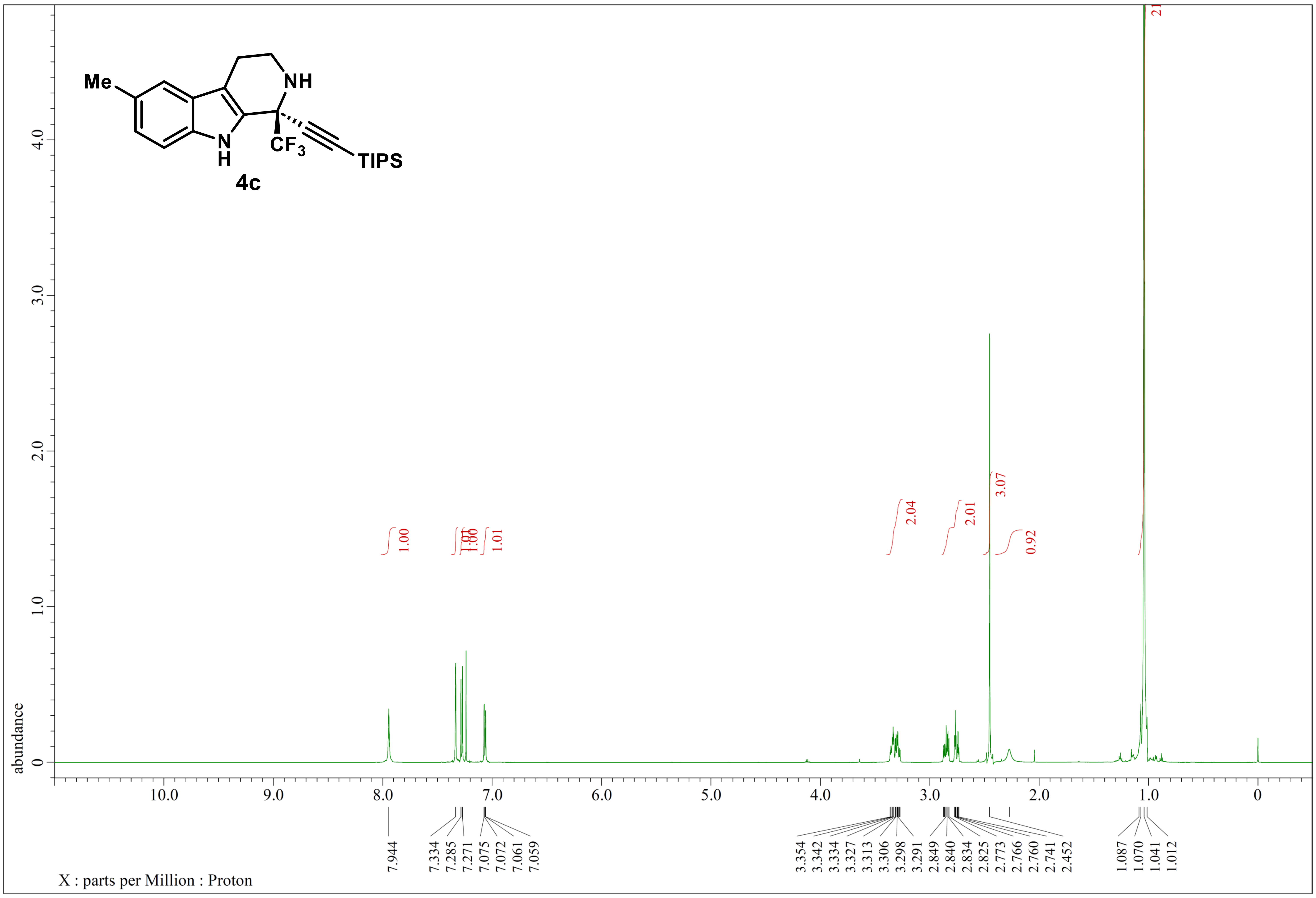

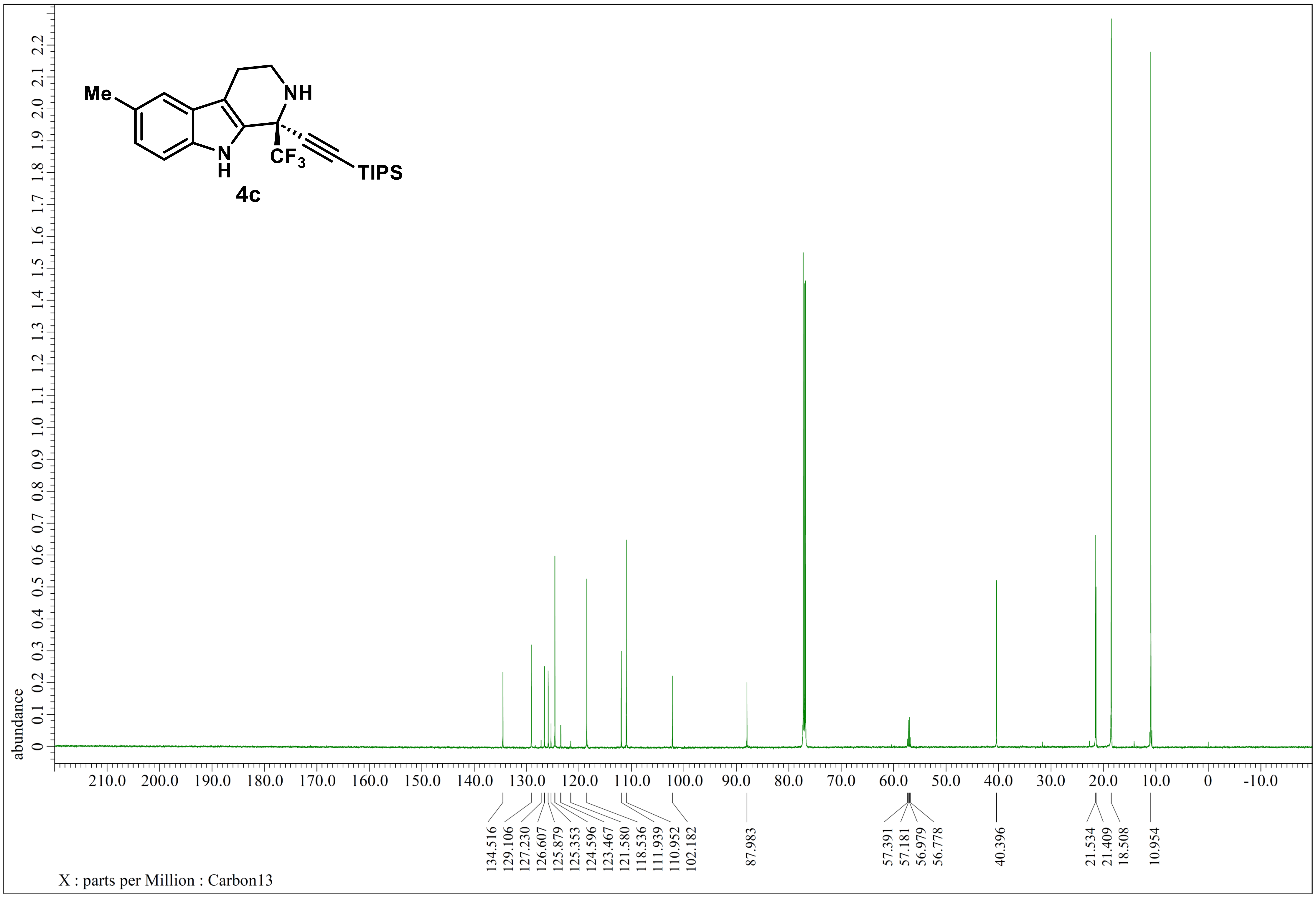

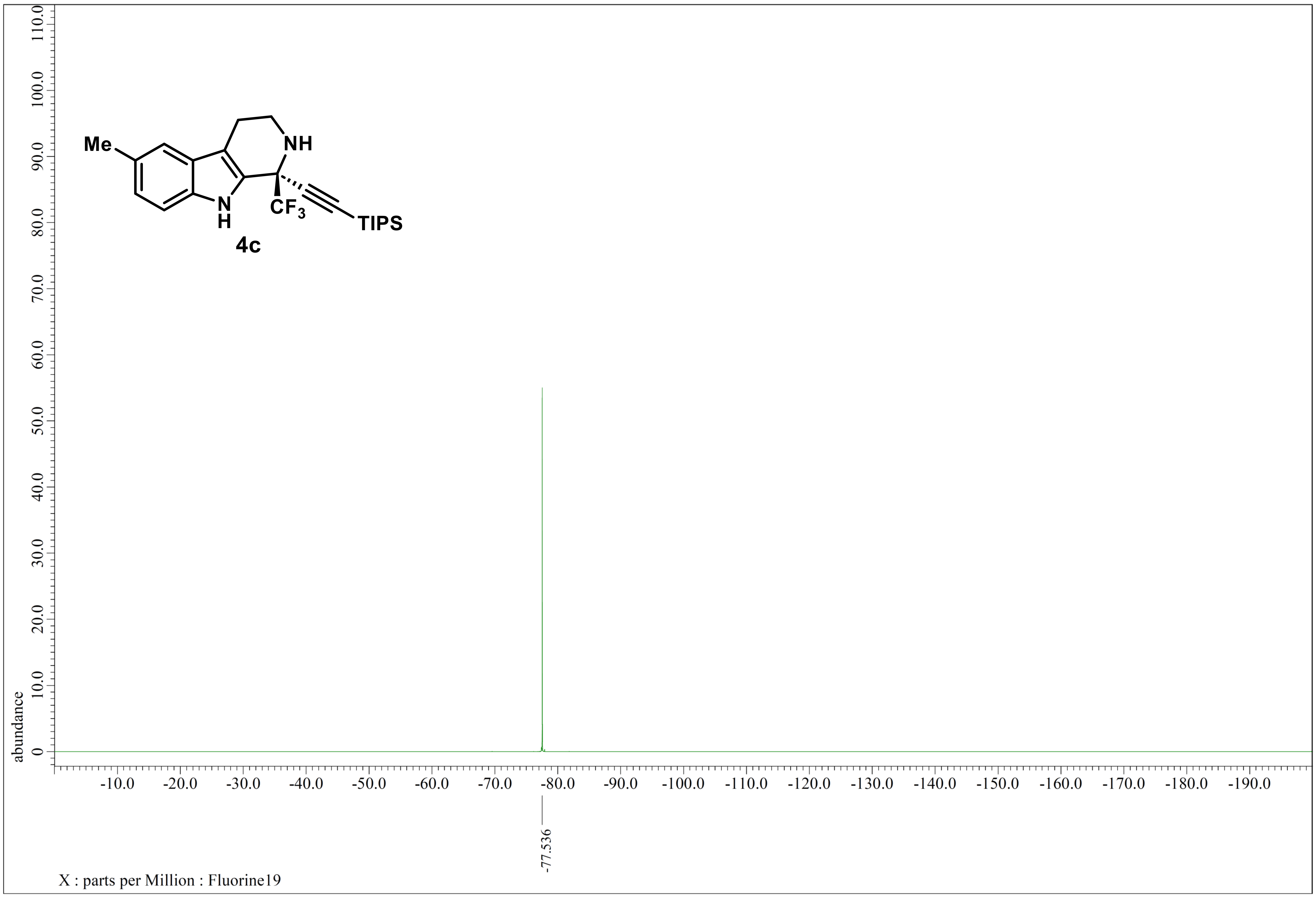

^{1}H NMR (600 MHz, $CDCl_3$), ^{13}C NMR (151 MHz $CDCl_3$) and ^{19}F NMR (565 MHz $CDCl_3$) spectra of **4d**

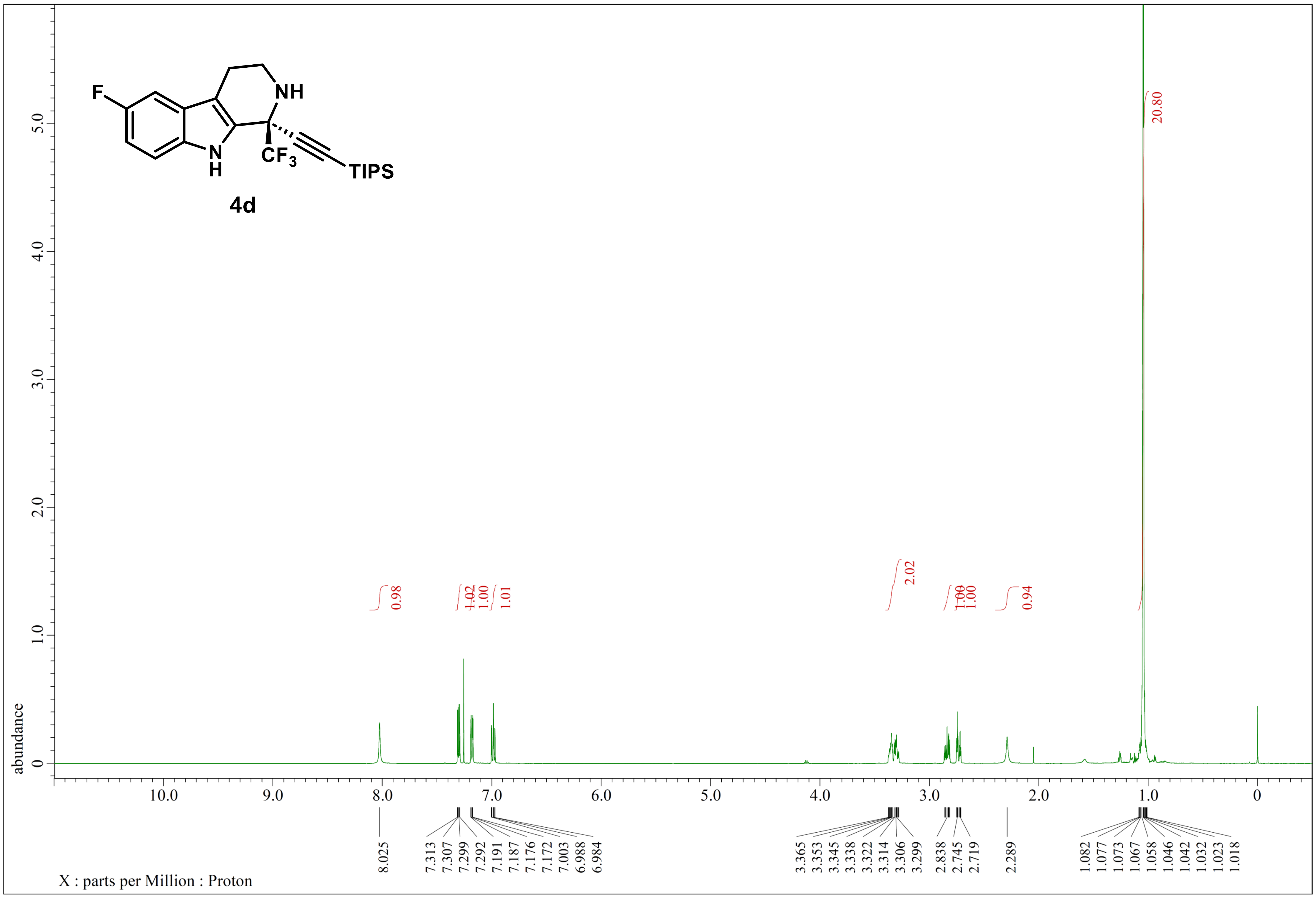

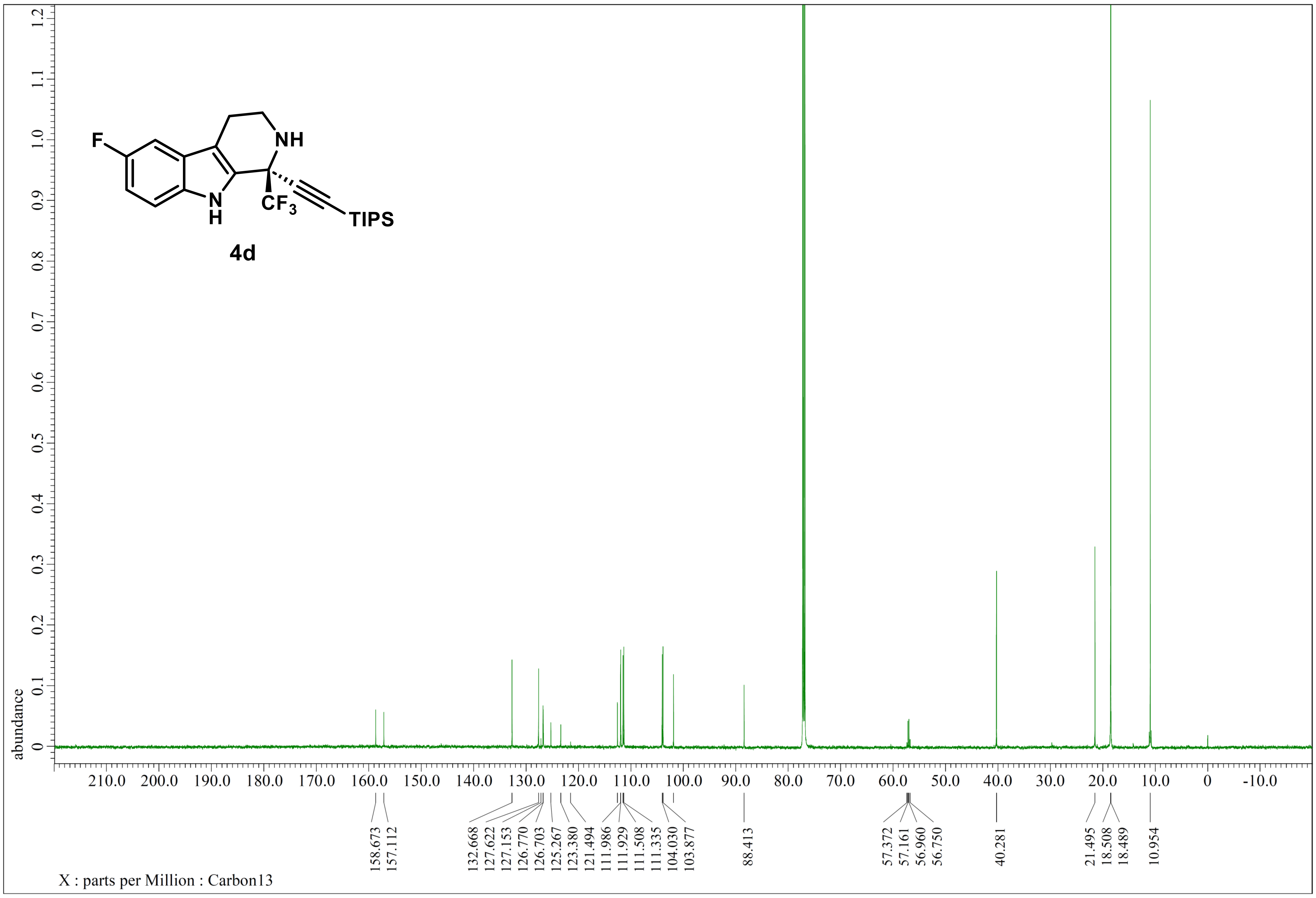
F
NH
N
H
CF_3
TIPS
4d
abundance
X : parts per Million : Carbon13
158.673
157.112
132.668
127.622
127.153
126.770
126.703
125.267
123.380
121.494
111.986
111.929
111.508
111.335
104.030
103.877
88.413
57.372
57.161
56.960
56.750
40.281
21.495
18.508
18.489
10.954

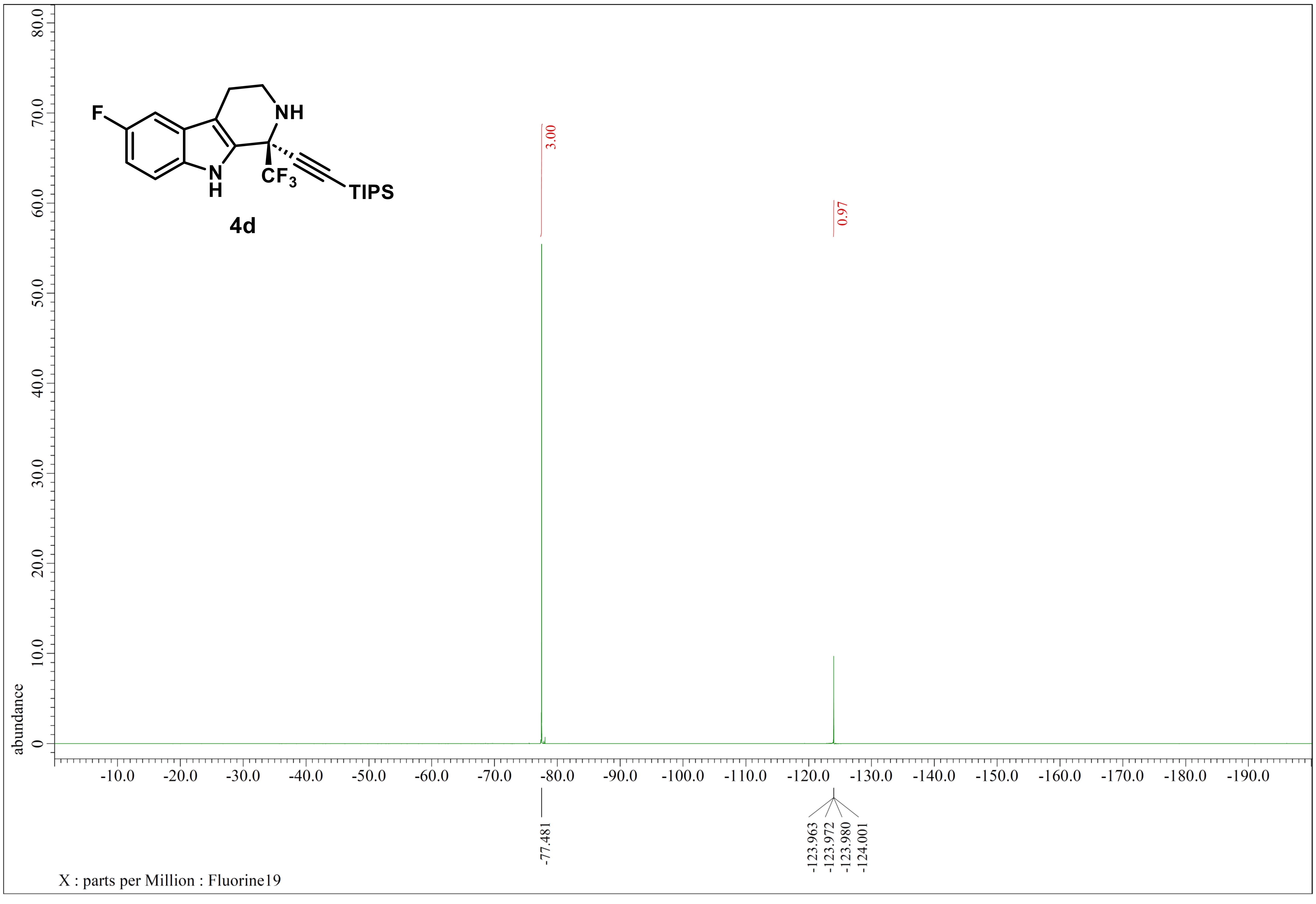
F
NH
N
H
CF_3
TIPS
4d
3.00
0.97
abundance
X : parts per Million : Fluorine19
-77.481
-123.963
-123.972
-123.980
-124.001

^{1}H NMR (600 MHz, $CDCl_3$), ^{13}C NMR (151 MHz $CDCl_3$) and ^{19}F NMR (565 MHz $CDCl_3$) spectra of **4e**

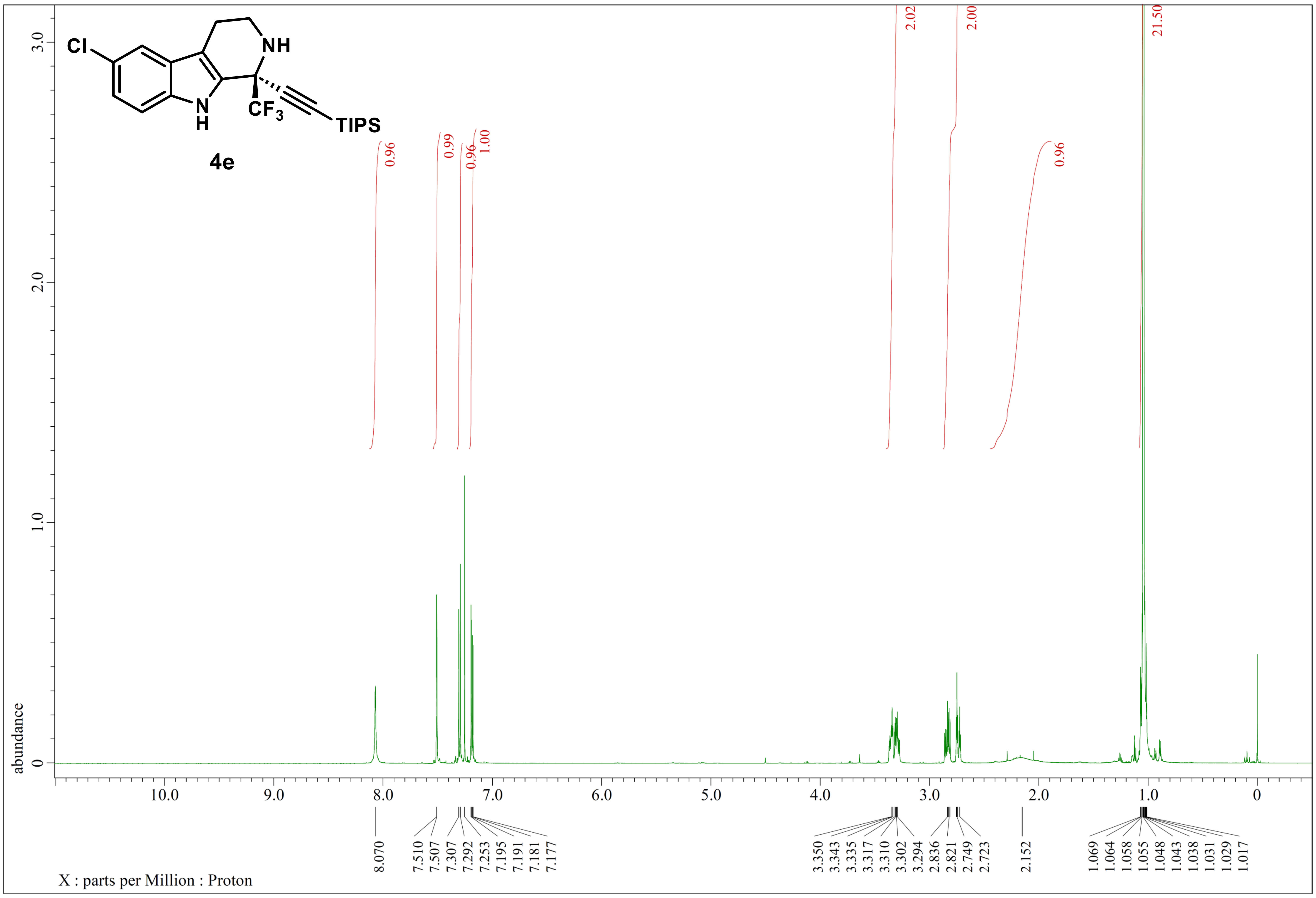

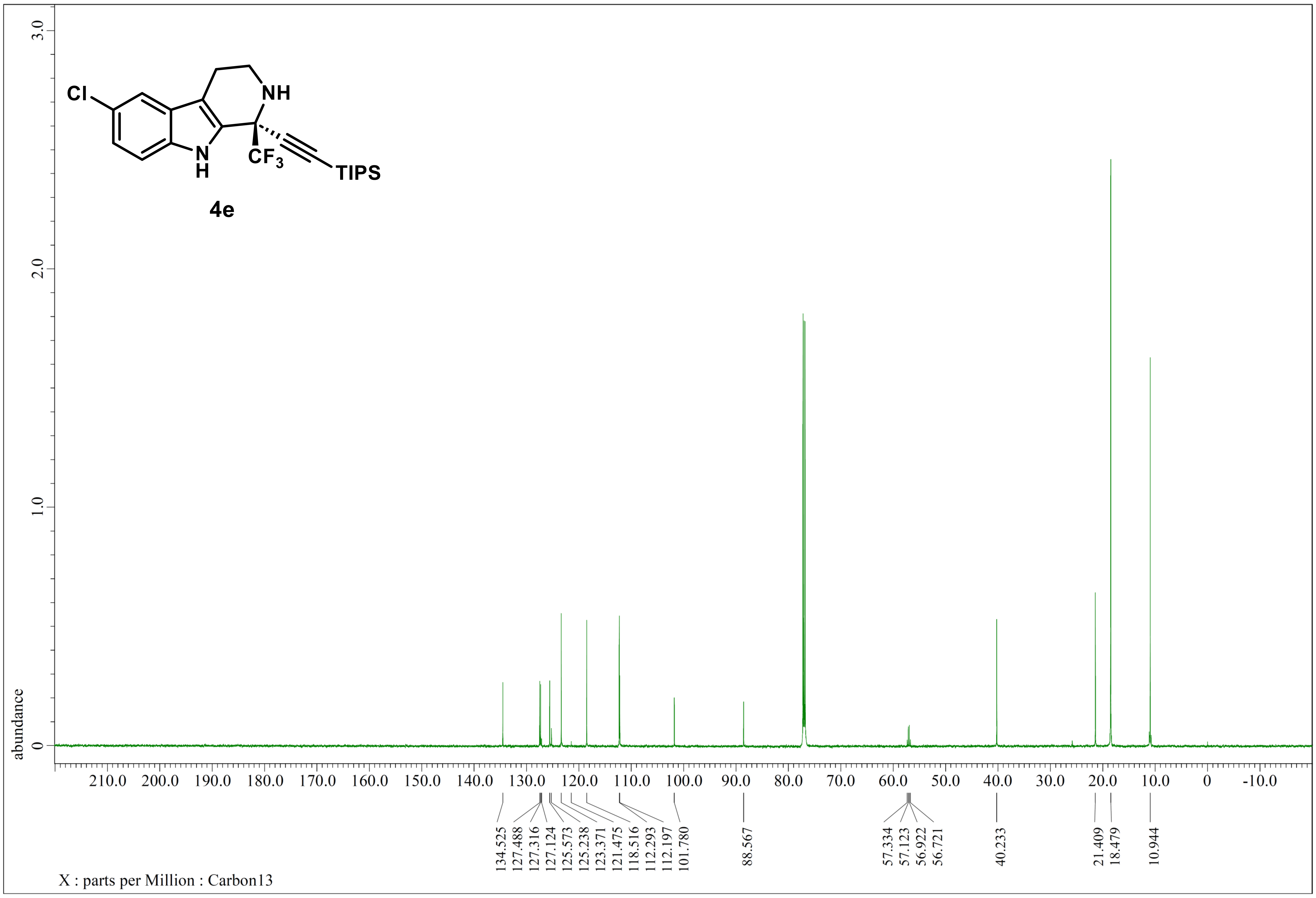

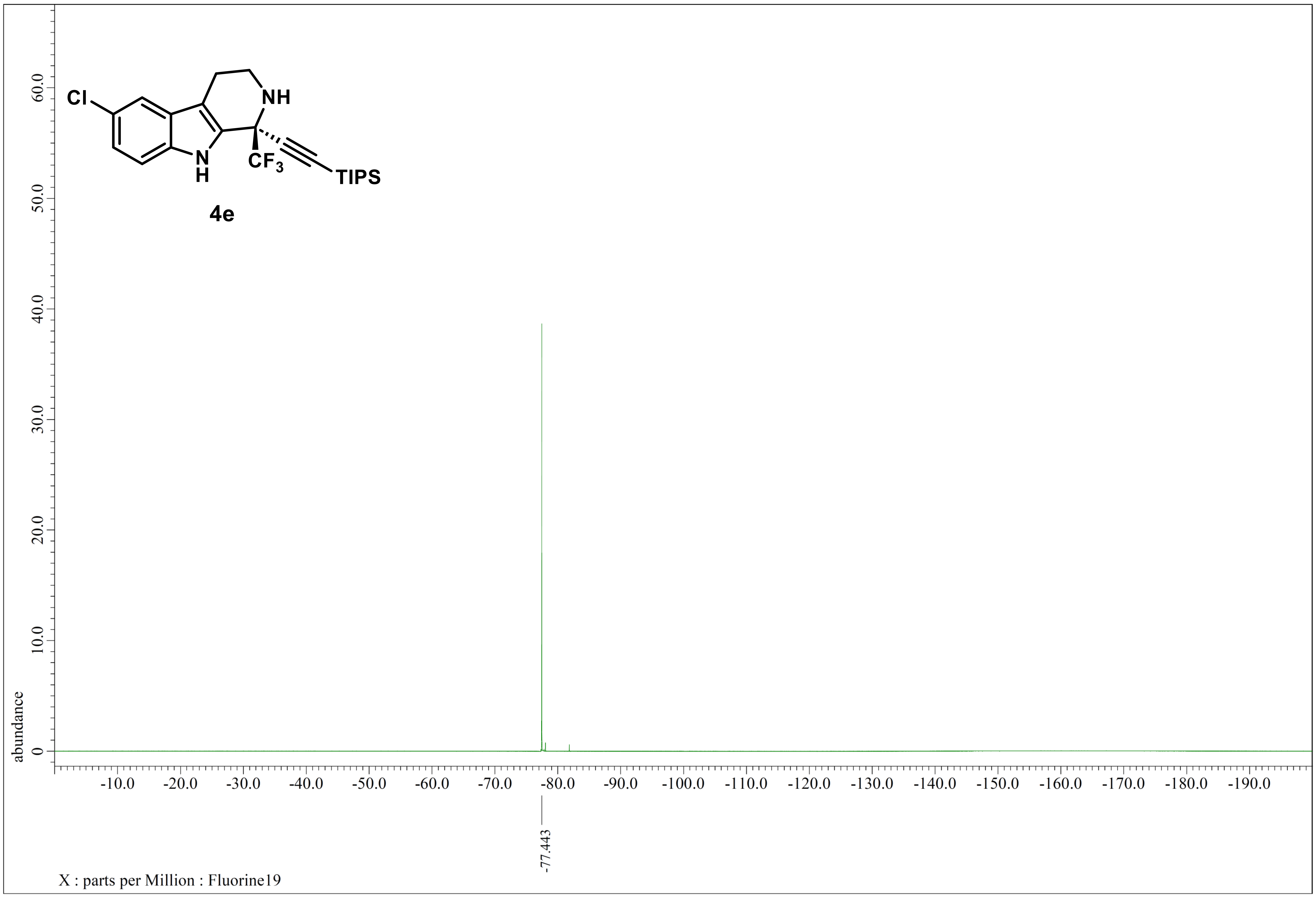

^{1}H NMR (600 MHz, $CDCl_3$), ^{13}C NMR (151 MHz $CDCl_3$) and ^{19}F NMR (565 MHz $CDCl_3$) spectra of **4f**

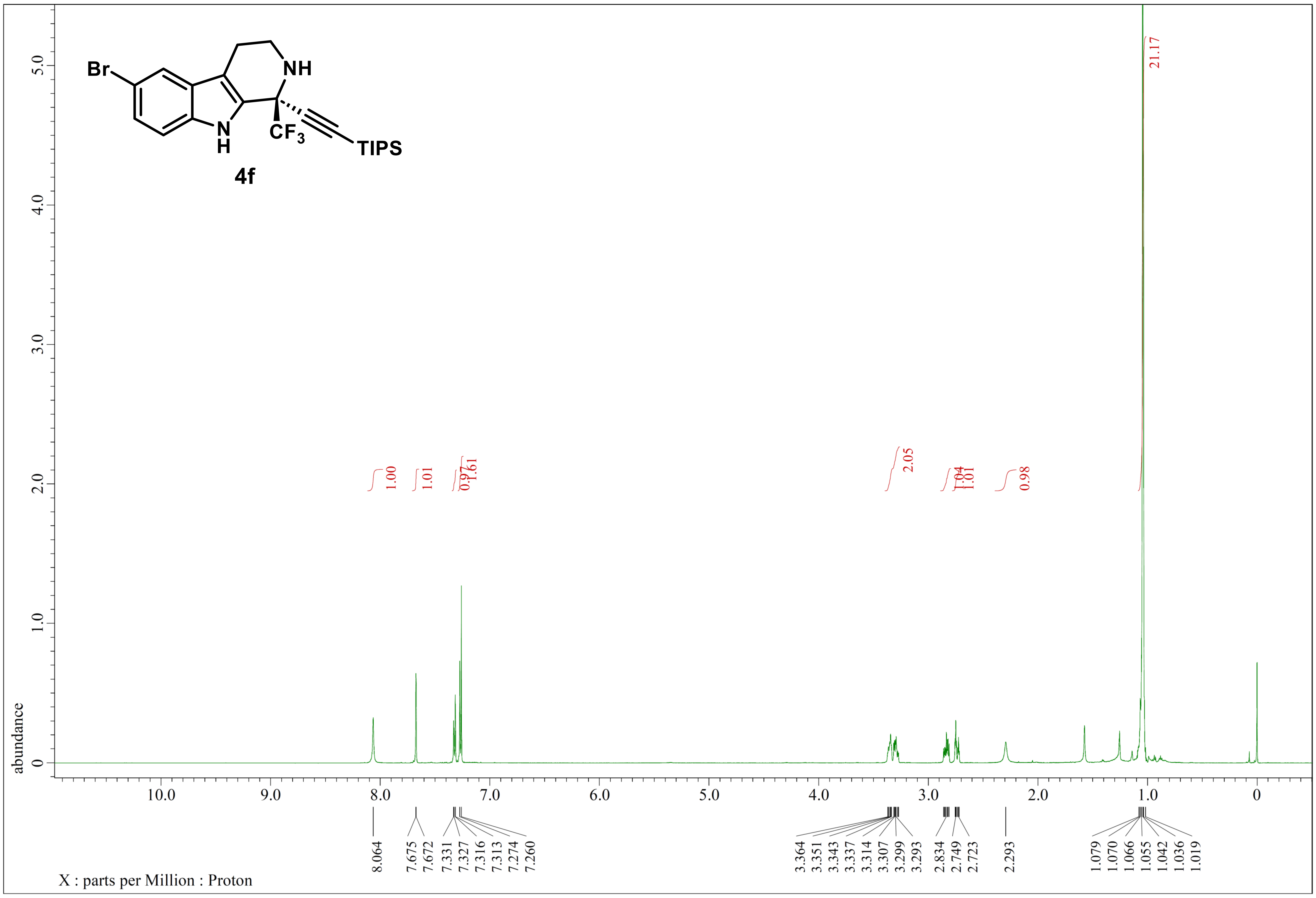

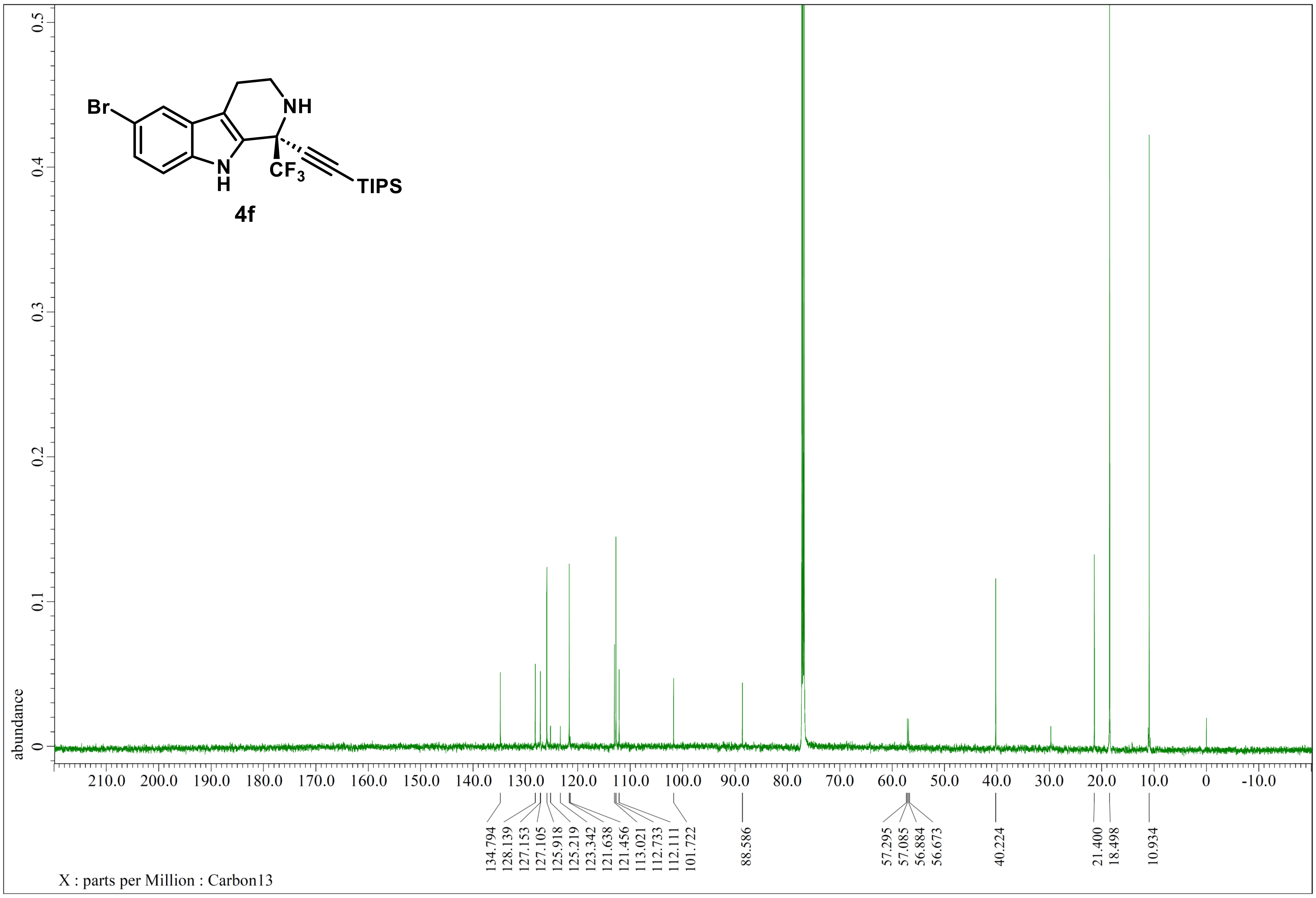
Br
NH
N
H
CF3
TIPS
4f
abundance
X : parts per Million : Carbon13
134.794
128.139
127.153
127.105
125.918
125.219
123.342
121.638
121.456
113.021
112.733
112.111
101.722
88.586
57.295
57.085
56.884
56.673
40.224
21.400
18.498
10.934

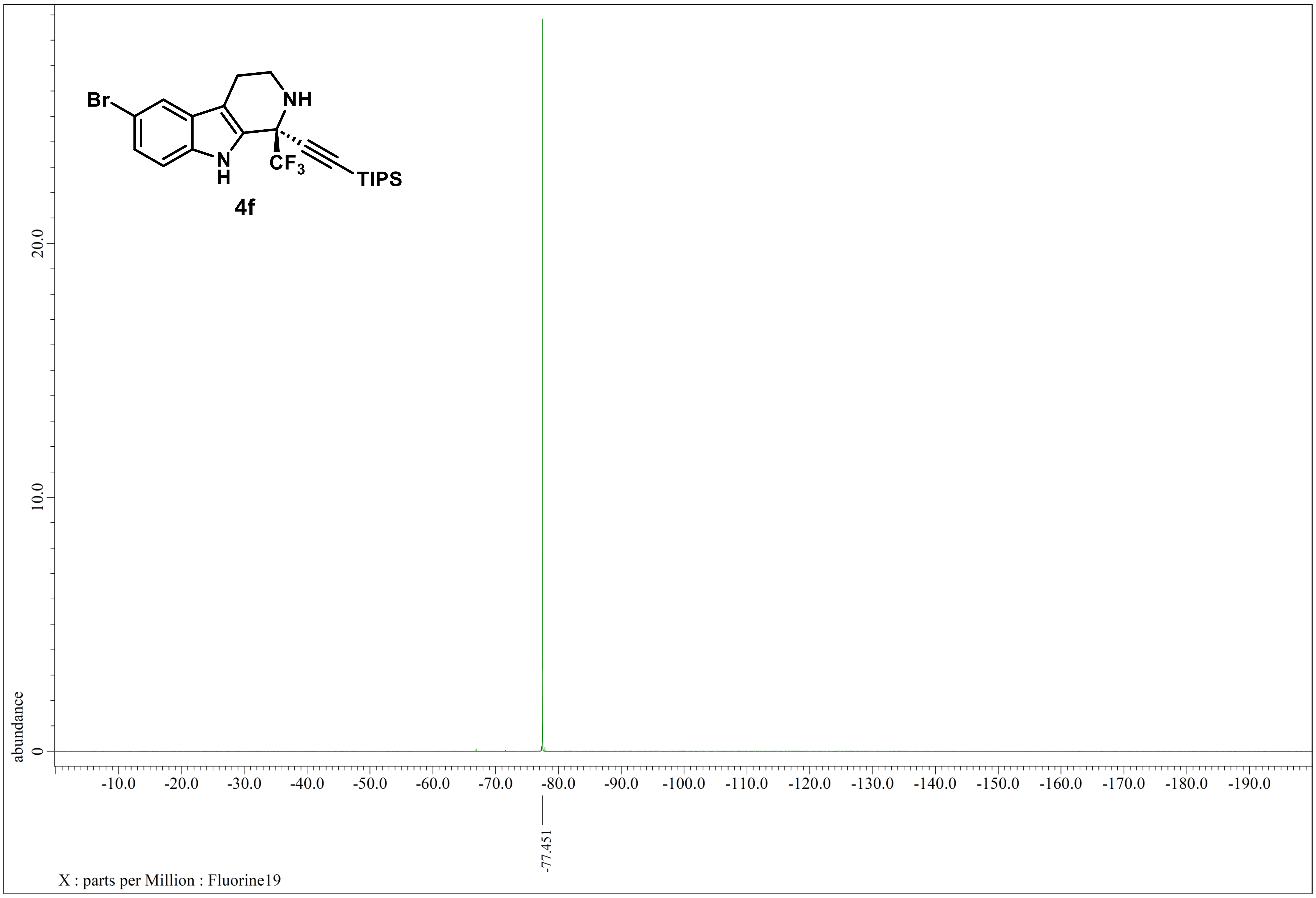
Br
NH
N
H
CF3
TIPS
4f
abundance
X : parts per Million : Fluorine19
-77.451

^{1}H NMR (600 MHz, $CDCl_3$), ^{13}C NMR (151 MHz $CDCl_3$) and ^{19}F NMR (565 MHz $CDCl_3$) spectra of **4g**

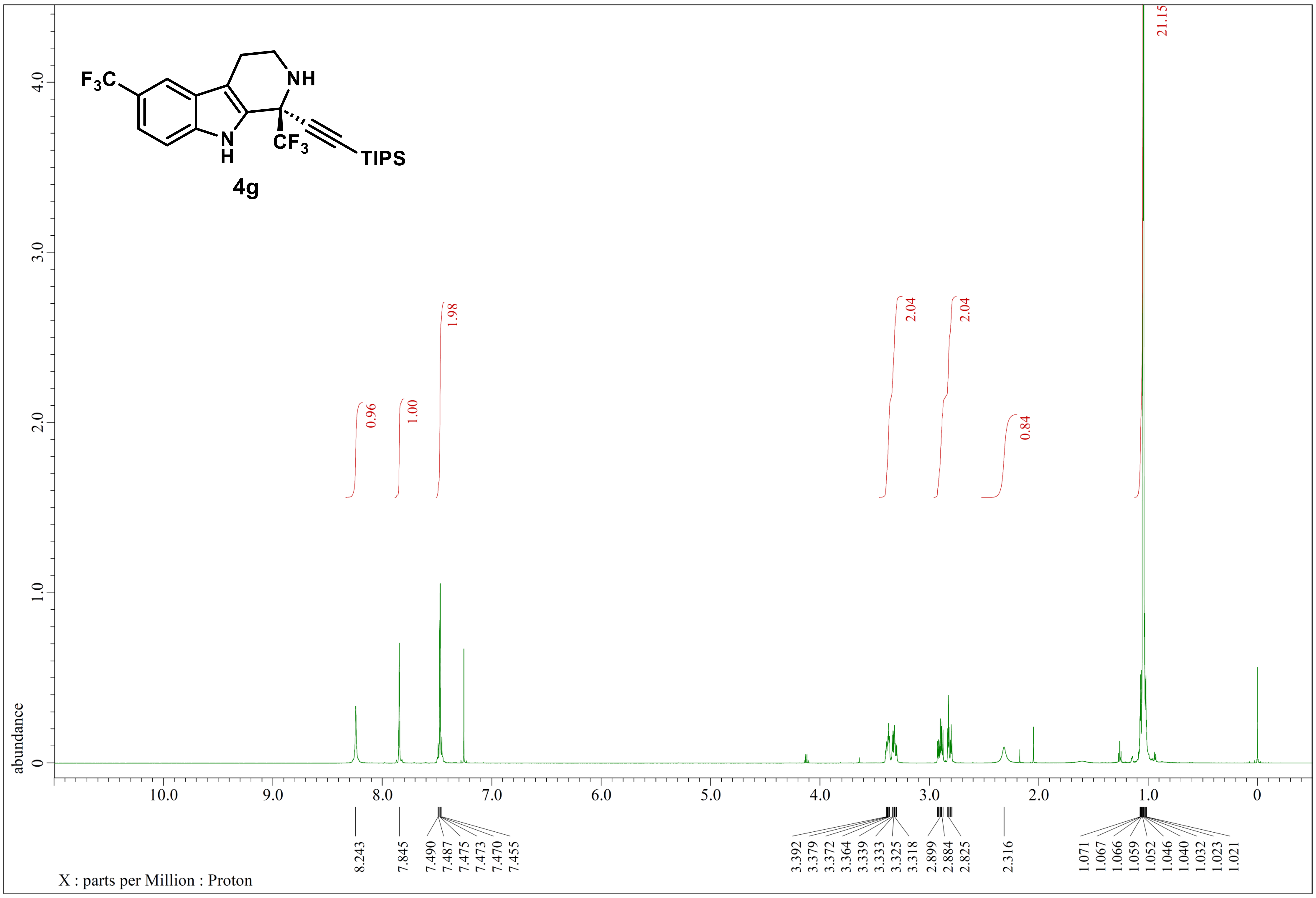

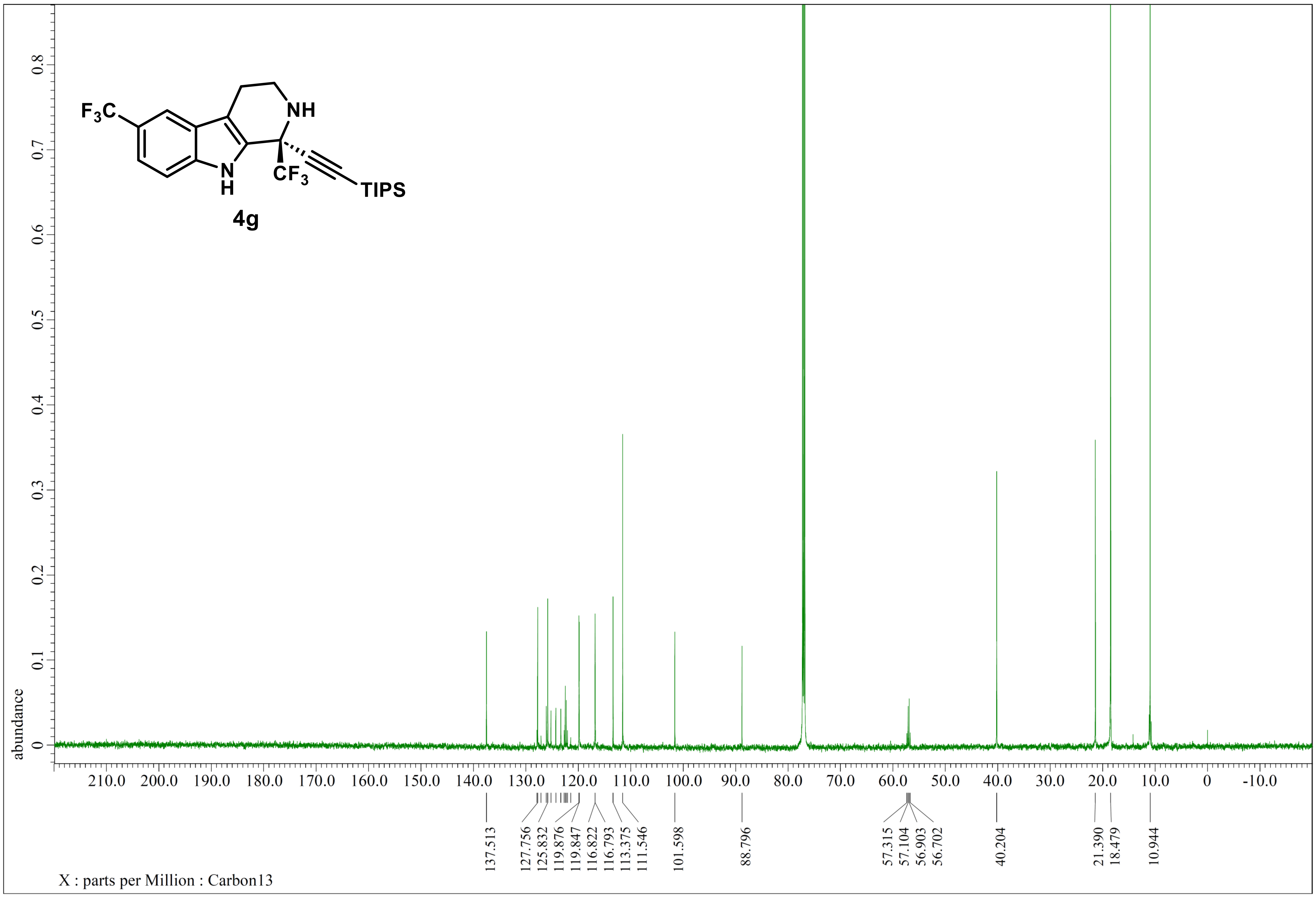

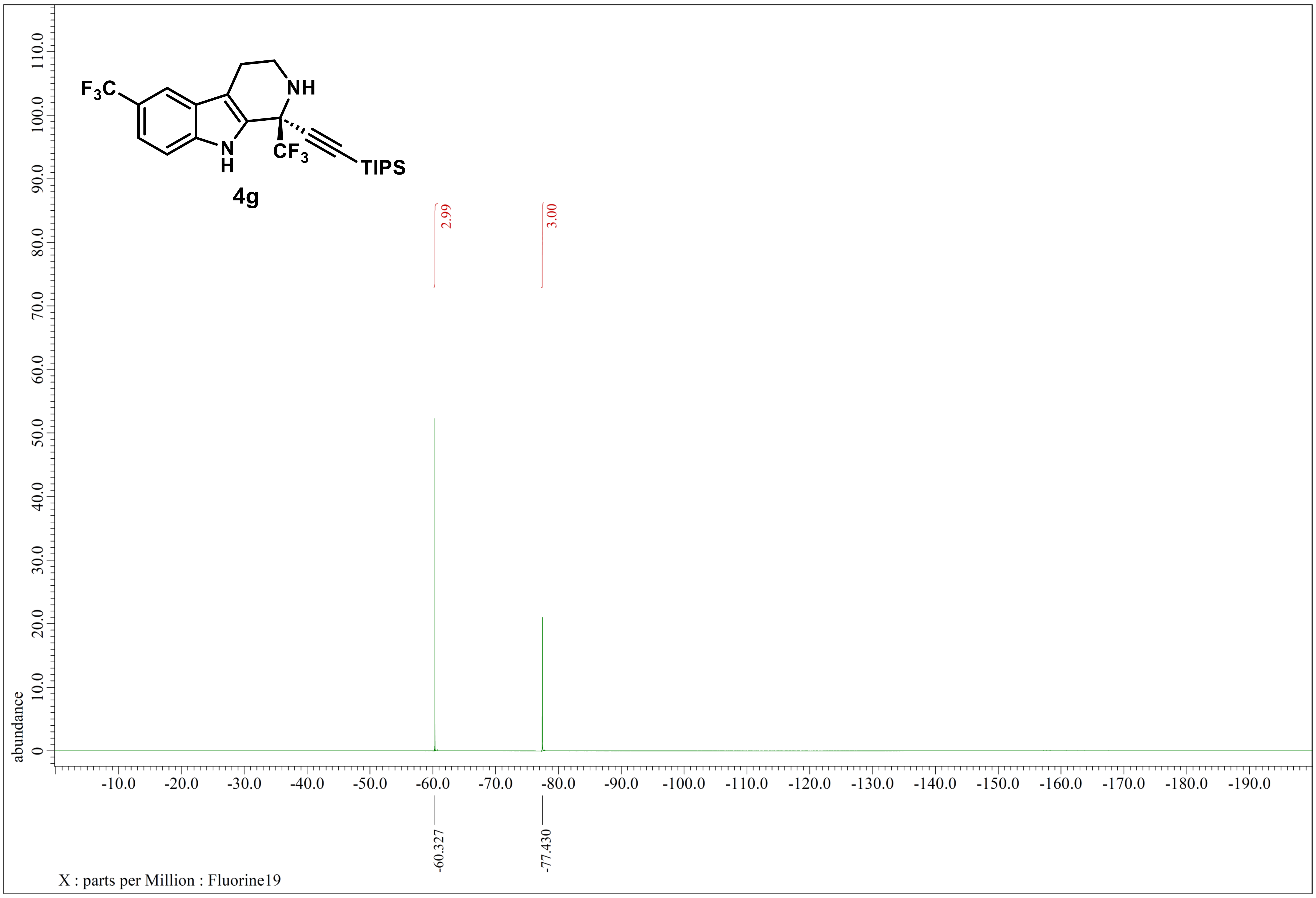

^{1}H NMR (600 MHz, $CDCl_3$), ^{13}C NMR (151 MHz $CDCl_3$) and ^{19}F NMR (565 MHz $CDCl_3$) spectra of **4h**

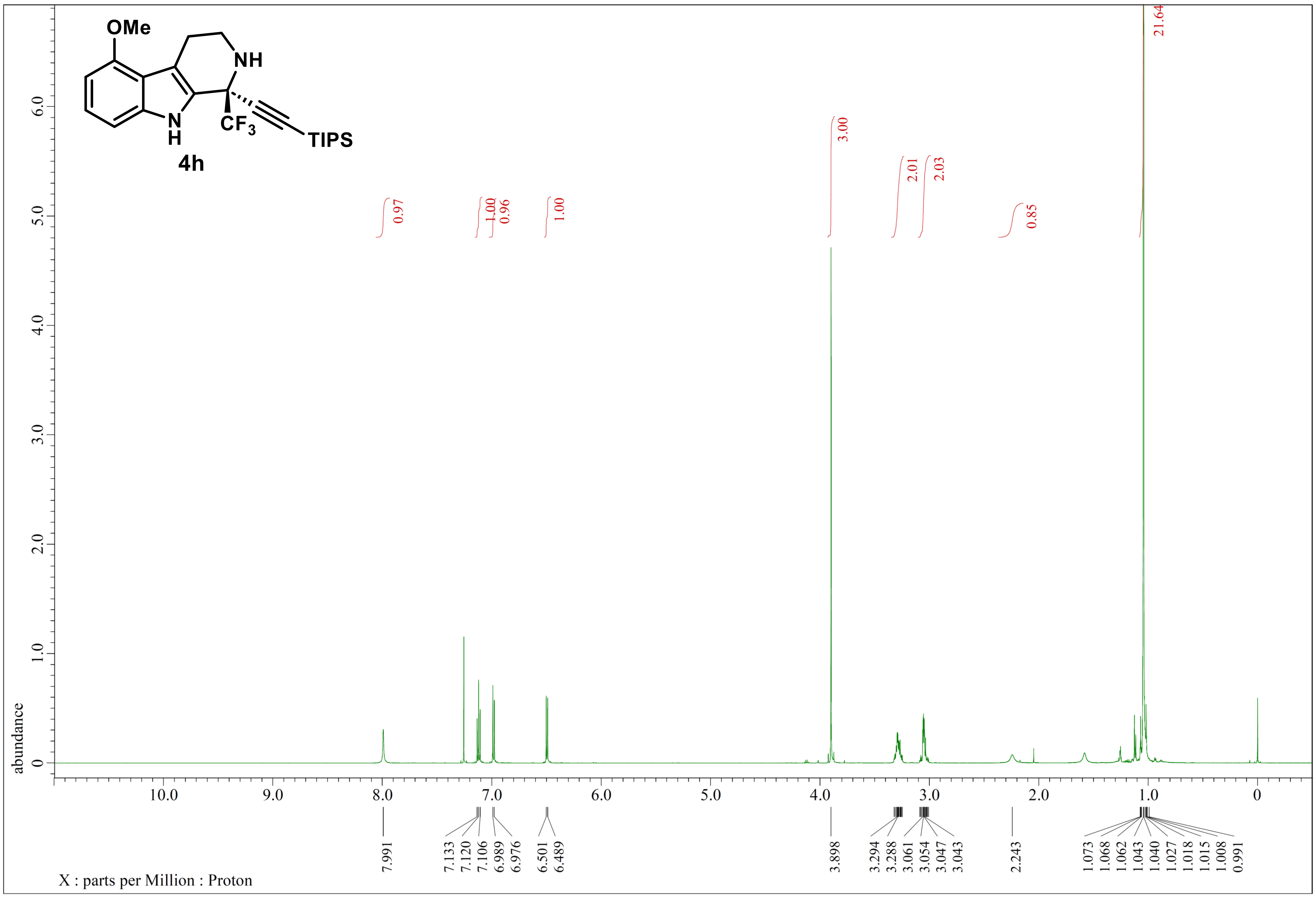

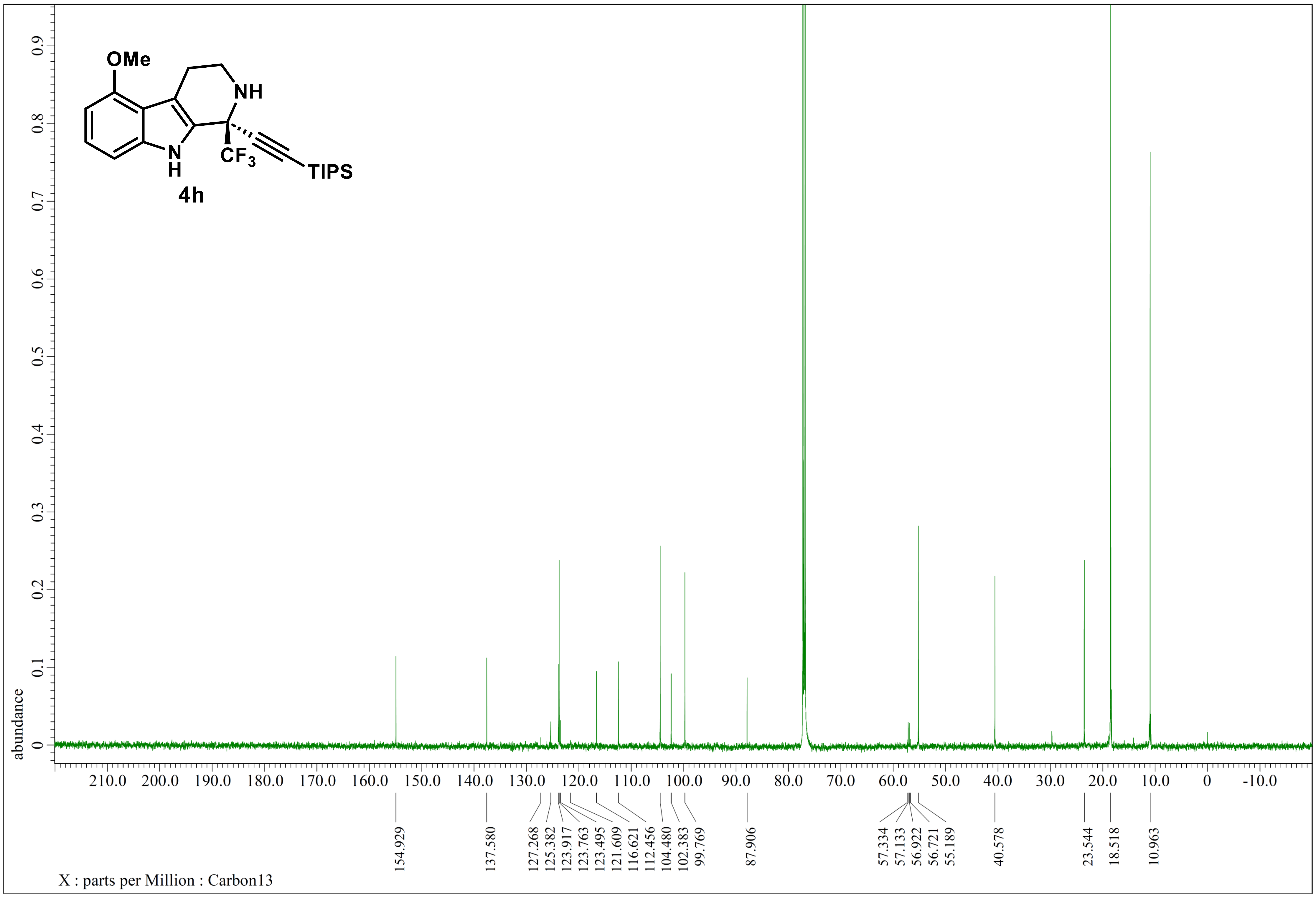
OMe
NH
N
H
CF3
TIPS
4h
abundance
X : parts per Million : Carbon13
154.929
137.580
127.268
125.382
123.917
123.763
123.495
121.609
116.621
112.456
104.480
102.383
99.769
87.906
57.334
57.133
56.922
56.721
55.189
40.578
23.544
18.518
10.963

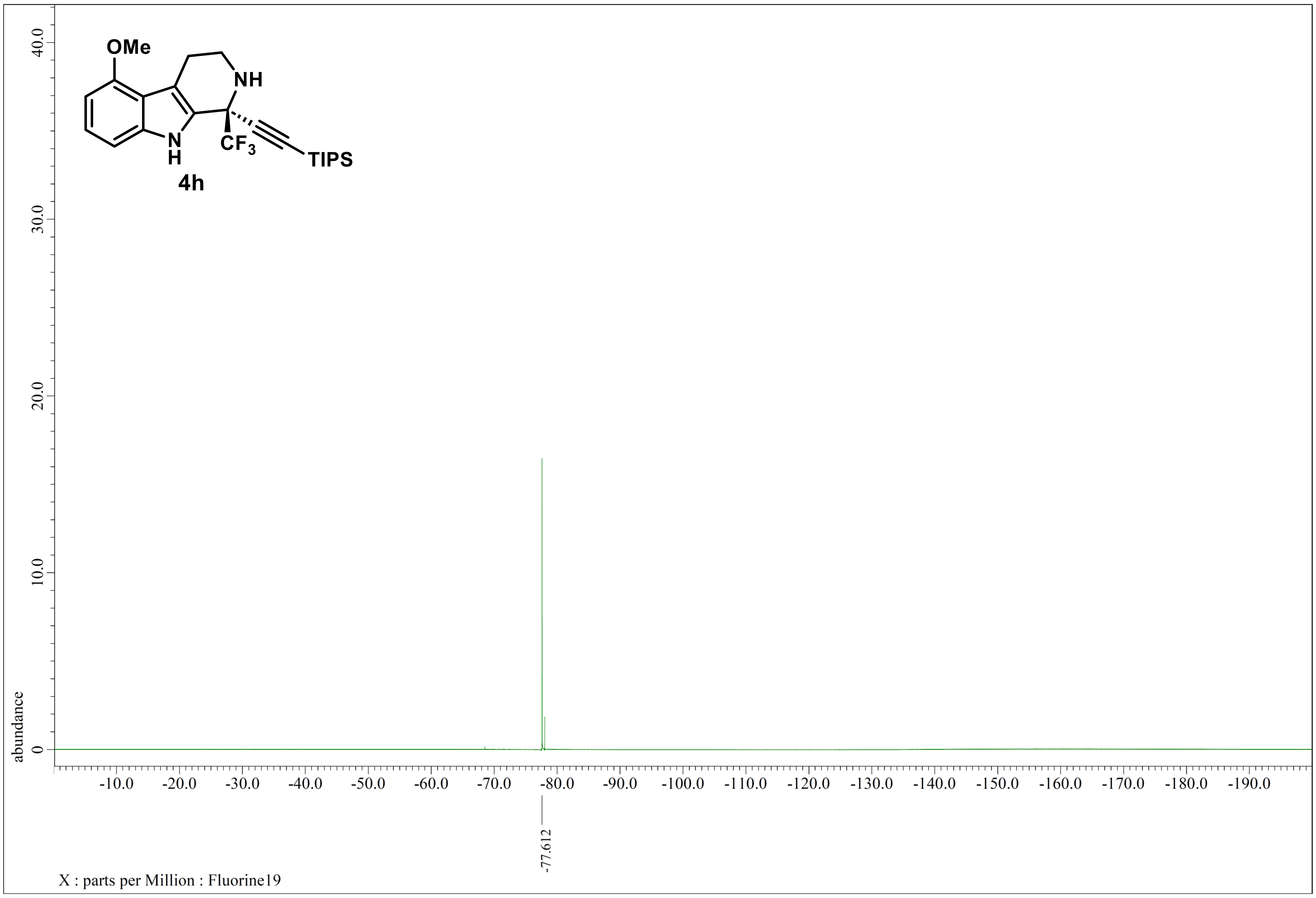
OMe
NH
N
H
CF3
TIPS
4h
abundance
X : parts per Million : Fluorine19
-77.612

^{1}H NMR (600 MHz, $CDCl_3$), ^{13}C NMR (151 MHz $CDCl_3$) and ^{19}F NMR (565 MHz $CDCl_3$) spectra of **4i**

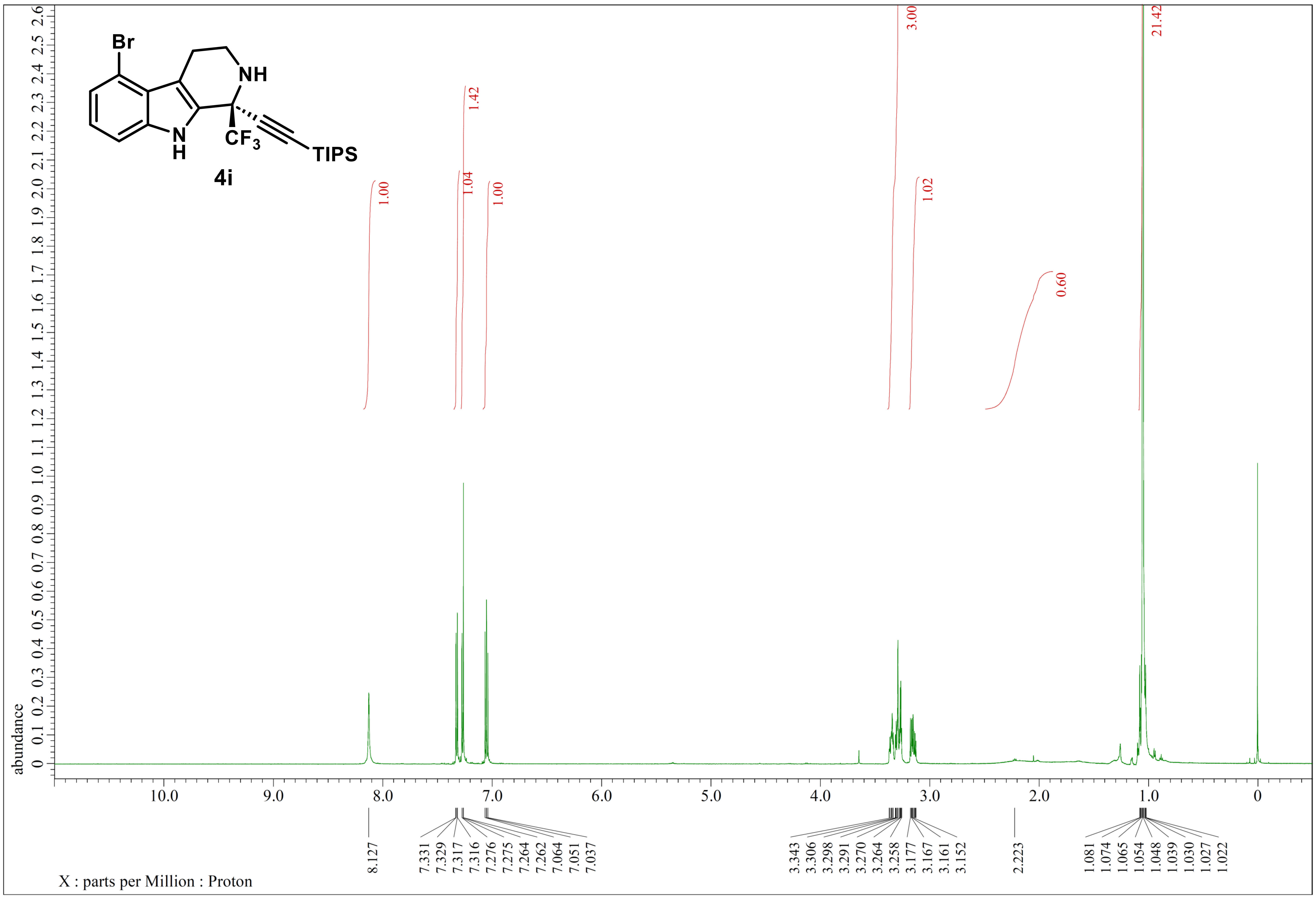

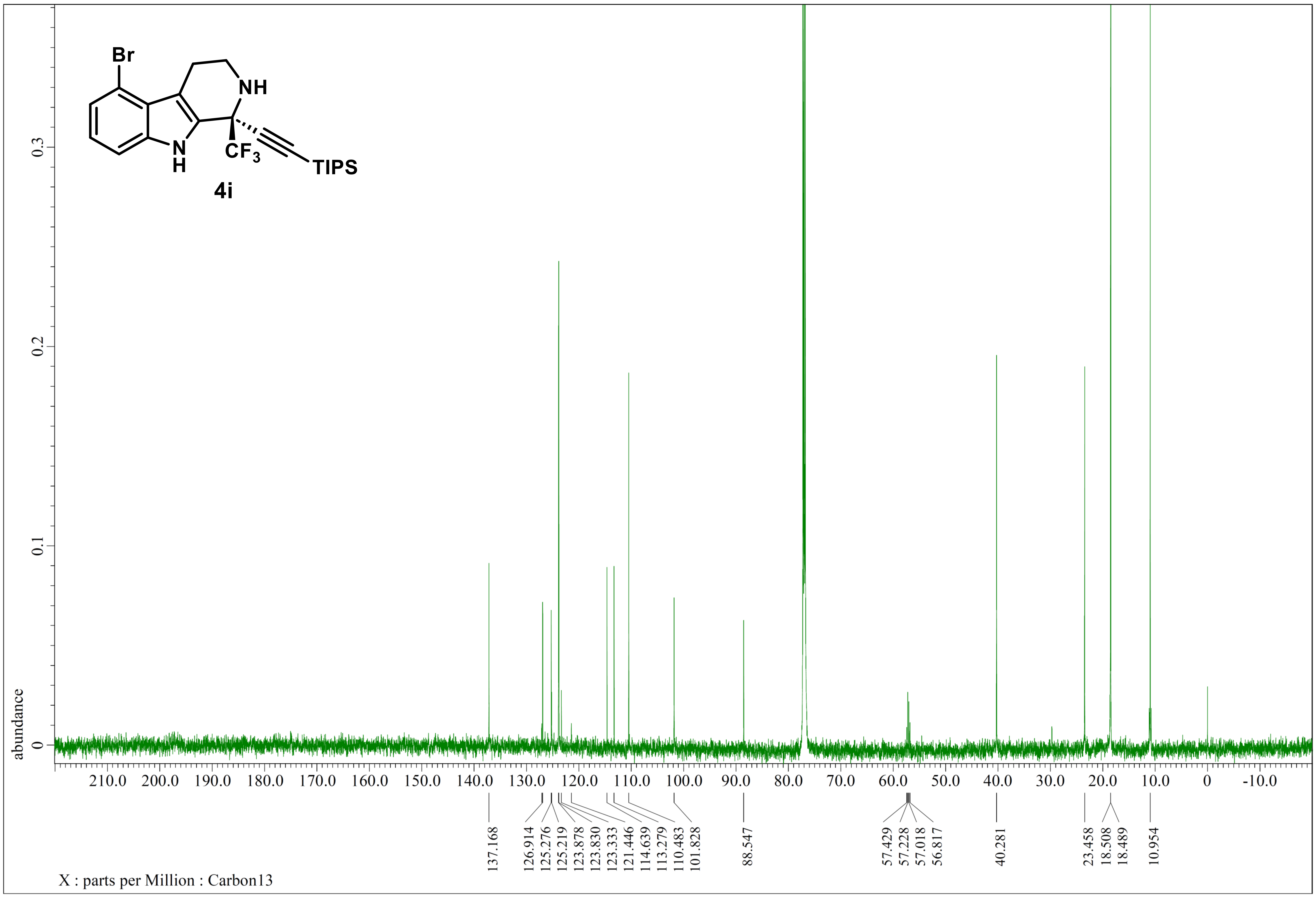

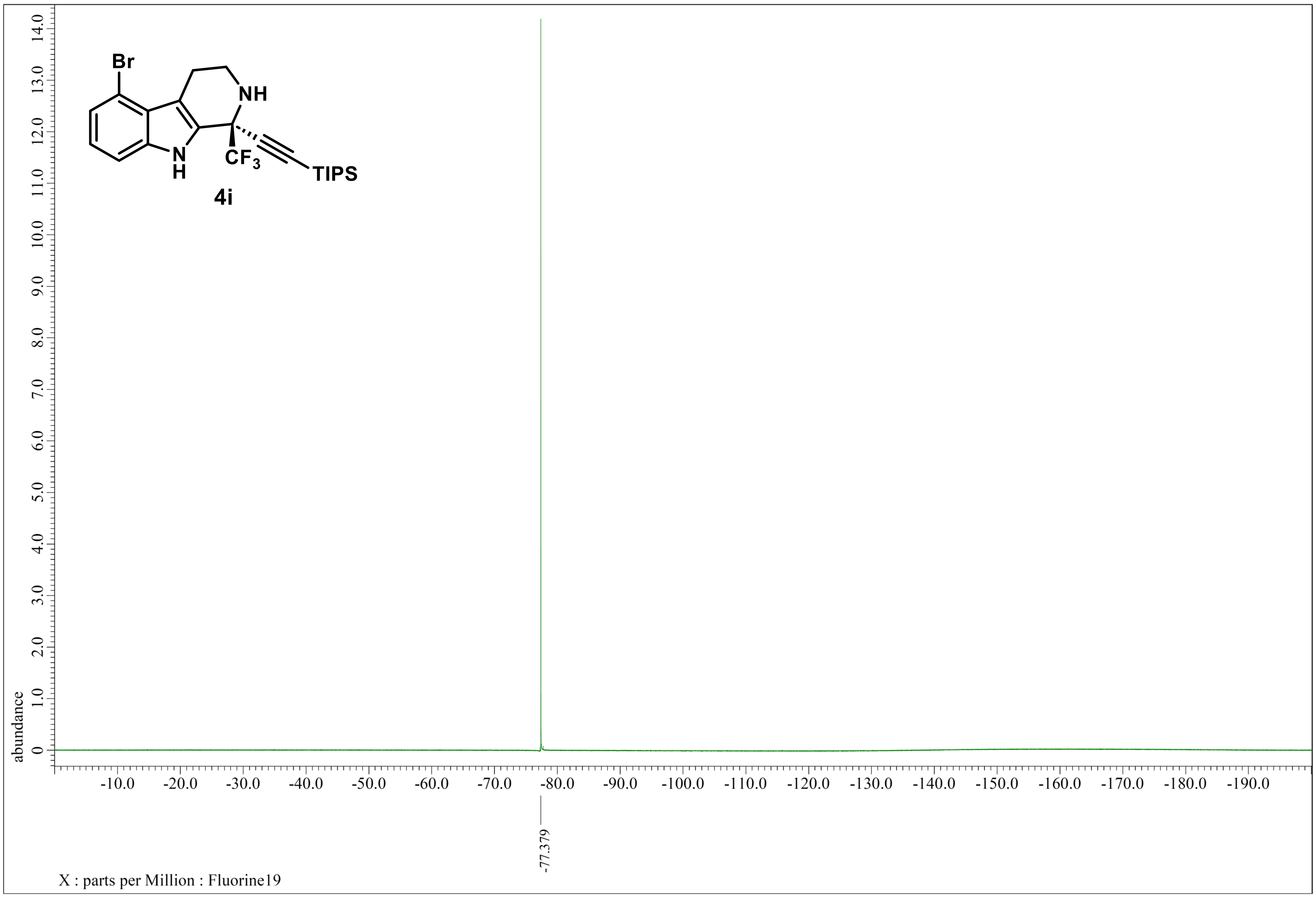

^{1}H NMR (600 MHz, $CDCl_3$), ^{13}C NMR (151 MHz $CDCl_3$) and ^{19}F NMR (565 MHz $CDCl_3$) spectra of **4j**

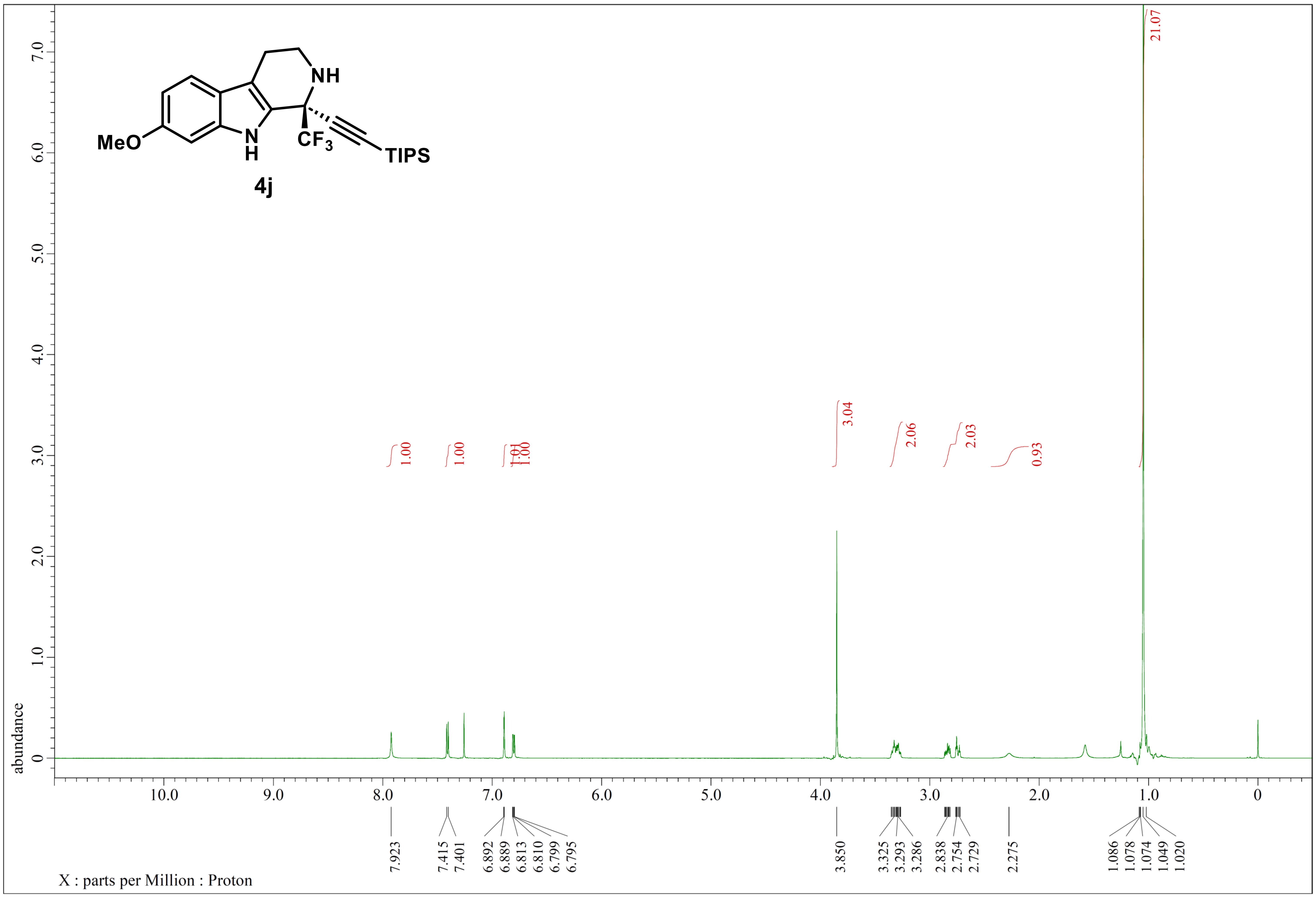

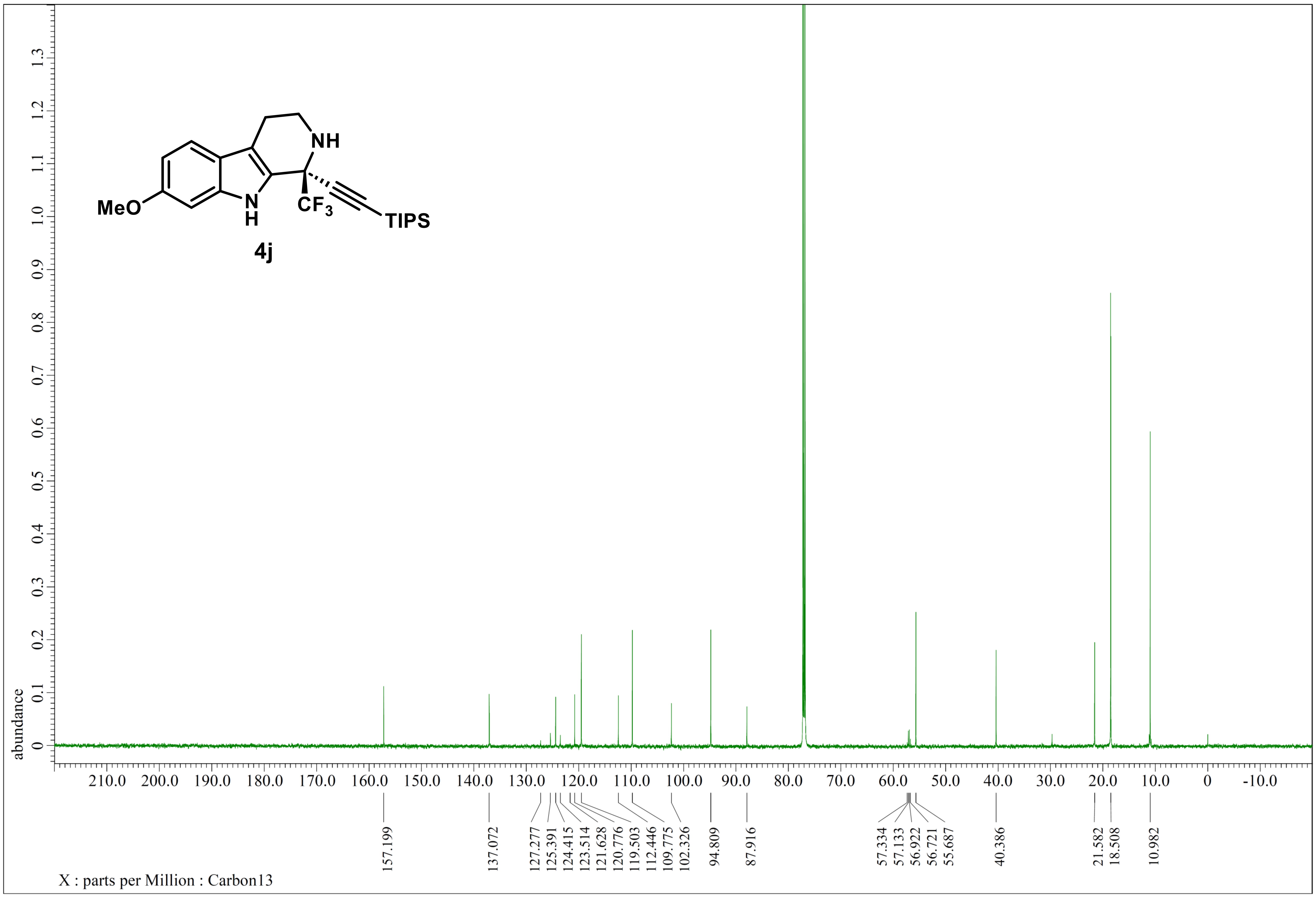
MeO
N
H
NH
CF3
TIPS
4j
abundance
0 0.1 0.2 0.3 0.4 0.5 0.6 0.7 0.8 0.9 1.0 1.1 1.2 1.3
210.0 200.0 190.0 180.0 170.0 160.0 150.0 140.0 130.0 120.0 110.0 100.0 90.0 80.0 70.0 60.0 50.0 40.0 30.0 20.0 10.0 0 -10.0
157.199
137.072
127.277
125.391
124.415
123.514
121.628
120.776
119.503
112.446
109.775
102.326
94.809
87.916
57.334
57.133
56.922
56.721
55.687
40.386
21.582
18.508
10.982
X : parts per Million : Carbon13

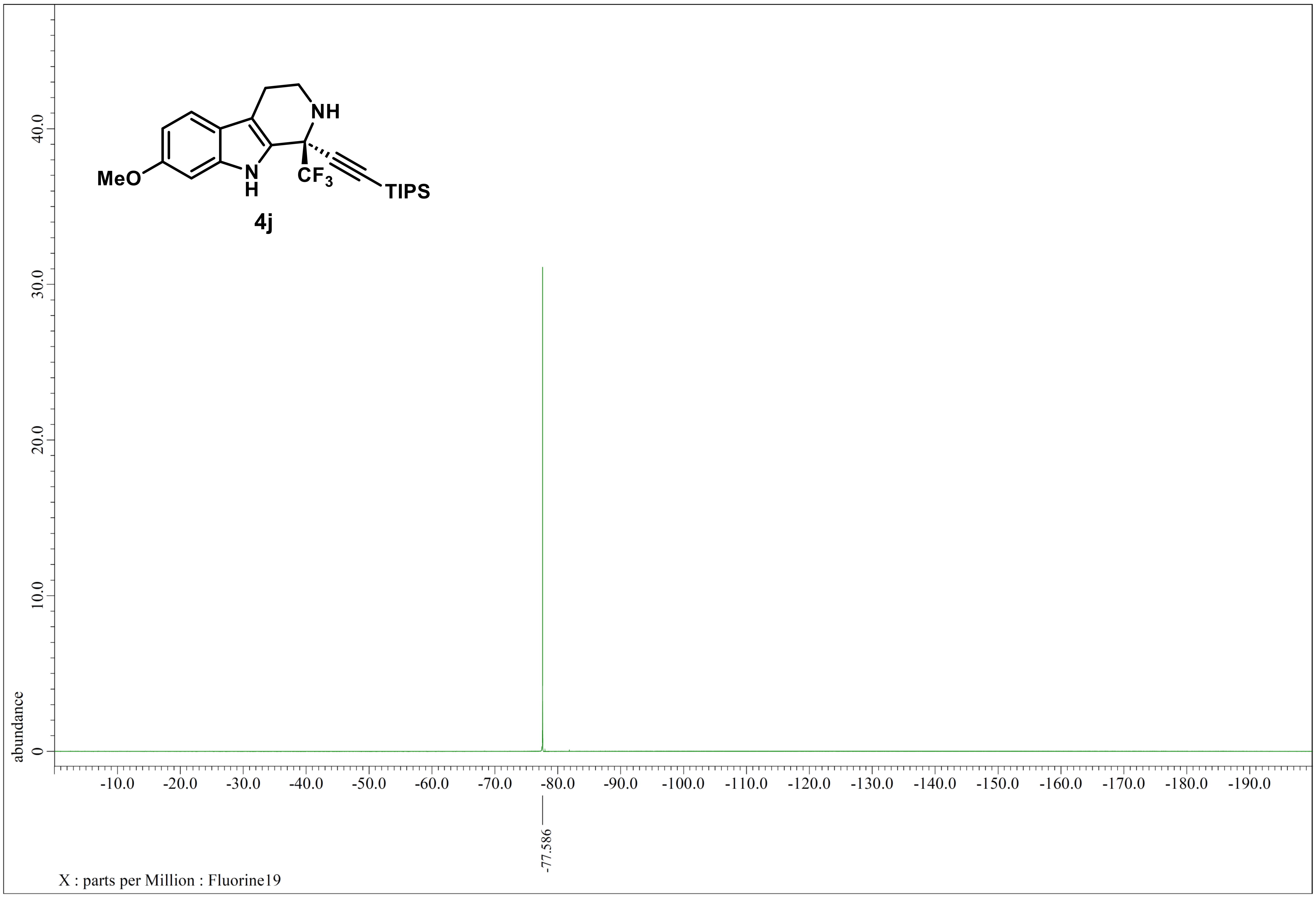
MeO
N
H
NH
CF3
TIPS
4j
abundance
0 10.0 20.0 30.0 40.0
-10.0 -20.0 -30.0 -40.0 -50.0 -60.0 -70.0 -80.0 -90.0 -100.0 -110.0 -120.0 -130.0 -140.0 -150.0 -160.0 -170.0 -180.0 -190.0
-77.586
X : parts per Million : Fluorine19

^{1}H NMR (600 MHz, $CDCl_3$), ^{13}C NMR (151 MHz $CDCl_3$) and ^{19}F NMR (565 MHz $CDCl_3$) spectra of **4k**

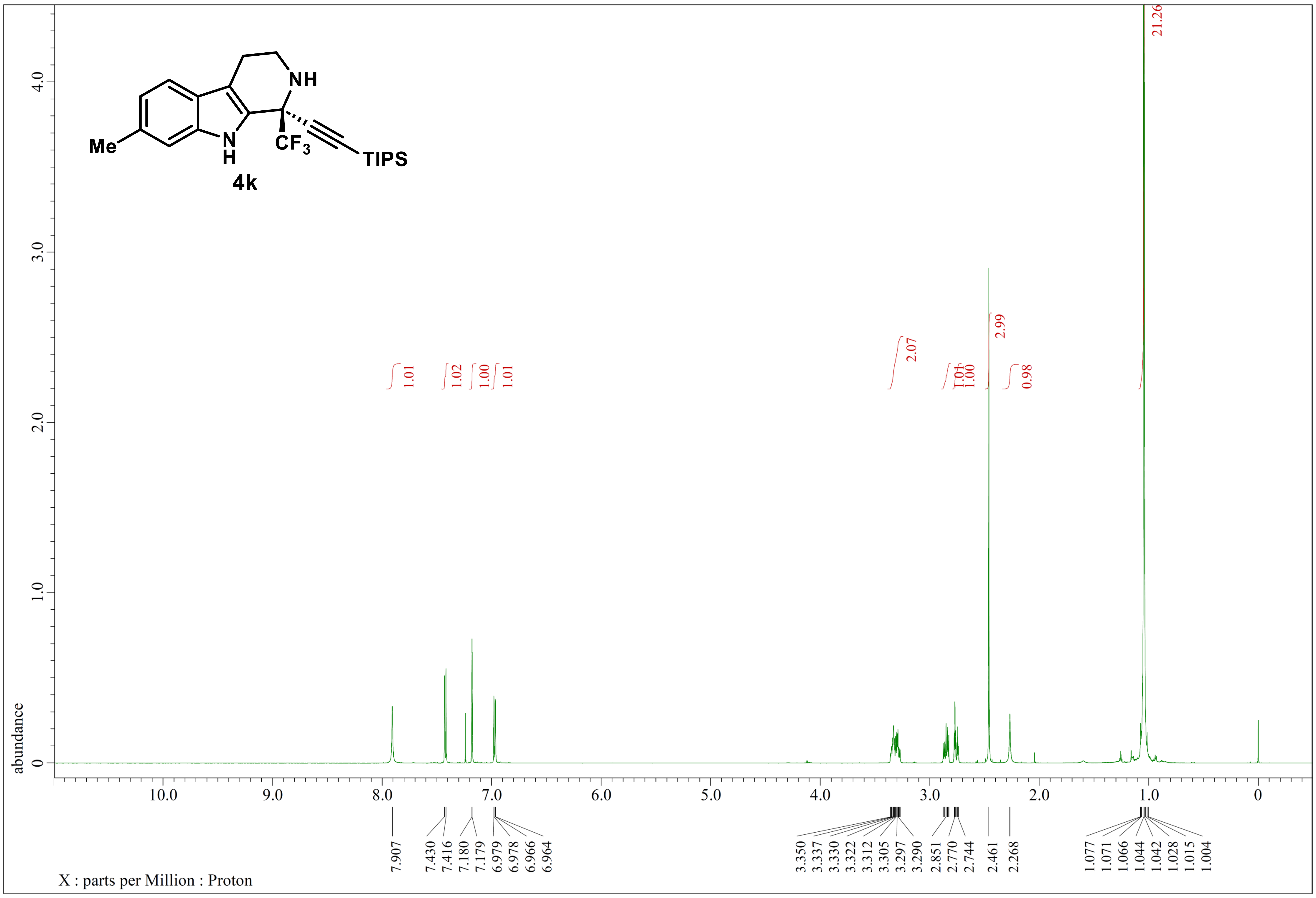

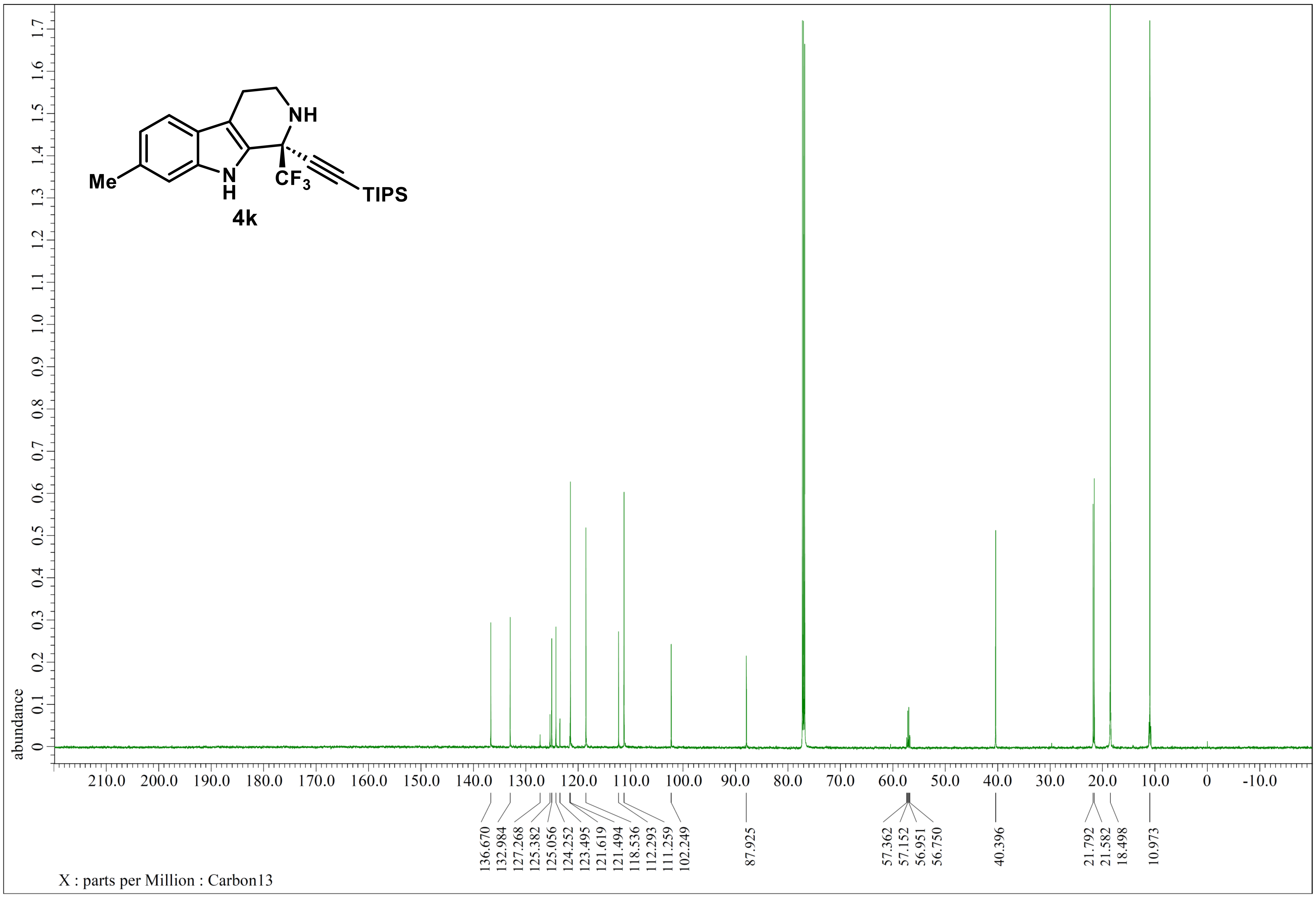

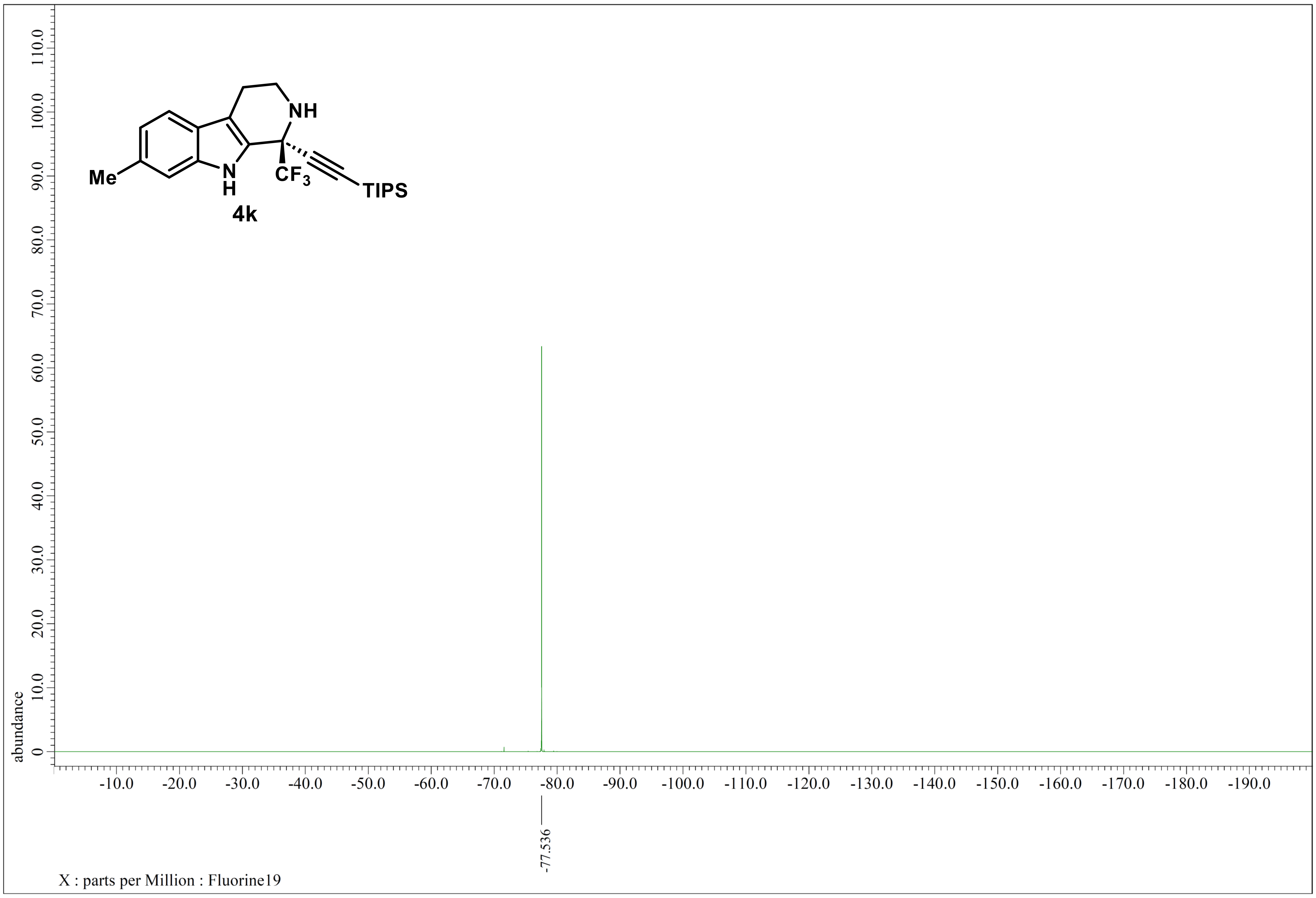

^{1}H NMR (600 MHz, $CDCl_3$), ^{13}C NMR (151 MHz $CDCl_3$) and ^{19}F NMR (565 MHz $CDCl_3$) spectra of **4l**

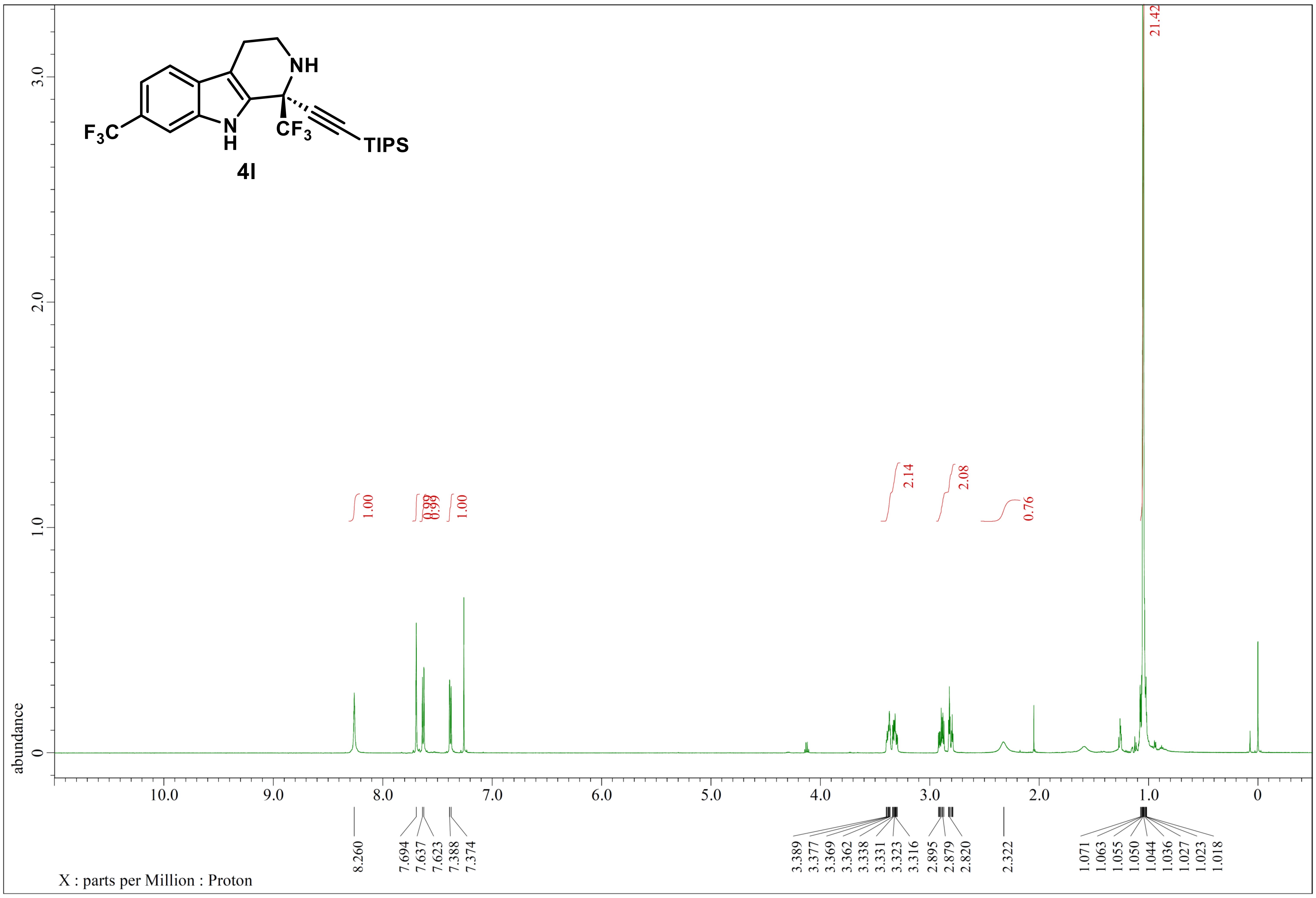

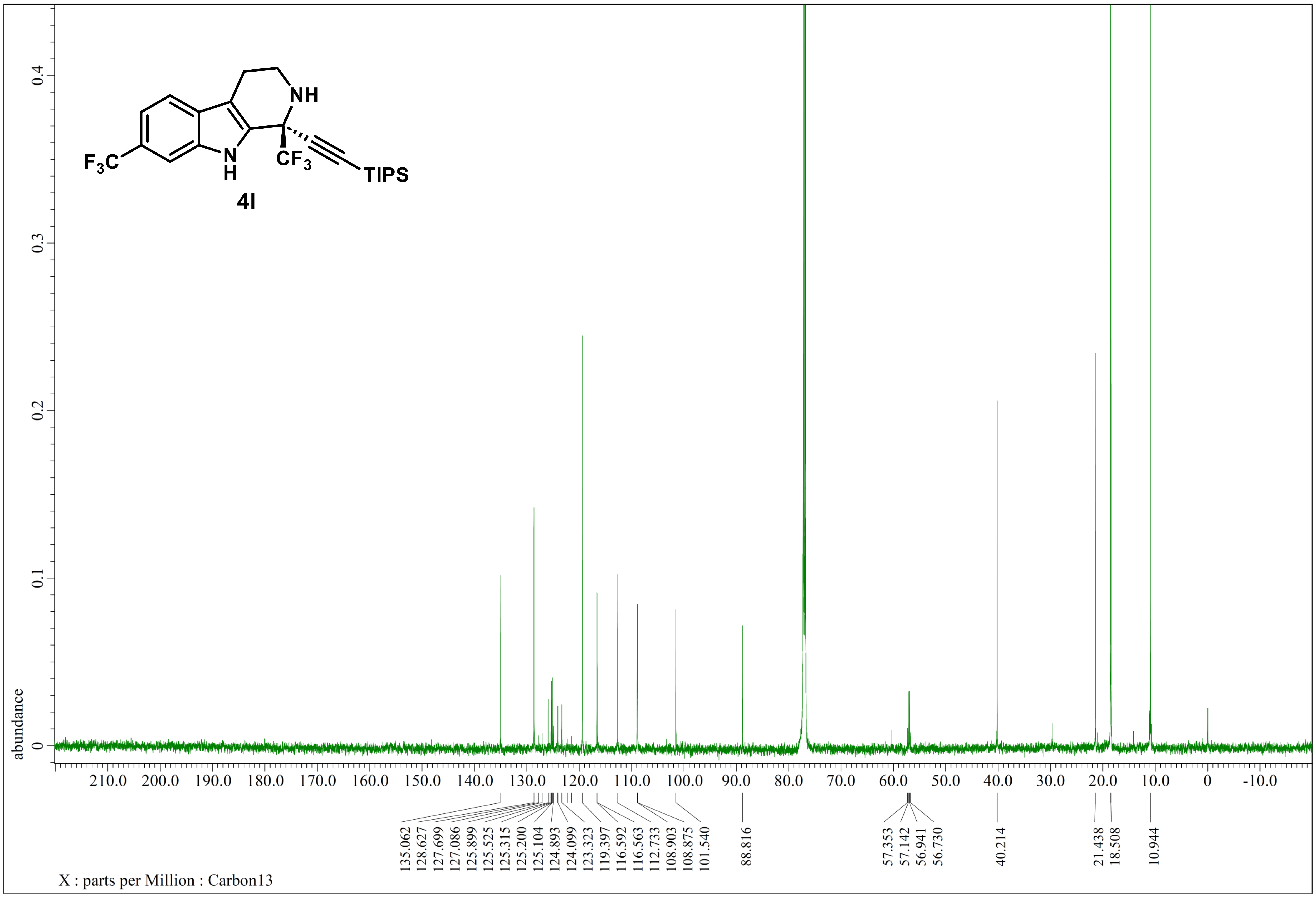
F3C
NH
N
H
CF3
TIPS
4l
abundance
X : parts per Million : Carbon13
135.062
128.627
127.699
127.086
125.899
125.525
125.315
125.200
125.104
124.893
124.099
123.323
119.397
116.592
116.563
112.733
108.903
108.875
101.540
88.816
57.353
57.142
56.941
56.730
40.214
21.438
18.508
10.944

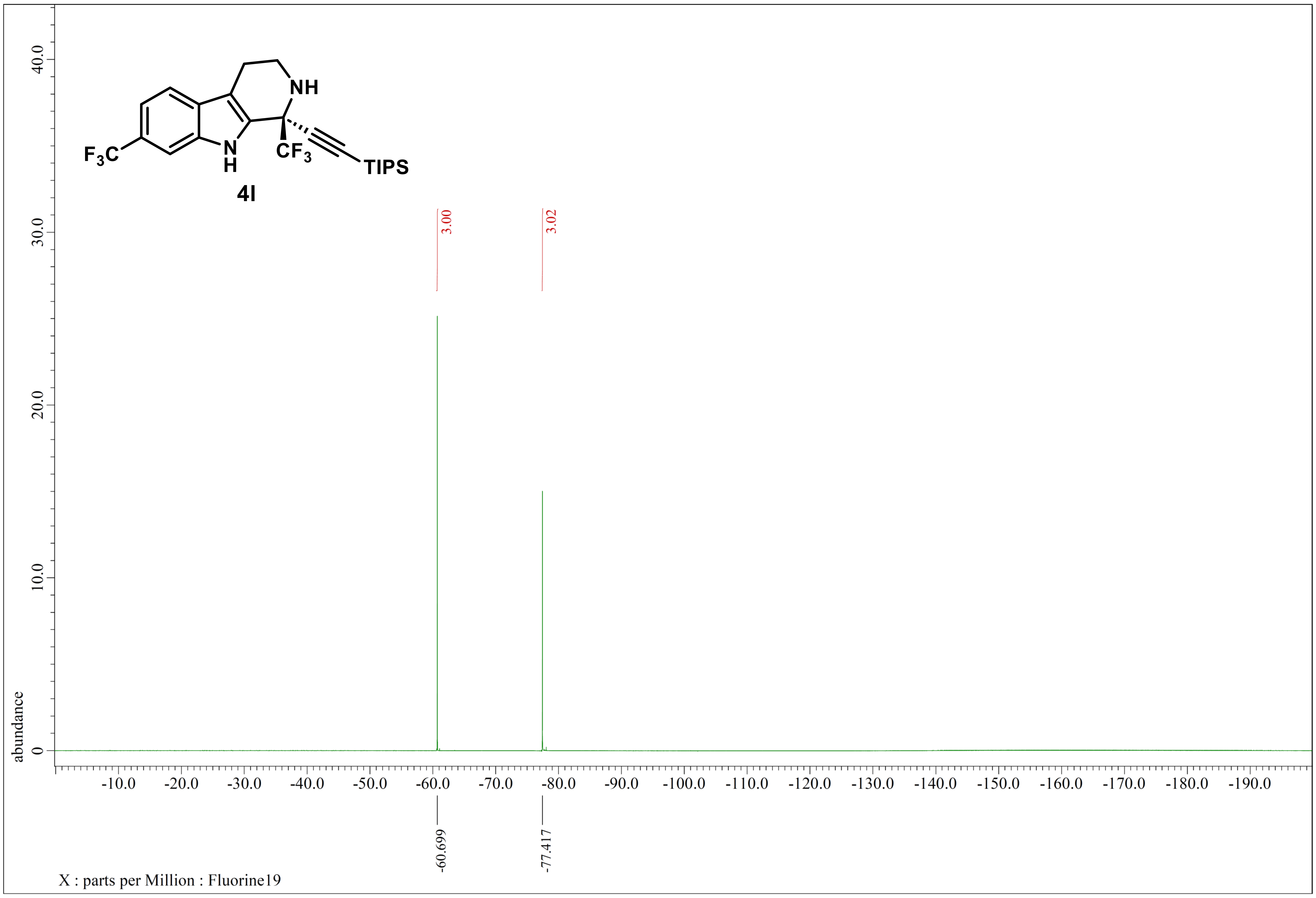
F3C
NH
N
H
CF3
TIPS
4l
3.00
3.02
abundance
X : parts per Million : Fluorine19
-60.699
-77.417

^{1}H NMR (600 MHz, $CDCl_3$), ^{13}C NMR (151 MHz $CDCl_3$) and ^{19}F NMR (565 MHz $CDCl_3$) spectra of **4m**

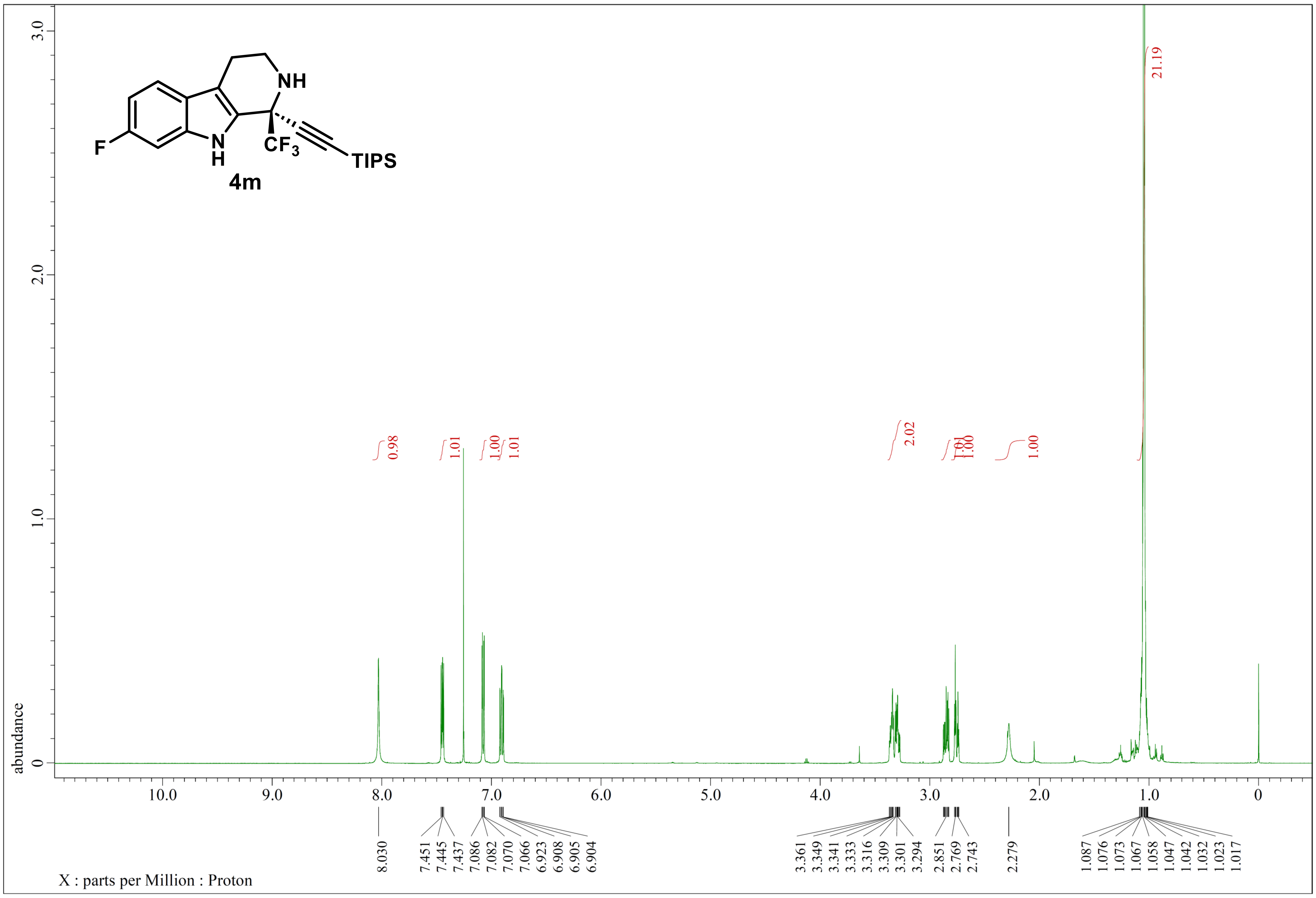

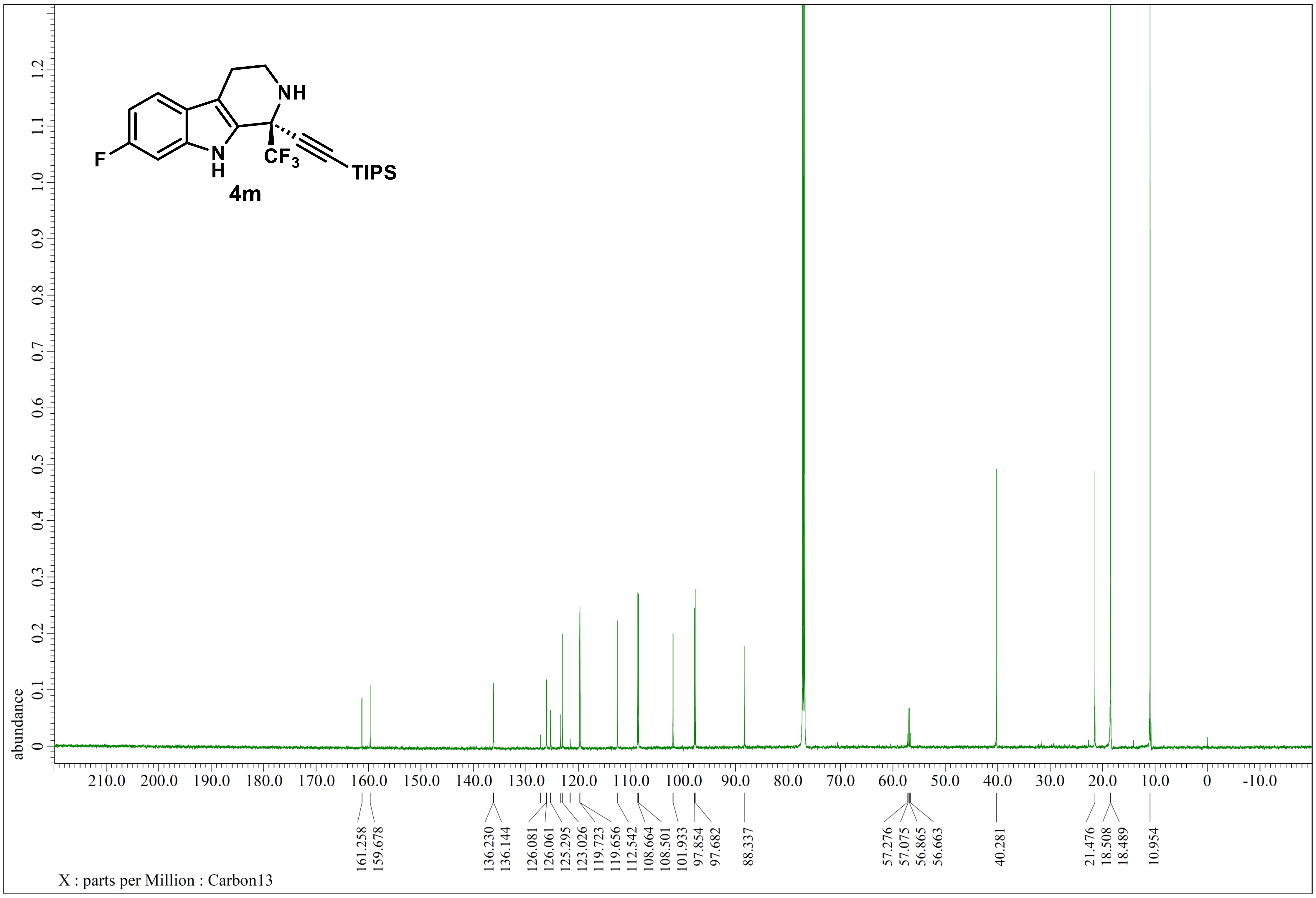

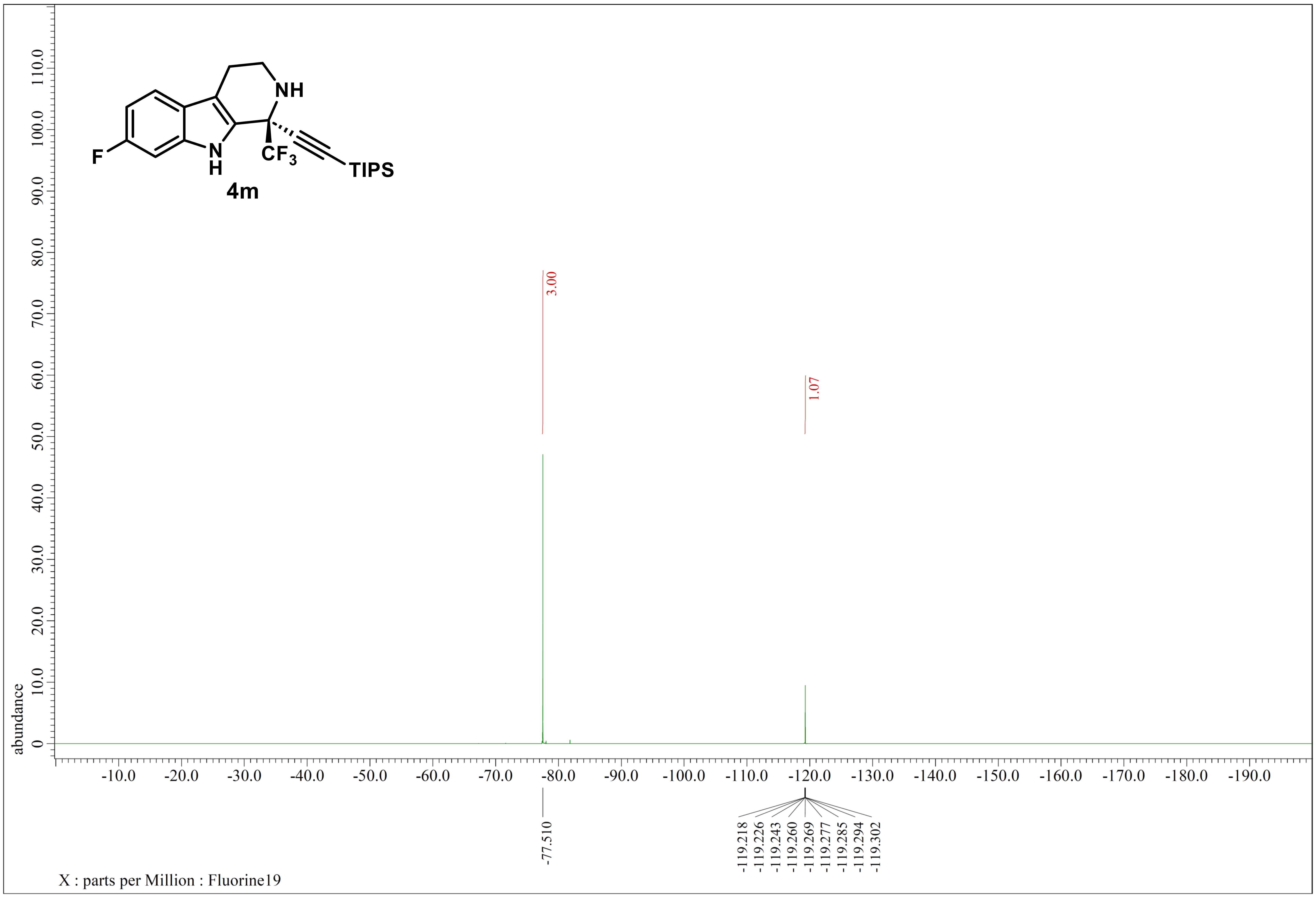

^1H NMR (600 MHz, $CDCl_3$), ^{13}C NMR (151 MHz $CDCl_3$) and ^{19}F NMR (565 MHz $CDCl_3$) spectra of **4n**

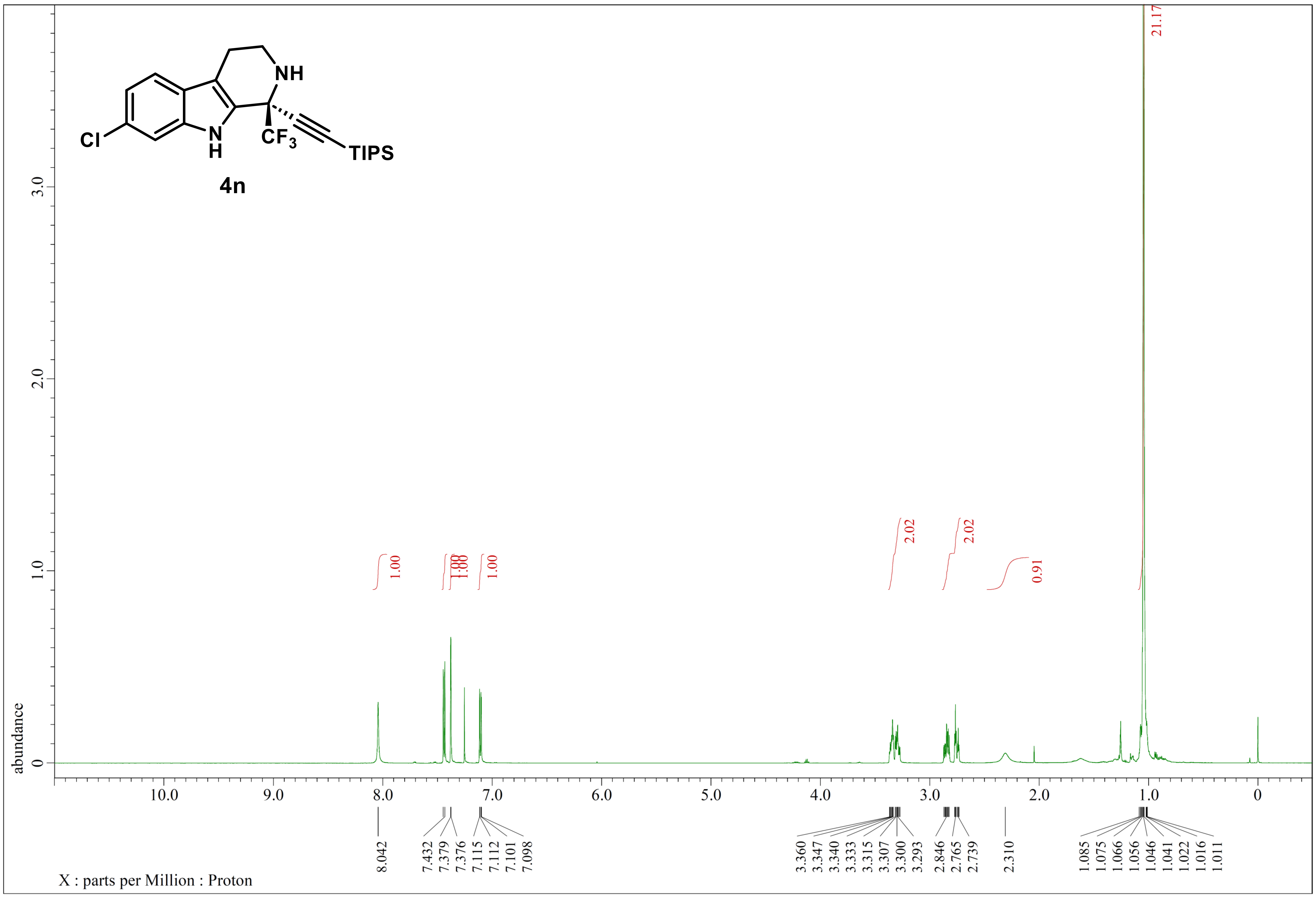

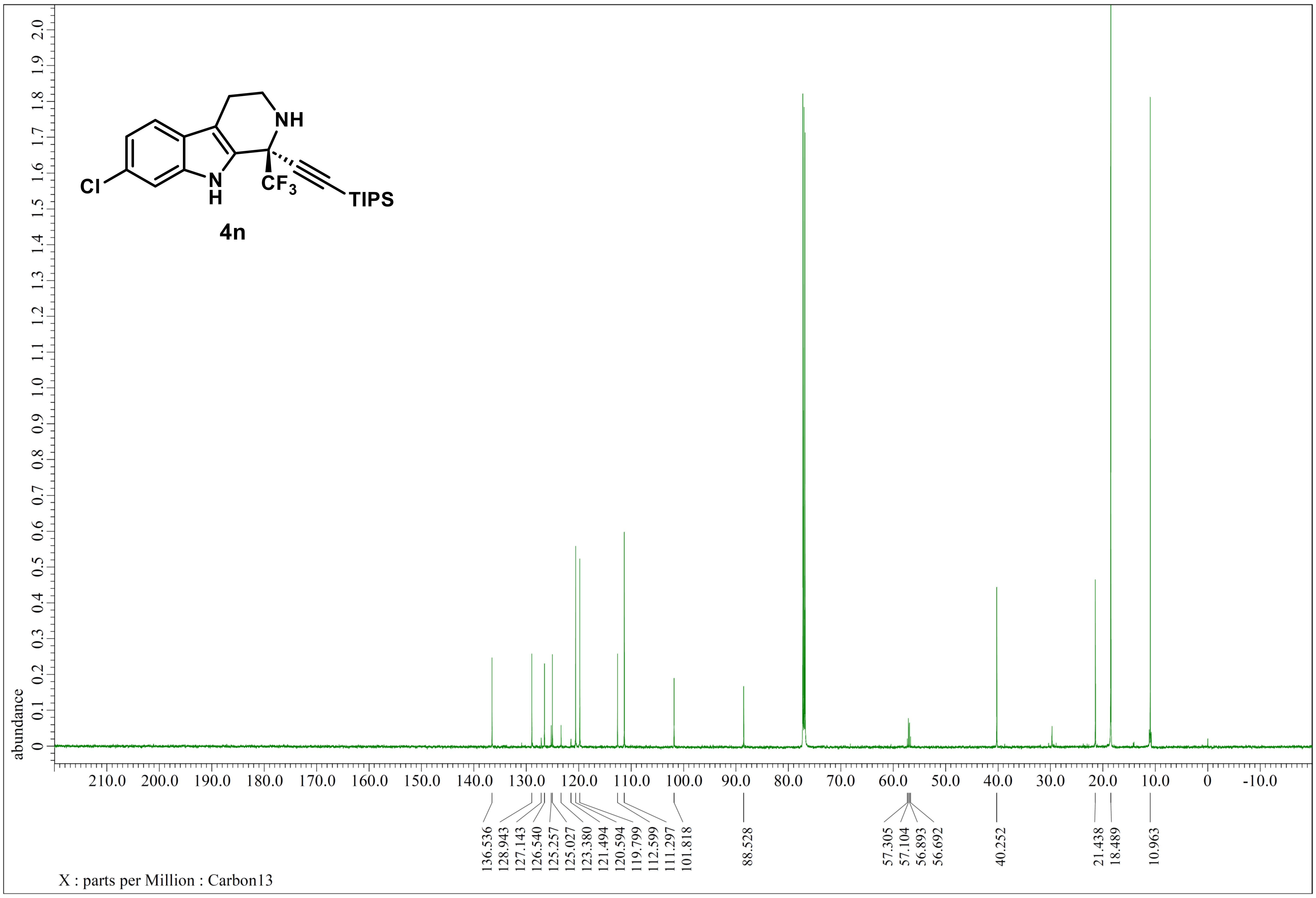
NH
Cl
N
H
CF3
TIPS
4n
abundance
X : parts per Million : Carbon13
136.536
128.943
127.143
126.540
125.257
125.027
123.380
121.494
120.594
119.799
112.599
111.297
101.818
88.528
57.305
57.104
56.893
56.692
40.252
21.438
18.489
10.963

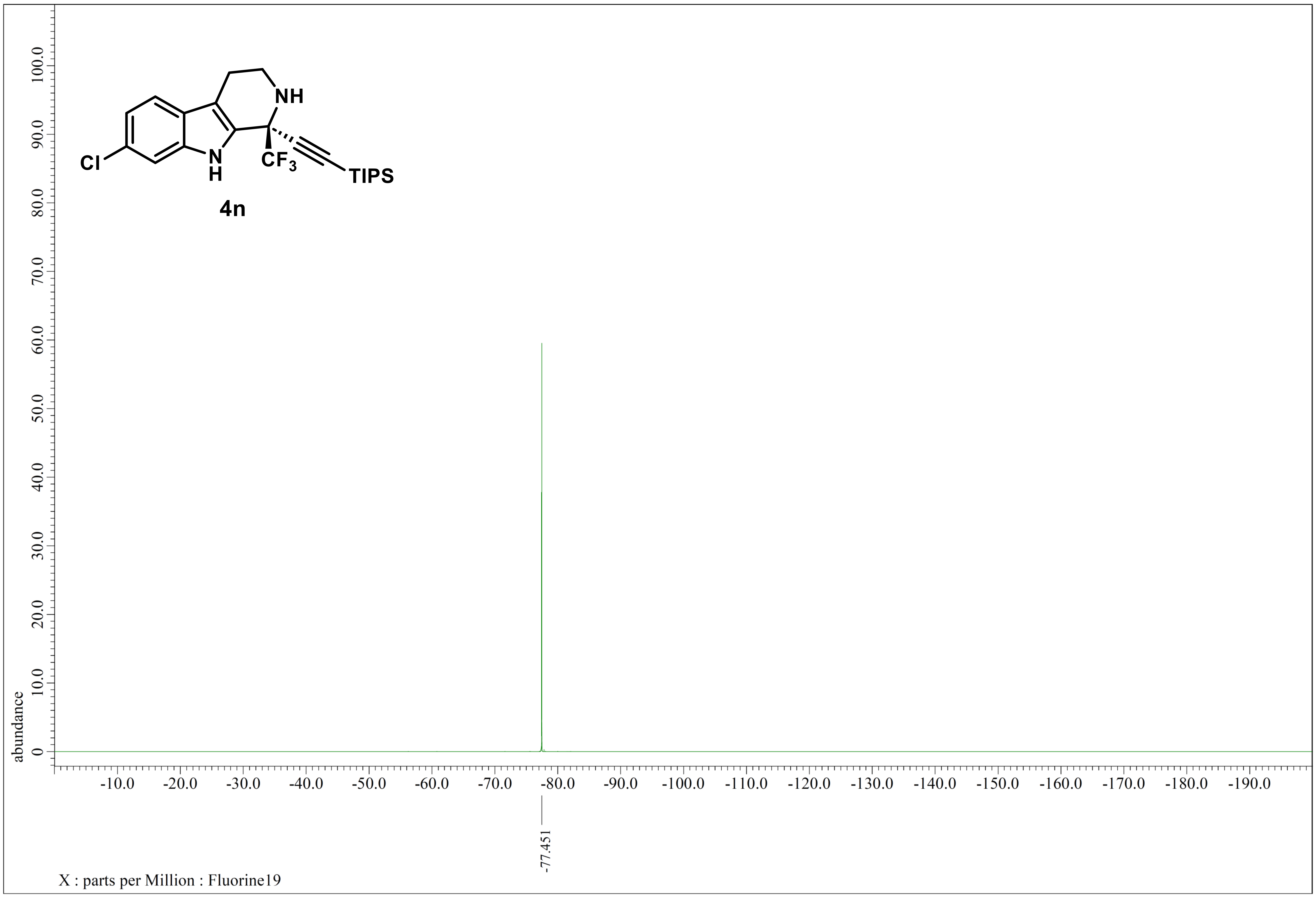
NH
Cl
N
H
CF3
TIPS
4n
abundance
X : parts per Million : Fluorine19
-77.451

^{1}H NMR (600 MHz, $CDCl_3$), ^{13}C NMR (151 MHz $CDCl_3$) and ^{19}F NMR (565 MHz $CDCl_3$) spectra of **4o**

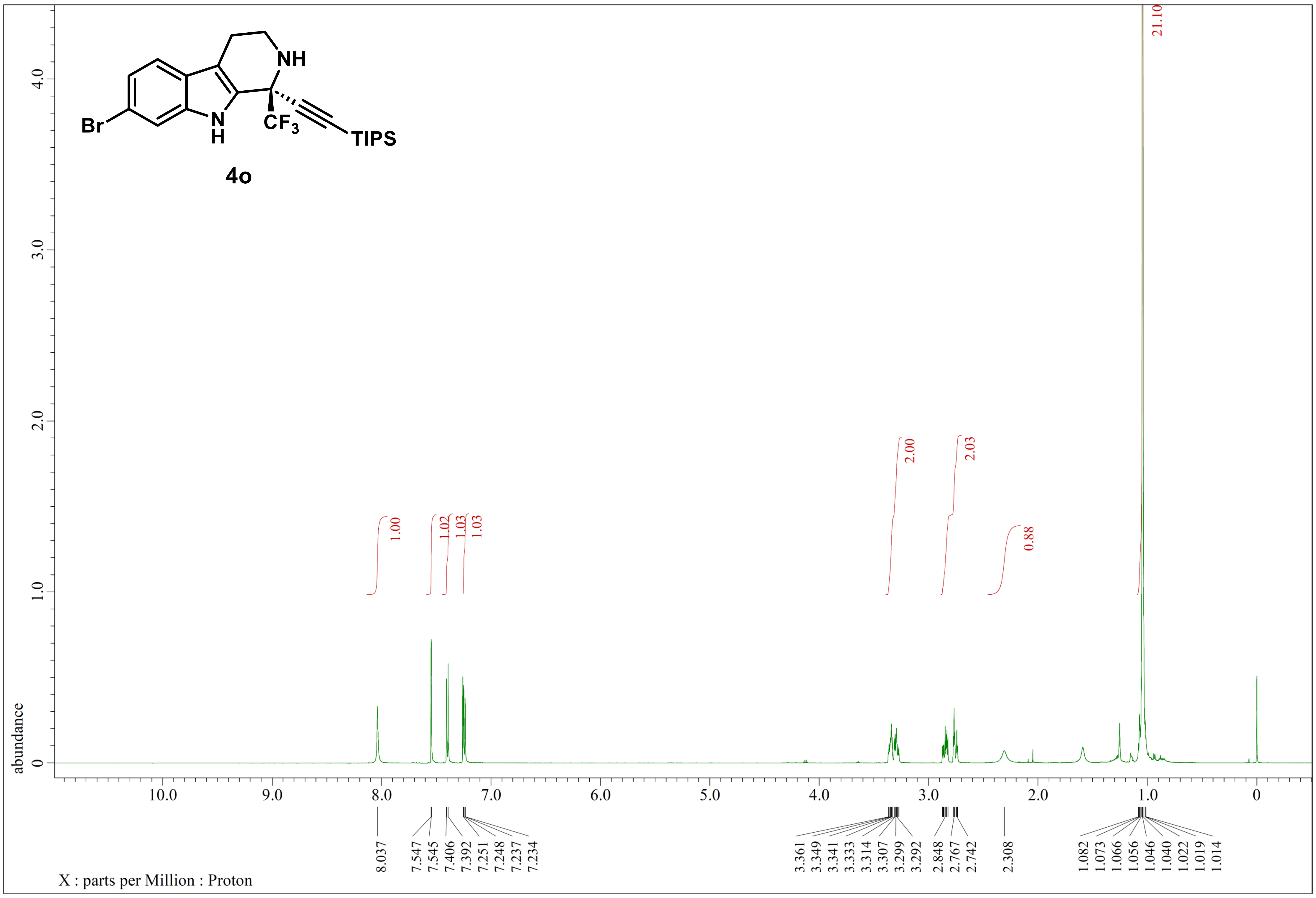

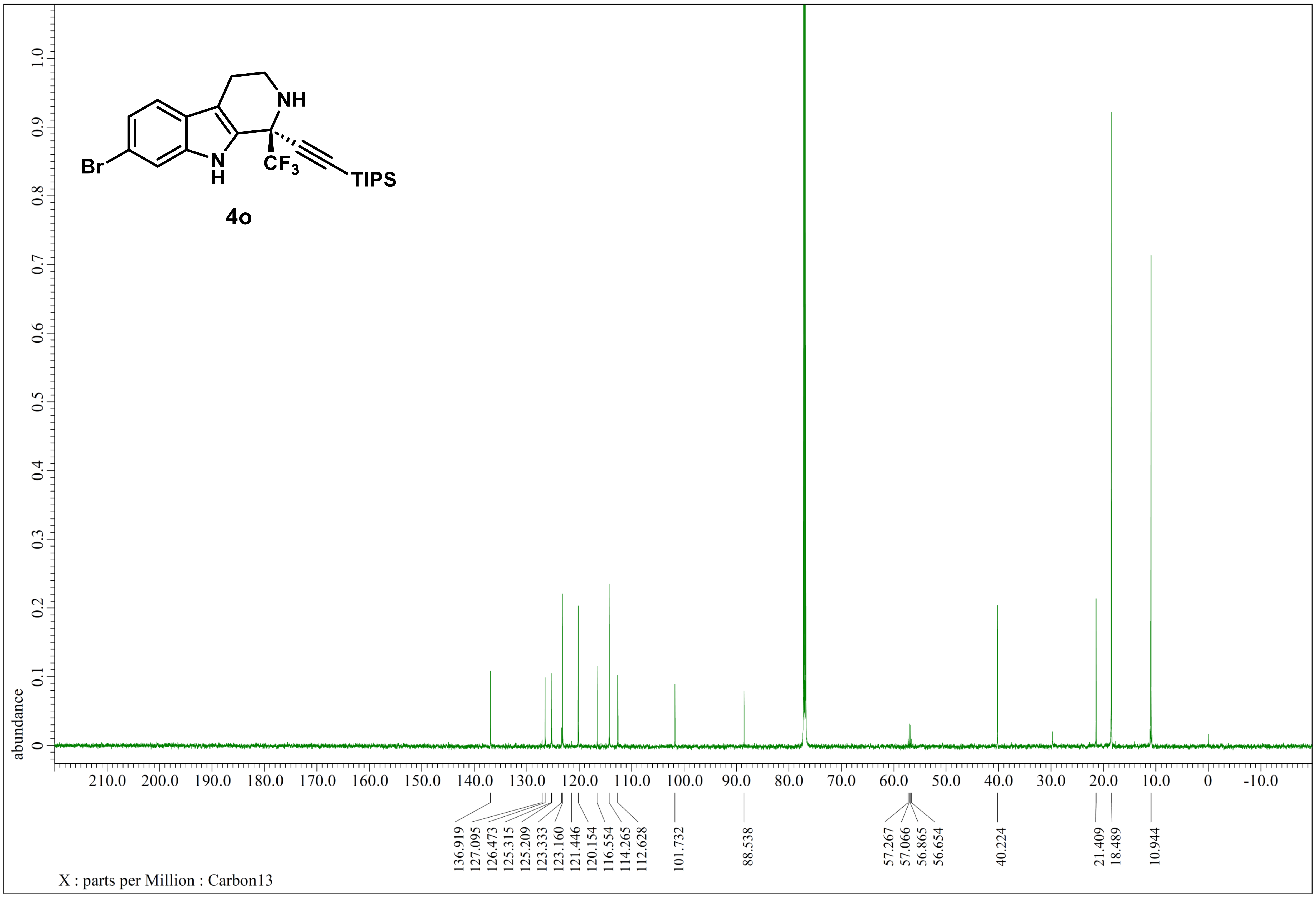

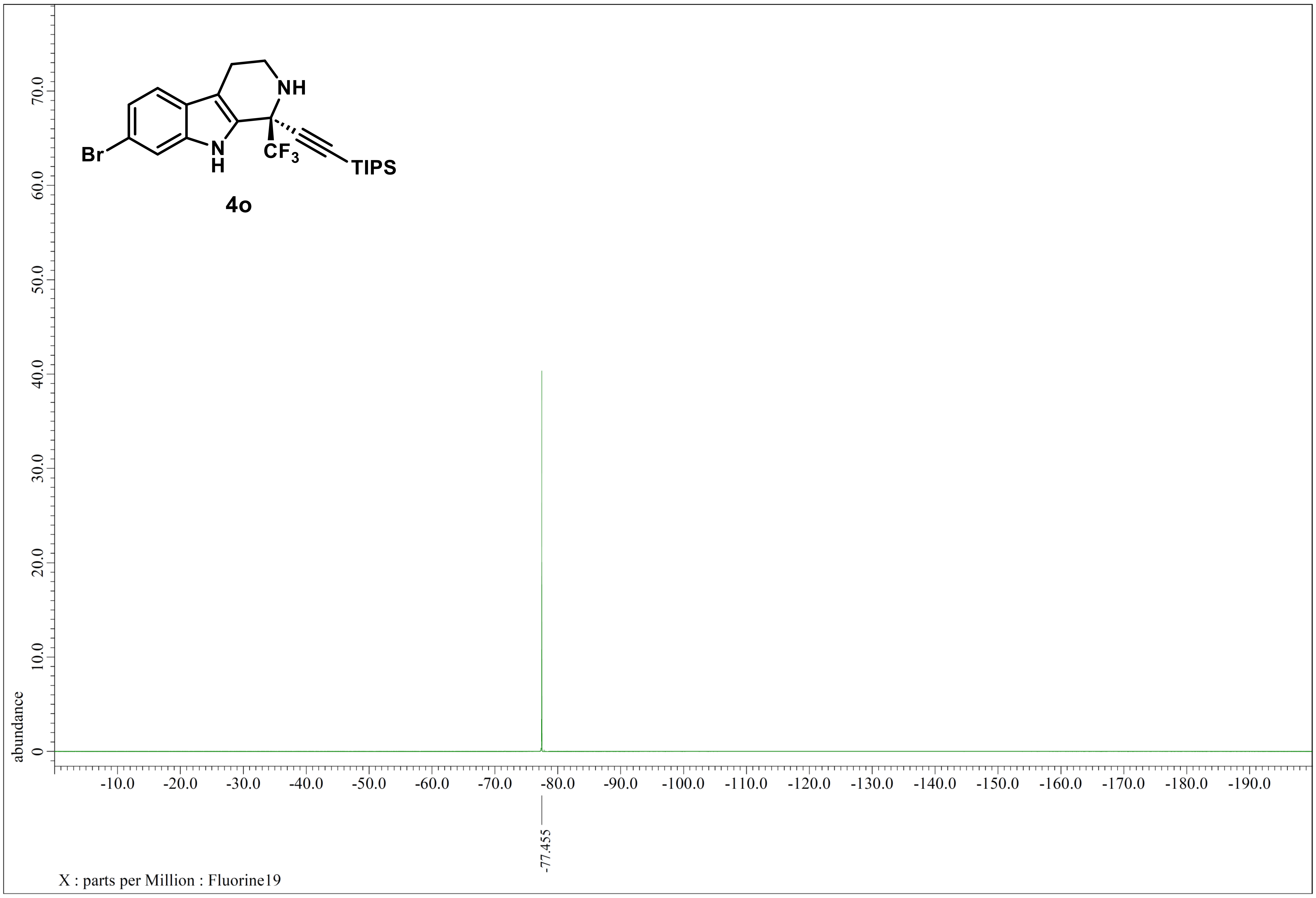

^{1}H NMR (600 MHz, $CDCl_3$), ^{13}C NMR (151 MHz $CDCl_3$) and ^{19}F NMR (565 MHz $CDCl_3$) spectra of

^{1}H NMR (600 MHz, $CDCl_3$), ^{13}C NMR (151 MHz $CDCl_3$) and ^{19}F NMR (565 MHz $CDCl_3$) spectra of **4ab**

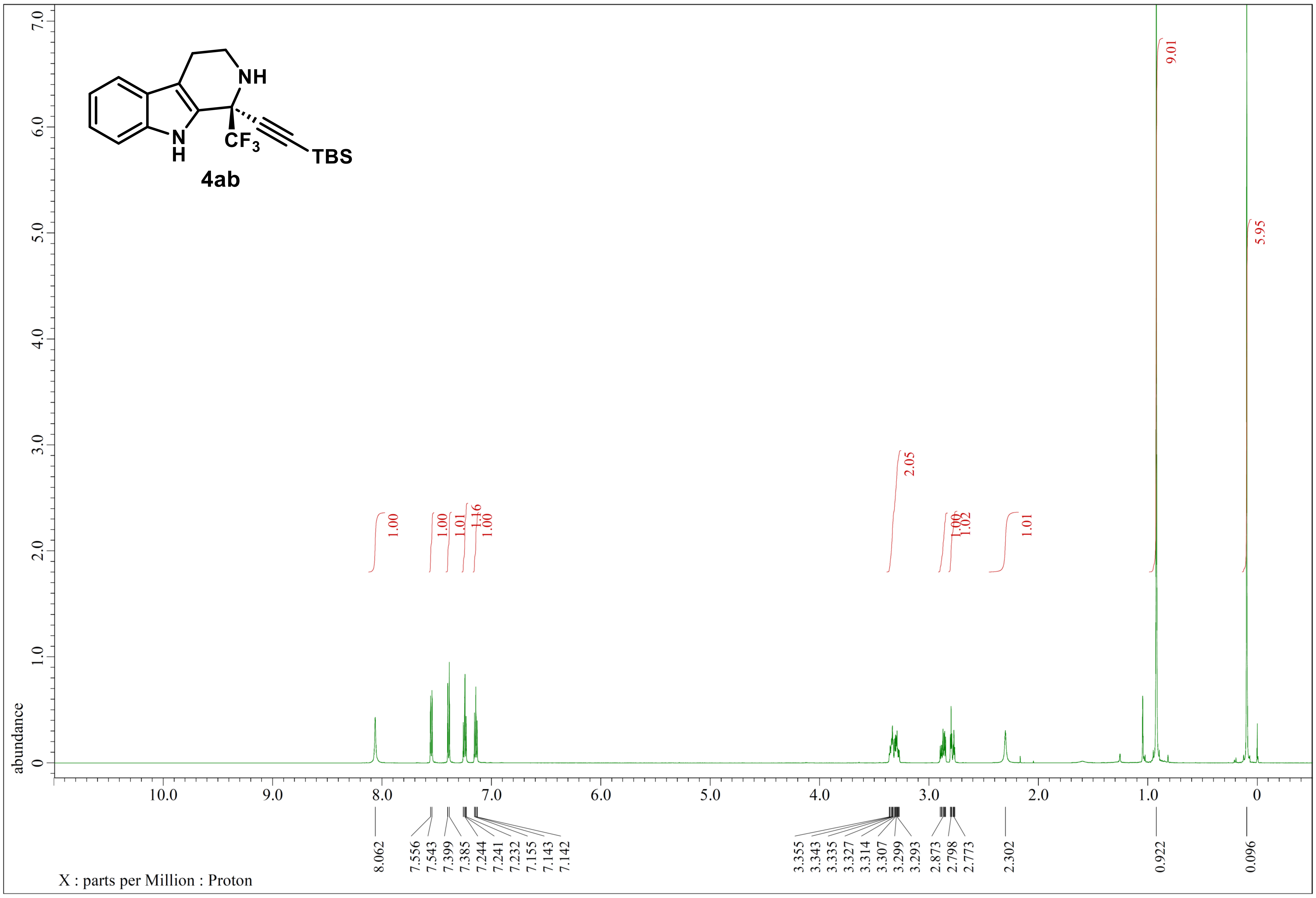

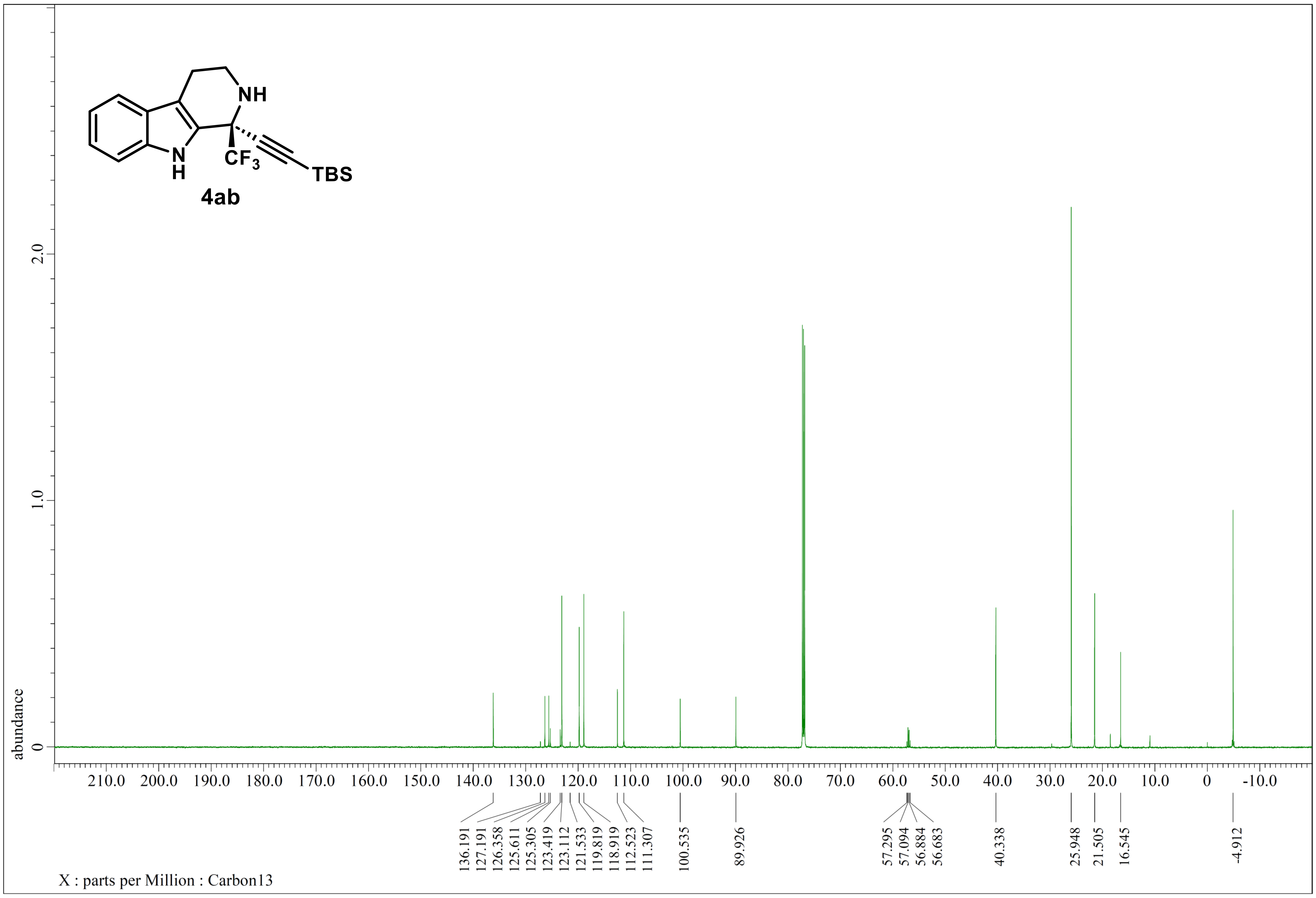

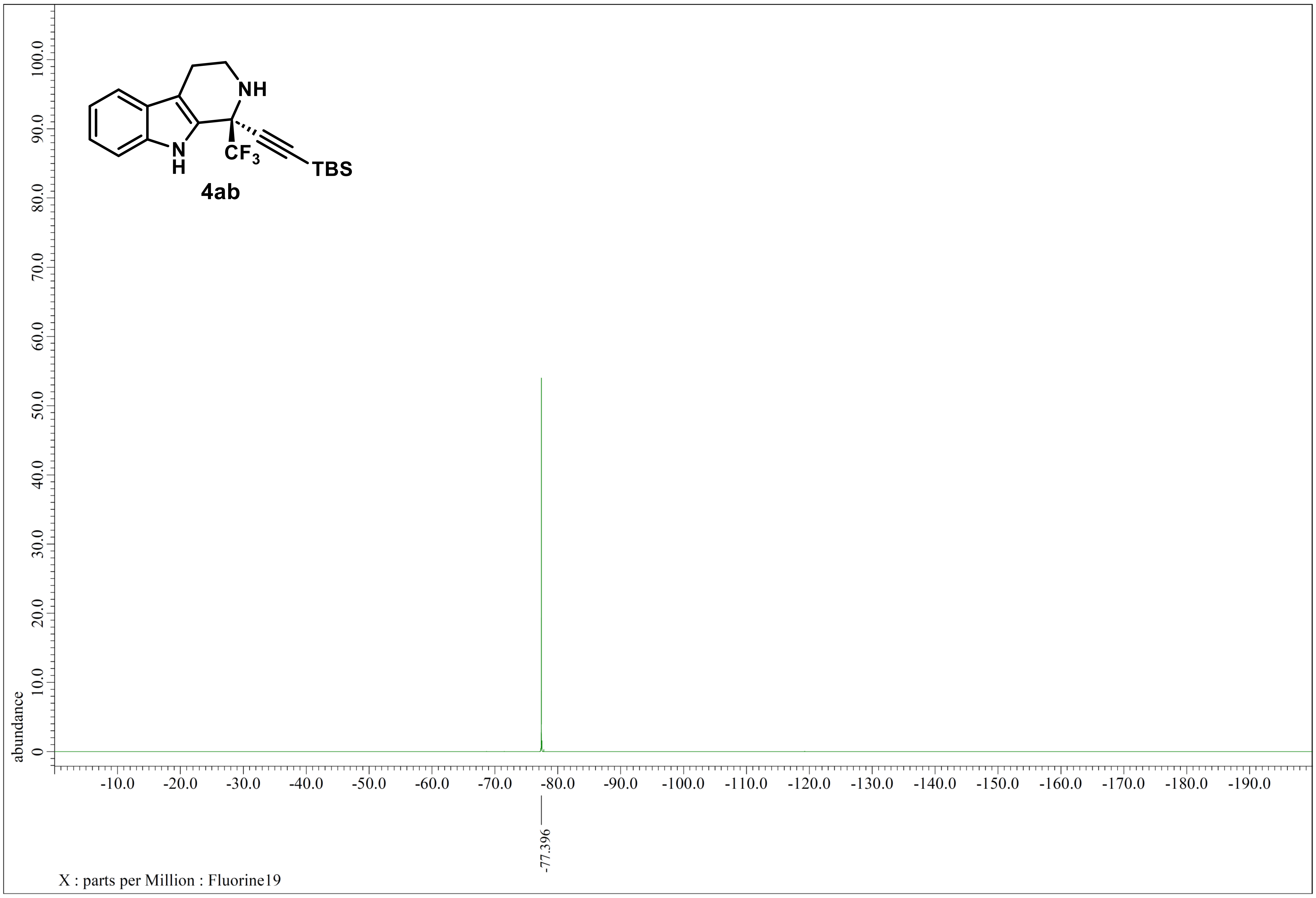
NH
N
H
CF3
TBS
4ab
abundance
-77.396
X : parts per Million : Fluorine19

^{1}H NMR (600 MHz, $CDCl_3$), ^{13}C NMR (151 MHz $CDCl_3$) and ^{19}F NMR (565 MHz $CDCl_3$) spectra of **5i**

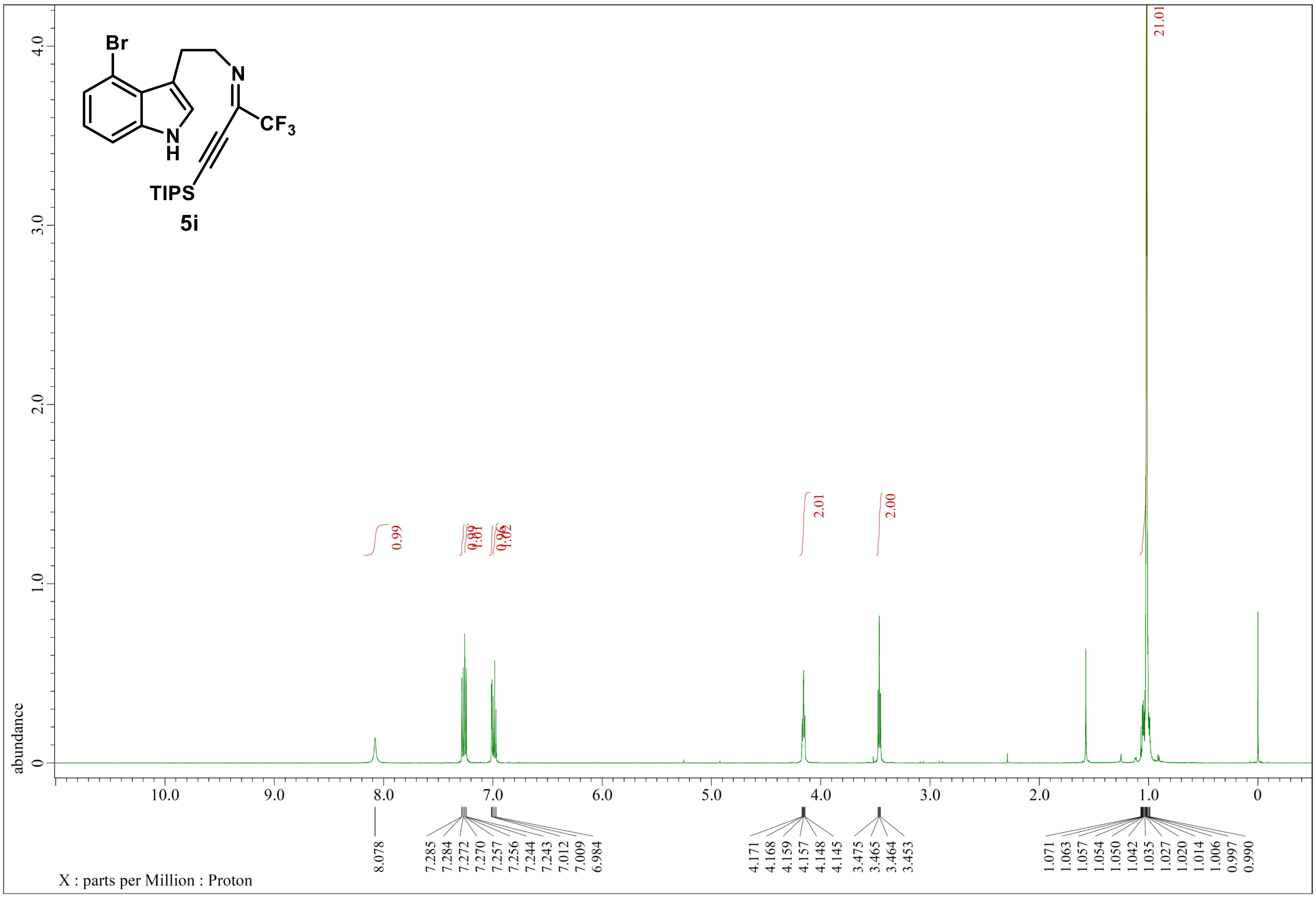

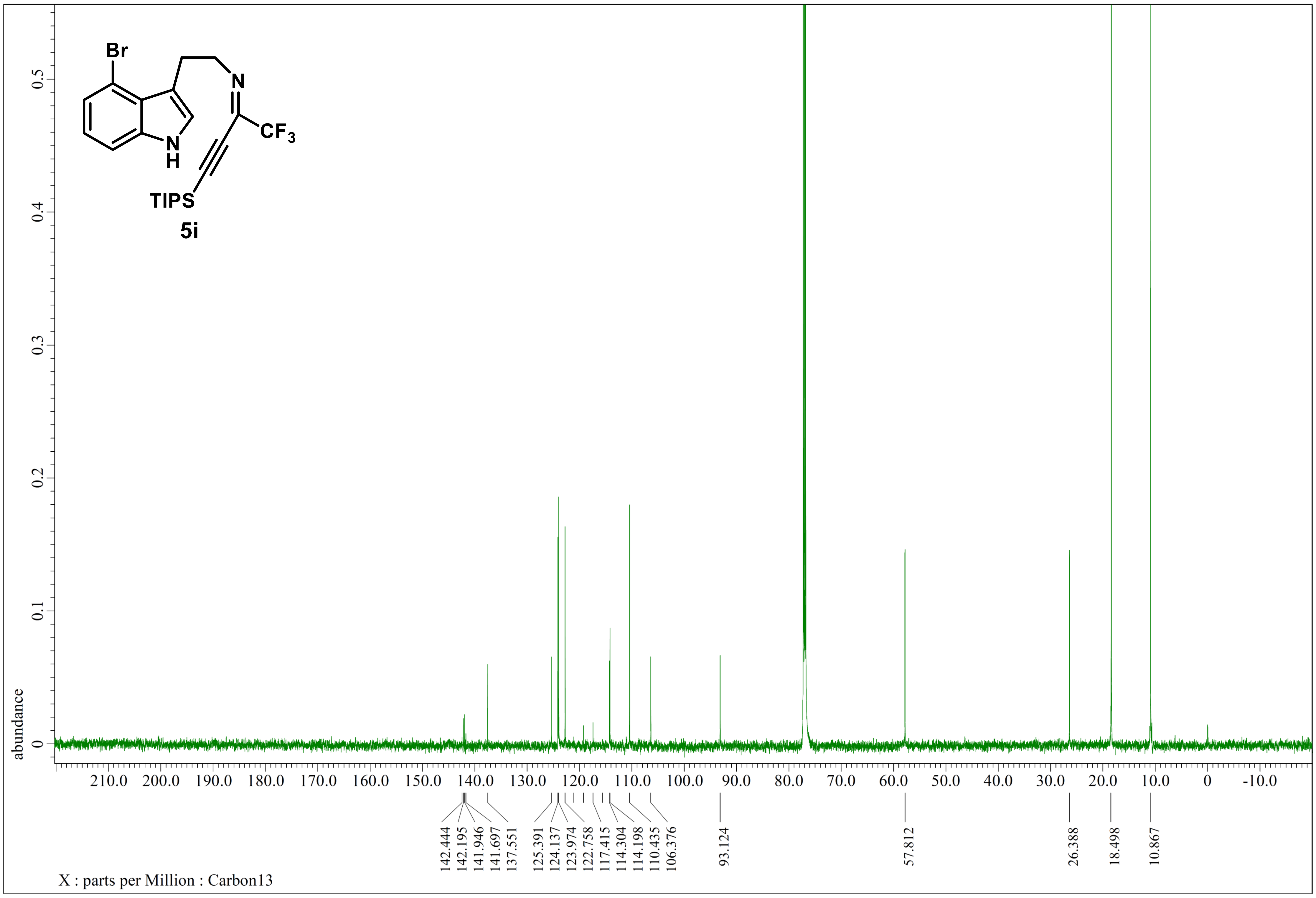

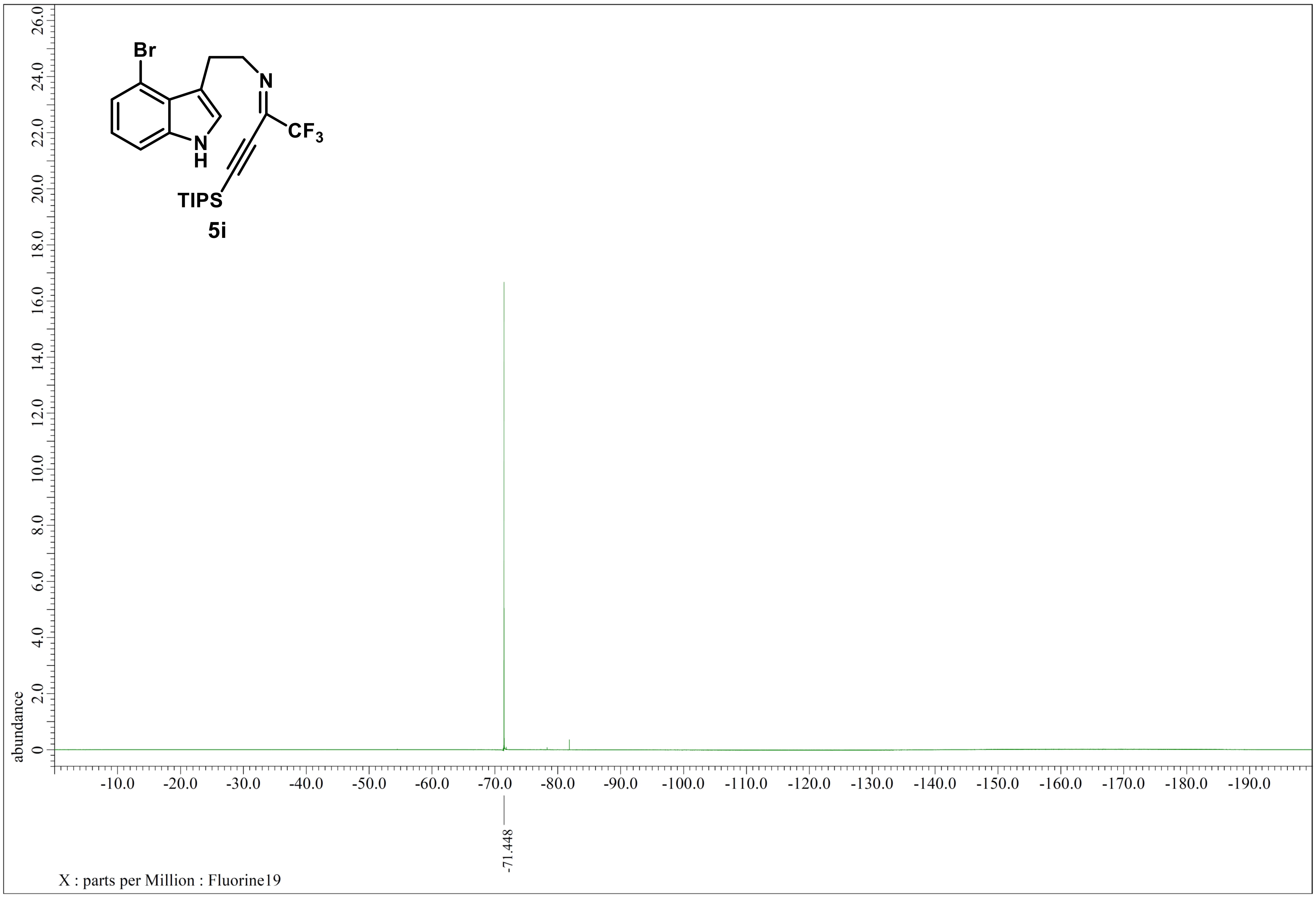
Br
N
CF3
N
H
TIPS
5i
abundance
26.0
24.0
22.0
20.0
18.0
16.0
14.0
12.0
10.0
8.0
6.0
4.0
2.0
0
-10.0
-20.0
-30.0
-40.0
-50.0
-60.0
-70.0
-80.0
-90.0
-100.0
-110.0
-120.0
-130.0
-140.0
-150.0
-160.0
-170.0
-180.0
-190.0
-71.448
X : parts per Million : Fluorine19

^{1}H NMR (600 MHz, $CDCl_3$), ^{13}C NMR (151 MHz $CDCl_3$) and ^{19}F NMR (565 MHz $CDCl_3$) spectra of **S5**

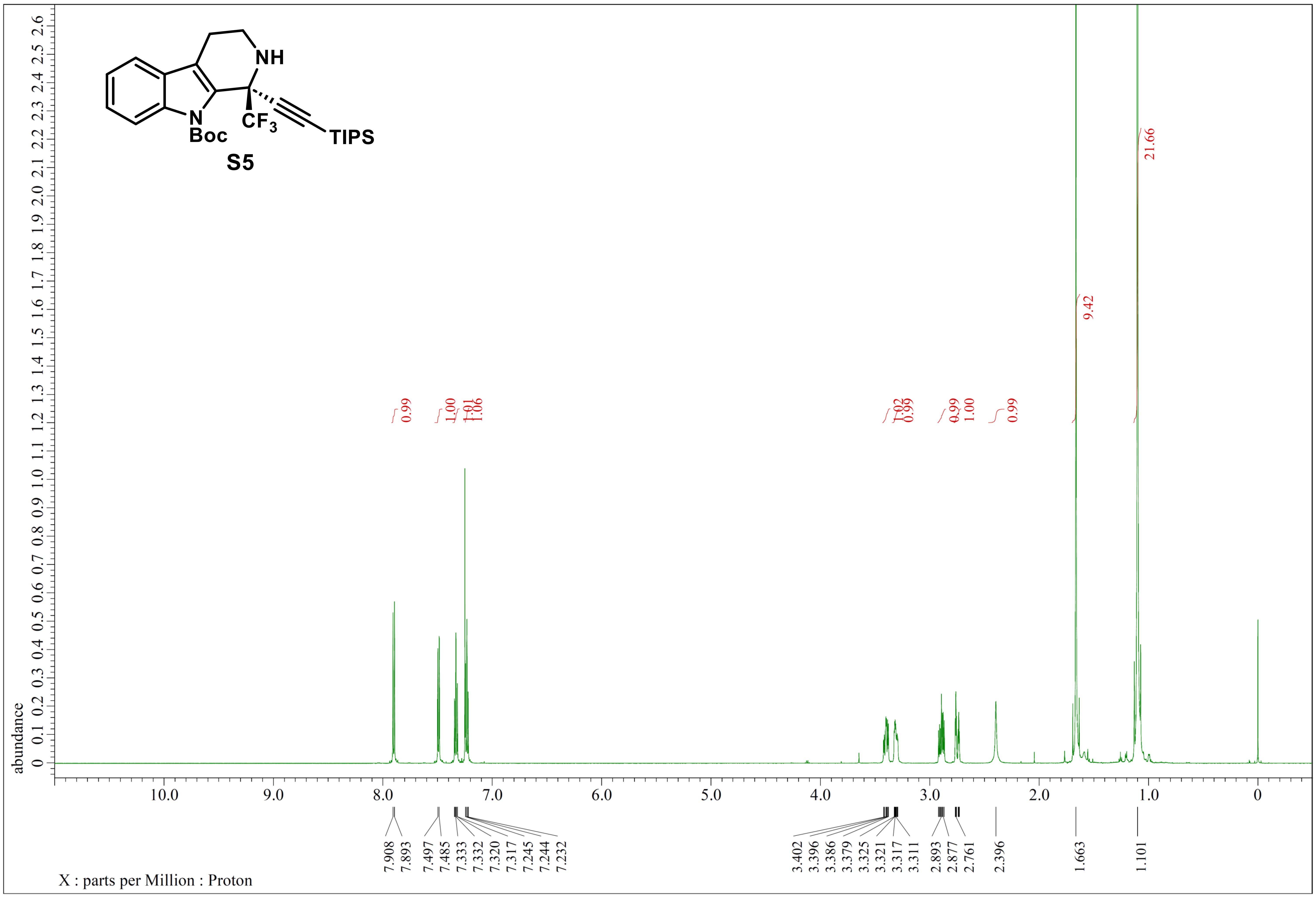

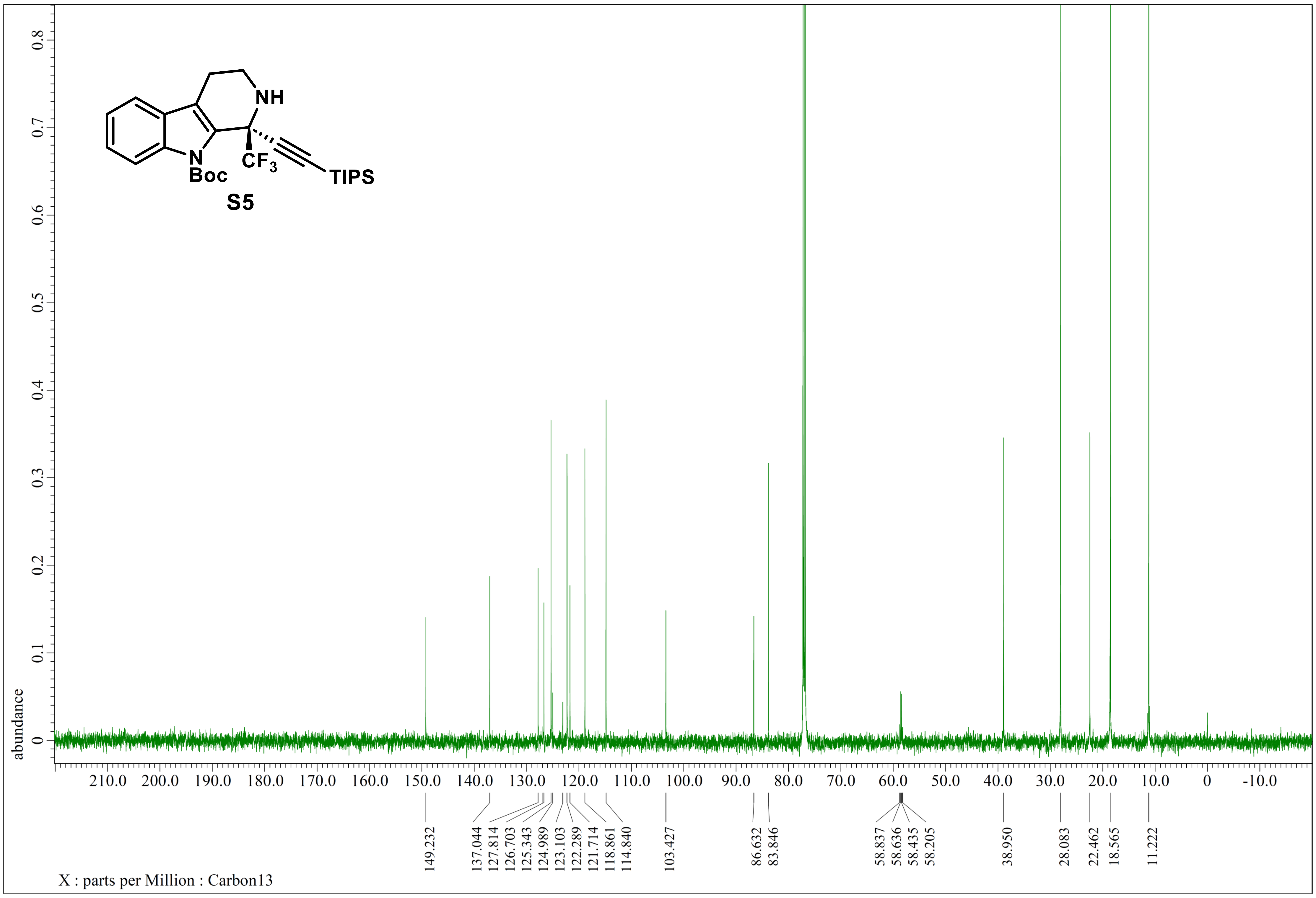

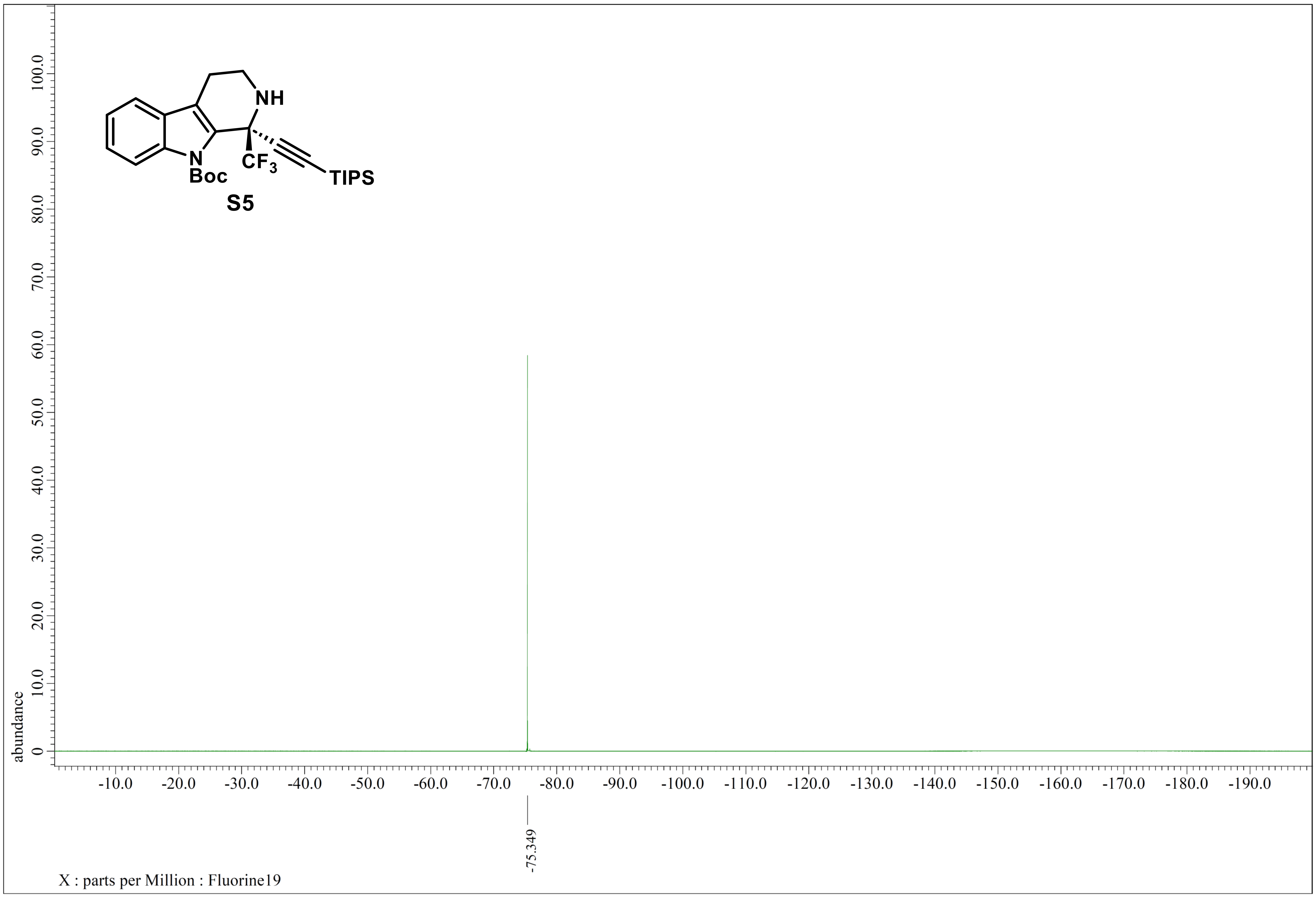

^{1}H NMR (600 MHz, $CDCl_3$), ^{13}C NMR (151 MHz $CDCl_3$) and ^{19}F NMR (565 MHz $CDCl_3$) spectra of **6**

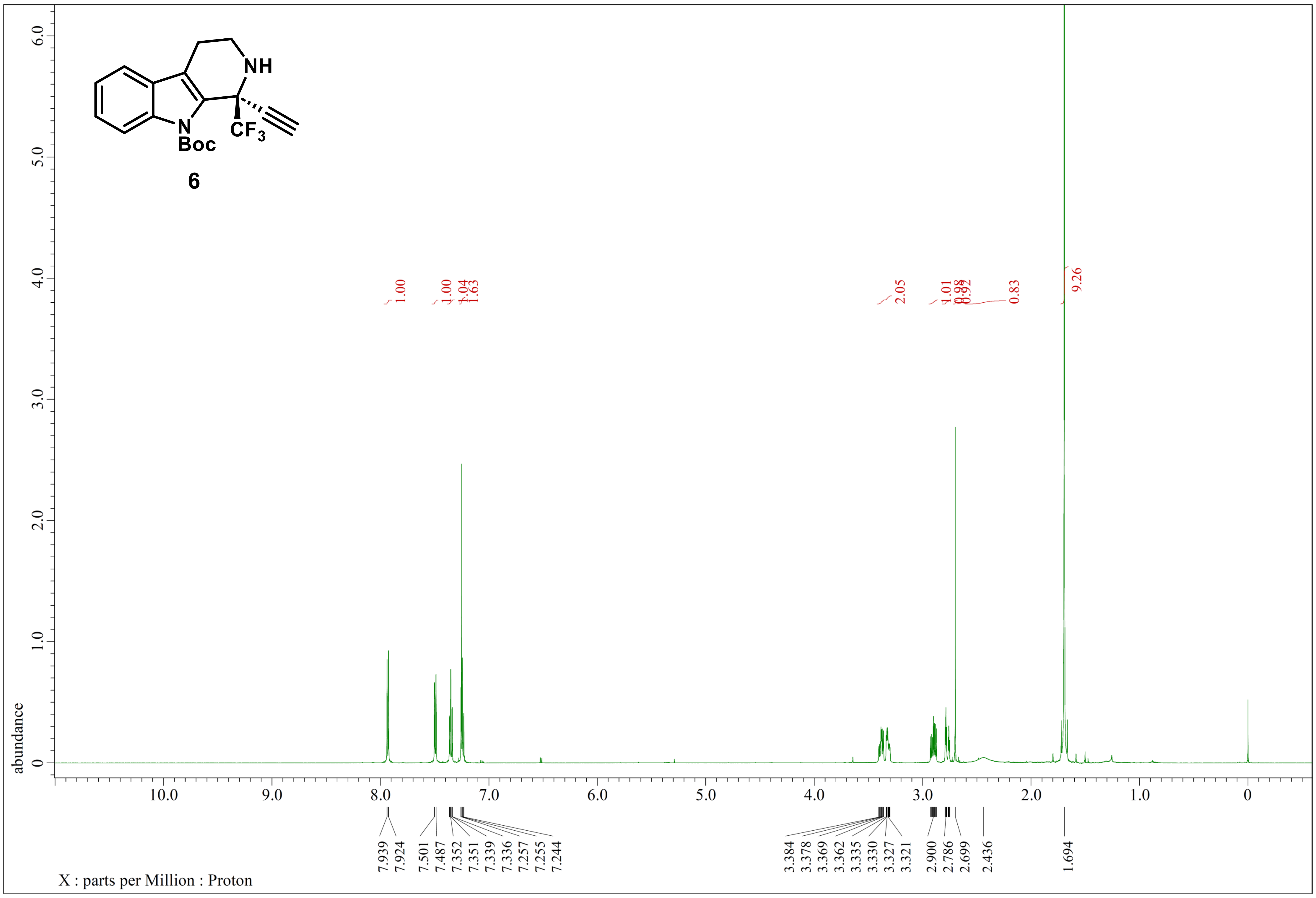

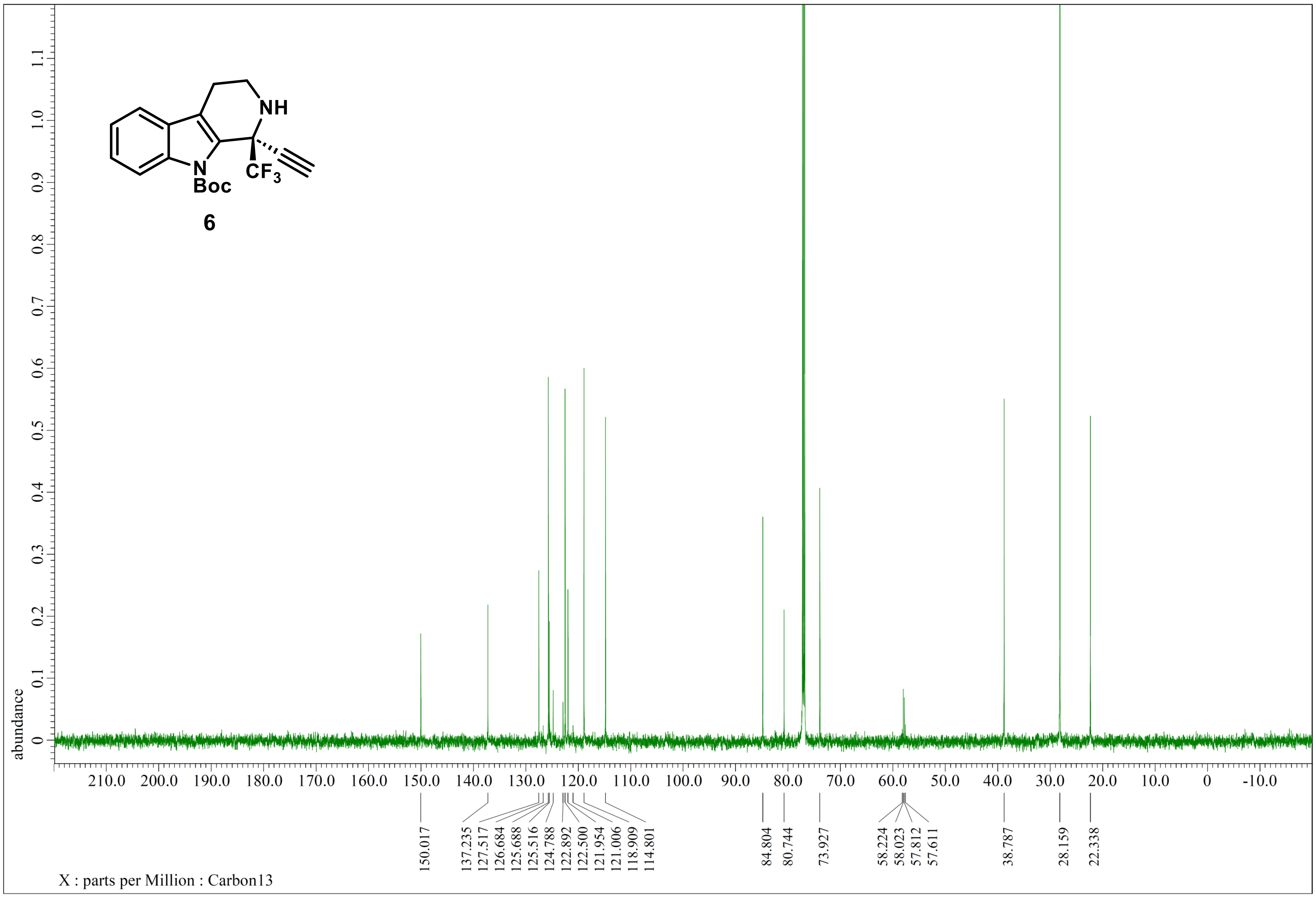
6
abundance
150.017
137.235
127.517
126.684
125.688
125.516
124.788
122.892
122.500
121.954
121.006
118.909
114.801
84.804
80.744
73.927
58.224
58.023
57.812
57.611
38.787
28.159
22.338
X : parts per Million : Carbon13

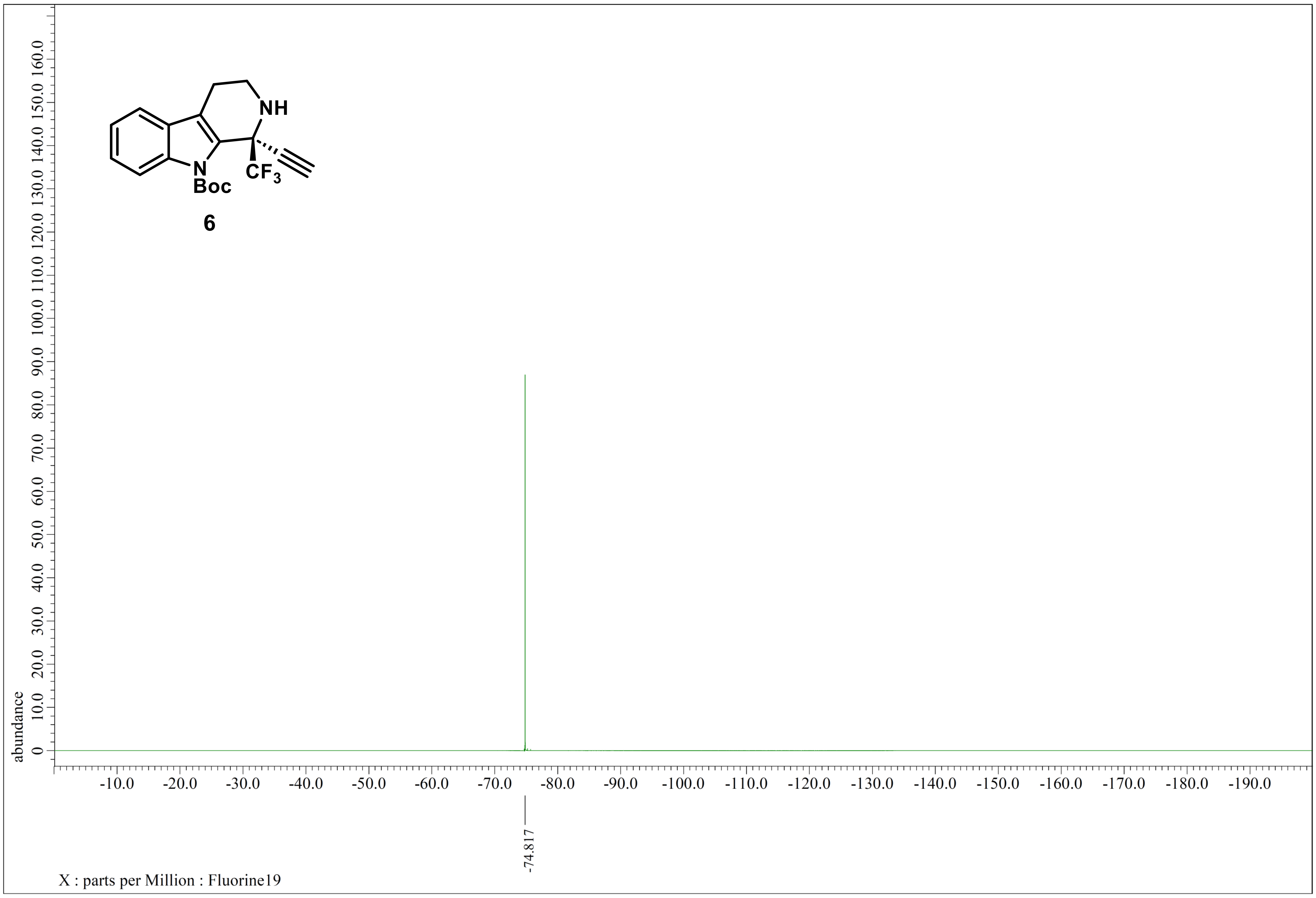
6
abundance
-74.817
X : parts per Million : Fluorine19

^{1}H NMR (600 MHz, $CDCl_3$), ^{13}C NMR (151 MHz $CDCl_3$) and ^{19}F NMR (565 MHz $CDCl_3$) spectra of **7**

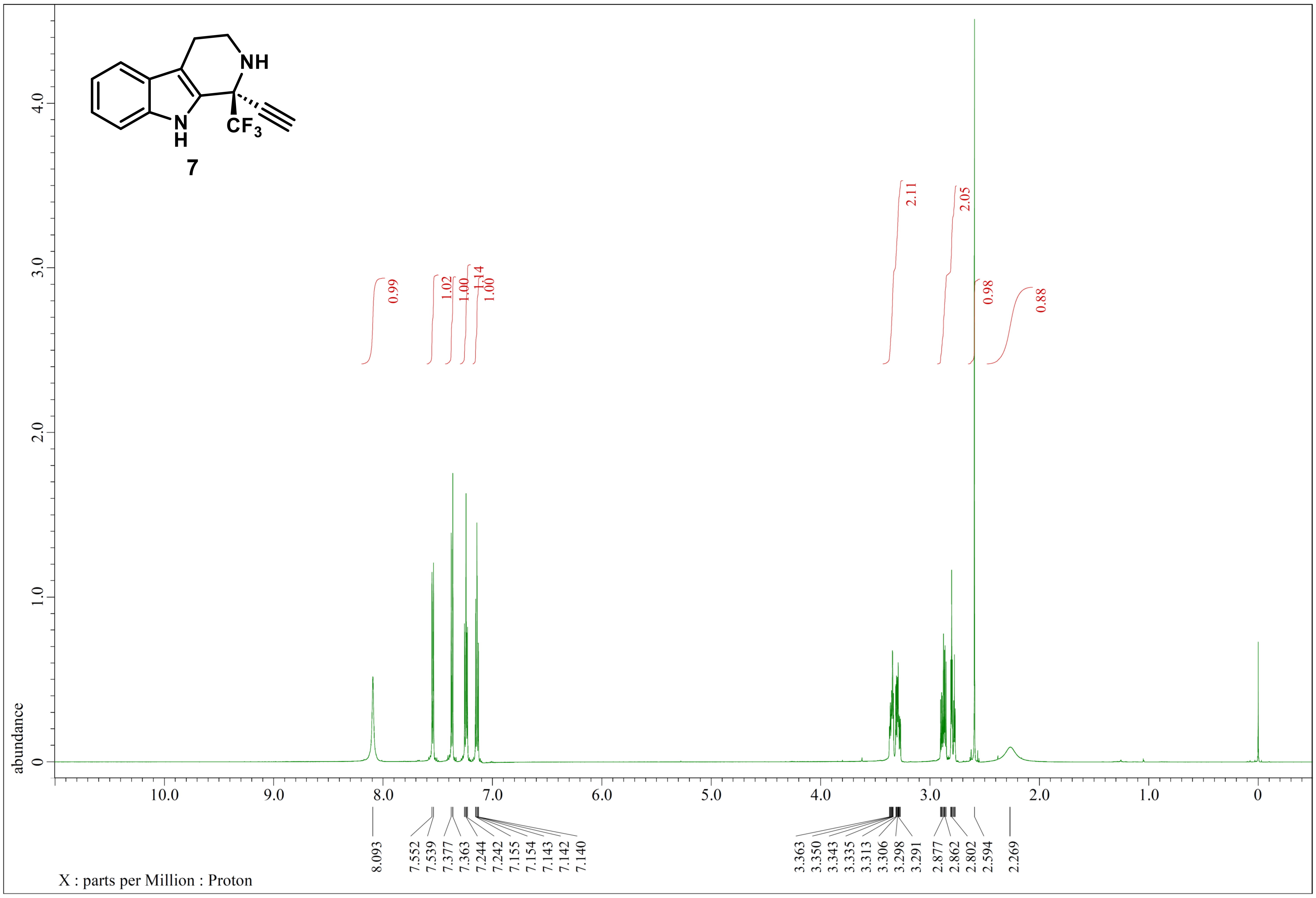

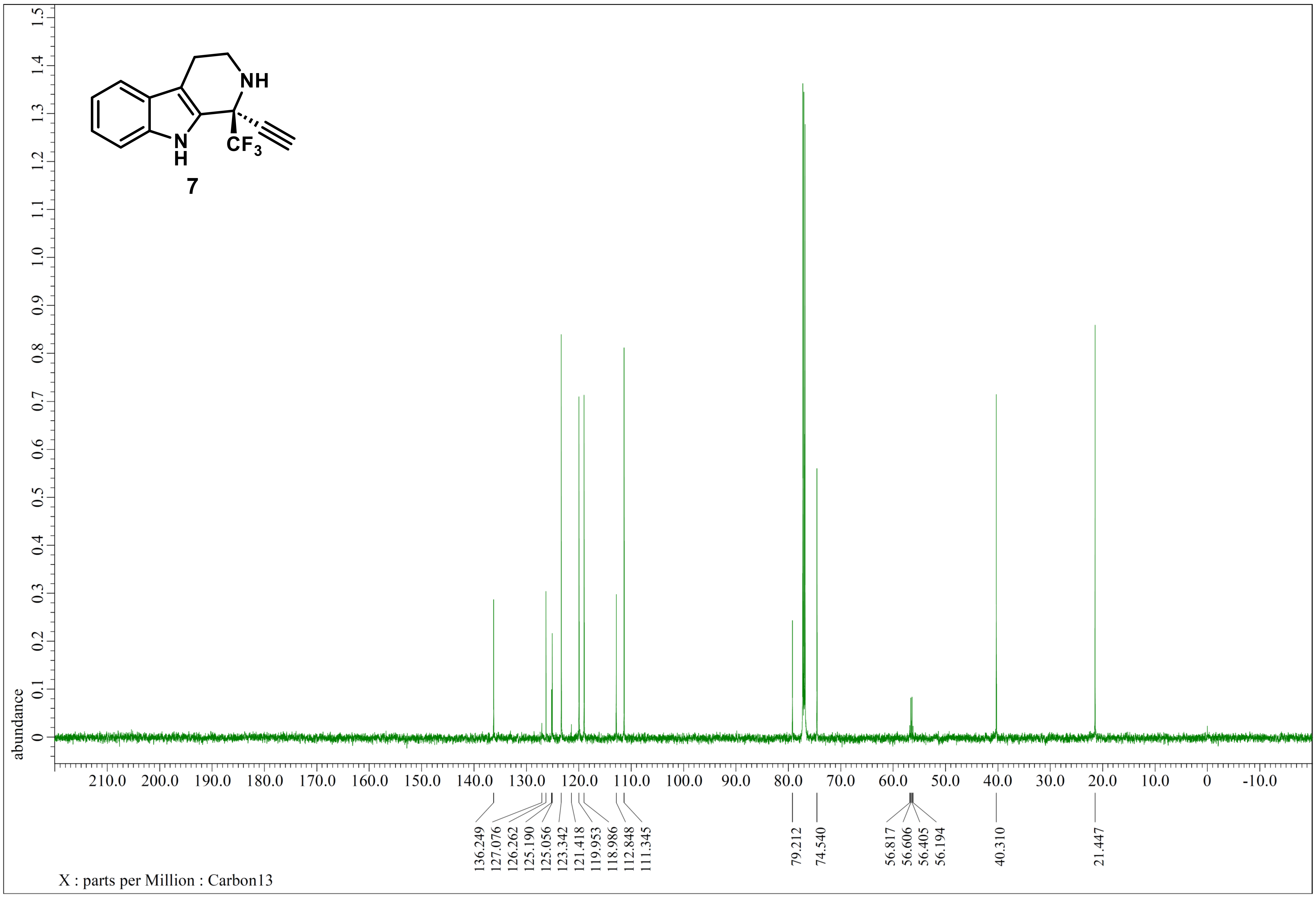

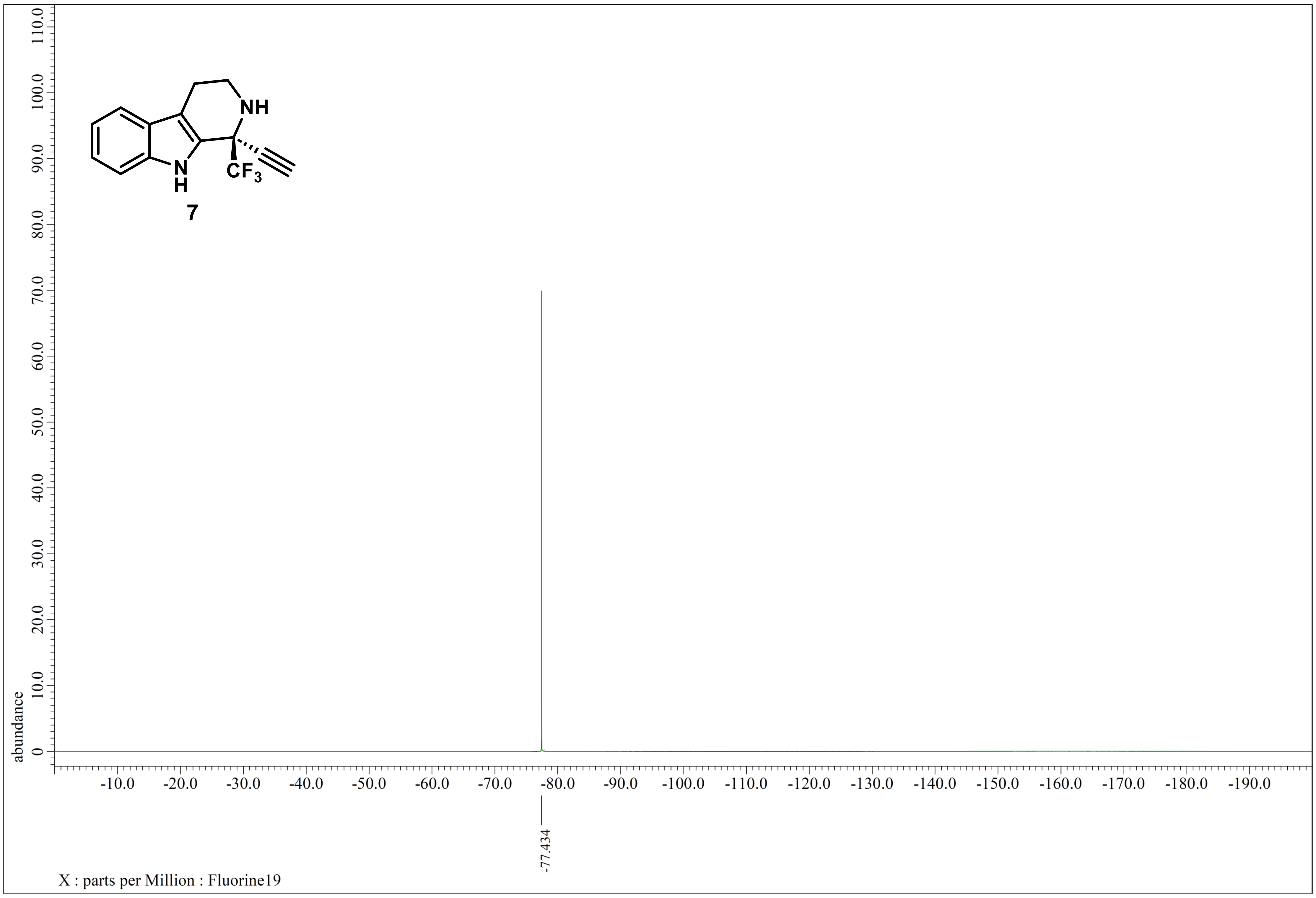

^{1}H NMR (600 MHz, $CDCl_3$), ^{13}C NMR (151 MHz $CDCl_3$) and ^{19}F NMR (565 MHz $CDCl_3$) spectra of **8**

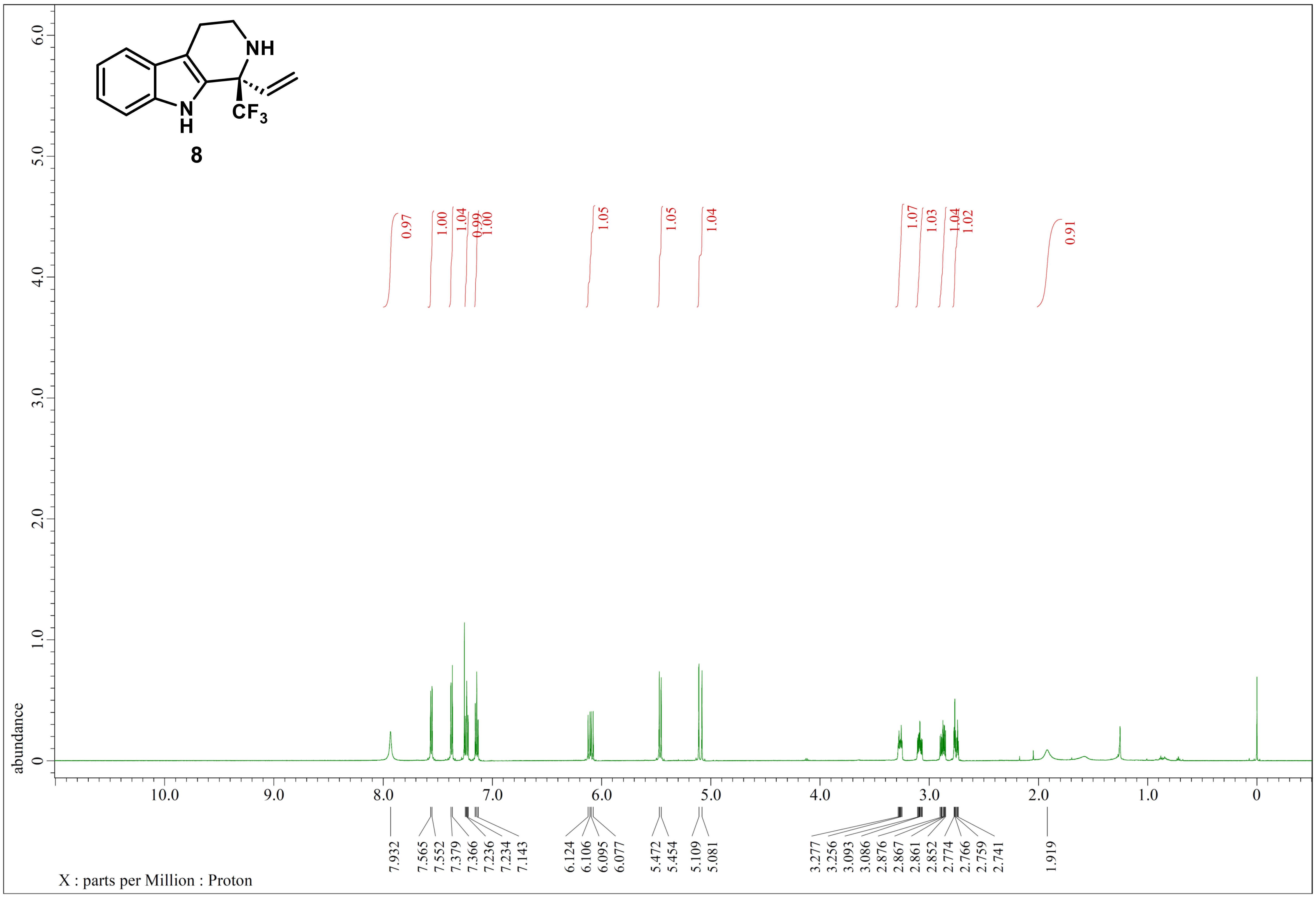

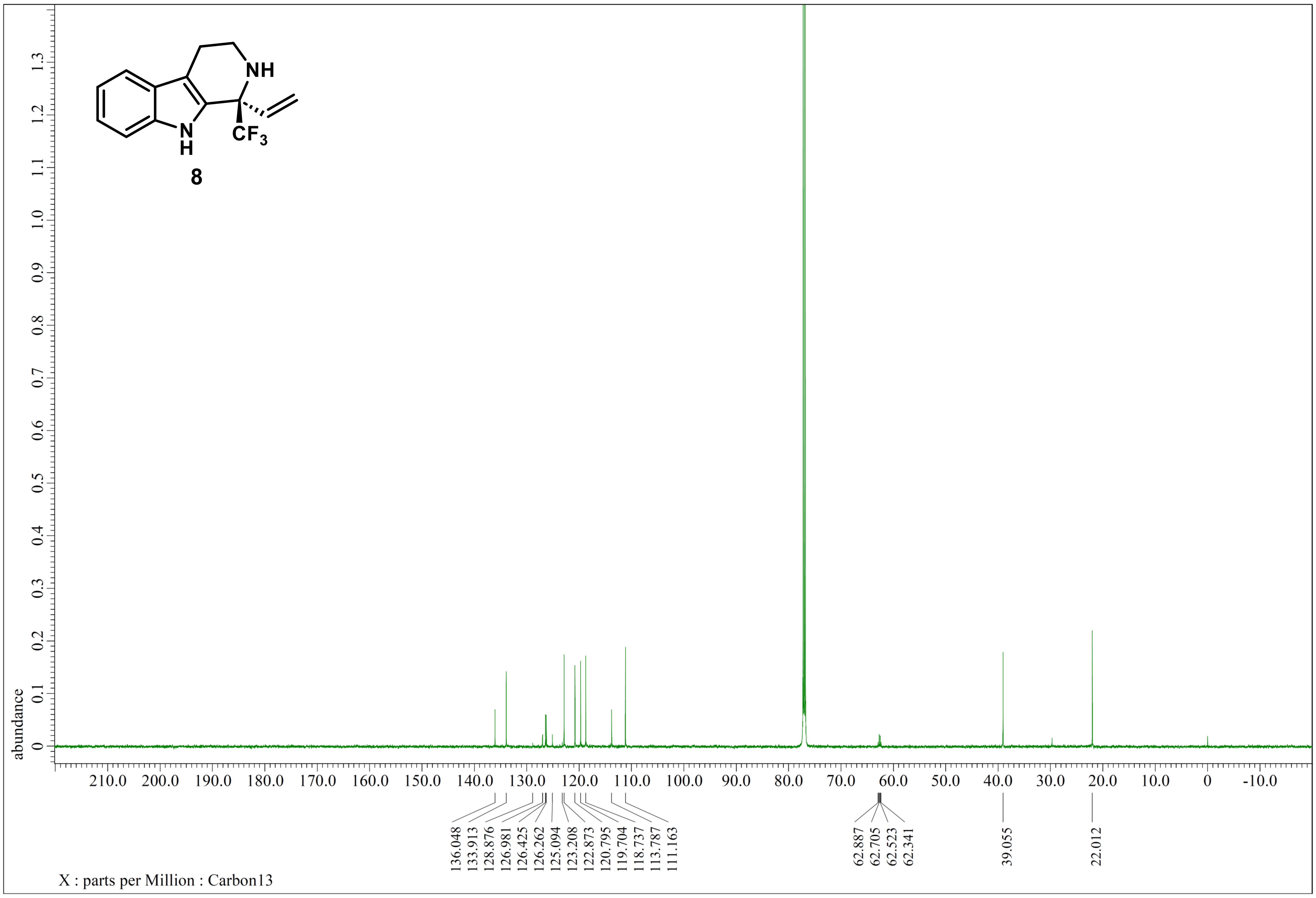
NH
N
H
CF3
8
abundance
X : parts per Million : Carbon13
136.048
133.913
128.876
126.981
126.425
126.262
125.094
123.208
122.873
120.795
119.704
118.737
113.787
111.163
62.887
62.705
62.523
62.341
39.055
22.012

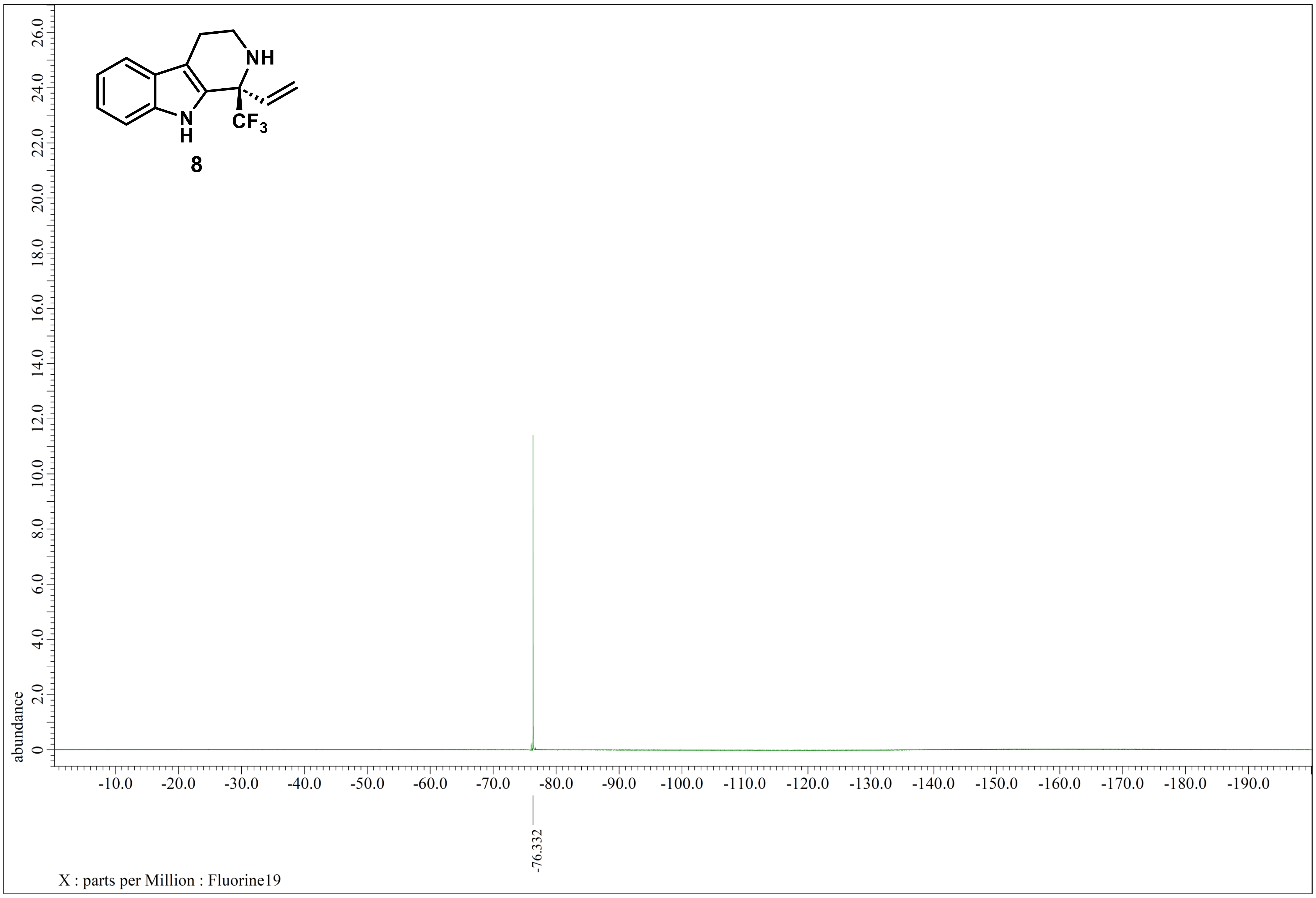
NH
N
H
CF3
8
abundance
X : parts per Million : Fluorine19
-76.332

^{1}H NMR (600 MHz, $CDCl_3$), ^{13}C NMR (151 MHz $CDCl_3$) and ^{19}F NMR (565 MHz $CDCl_3$) spectra of **9**

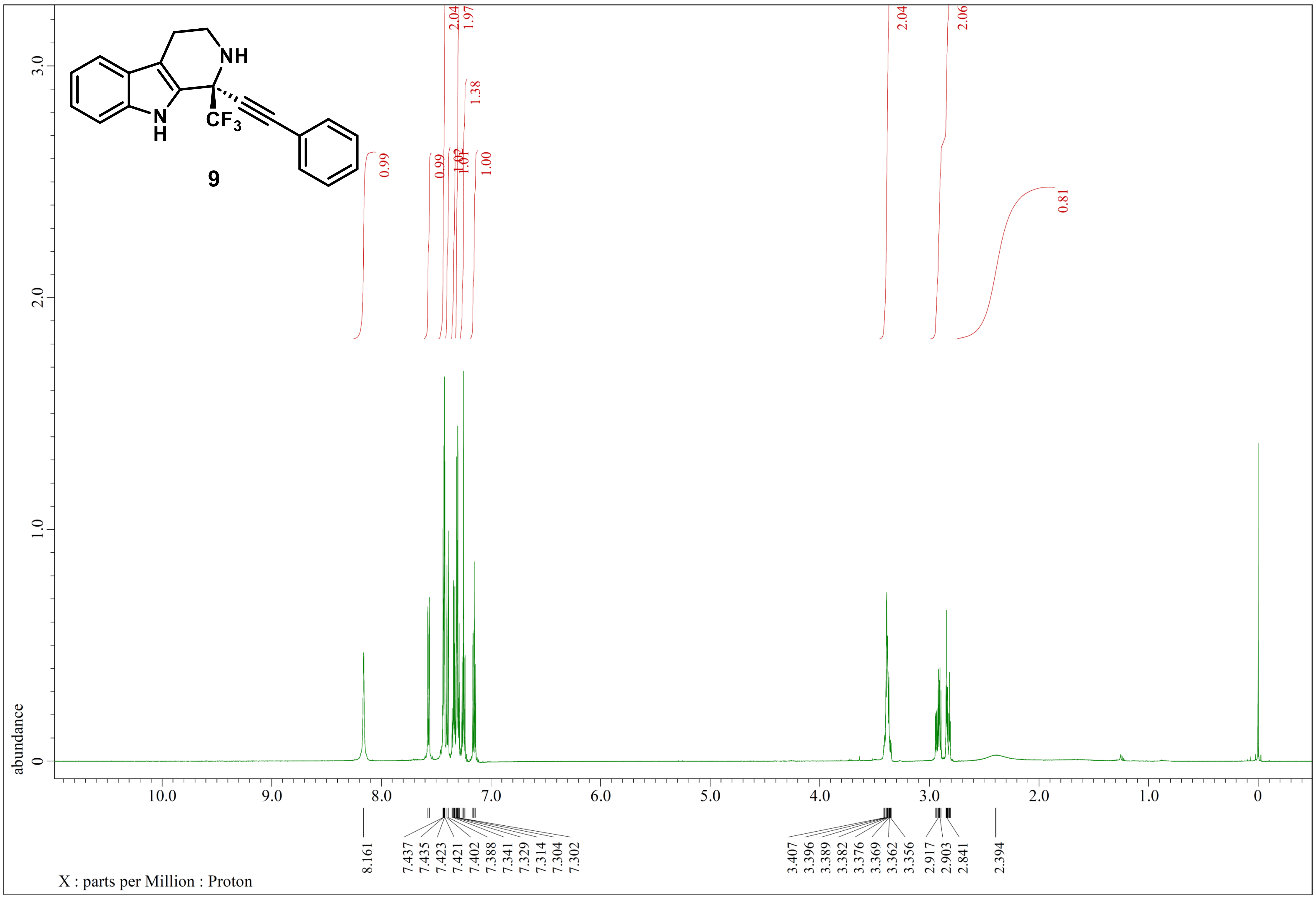

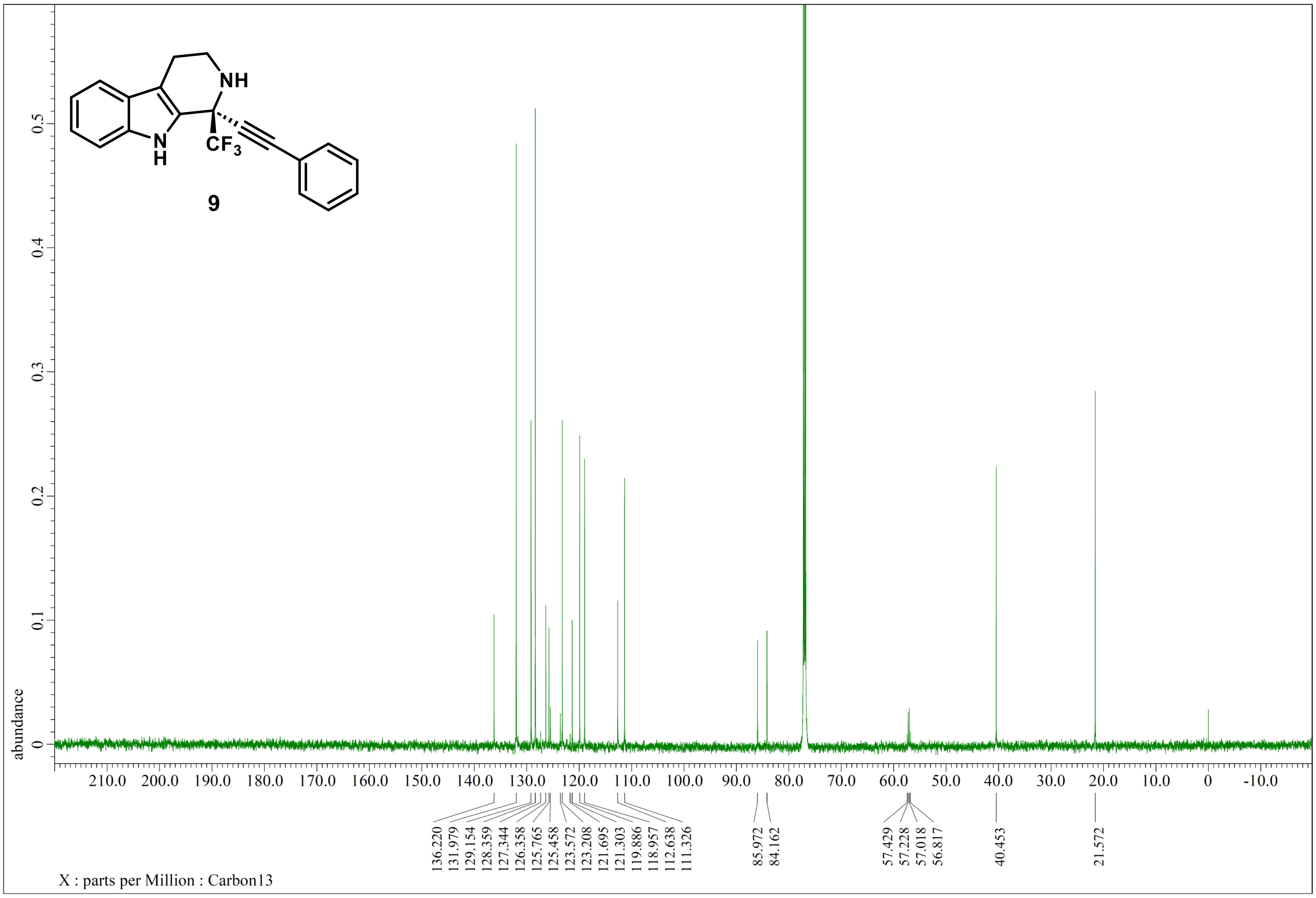

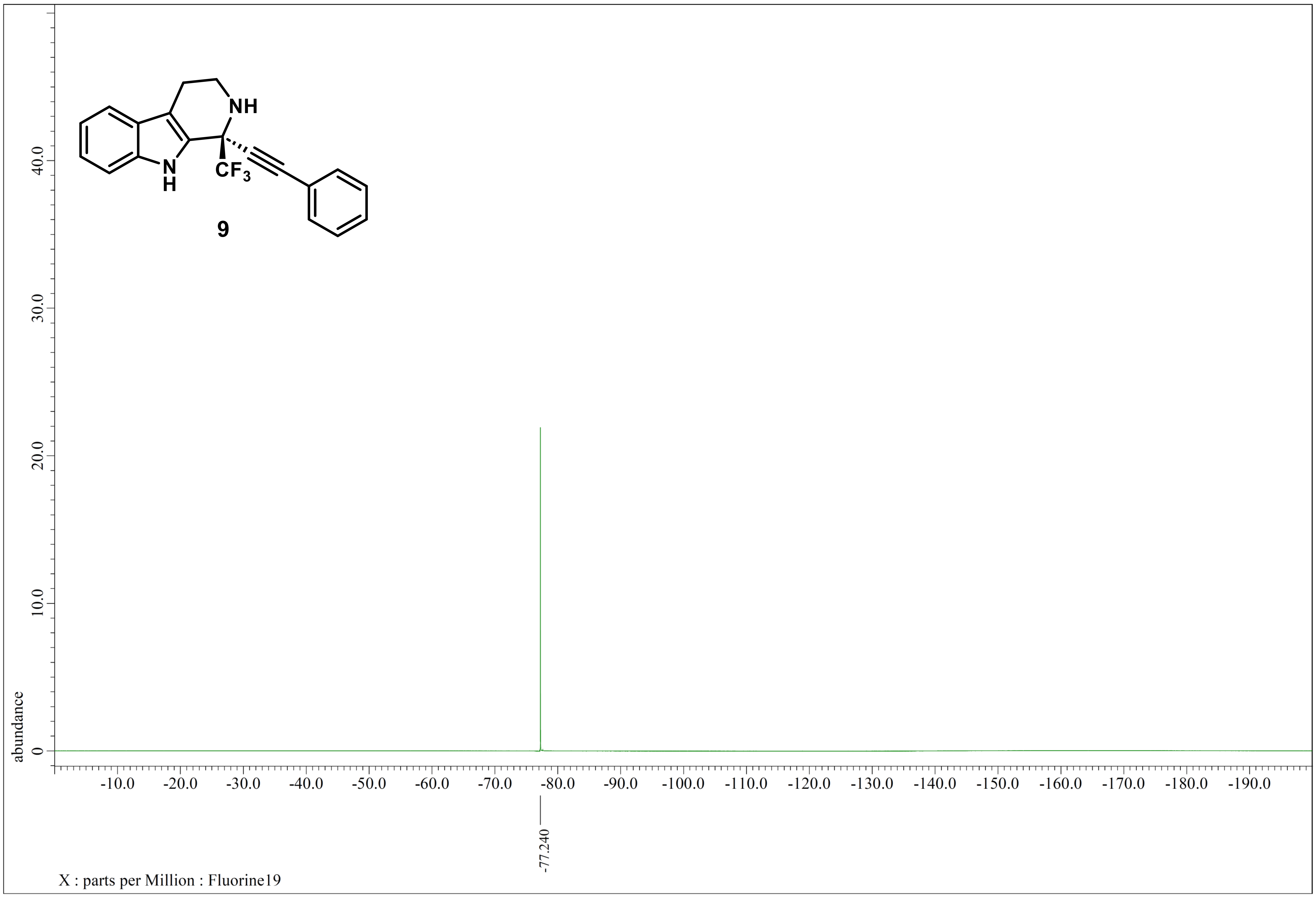

^{1}H NMR (600 MHz, $CDCl_3$), ^{13}C NMR (151 MHz $CDCl_3$) and ^{19}F NMR (565 MHz $CDCl_3$) spectra of **10**

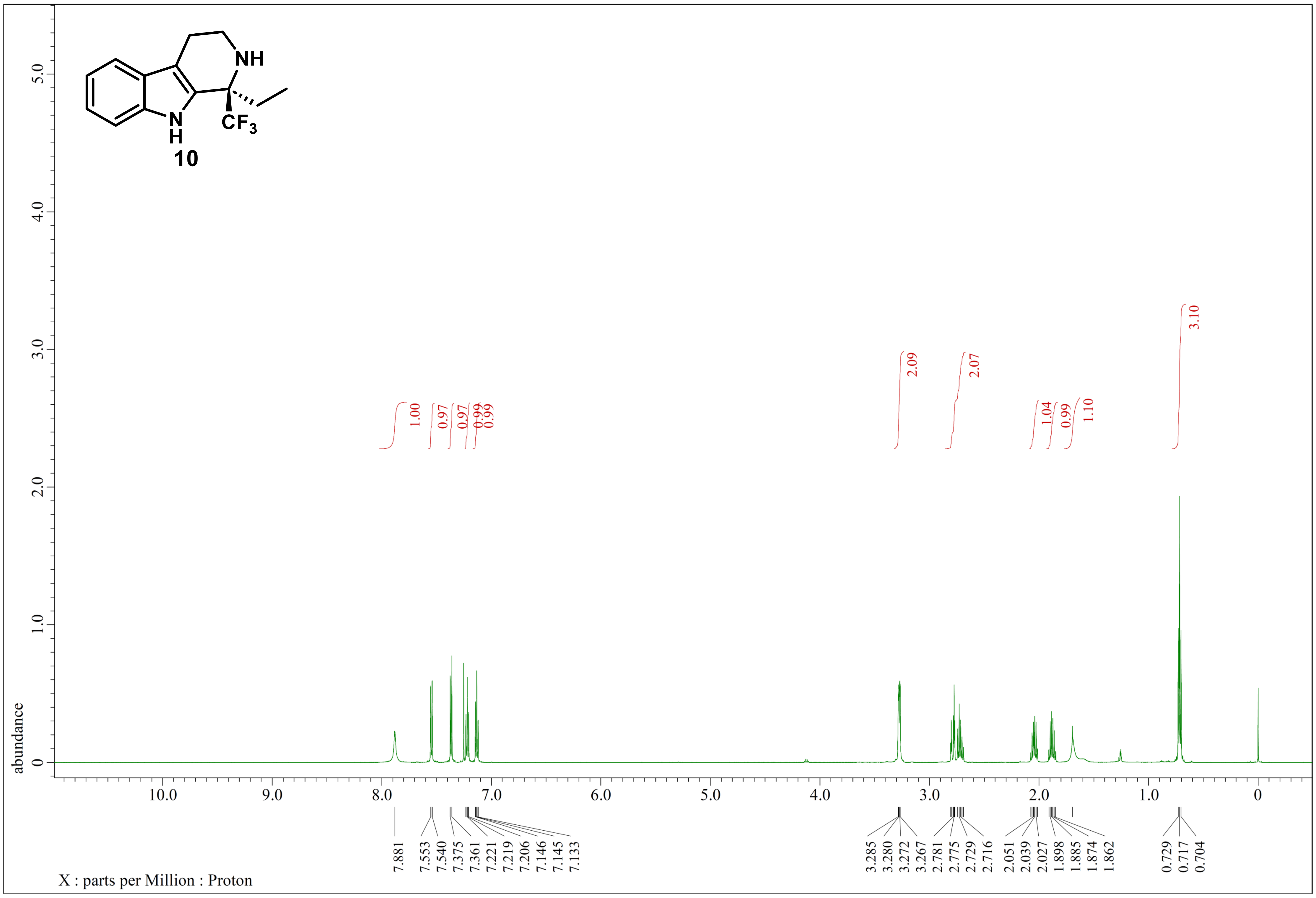

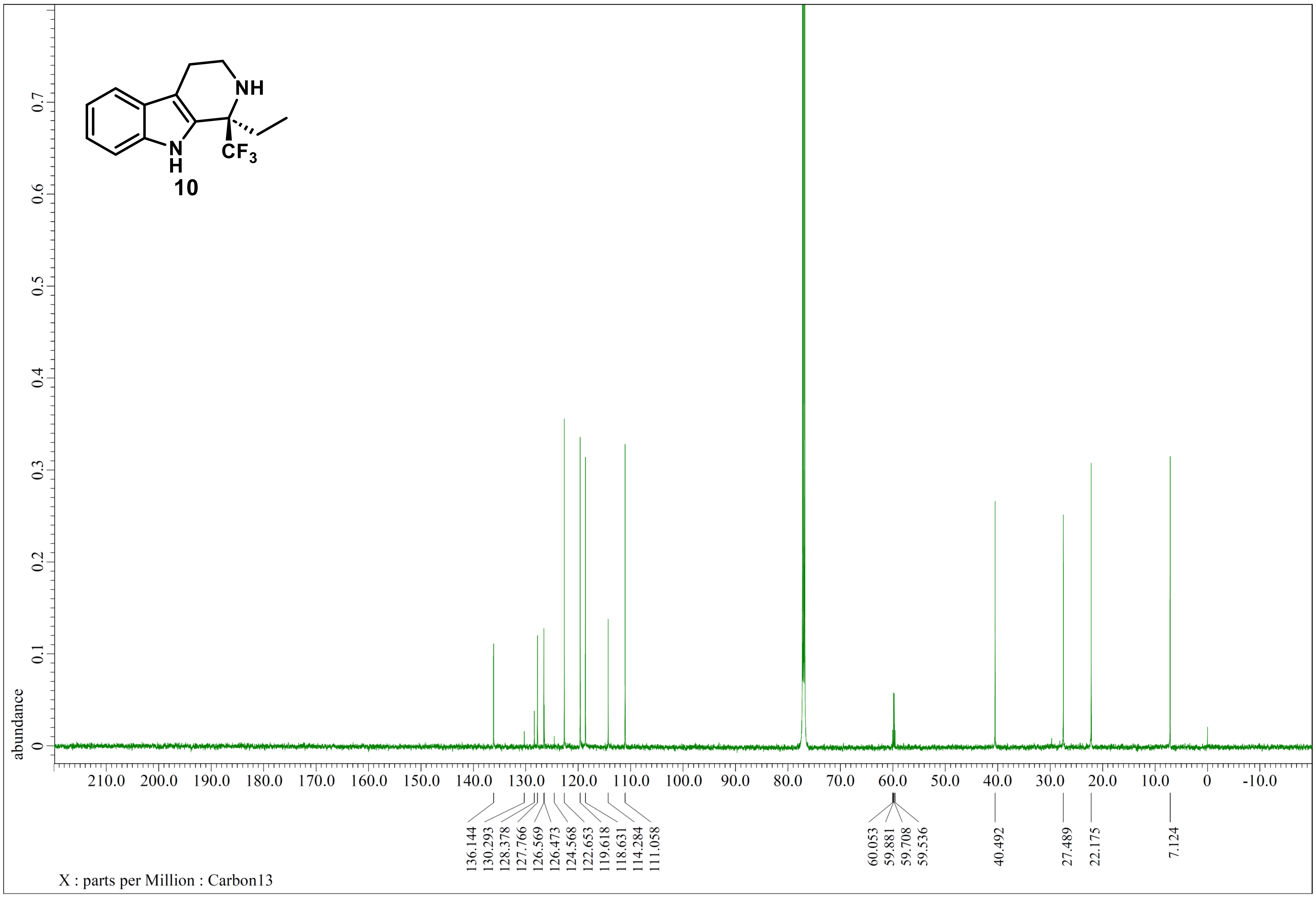
NH
N
H
CF3
10
abundance
X : parts per Million : Carbon13
136.144
130.293
128.378
127.766
126.569
126.473
124.568
122.653
119.618
118.631
114.284
111.058
60.053
59.881
59.708
59.536
40.492
27.489
22.175
7.124

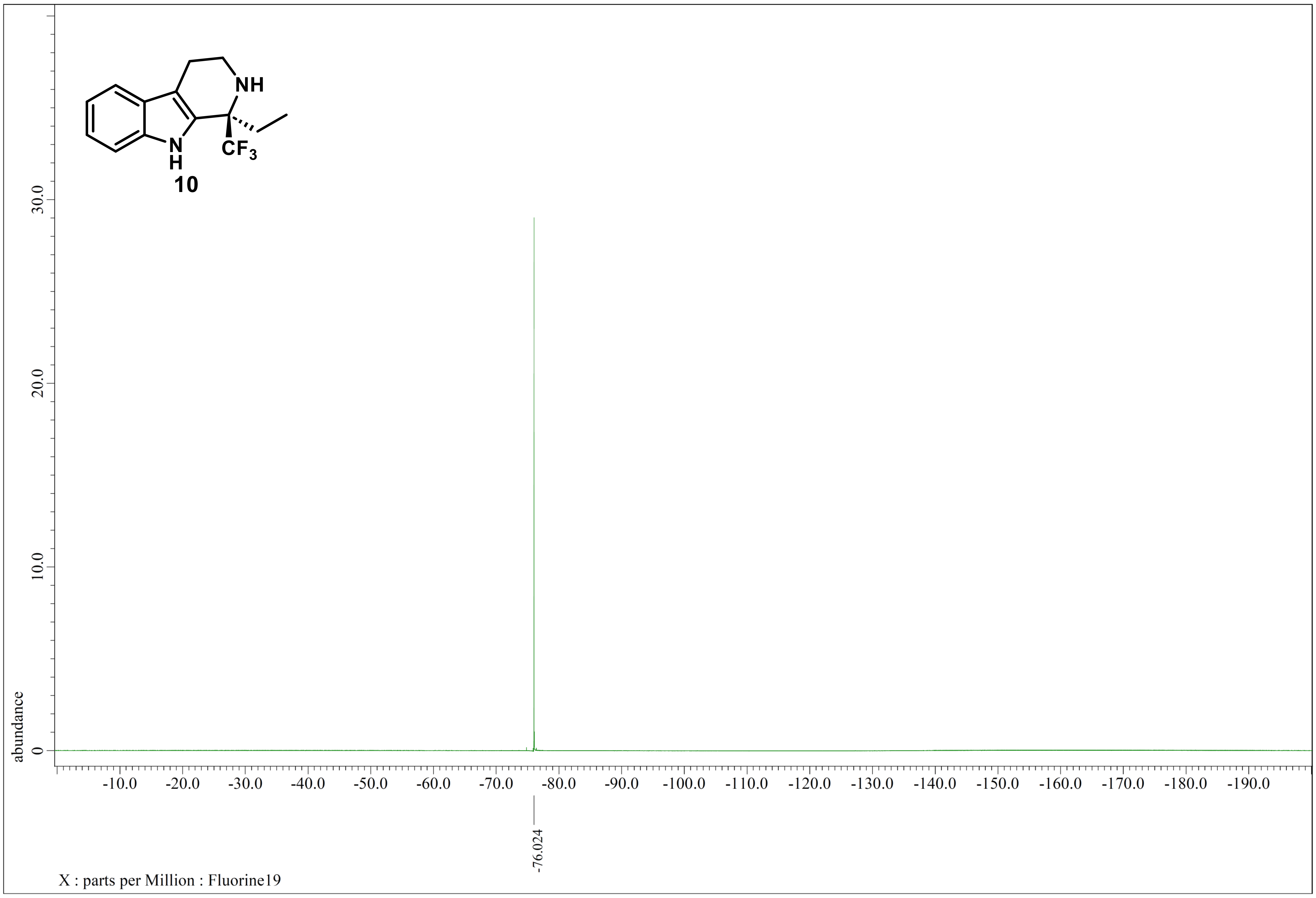
NH
N
H
CF3
10
abundance
X : parts per Million : Fluorine19
-76.024

^{1}H NMR (600 MHz, $CDCl_3$), ^{13}C NMR (151 MHz $CDCl_3$) and ^{19}F NMR (565 MHz $CDCl_3$) spectra of **11**

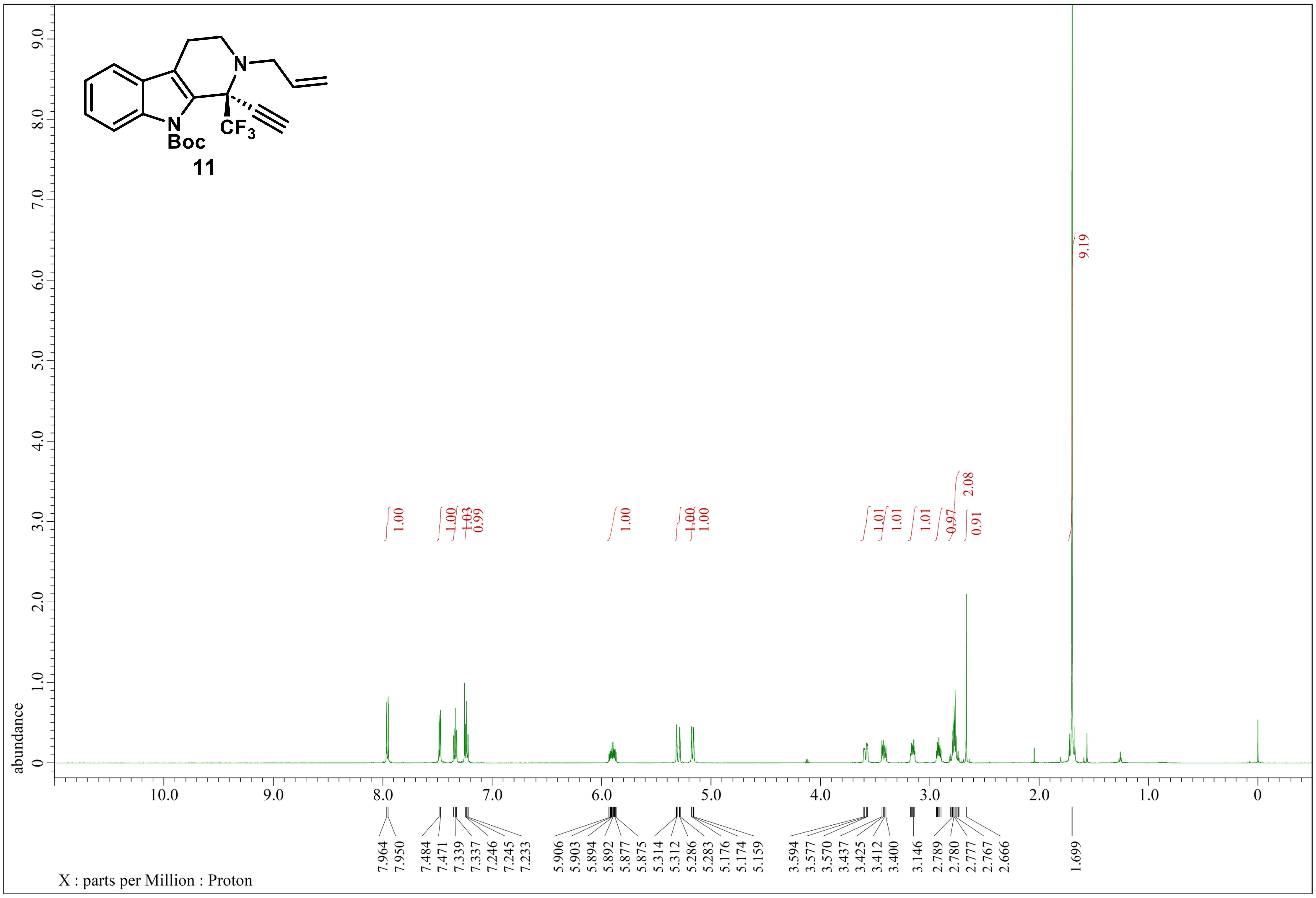

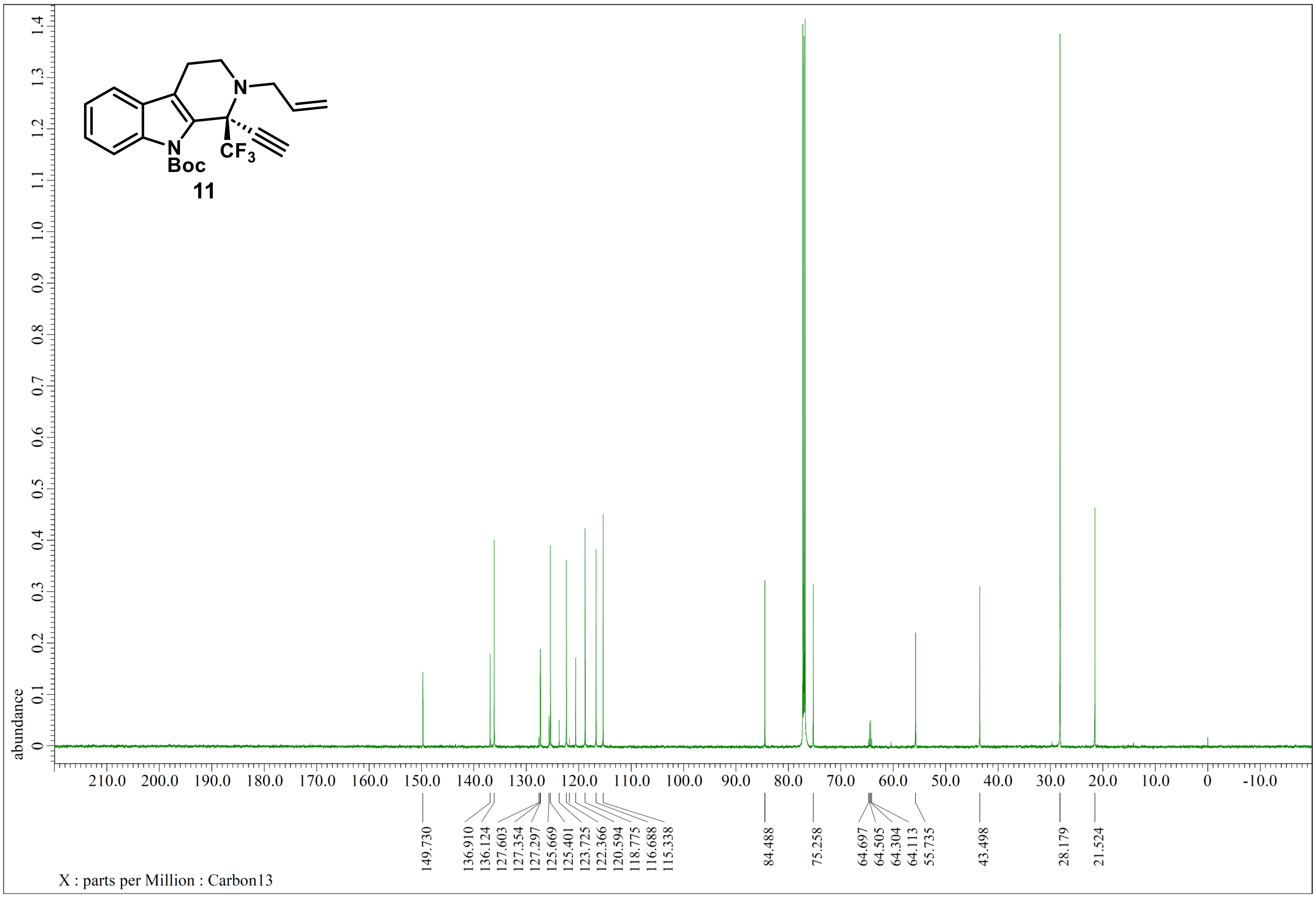

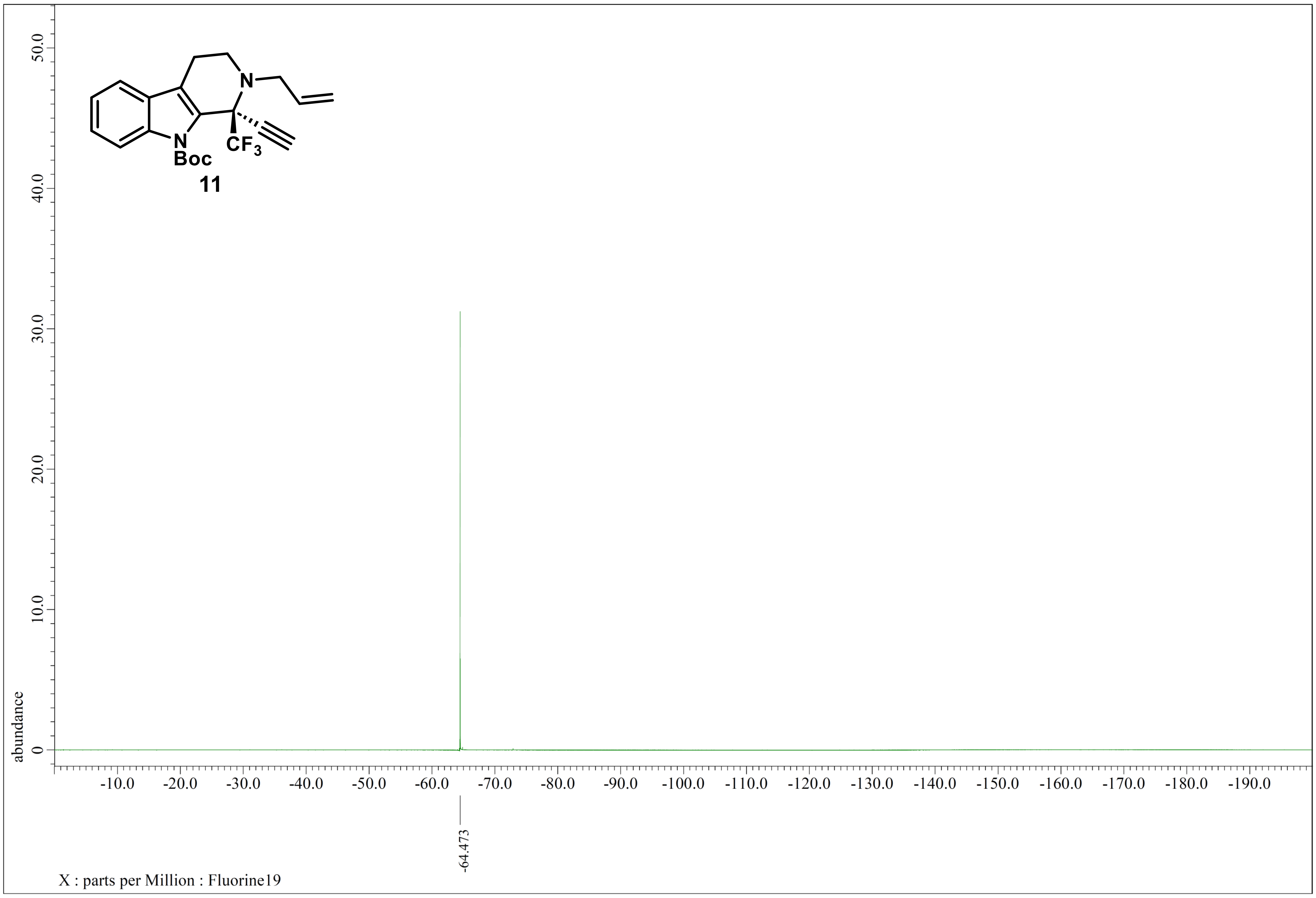

^{1}H NMR (600 MHz, $CDCl_3$), ^{13}C NMR (151 MHz $CDCl_3$) and ^{19}F NMR (565 MHz $CDCl_3$) spectra of **12**

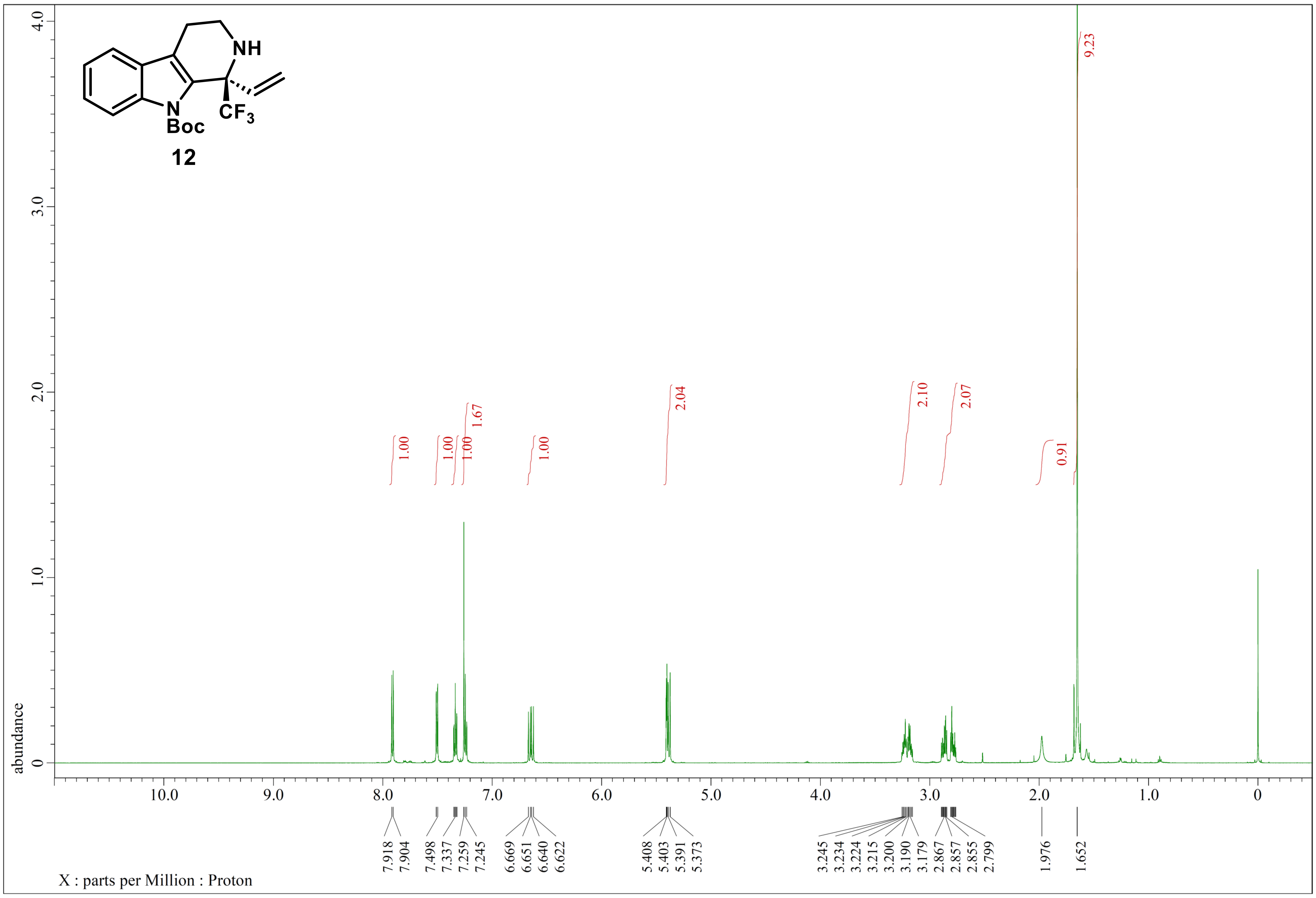

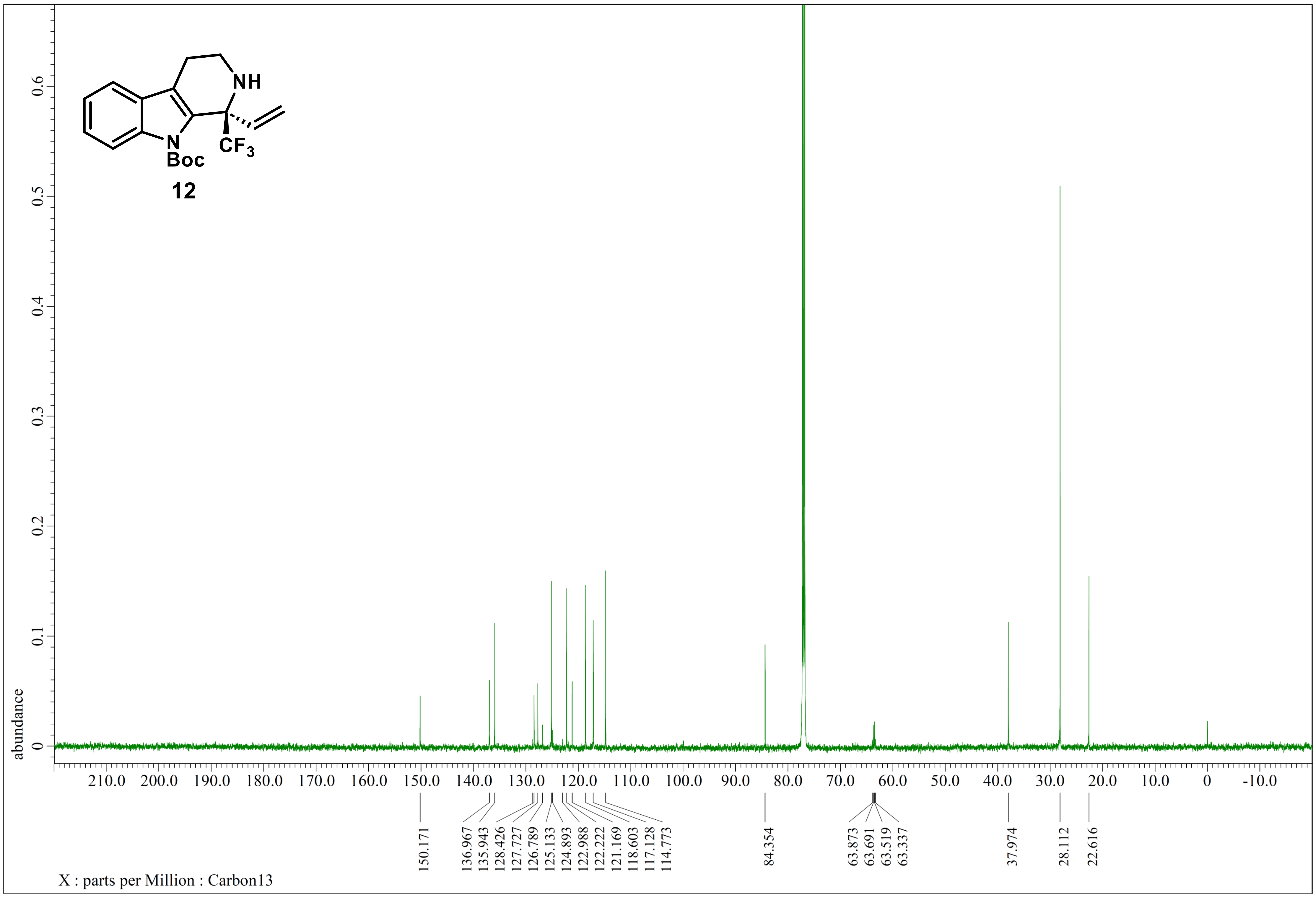

NH
N
Boc
CF_3
12
abundance
X : parts per Million : Carbon13
150.171
136.967
135.943
128.426
127.727
126.789
125.133
124.893
122.988
122.222
121.169
118.603
117.128
114.773
84.354
63.873
63.691
63.519
63.337
37.974
28.112
22.616

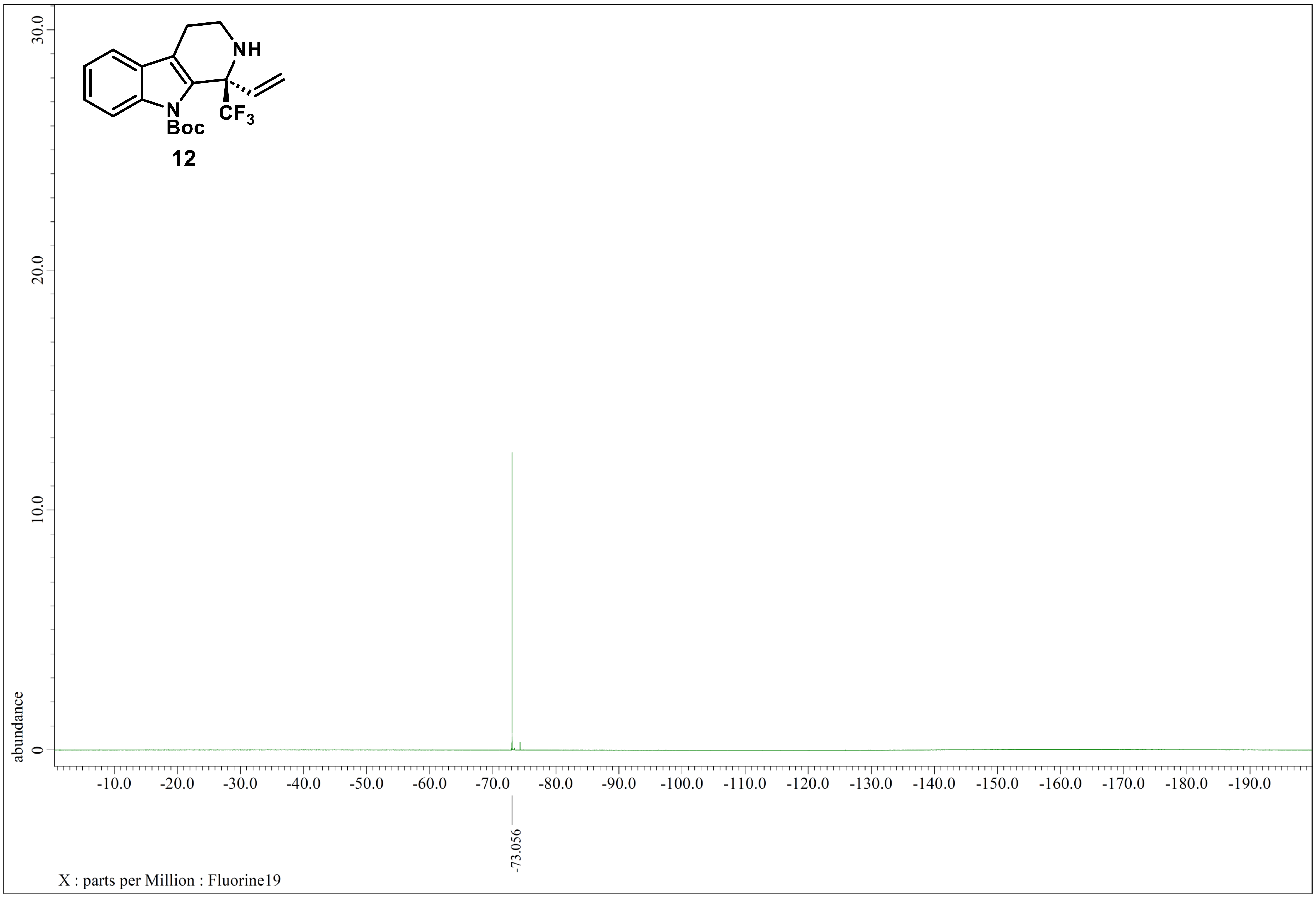

NH
N
Boc
CF_3
12
abundance
X : parts per Million : Fluorine19
-73.056

^1H NMR (600 MHz, $CDCl_3$), ^{13}C NMR (151 MHz $CDCl_3$) and ^{19}F NMR (565 MHz $CDCl_3$) spectra of **13**

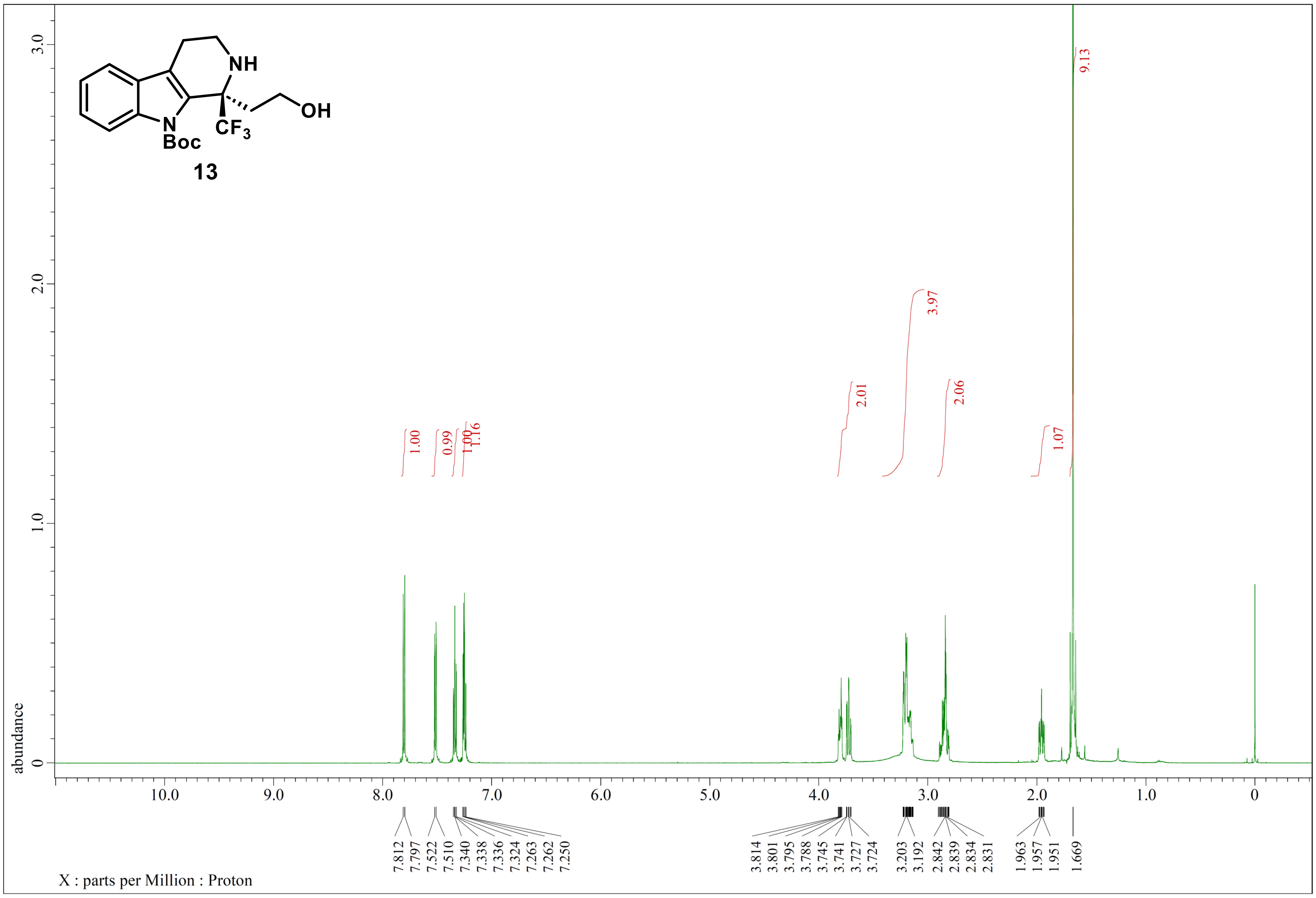

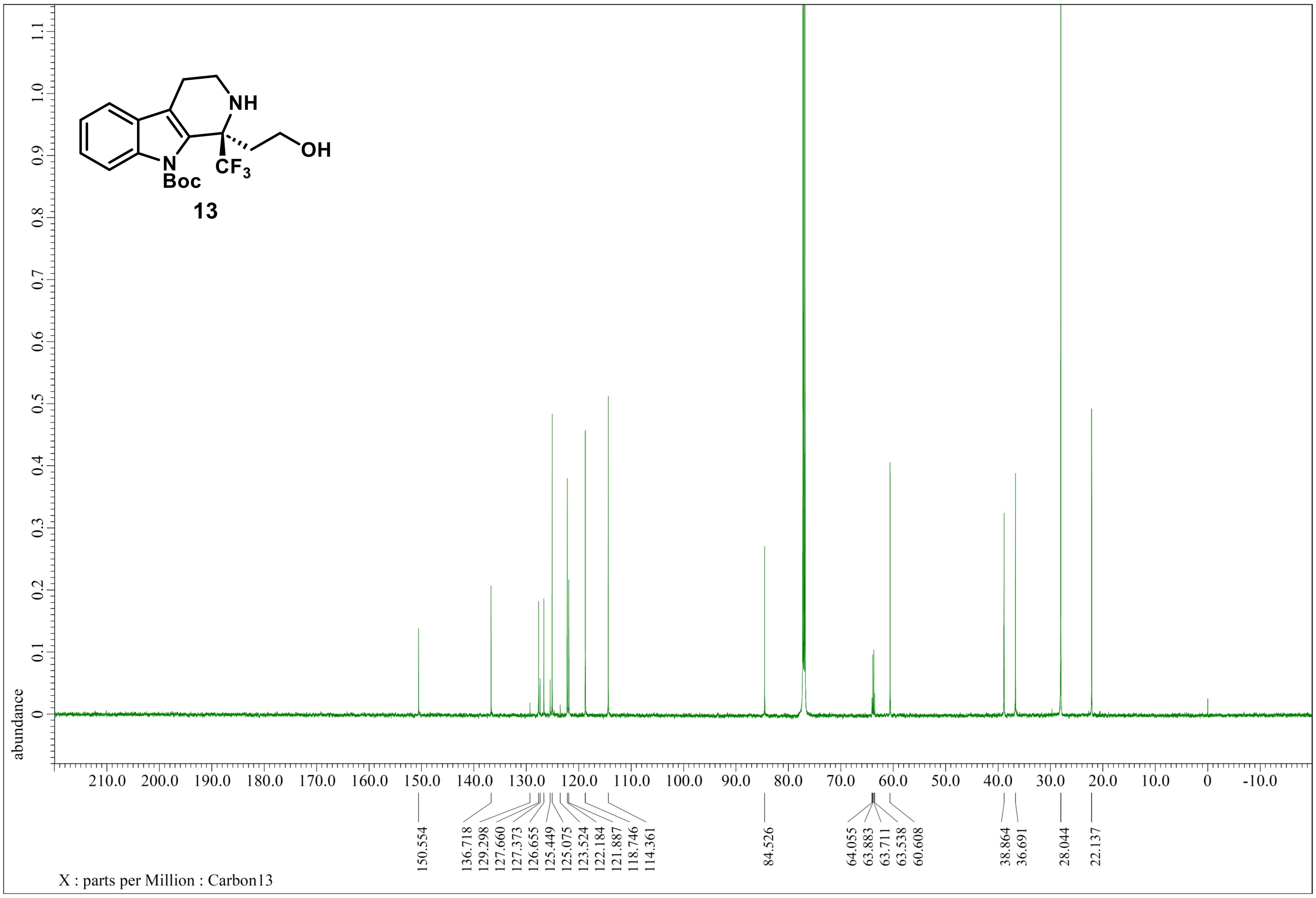

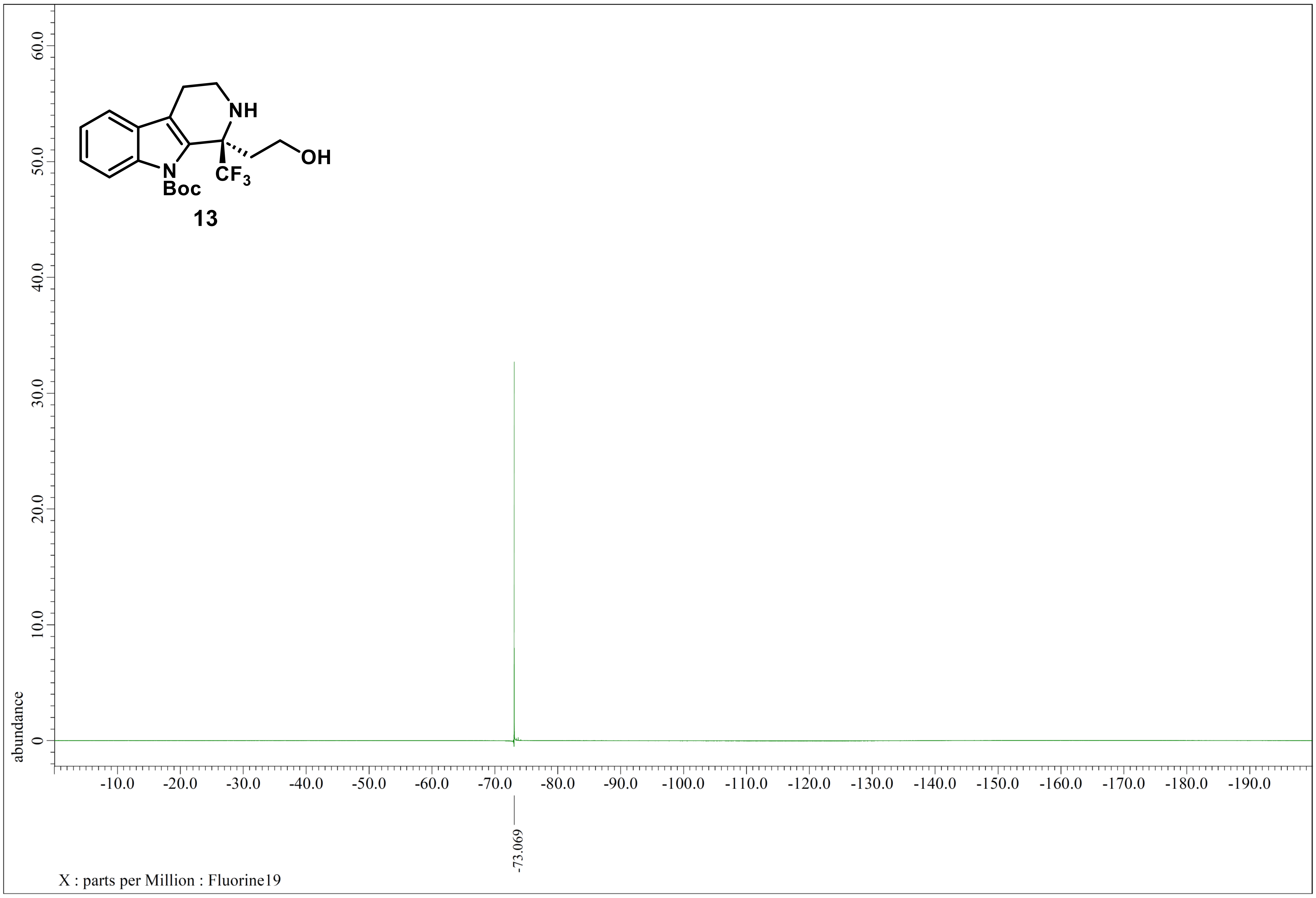

^{1}H NMR (600 MHz, $CDCl_3$), ^{13}C NMR (151 MHz $CDCl_3$) and ^{19}F NMR (565 MHz $CDCl_3$) spectra of **S6**

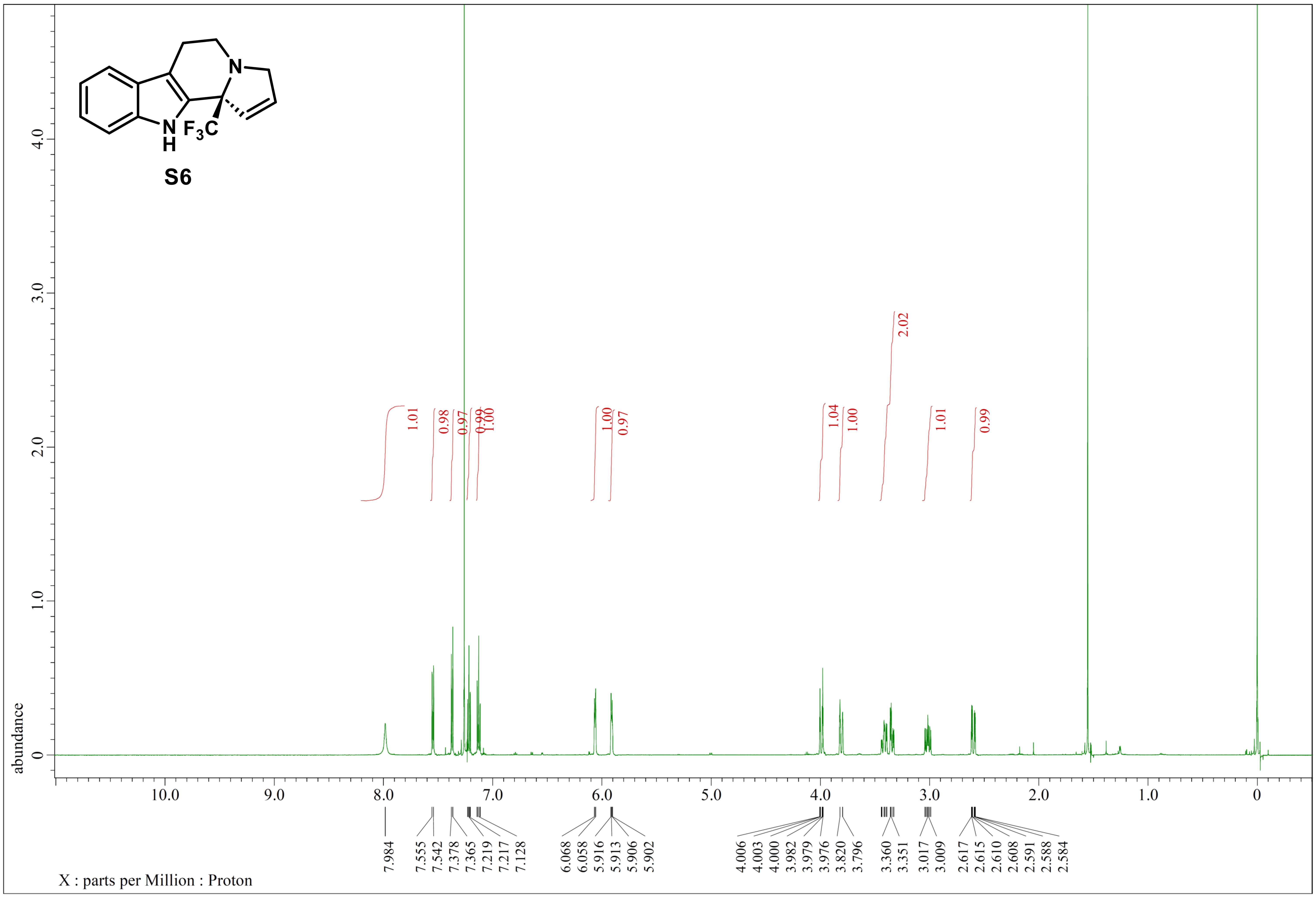

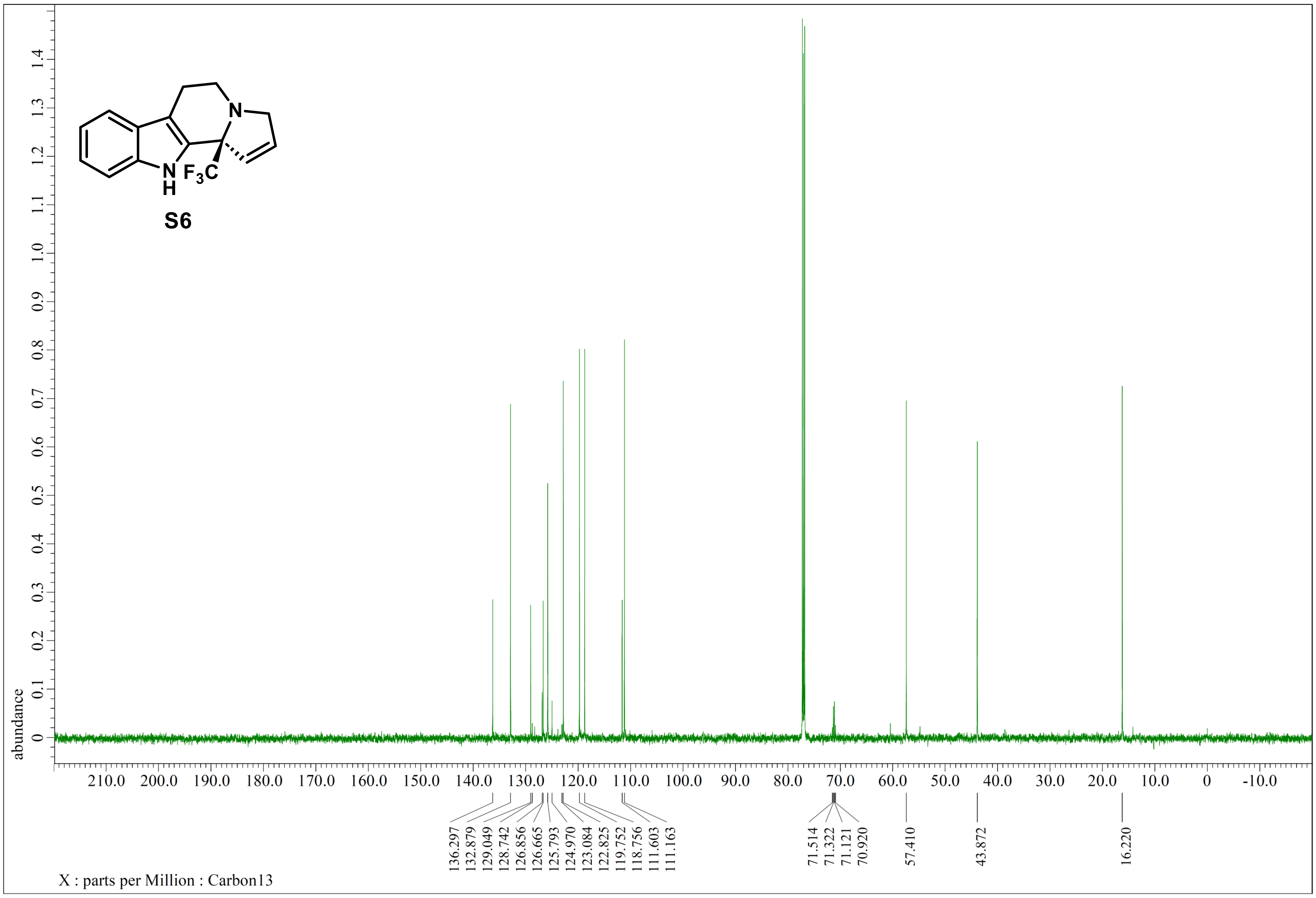
abundance
X : parts per Million : Carbon13
136.297
132.879
129.049
128.742
126.856
126.665
125.793
124.970
123.084
122.825
119.752
118.756
111.603
111.163
71.514
71.322
71.121
70.920
57.410
43.872
16.220

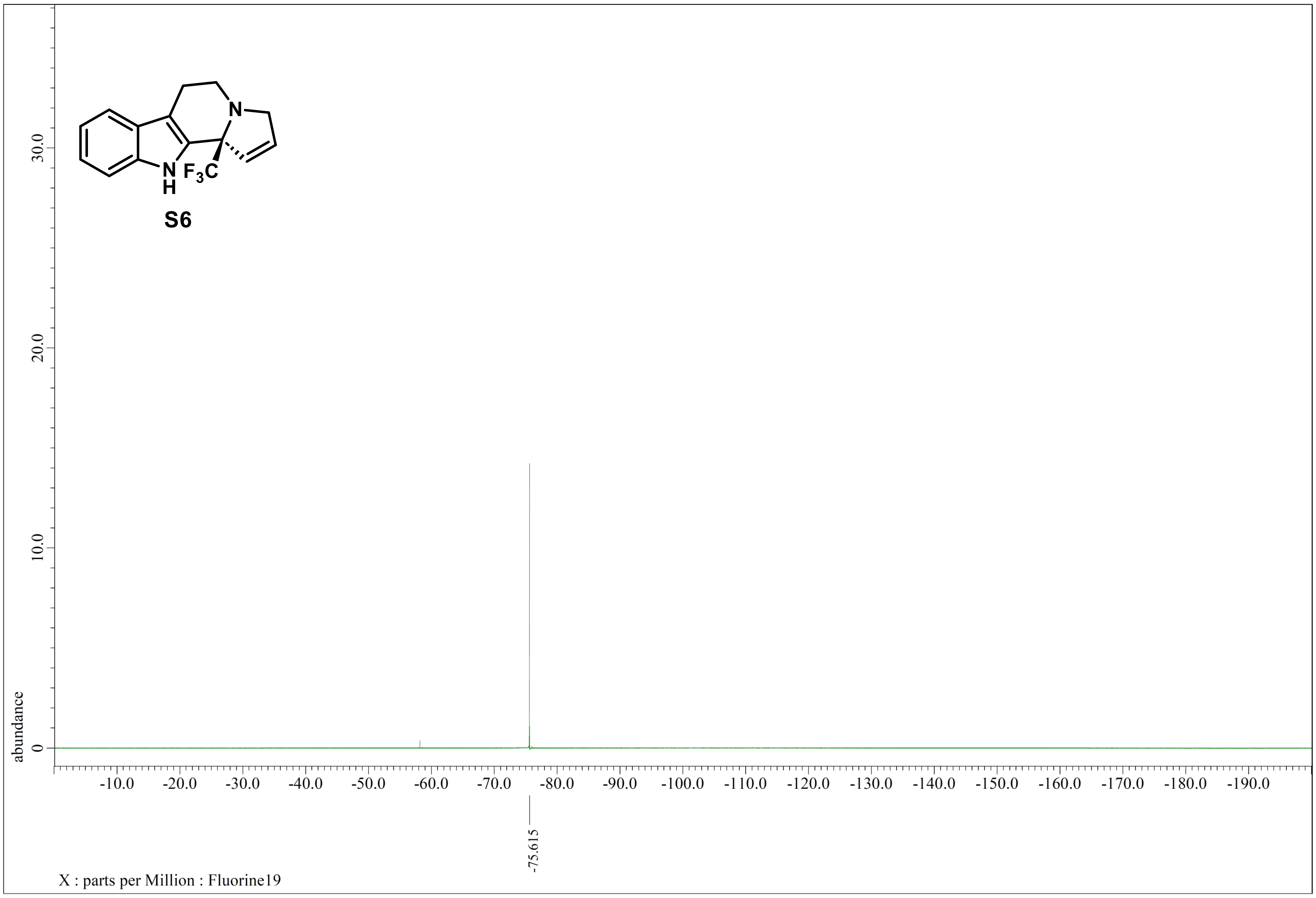
abundance
X : parts per Million : Fluorine19
-75.615

^1H NMR (600 MHz, $CDCl_3$), ^{13}C NMR (151 MHz $CDCl_3$) and ^{19}F NMR (565 MHz $CDCl_3$) spectra of **15**

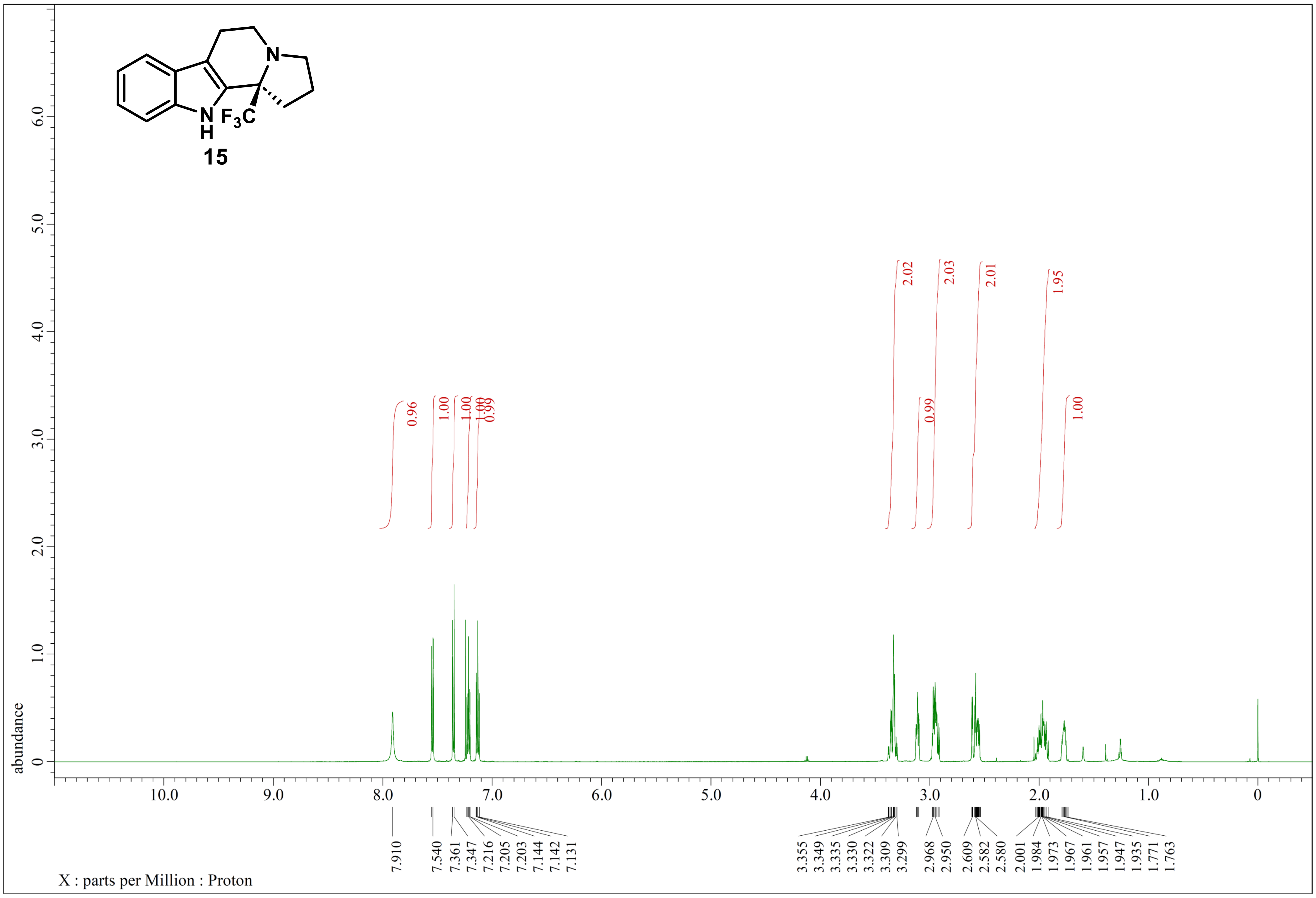

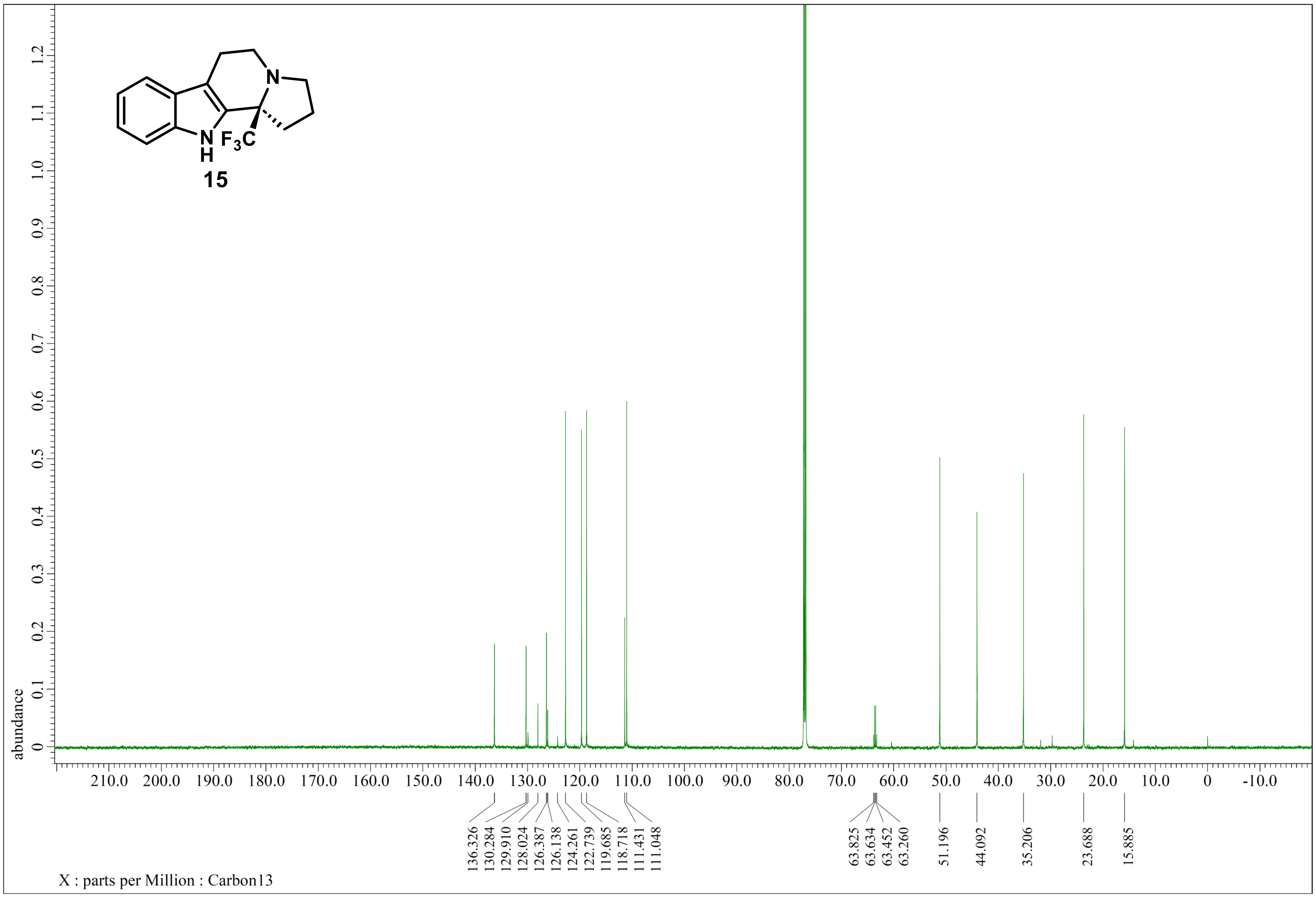

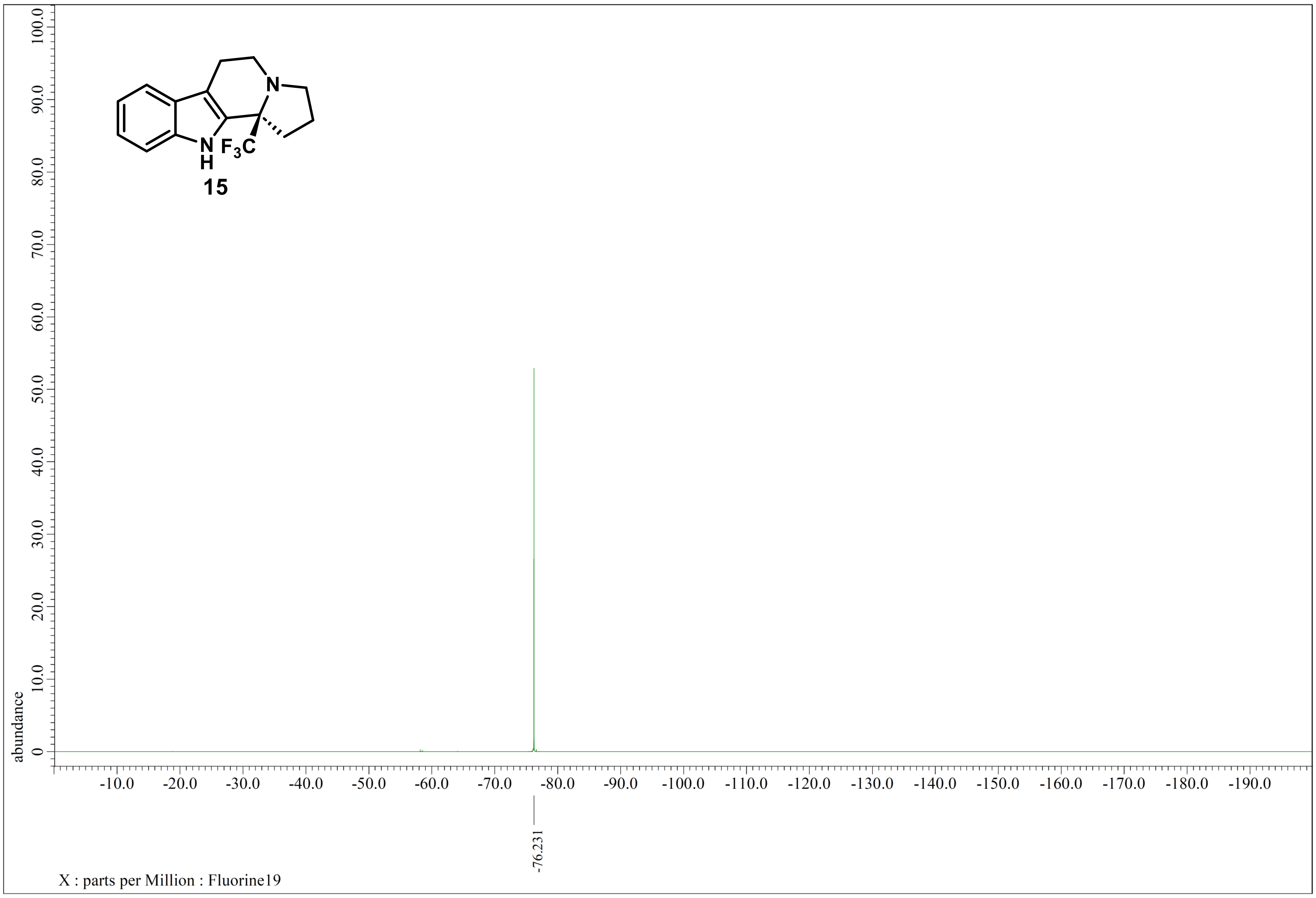

N
H
F_3C
N
15
abundance
X : parts per Million : Fluorine19
-76.231

HPLC Charts

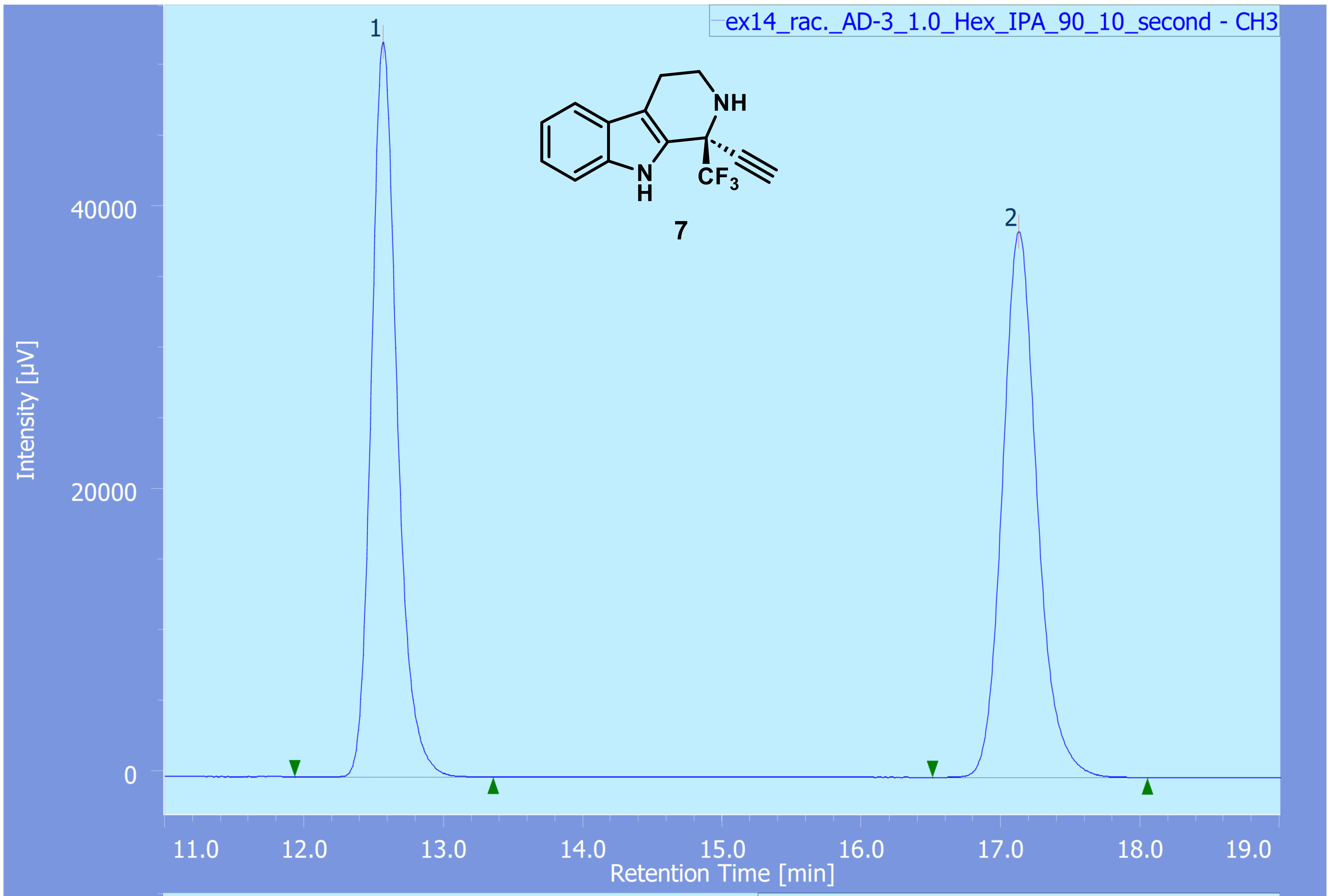

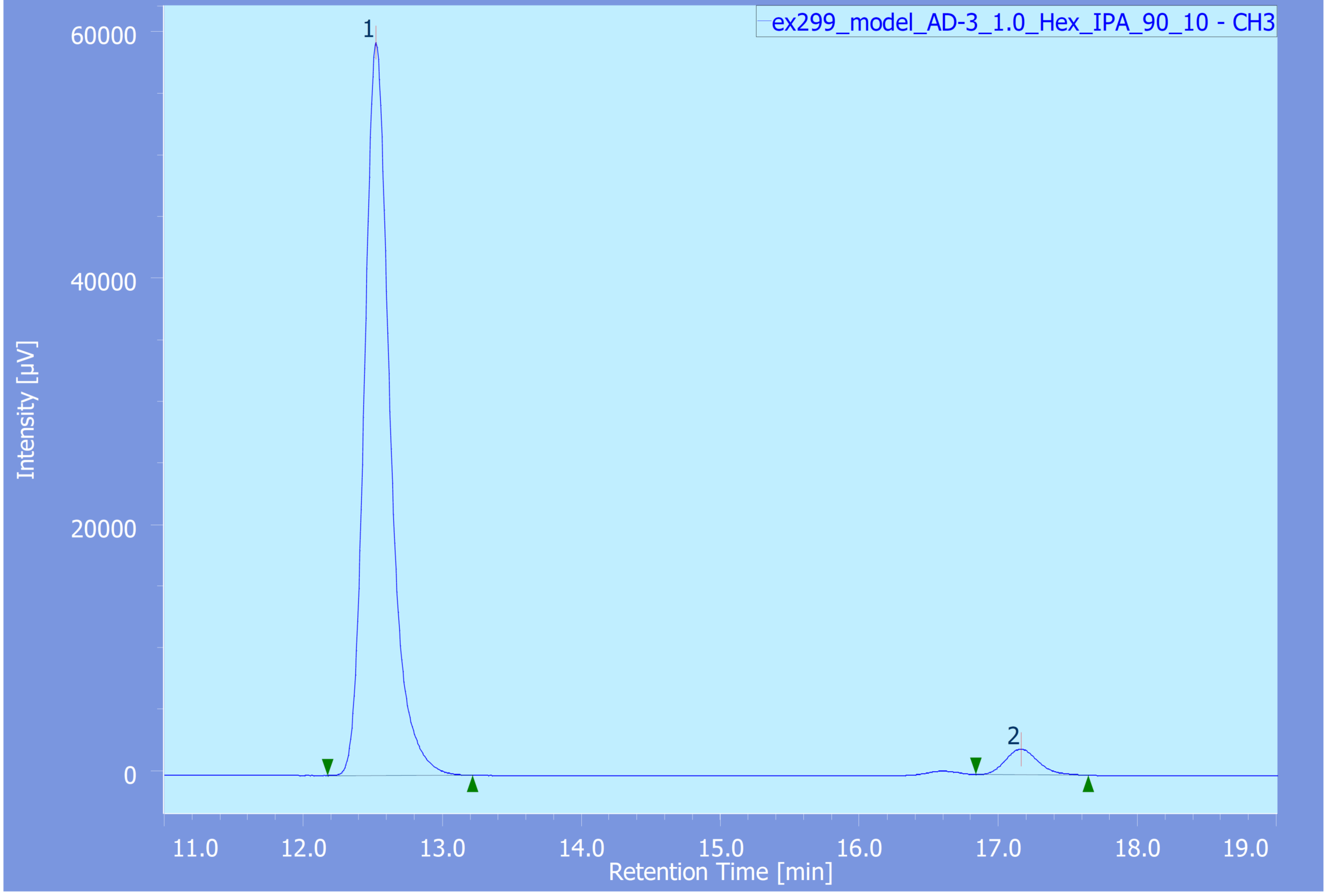

	Retention time (1)	Retention time (2)	% area (1)	% area (2)
rac-**4ac**	12.567	17.125	49.946	50.054
**4ac**	12.525	17.158	95.683	4.317

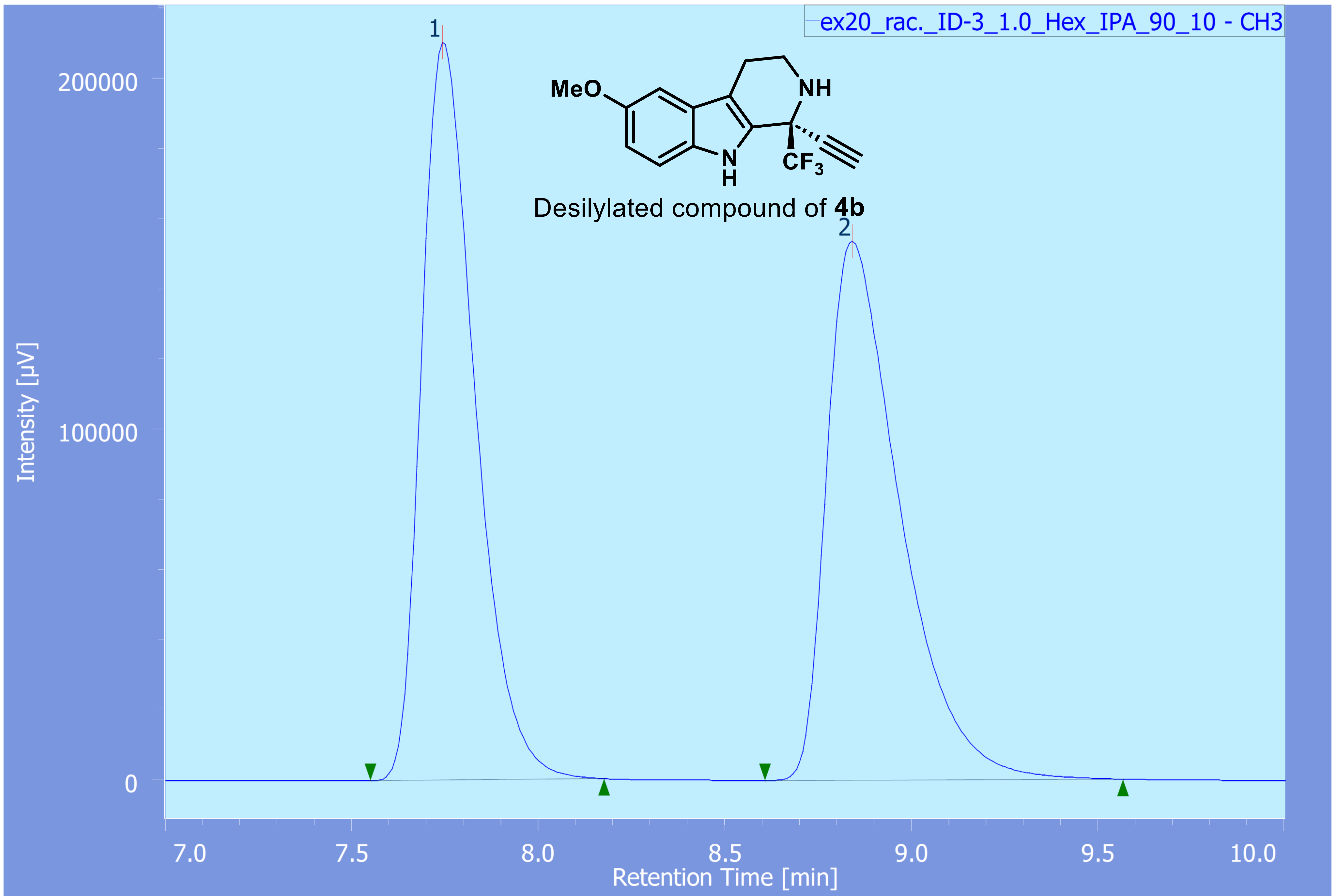

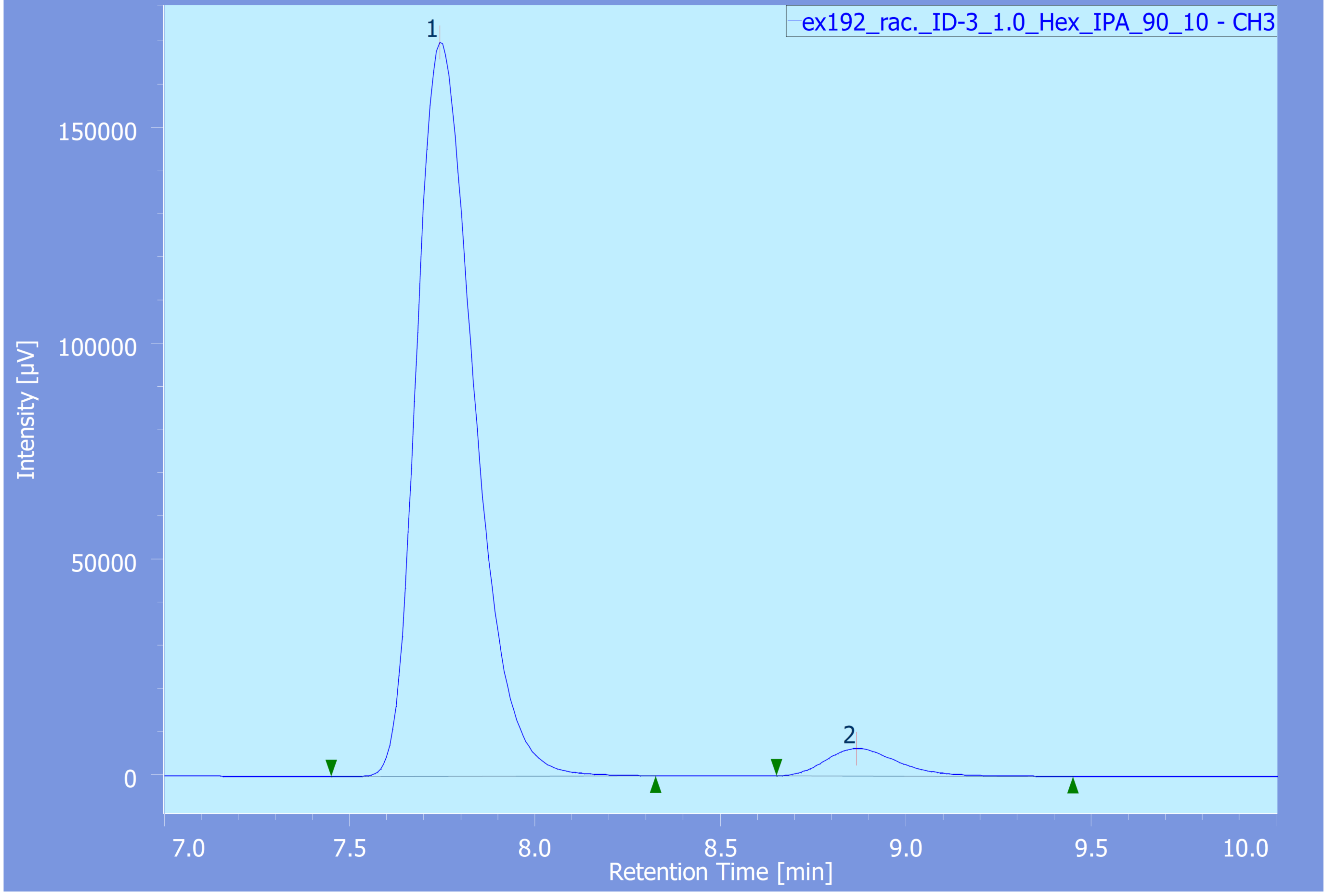

	Retention time (1)	Retention time (2)	% area (1)	% area (2)
rac-**4b**	7.742	8.842	50.040	49.960
**4b**	7.742	8.867	95.496	4.504

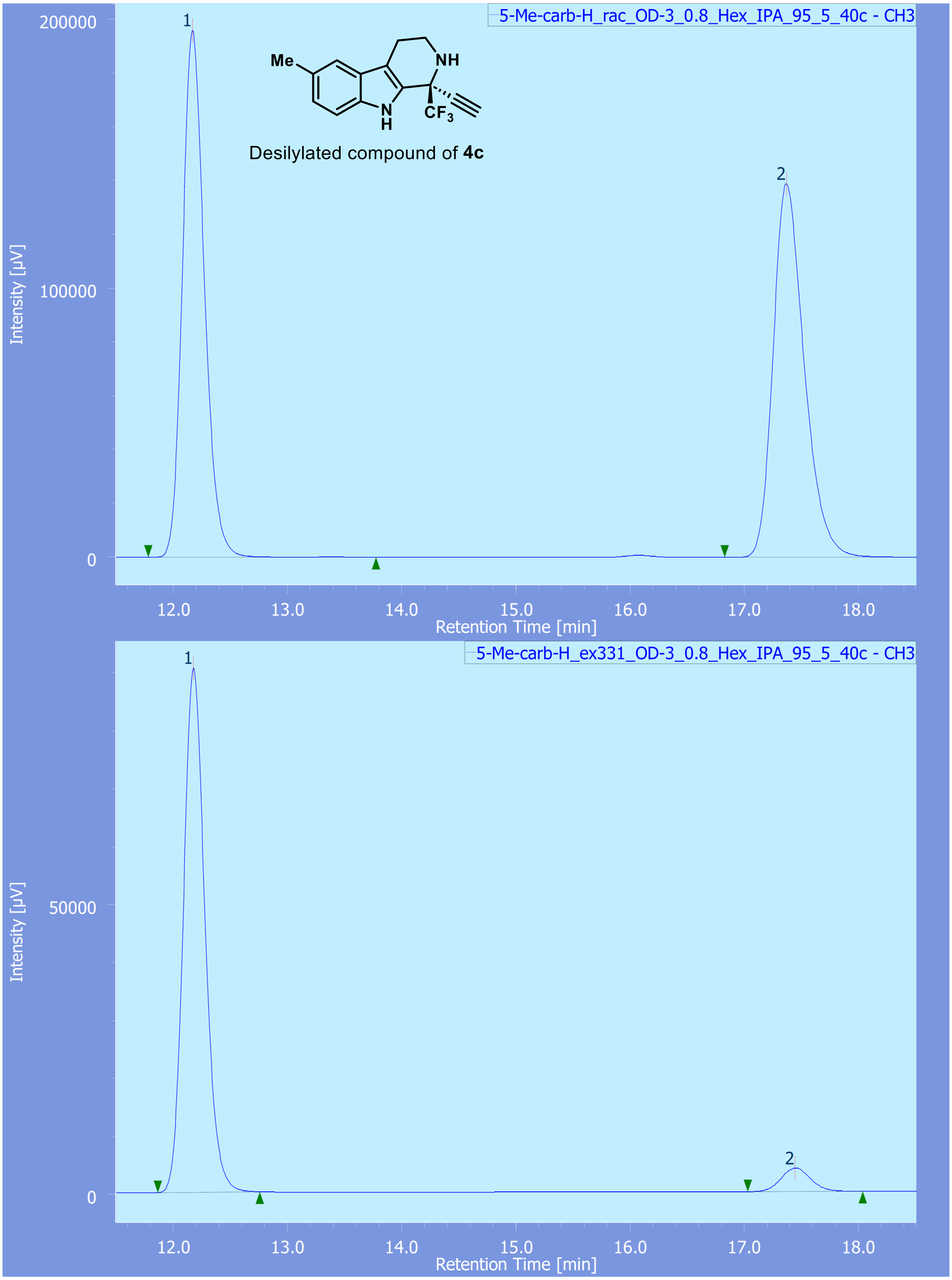

	Retention time (1)	Retention time (2)	% area (1)	% area (2)
rac-**4c**	12.167	17.367	49.906	50.094
**4c**	12.175	17.442	94.110	5.890

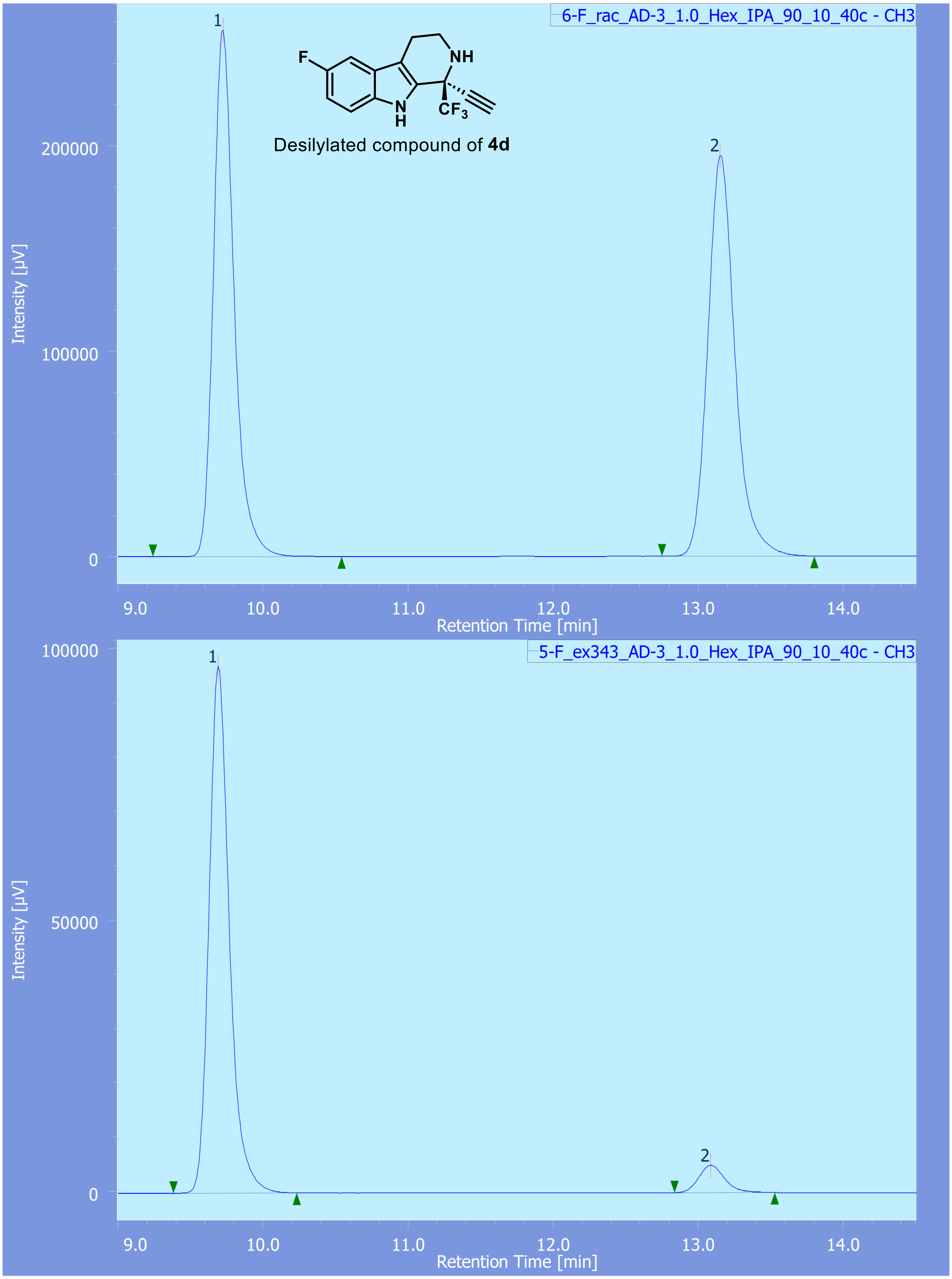

	Retention time (1)	Retention time (2)	% area (1)	% area (2)
rac-**4d**	9.725	13.150	49.965	50.035
**4d**	9.692	13.083	93.683	6.317

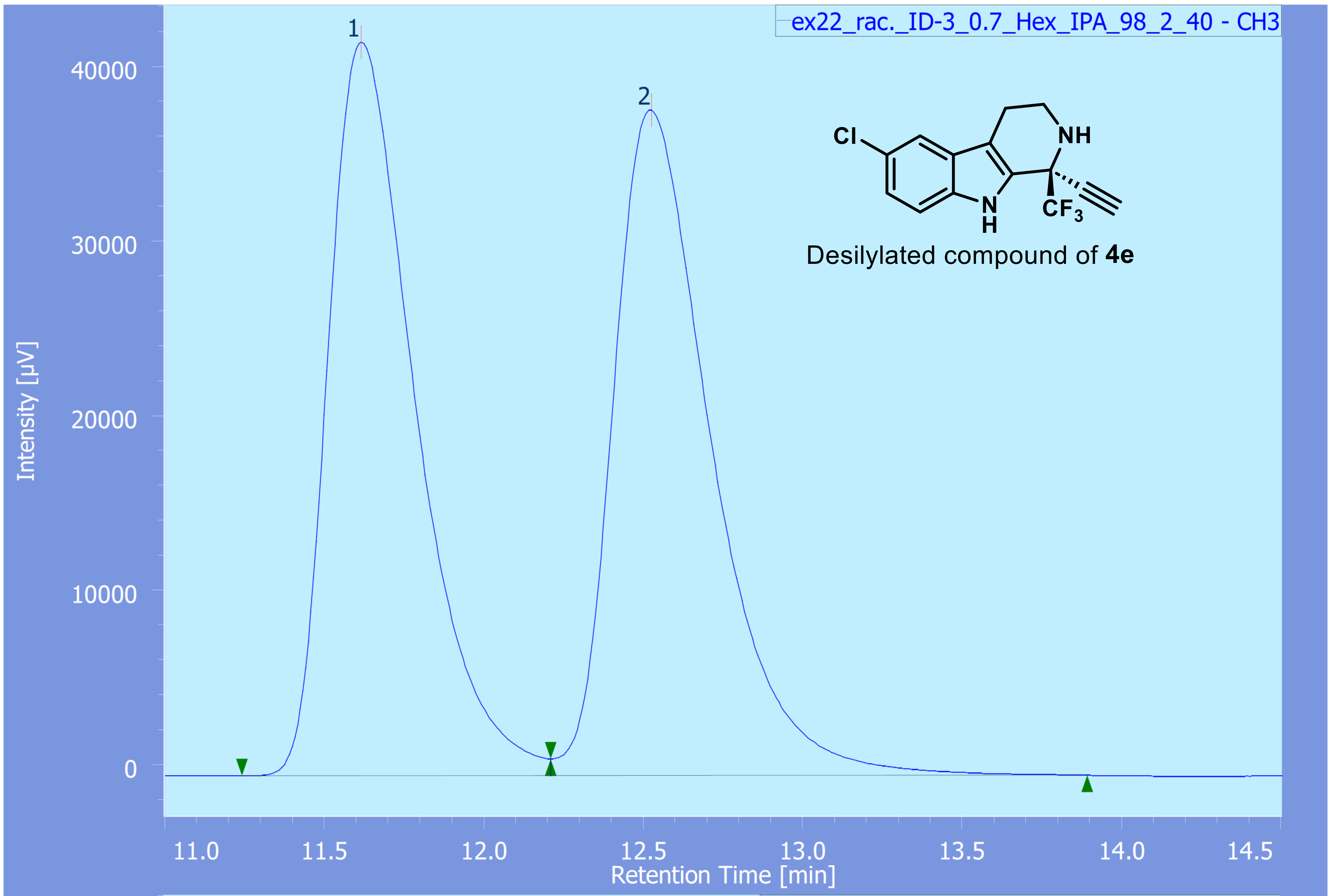

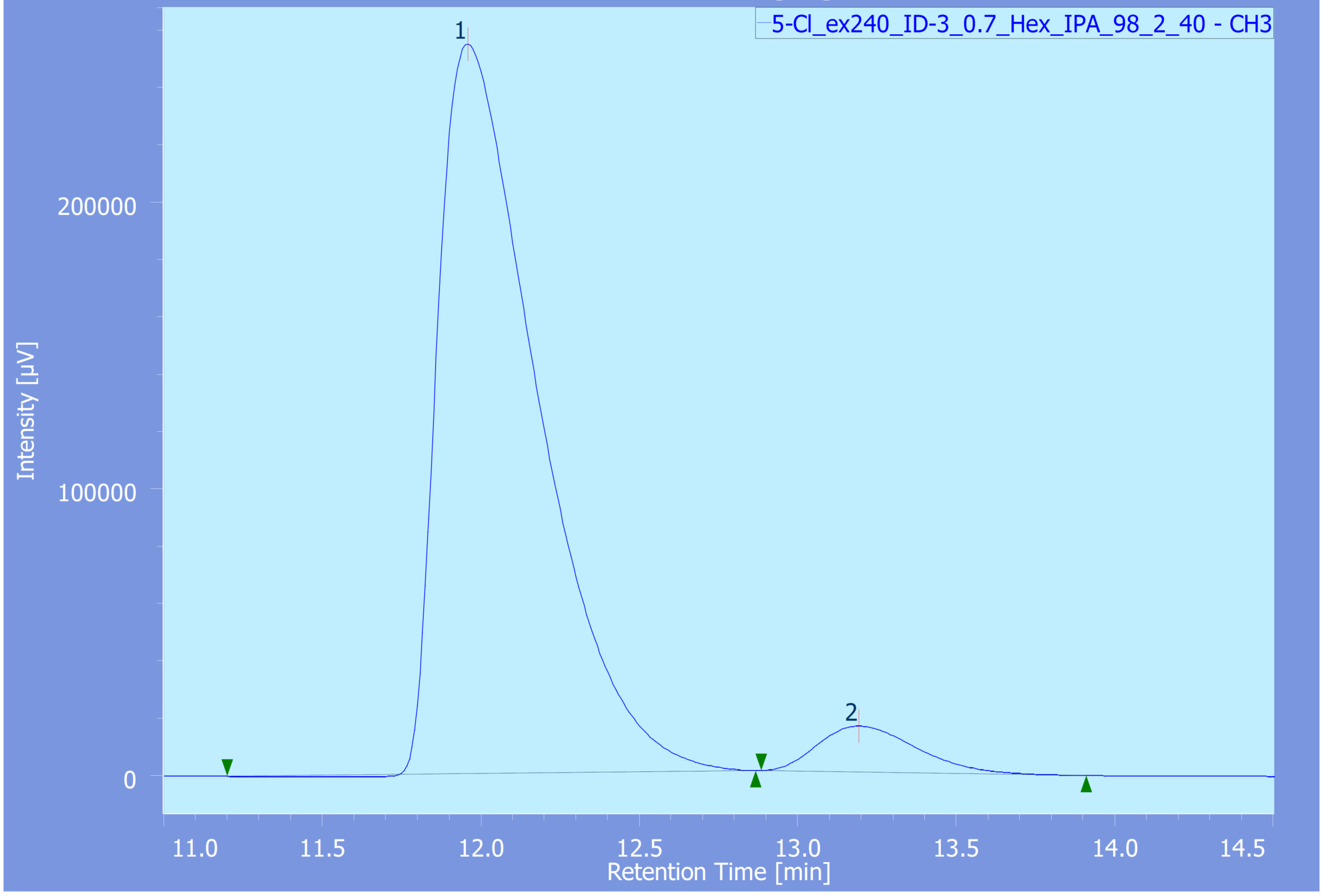

	Retention time (1)	Retention time (2)	% area (1)	% area (2)
rac-**4e**	11.617	12.525	49.396	50.604
**4e**	11.958	13.192	94.137	5.863

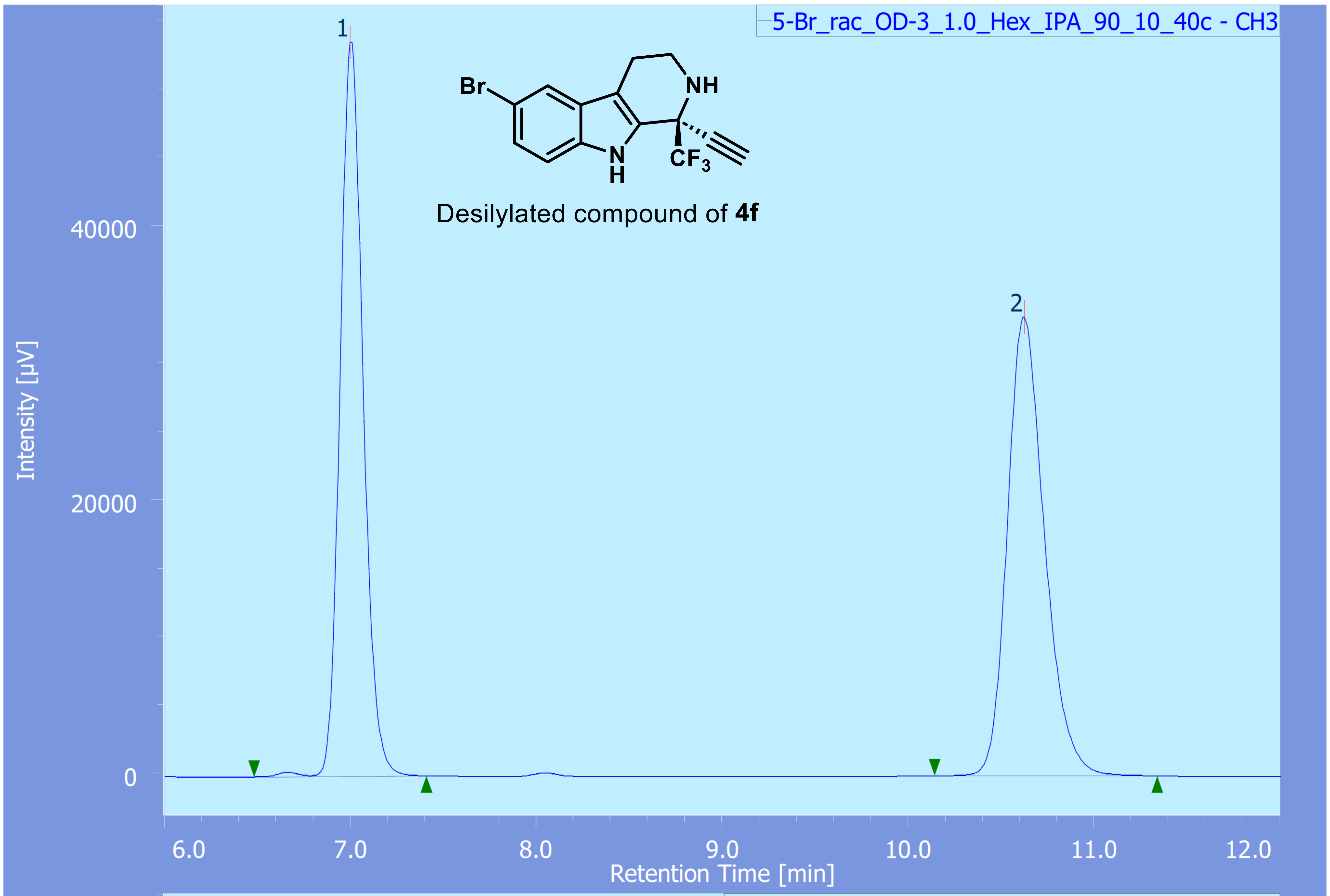

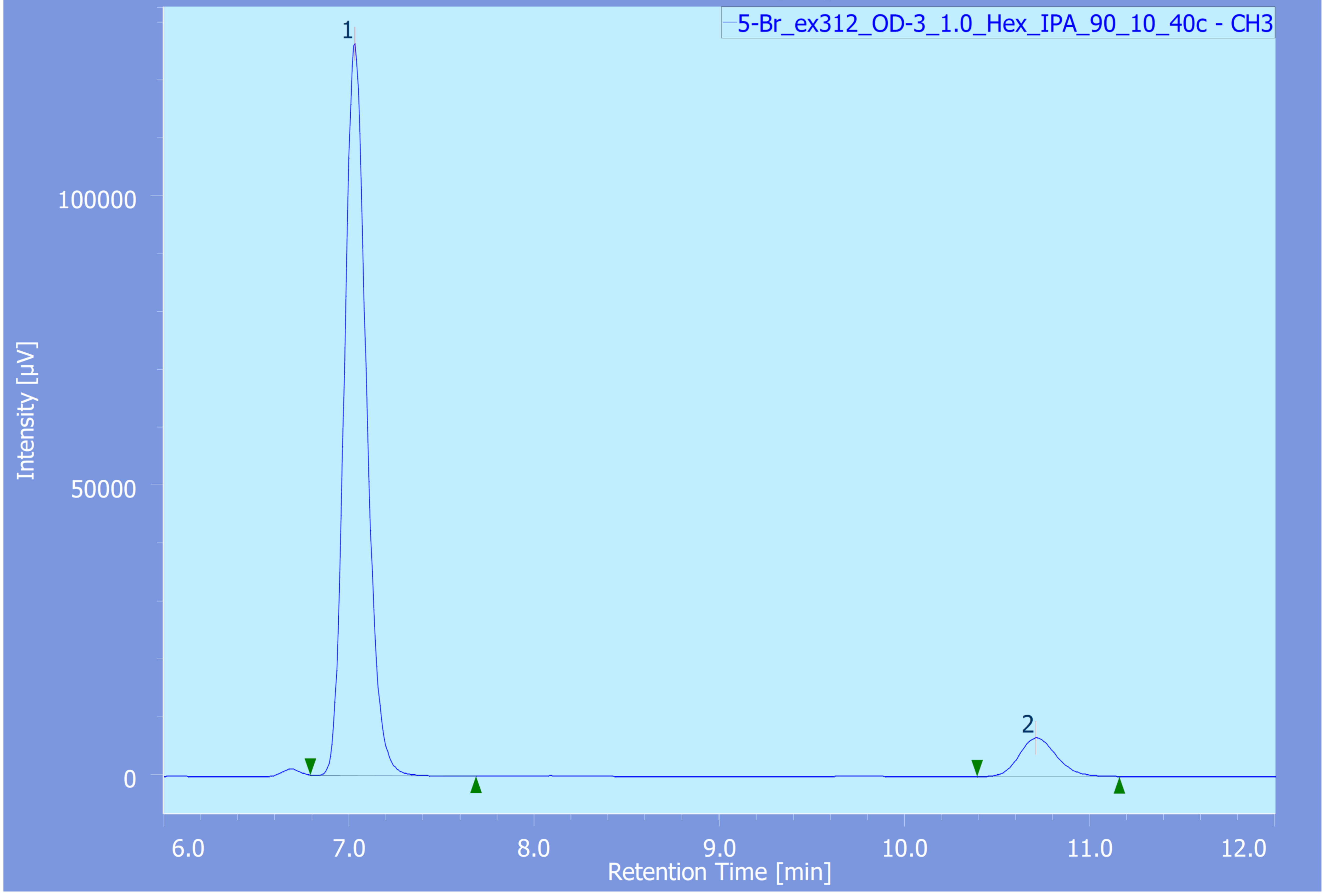

	Retention time (1)	Retention time (2)	% area (1)	% area (2)
rac-**4f**	7.000	10.625	49.863	50.137
**4f**	7.033	10.708	91.792	8.208

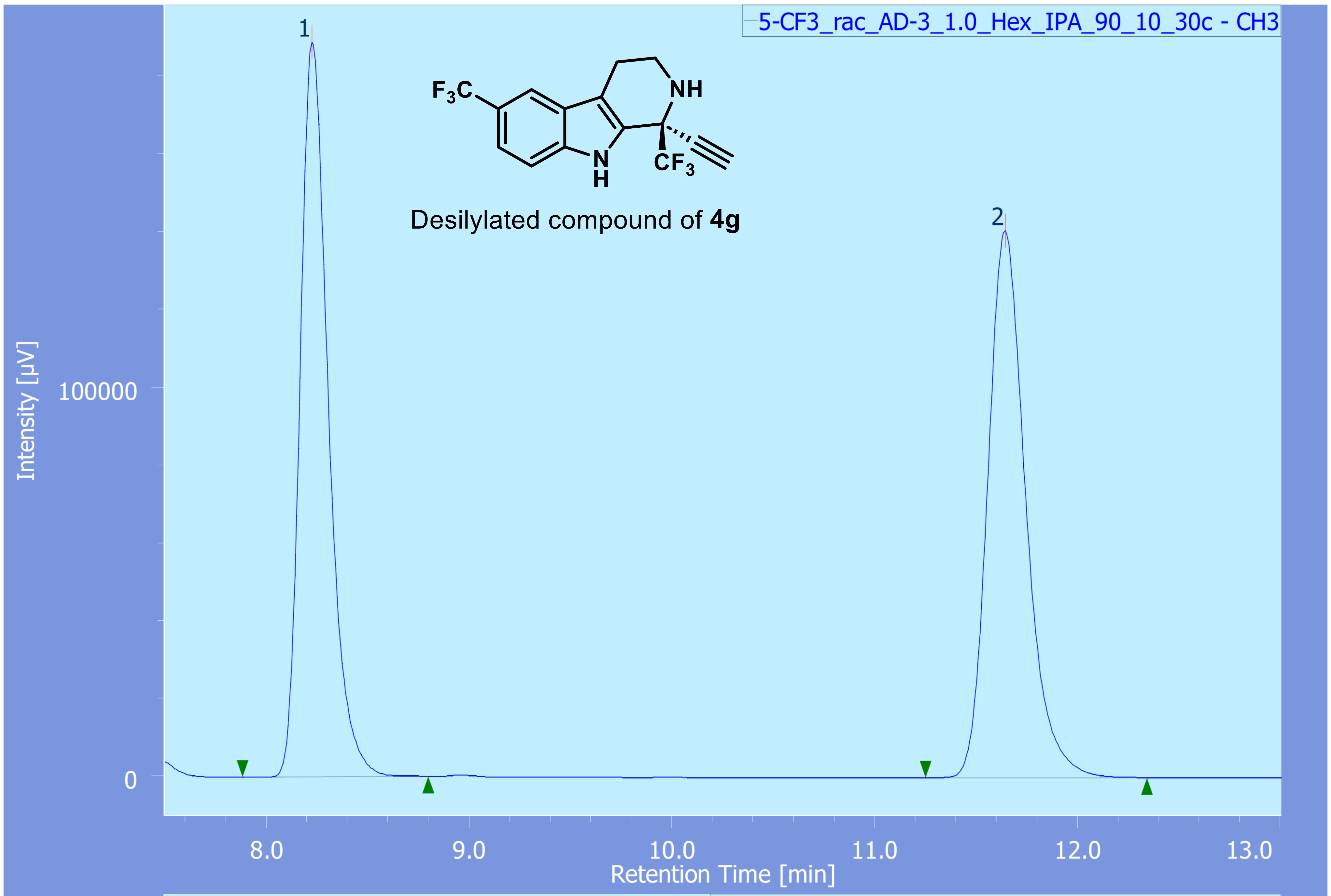

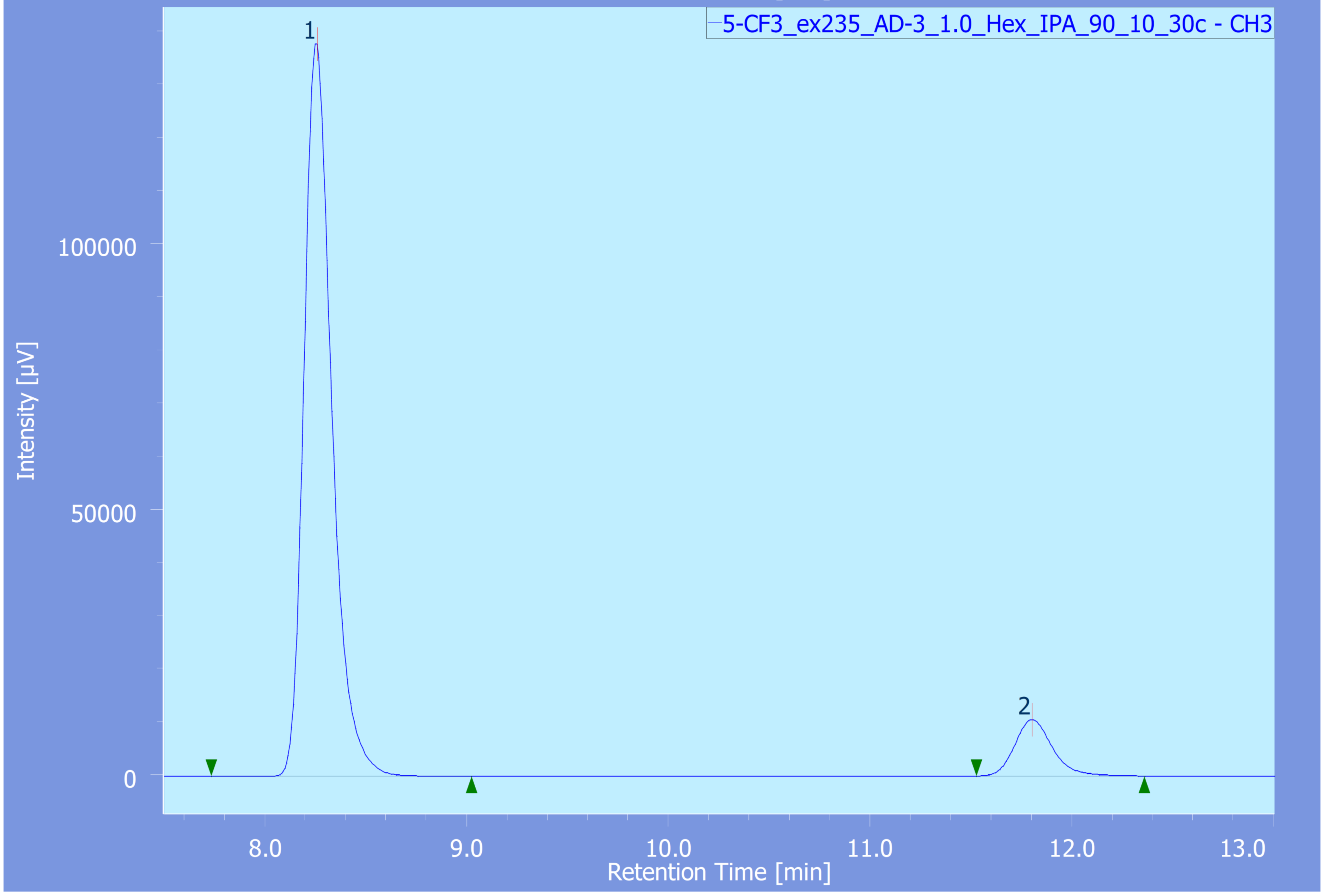

	Retention time (1)	Retention time (2)	% area (1)	% area (2)
rac-**4g**	8.225	11.642	49.935	50.065
**4g**	8.258	11.800	90.582	9.418

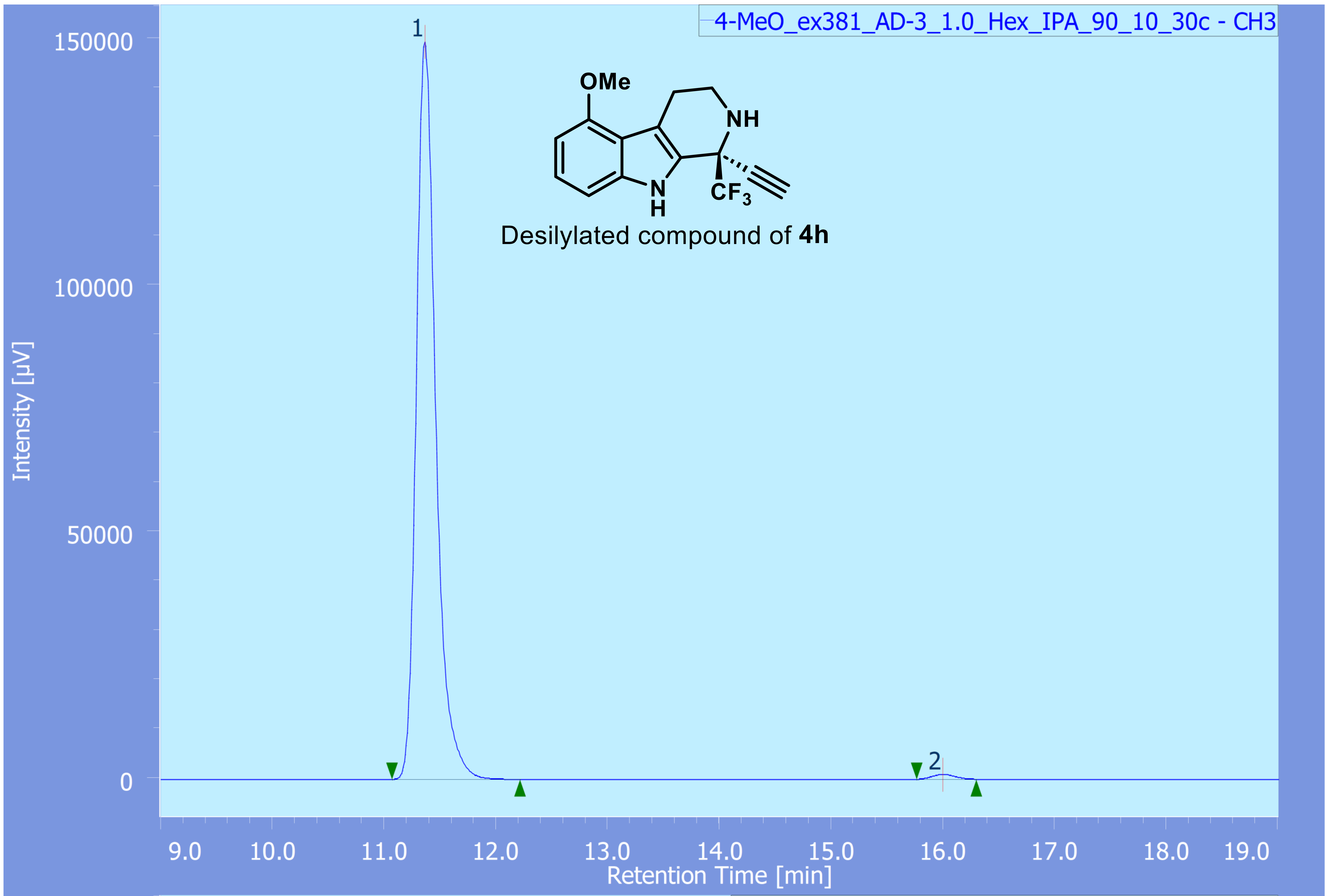

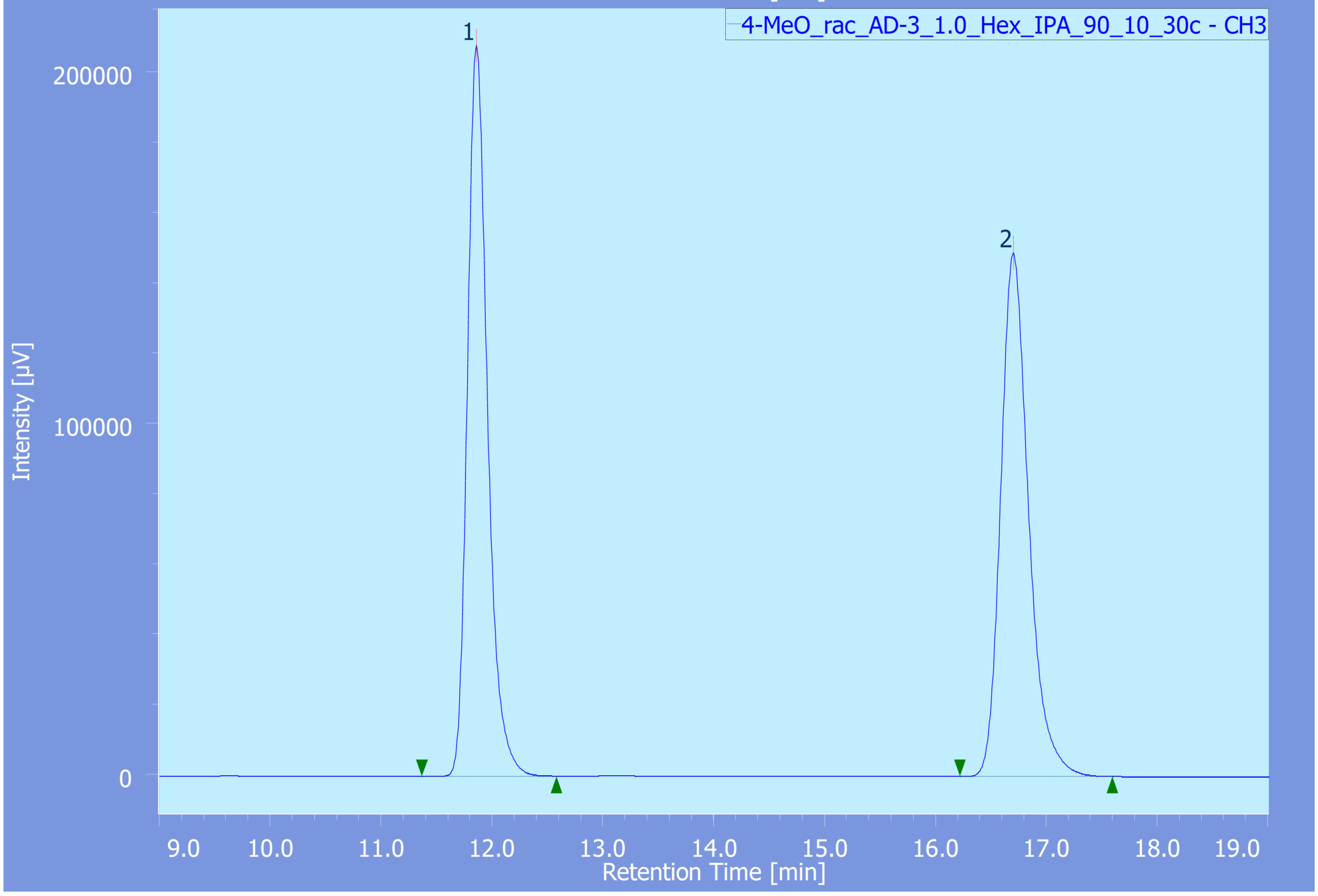

	Retention time (1)	Retention time (2)	% area (1)	% area (2)
rac-**4h**	11.858	16.700	49.945	50.055
**4h**	11.367	16.000	99.18	0.819

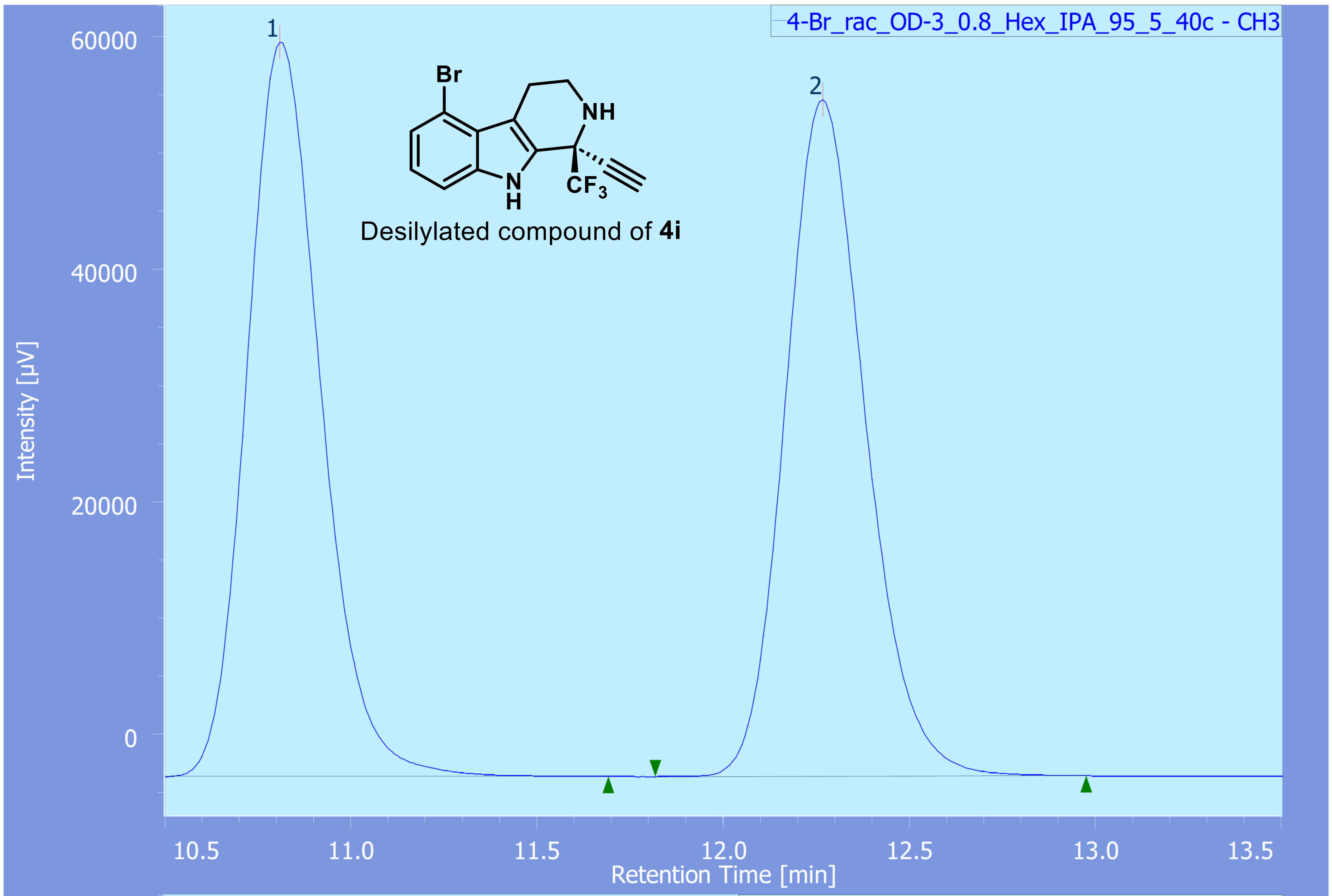

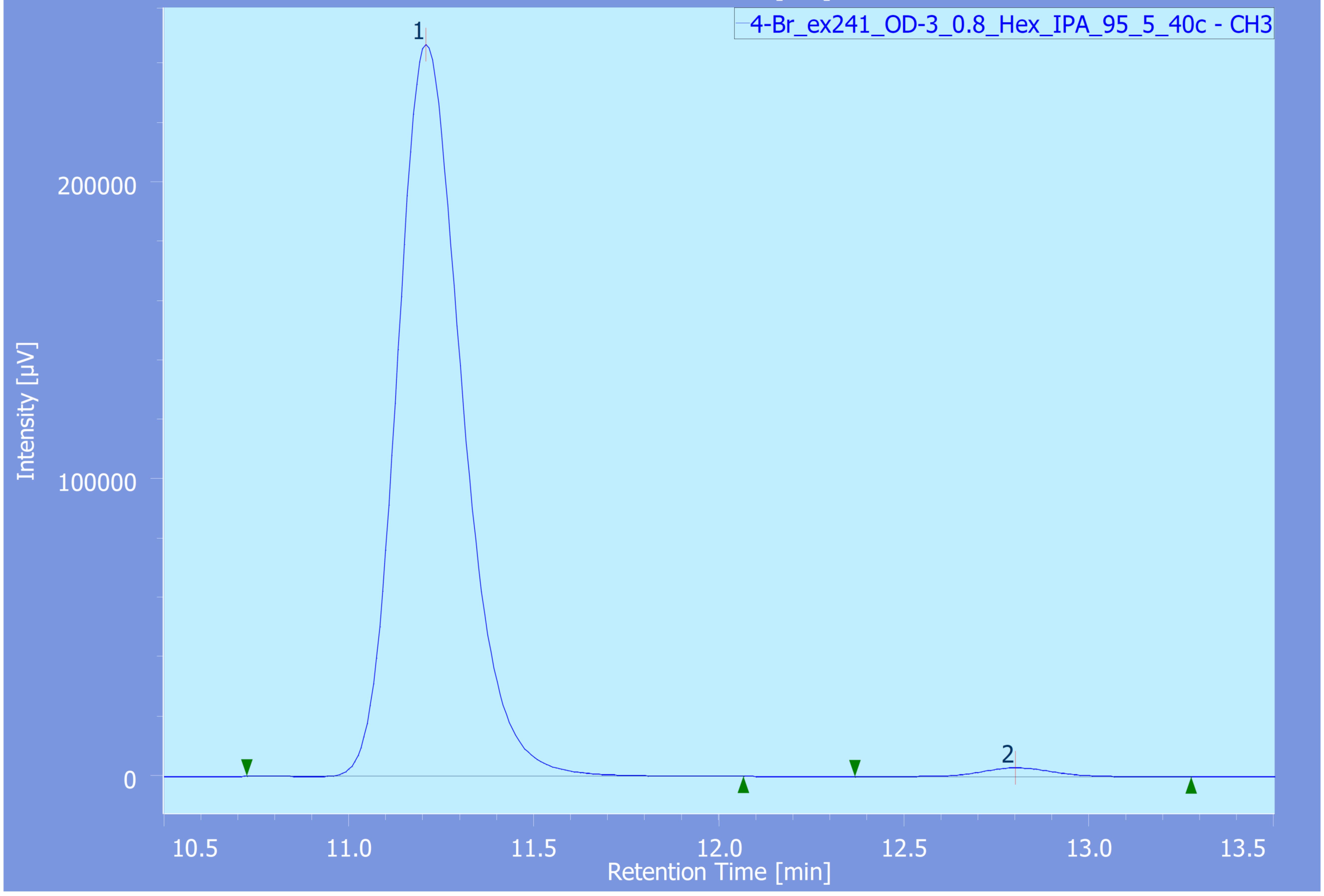

	Retention time (1)	Retention time (2)	% area (1)	% area (2)
rac-**4i**	10.808	12.267	50.113	49.887
**4i**	11.208	12.800	98.657	1.343

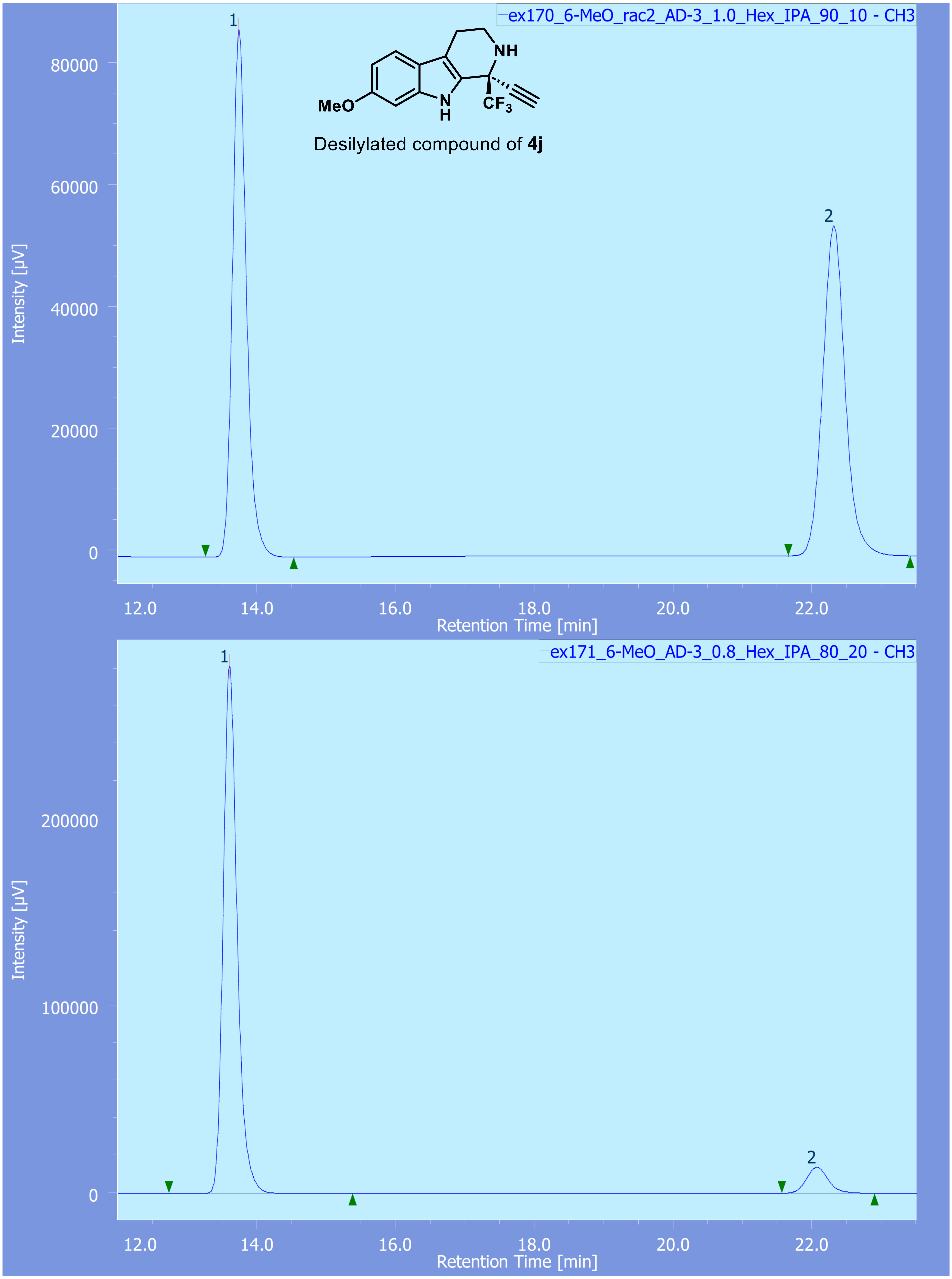

	Retention time (1)	Retention time (2)	% area (1)	% area (2)
rac-**4j**	13.742	22.317	49.946	50.054
**4j**	13.608	22.067	92.726	7.274

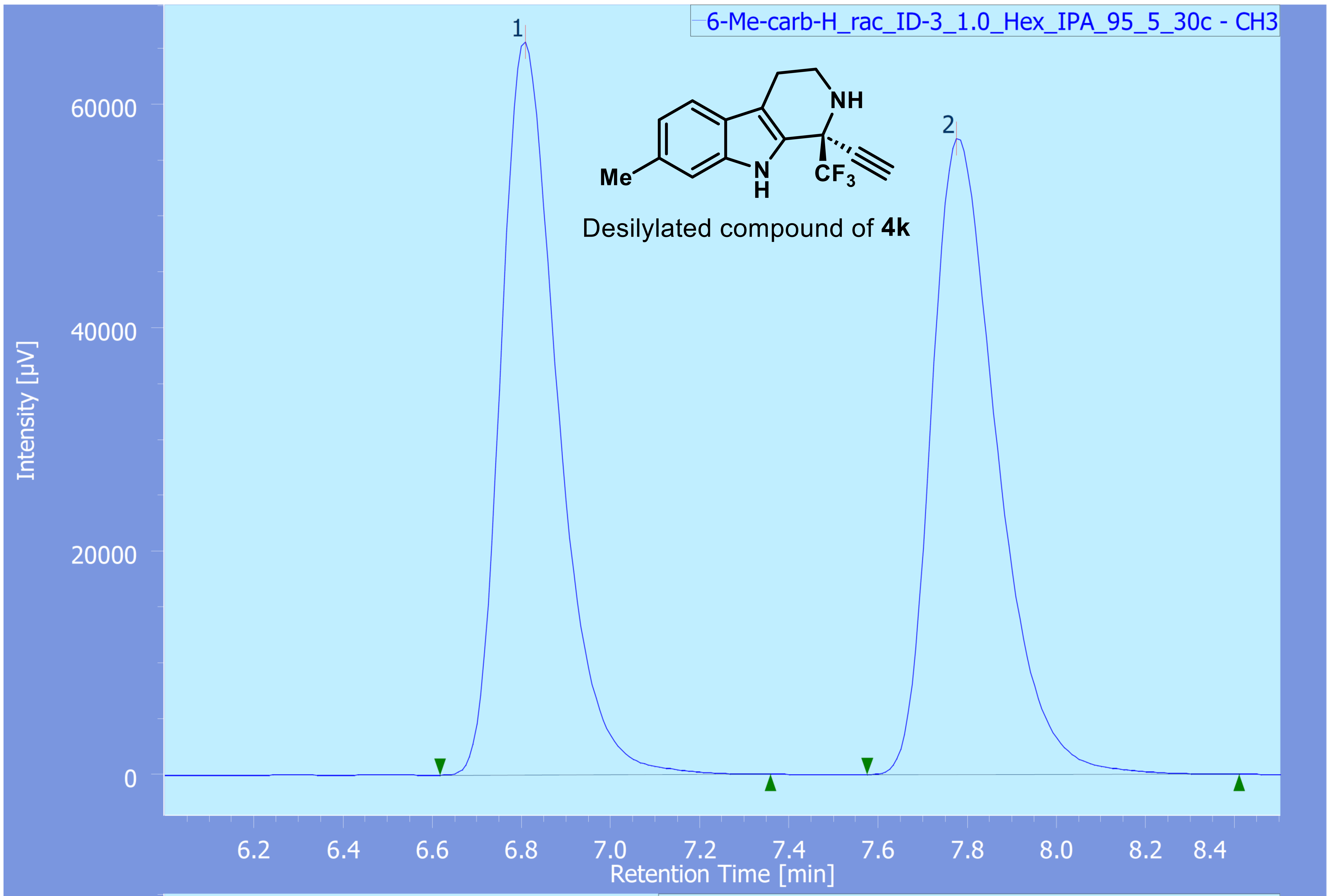

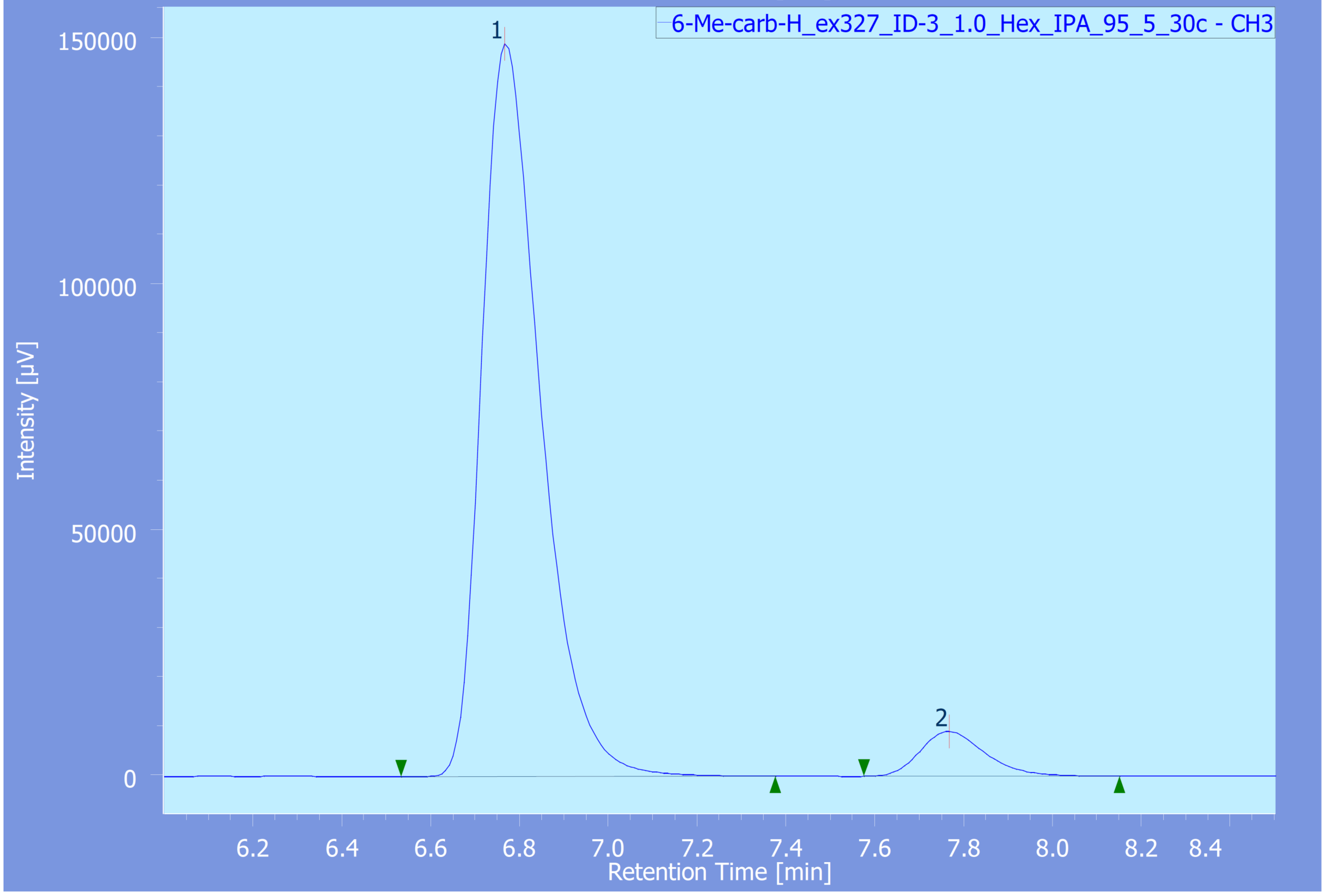

	Retention time (1)	Retention time (2)	% area (1)	% area (2)
rac-**4k**	6.808	7.775	49.967	50.033
**4k**	6.767	7.767	93.630	6.370

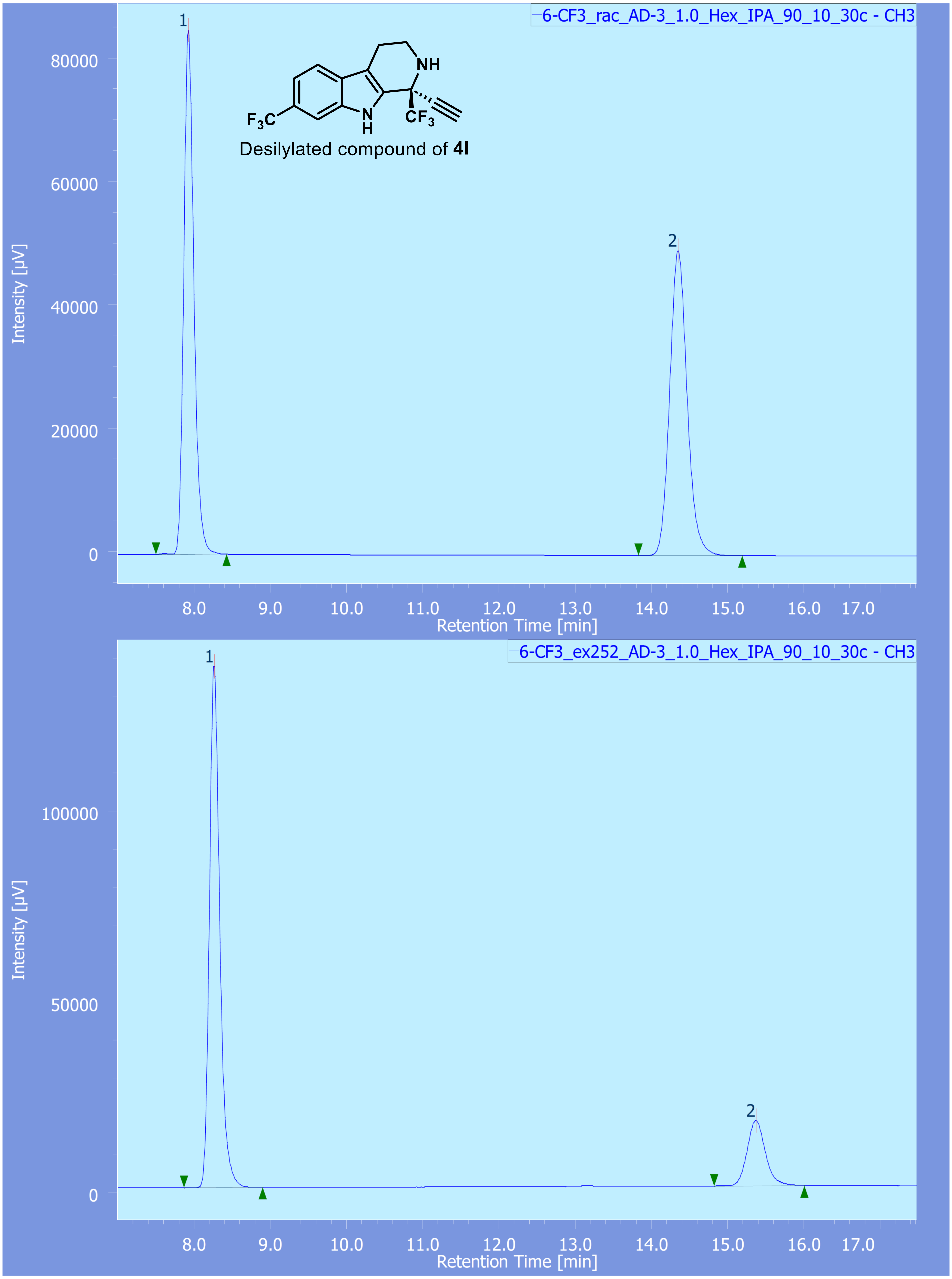

	Retention time (1)	Retention time (2)	% area (1)	% area (2)
rac-**4l**	7.925	14.342	49.945	50.055
**4l**	8.267	15.367	81.991	18.009

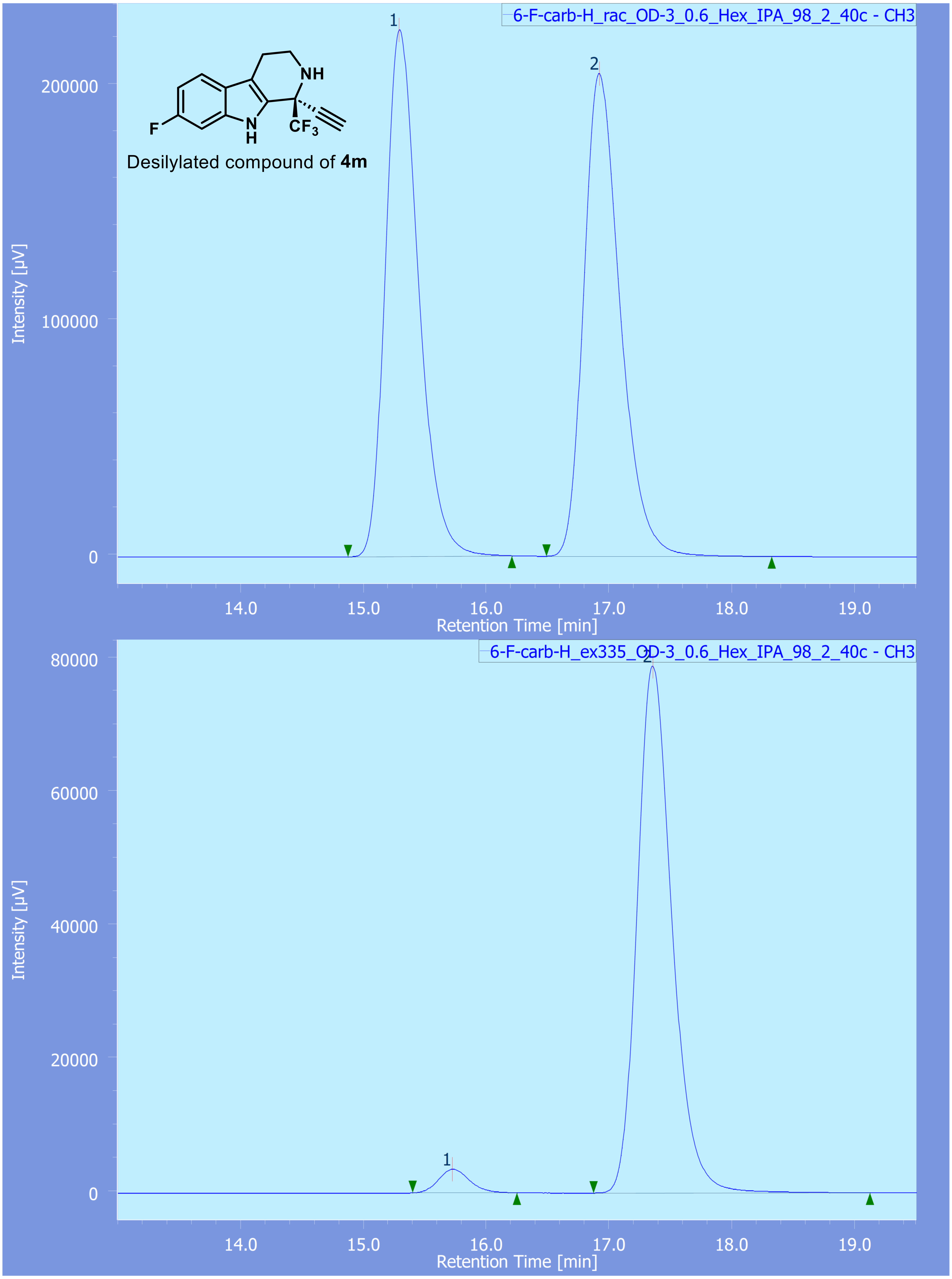

	Retention time (1)	Retention time (2)	% area (1)	% area (2)
rac-**4m**	15.292	16.925	49.903	50.097
**4m**	15.725	17.358	3.848	96.152

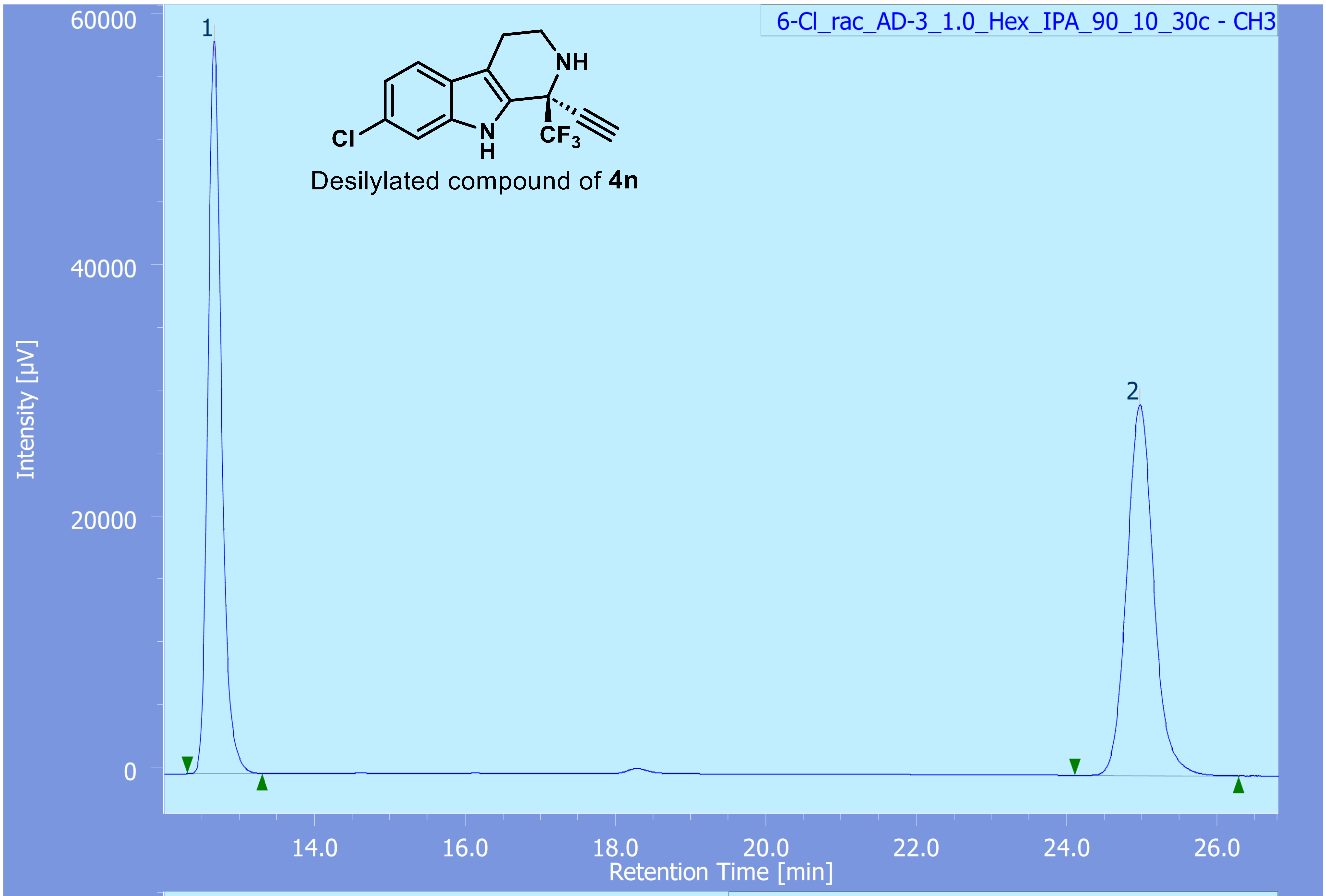

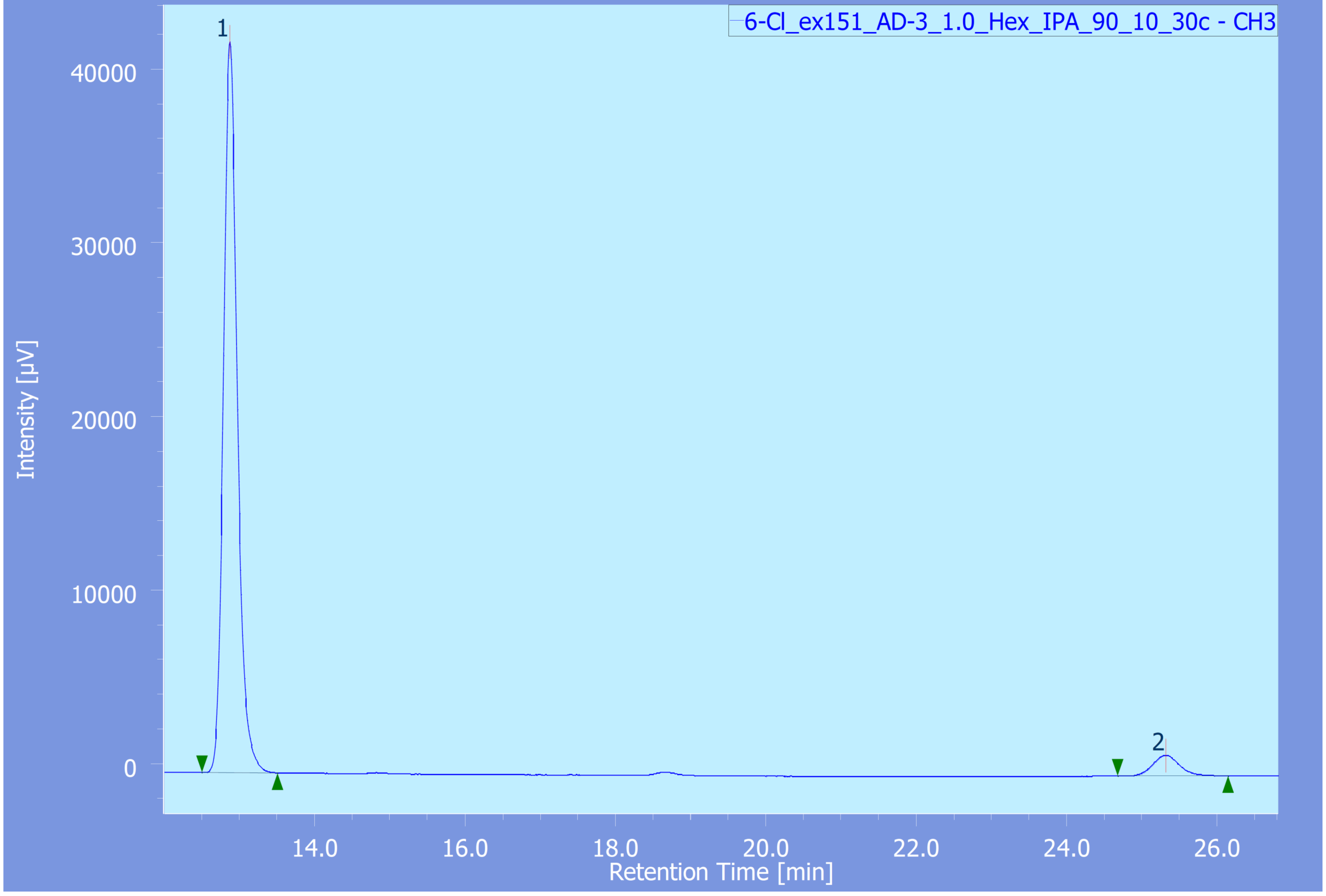

	Retention time (1)	Retention time (2)	% area (1)	% area (2)
rac-**4n**	12.667	24.967	49.879	50.121
**4n**	12.875	25.317	94.759	5.241

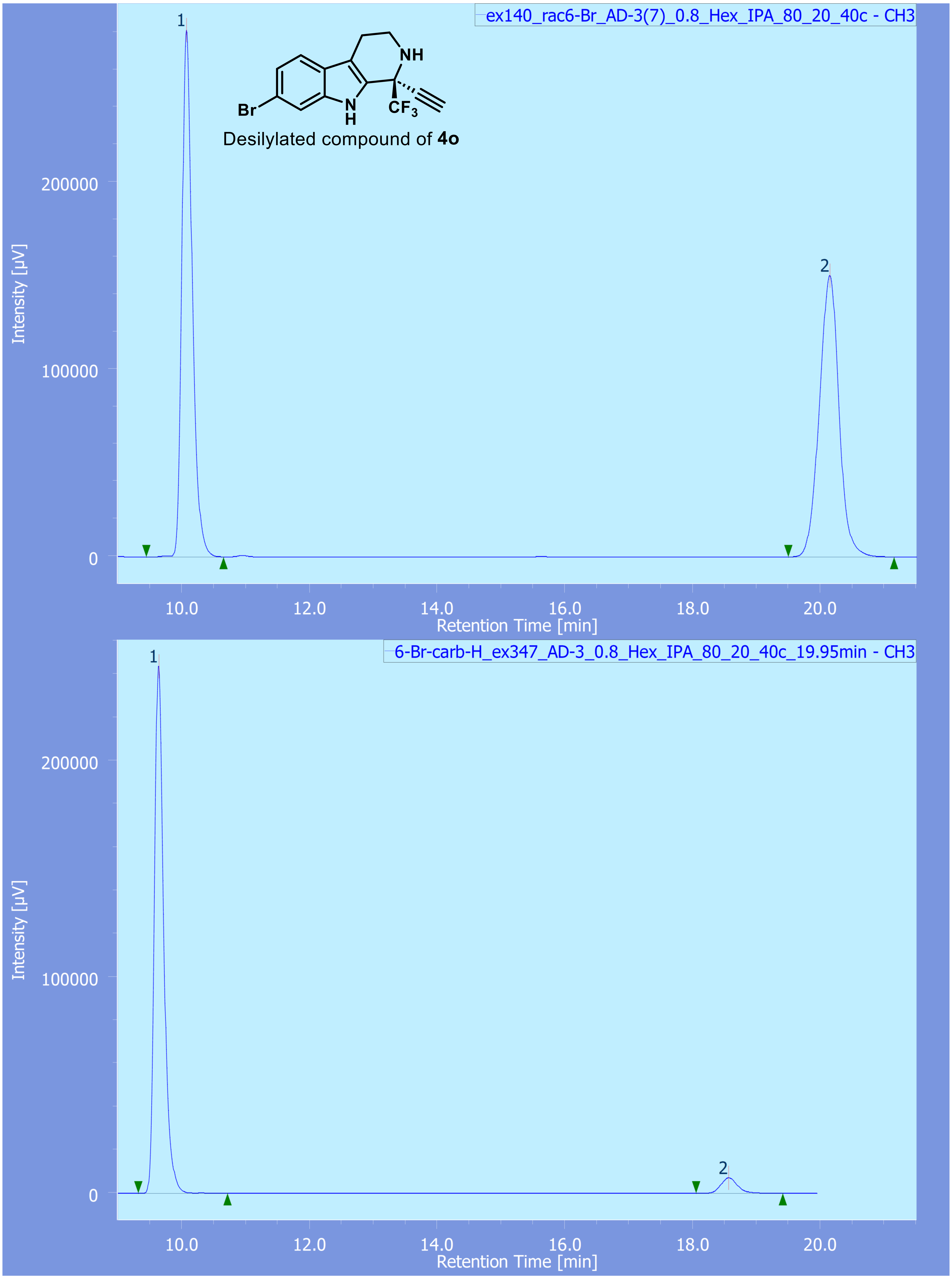

	Retention time (1)	Retention time (2)	% area (1)	% area (2)
rac-**4o**	10.075	20.150	49.866	50.134
**4o**	9.642	18.567	94.926	5.074

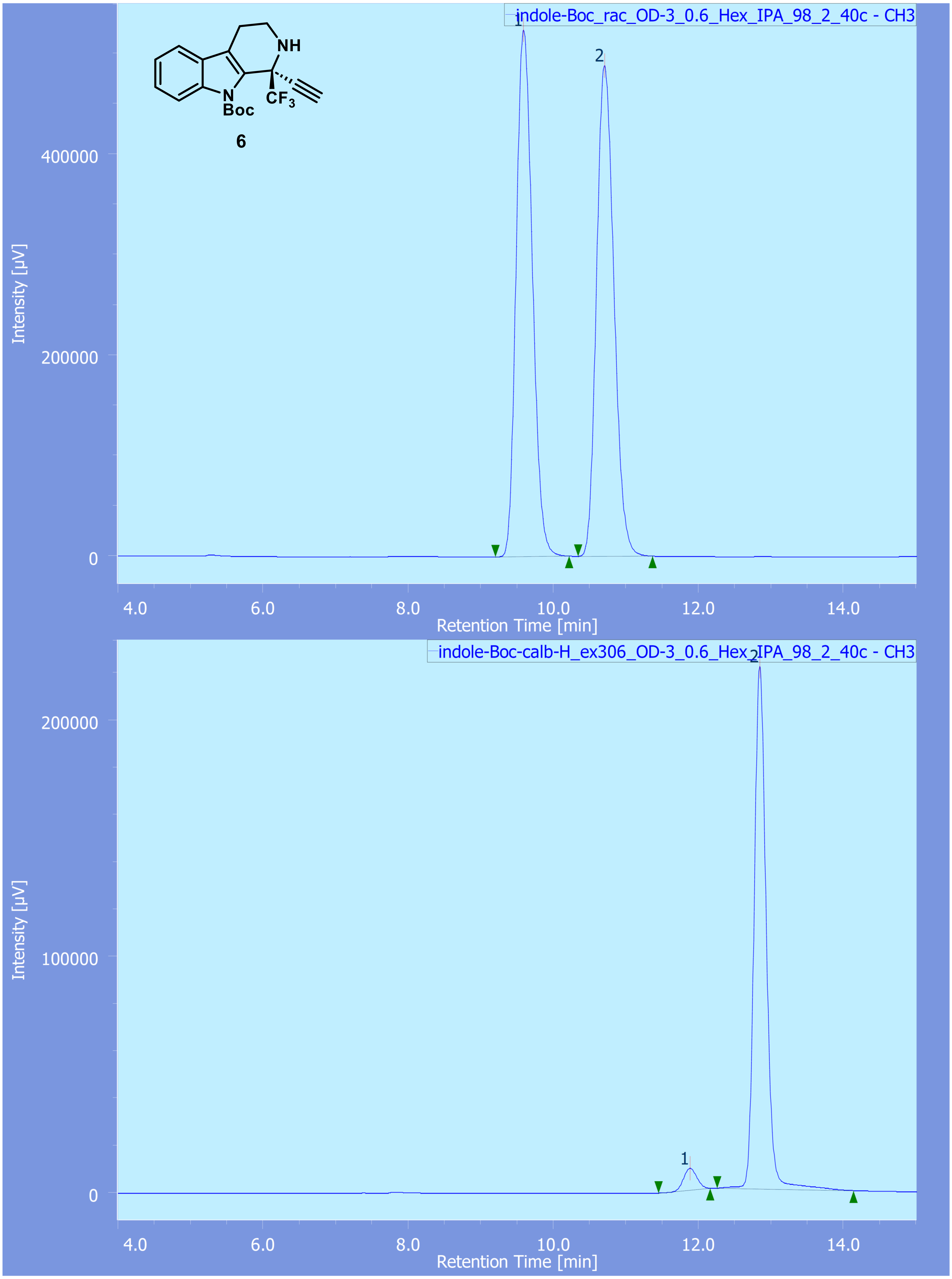

	Retention time (1)	Retention time (2)	% area (1)	% area (2)
rac-**6**	9.592	10.708	50.038	49.962
**6**	11.883	12.842	4.401	95.599

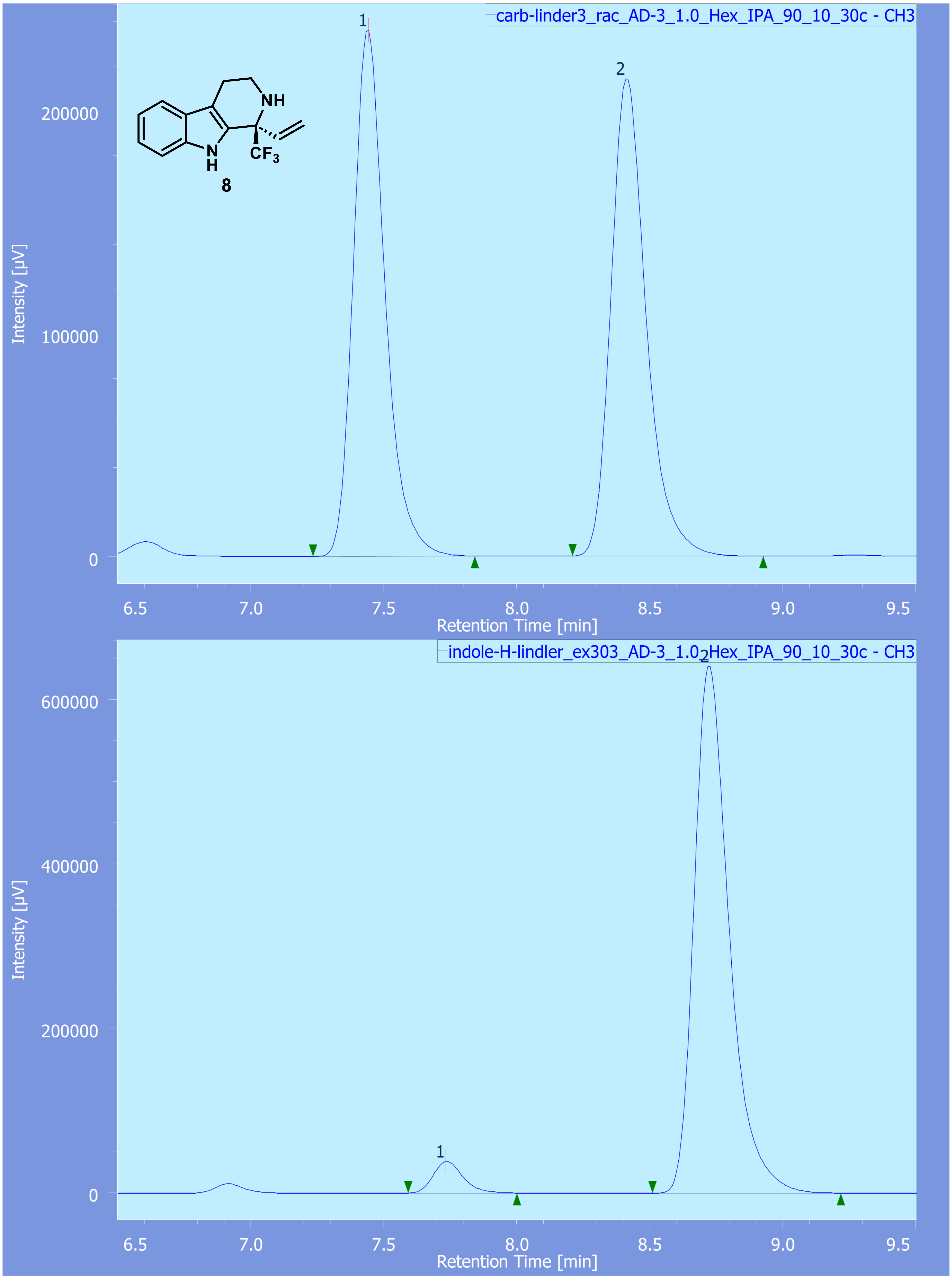

	Retention time (1)	Retention time (2)	% area (1)	% area (2)
rac-**8**	7.442	8.408	49.974	50.026
**8**	7.733	8.725	4.885	95.115

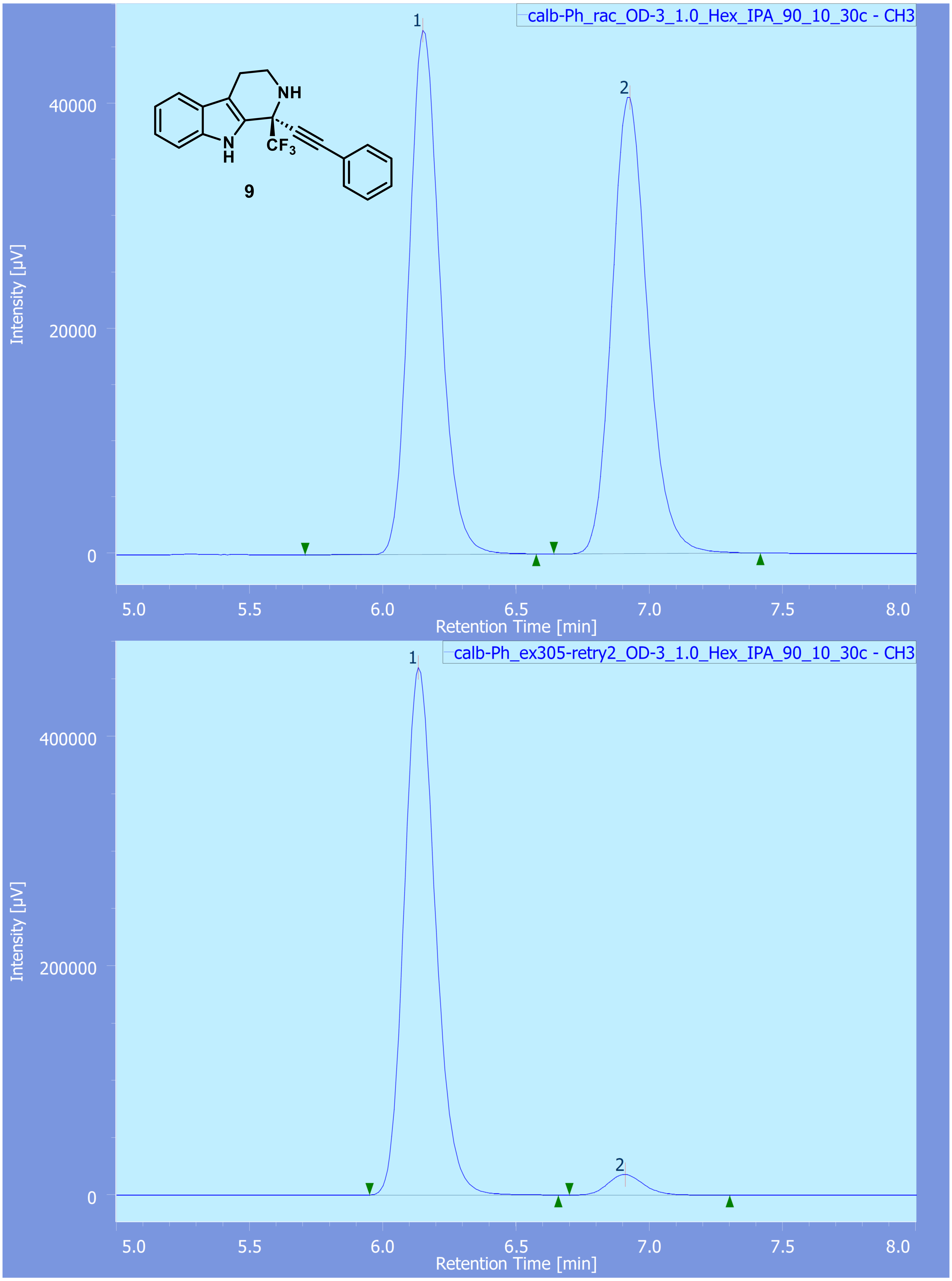

	Retention time (1)	Retention time (2)	% area (1)	% area (2)
rac-**9**	6.150	6.925	49.979	50.021
**9**	6.133	6.908	95.744	4.256

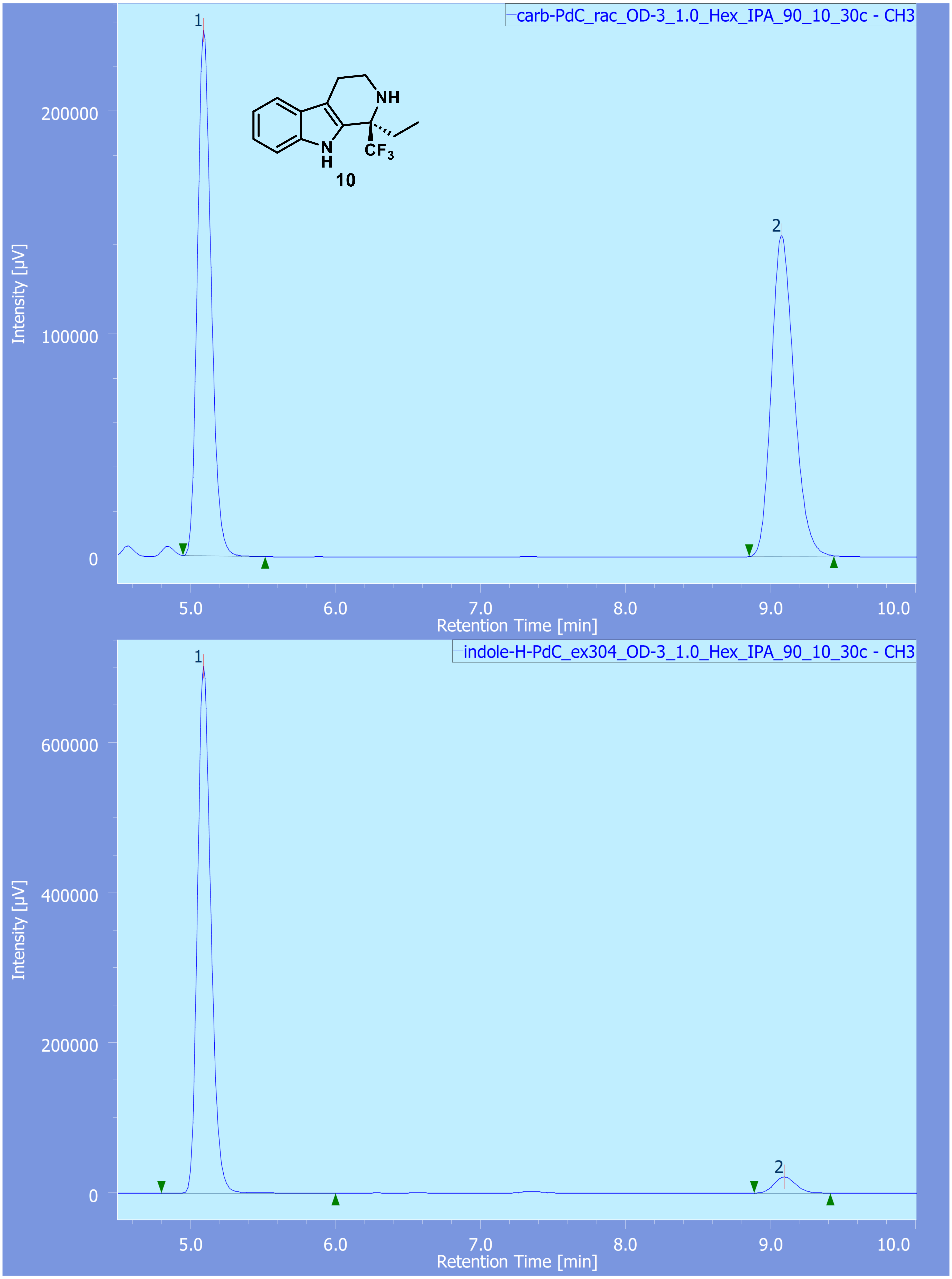

	Retention time (1)	Retention time (2)	% area (1)	% area (2)
rac-**10**	5.092	9.075	49.911	50.089
**10**	5.092	9.092	95.221	4.779

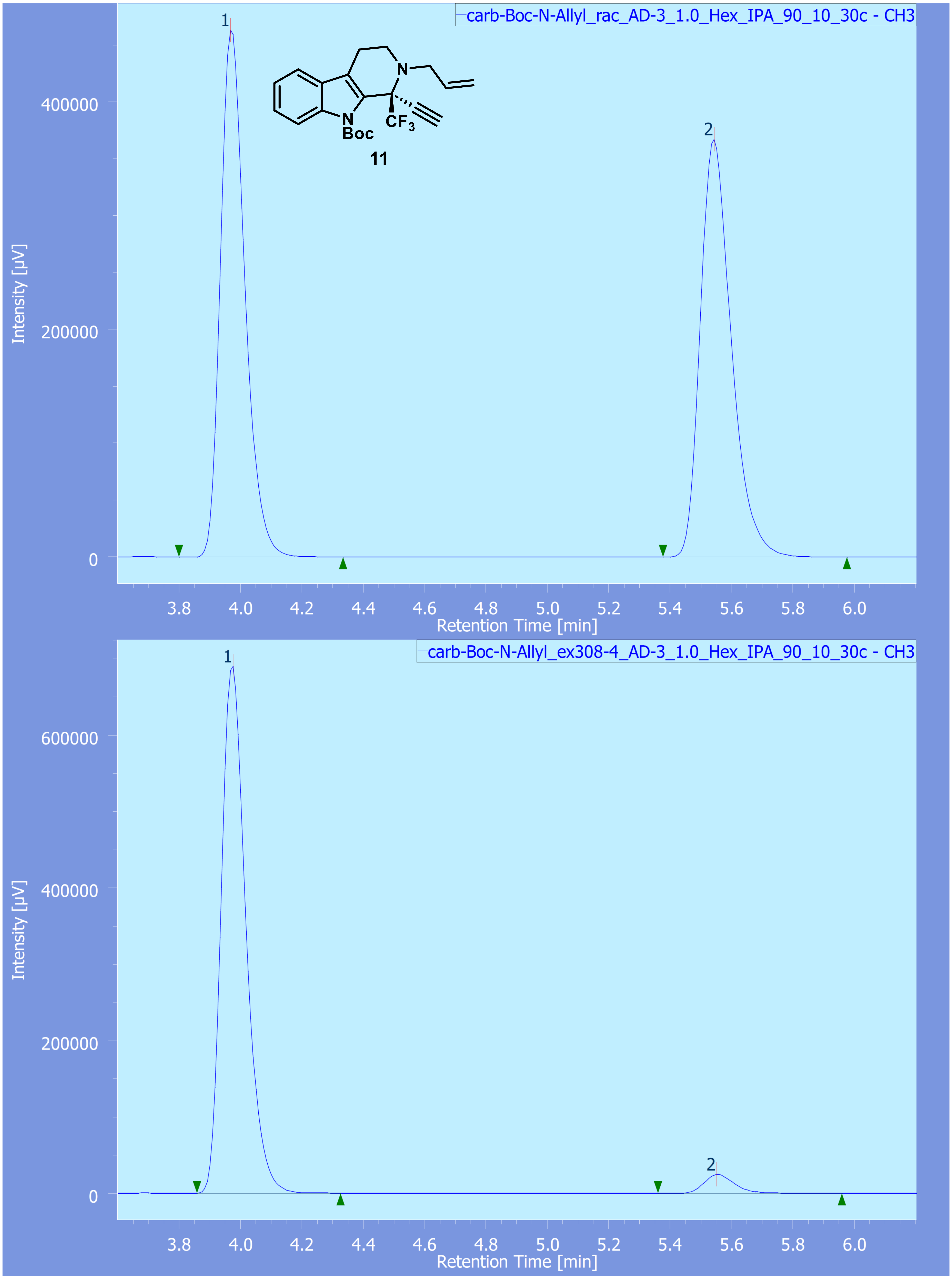

	Retention time (1)	Retention time (2)	% area (1)	% area (2)
rac-**11**	3.967	5.542	49.920	50.080
**11**	3.975	5.550	95.735	4.265

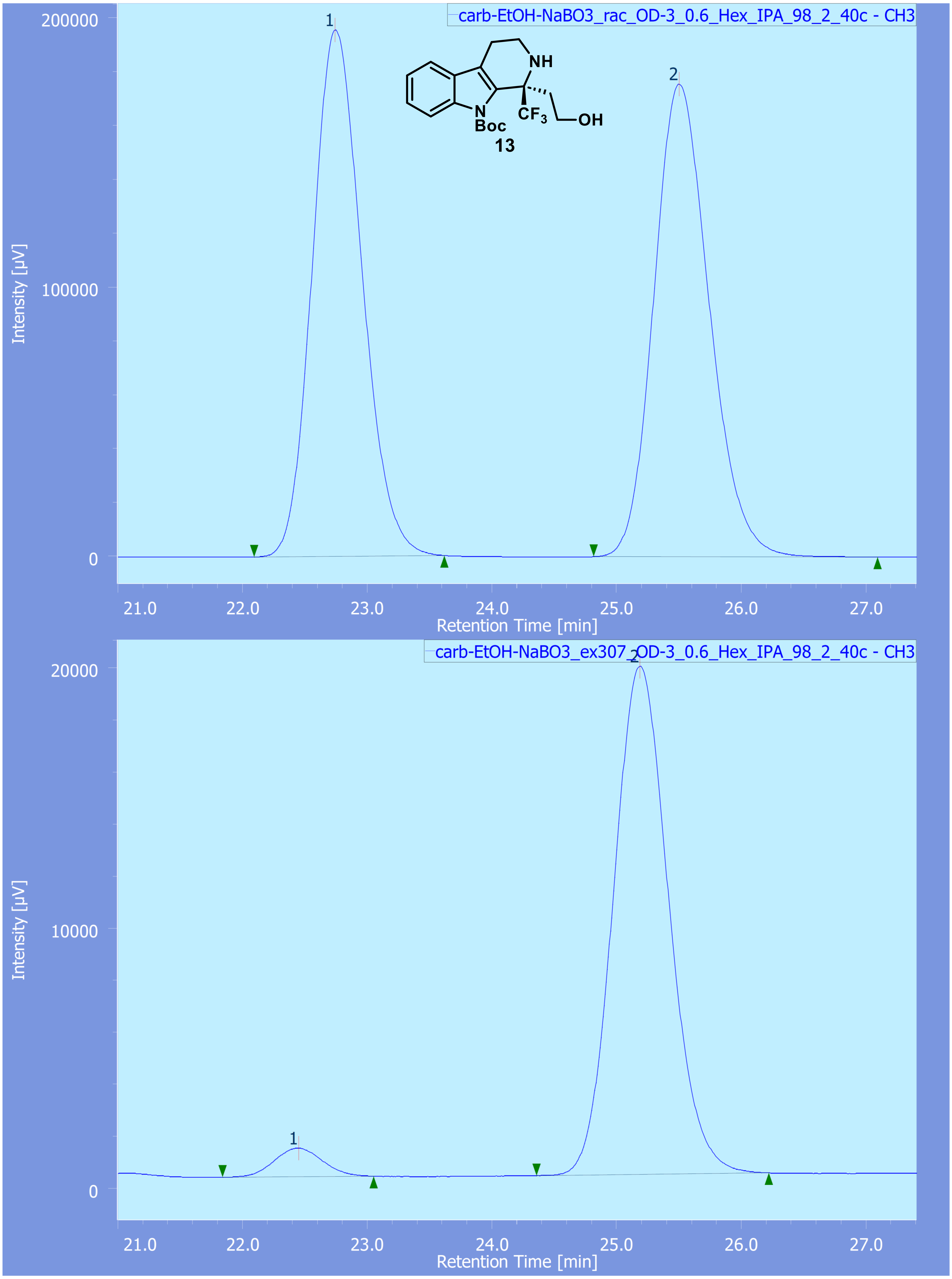

	Retention time (1)	Retention time (2)	% area (1)	% area (2)
rac-**13**	22.742	25.500	50.013	49.987
**13**	22.450	25.183	4.687	95.313

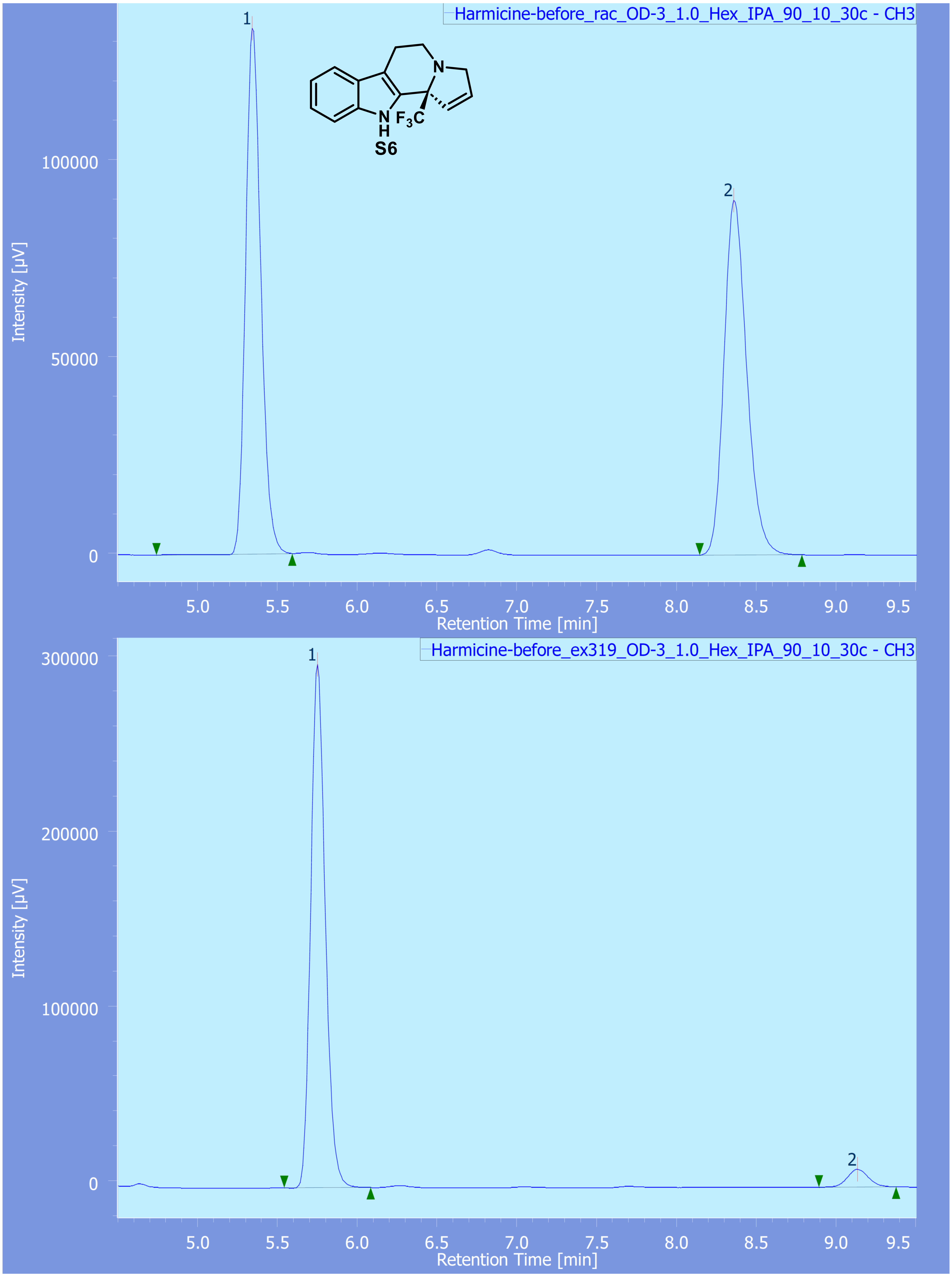

	Retention time (1)	Retention time (2)	% area (1)	% area (2)
rac-*S*10	5.342	8.358	49.821	50.179
***S*10**	5.750	9.133	95.304	4.696

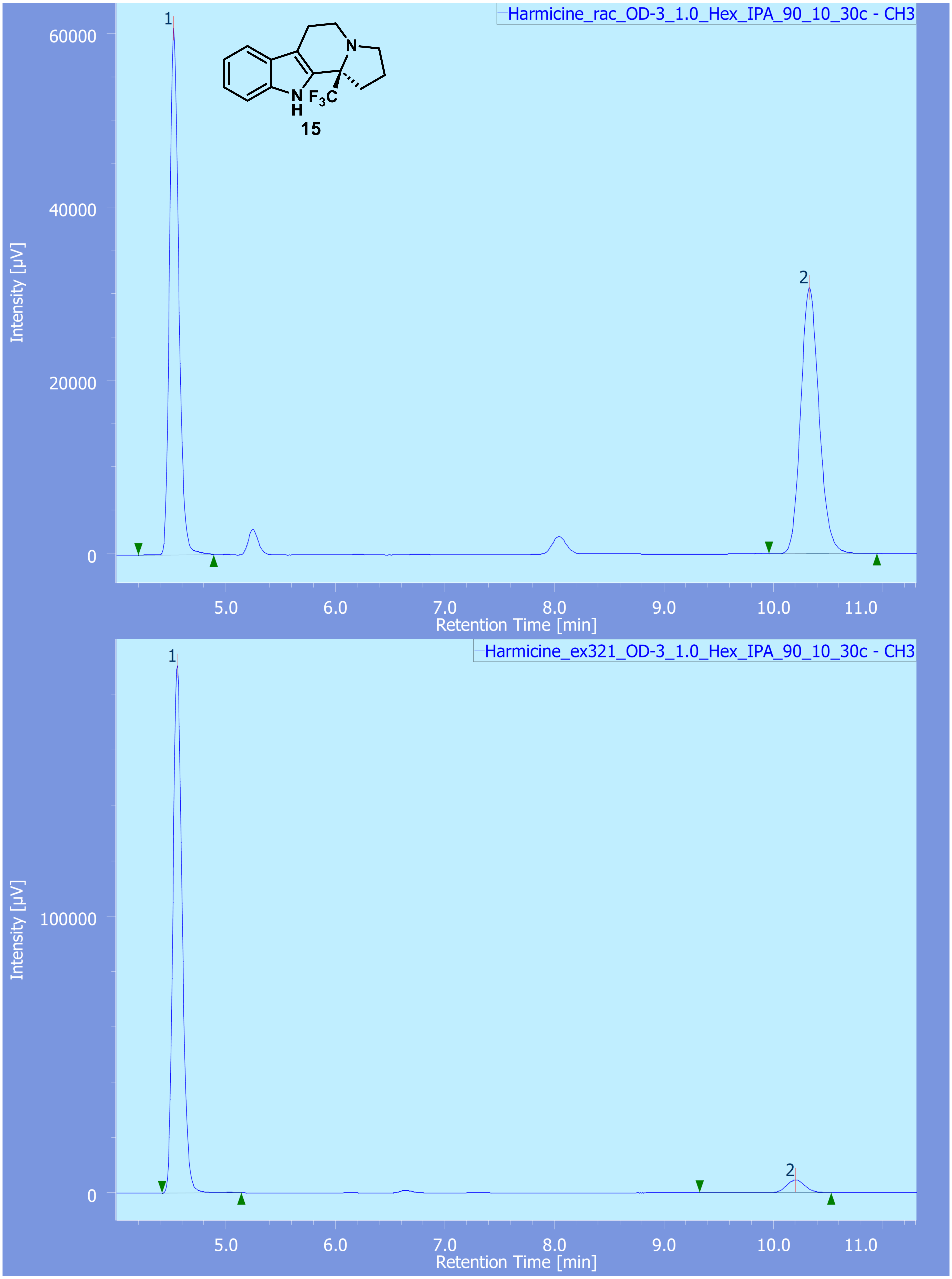

	Retention time (1)	Retention time (2)	% area (1)	% area (2)
rac-**15**	4.525	10.325	50.096	49.904
**15**	4.558	10.200	95.349	4.651
